# Synthetic Studies Towards Darobactin A

**DOI:** 10.1002/chem.202503359

**Published:** 2026-01-10

**Authors:** Till Steiner, Antoine Versini, Marco Schellenberg, Emma Schuler, Karl Gademann

**Affiliations:** ^1^ Department of Chemistry University of Zurich Zürich Switzerland

**Keywords:** darobactin, larock cyclization, natural products, polypeptide, total synthesis

## Abstract

We describe our efforts toward the total synthesis of darobactin A, an antibiotic that selectively targets Gram‐negative bacteria. The first route comprised the preparation of two separate advanced fragments. The unusual ether‐linked tryptophan moiety was constructed utilizing a nucleophilic aromatic substitution‐Bartoli indole synthesis‐Negishi coupling sequence, while C‐H arylation was used to prepare the β‐arylated lysine scaffold. Larock indole synthesis was confirmed as a valuable approach to achieve the first macrocyclization. An approach using macrolactamization for the construction of the second, western macrocycle proved to be unsuitable for the darobactin A scaffold. Thus, a second strategy focused on the synthesis of both macrocycles through two distinct intramolecular Larock indole syntheses that further brought valuable insights into the atroposelectivity of such reactions. Herein, we report our study toward the total synthesis of darobactin A, including the synthesis of advanced intermediates.

## Graphical abstract

The urgent need for new antibiotics led us to develop a new methodology for the synthesis of the natural product darobactin A selectively targeting gram‐negative bacteria. This study provides new insights into the synthesis of strained indole‐containing macrocycles. Key steps comprise a nucleophilic aromatic substitution–Bartoli indole synthesis–Negishi coupling alkylation sequence as well as a Larock indole macrolactamization.

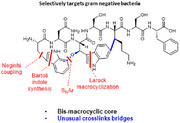

## 1 Introduction

The demand for novel antibiotic agents has increased in recent years in light of the antimicrobial resistance crisis, an issue regarded by the WHO as a major threat to global human health [1]. Many of the infections listed as most urgent are caused by Gram‐negative bacteria [2]. The development of novel antibiotics specifically targeting these multidrug‐resistant strains is therefore of utmost importance. Possessing a protective outer membrane, these pathogens traditionally represent very challenging targets and are usually treated with harsh broad‐spectrum antibiotics [3].

**FIGURE 1:**
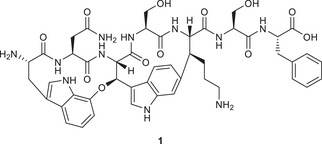
Molecular structure of darobactin A (**1**).

One potential novel antibiotic drug candidate is the ribosomally synthesized and post‐translationally modified heptapeptide natural product darobactin A (**1**, Figure 1), which was isolated from *Photorhabdus khanii* [4, 5, 6]. Due to its highly strained bis‐macrocyclic core, it adopts a β‐sheet‐like structure, enabling binding to the BamA complex, located on the outer membrane of Gram‐negative bacteria [7, 8]. Consequently, outer membrane proteins are no longer folded and integrated properly into the outer membrane, therefore impairing its function. This unique mechanism of action offers the prospect of specifically targeting Gram‐negative pathogens and makes darobactin A (**1**) an attractive lead compound for the development of a novel class of antibiotics. This was further underlined by recent studies on the heterologous expression of **1** and nonnatural analogues, furnishing novel ‘darobactins’ with increased potency [9, 10, 11, 12, 13].

While heterologous expression proved to be a valuable platform to produce darobactin analogues by incorporation of various natural amino acids, chemical synthesis could potentially offer the advantage of including a larger variety of structural motifs. Therefore, the development of a total synthesis for darobactin A (**1**) could not only provide more material for biological studies but would also offer the basis for the preparation of structurally diverse analogues. In addition, the development of such syntheses could give valuable insight into the construction of highly strained, indole‐containing macrocycles. Successful total syntheses have recently been achieved independently by the Sarlah group (in collaboration with Merck, Sharp & Dohme) and the Baran group [14, 15]. Their work highlighted the synthetic challenges posed by target molecule **1**.

The most notable structural features of darobactin A (**1**) are the two unusual macrocyclic crosslinks: an alkyl‐aryl ether bond between the C7 and β‐position of Trp^1^ and Trp^3^ forming the western macrocycle, and a C–C bond between C6 of Trp^3^ and the β‐position of Lys^5^ forming the eastern macrocycle. The resulting strain leads to the emergence of atropisomerism due to hindered rotation of the indole moieties. Therefore, the design of a synthetic route to access darobactin A (**1**) should be focused on stereoselective methods for the formation of the desired crosslinks and strategies allowing for atroposelectivity. Herein, we report various strategies toward the preparation of darobactin A (**1**), taking these key points into consideration. Several approaches to reach advanced intermediates were investigated and will be presented in this study, thus increasing chemical knowledge on this intriguing natural product.

**SCHEME 1:**
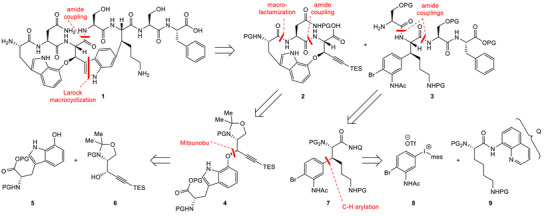
First‐generation retrosynthetic analysis of darobactin A (**1**).

Our retrosynthetic analysis was designed with the aim of reaching a convergent synthesis. We envisioned the two advanced intermediates **2** and **3**, and to achieve the desired macrocyclization of the eastern macrocycle by intramolecular Larock indole synthesis at a late stage of the synthesis, a concise and elegant approach independently also reported by Sarlah and Baran (Scheme 1) [14, 15].

The macrocycle of western fragment **2** would be obtained through a macrolactamization reaction. The crucial ether bridge in intermediate **4** was envisioned to be constructed employing a Mitsunobu reaction in a stereoinvertive fashion between hydroxylated tryptophan derivative **5** and propargylic alcohol **6**. The preparation of both fragments had previously been reported [16, 17, 18]. Regarding the synthesis of the eastern fragment **3**, the strategy would be focused on the preparation of the β‐arylated lysine derivative **7**. The key step of this sequence was envisioned to be a C‐H arylation of lysine derivative **9**, featuring an aminoquinoline directing group, and utilizing iodonium salt **8** as the reaction partner. This methodology was previously applied efficiently by the Boger group on a lysine‐based substrate in their synthesis of streptide [19].

## 2 Results and Discussion

**SCHEME 2:**
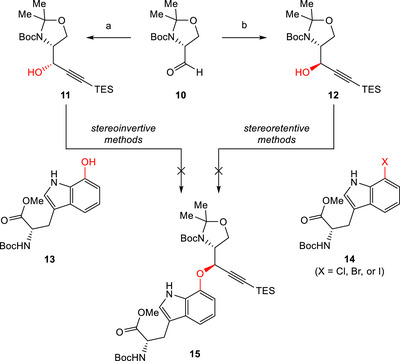
Reagents and conditions: (a) TES‐acetylene, *n*BuLi, HMPA, THF, −78°C, 2 h, 86%, d.r. >25:1; (b) TES‐acetylene, EtMgBr, THF, reflux, 2 h, *then* **10**, CuI, SMe_2_, THF, −78°C to rt, 22 h, 79%, d.r. >25:1.

Our efforts commenced with the preparation of propargylic alcohol **11**, which already comprised the alkyne moiety, which would later be used to construct the eastern macrocycle in an intramolecular Larock indole synthesis. The versatile chiral building block d‐Garner's aldehyde (**10**) [20, 21] served as the starting material (Scheme 2). The oxazolidine ring in **10** served both as a bifunctional protecting group and as a guiding scaffold, allowing for the stereoselective introduction of nucleophiles. Utilizing previously reported conditions for nucleophilic additions to **10** [16], we were able to obtain propargylic alcohol **11** and its diastereomeric counterpart **12** with excellent stereoselectivity. The known 7‐hydroxy tryptophan derivative **13** was prepared in three steps from a commercially available tryptophan precursor [18, 22].

Unfortunately, the desired product **15** could not be obtained after subjecting propargylic alcohol **11** and phenol **13** to Mitsunobu conditions (see SI‐Table 1). While hydroxy‐Trp derivative **13** reacted smoothly with simple secondary alcohols as test substrates to form alkyl‐aryl ethers, propargylic alcohol **11** did not show the desired reactivity even with simpler phenolic nucleophiles. Furthermore, we noticed that alcohol **11** was prone to decomposition at elevated temperatures. Similar observations were made when we investigated alternative stereoinvertive methodologies for the construction of alkyl‐aryl ethers, such as oxidation‐reduction protocols reported by Mukaiyama and coworkers [23]. alternatively employing PhenoFluor as shown by Ritter and coworkers [24], or engaging the corresponding mesylated or tosylated analogues to substitution conditions (see SI‐Scheme 1a). In order to activate the propargylic position, we considered Nicholas reaction conditions (see SI‐Scheme 1b) [25, 26]. While the cobalt complex of **11** and cobalt octacarbonyl was obtained, the desired product **15** was not formed upon treatment with different Lewis acids typically employed in such reactions.

After extensively studying stereoinvertive methods for the preparation of ether **15** (Scheme 2, left), we then focused on stereoretentive alternatives leveraging alcohol **12** (Scheme 2, right). For this purpose, tryptophane derivatives **14**, halogenated at the 7‐position, were synthesized following literature‐known procedures [18]. First, we investigated the possibility of using these substrates in Ullmann‐type copper‐catalyzed cross‐couplings [27, 28, 29, 30, 31]. It became apparent that propargylic alcohol **12** also decomposed at the elevated temperatures typically used in such reactions. In addition, no product formation could be observed either when substrates **12** and **14** were subjected to electrocatalytic conditions as reported by Baran and coworkers (see SI‐Scheme 1c) [32].

**SCHEME 3:**
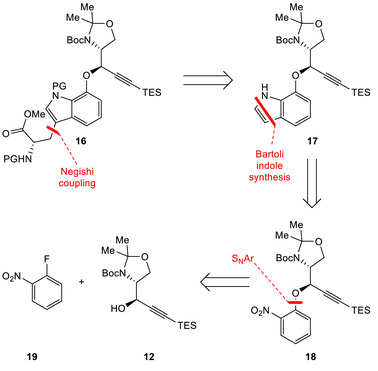
Revised retrosynthetic analysis of ether fragment **16**.

At this point it became apparent that our initial plan for the preparation of the desired ether fragment **15** would need to be revised. We turned our attention to the use of nucleophilic aromatic substitution (S_N_Ar), a classical method often utilized for the preparation of alkyl‐aryl ethers and typically approached with fluorinated nitrobenzenes [33, 34, 35]. This created the task of designing a synthesis for the tryptophan moiety in **16**. As depicted in Scheme 3, our revised approach anticipated the introduction of the alkyl chain *via* Negishi cross‐coupling [36]. We then envisioned constructing indole **17** through Bartoli indole synthesis [37] from nitrobenzene **18** which would be reached from propargylic alcohol **12** after S_N_Ar with 2‐fluoronitrobenzene (**19**).

**SCHEME 4:**
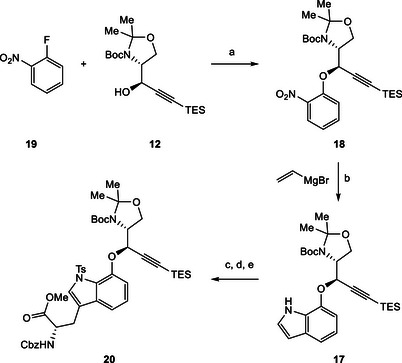
Reagents and conditions: (a) NaHMDS, THF, −40°C, 3 h, 94%; (b) vinylmagnesium bromide, THF/dioxane (3:1), −40°C, 3 h, 41%; (c) Br_2_, DMF, 0°C, 2 h, 87%; (d) TsCl, NaOH, BnNEt_3_Cl, CH_2_Cl_2_, rt, 6 h, 72%; (e) Zn, 1,2‐dibromoethane, DMF, 50°C, 30 min, *then* TMSCl, rt, 30 min, *then* N^α^‐Cbz‐3‐iodo‐l‐alanine methyl ester, rt, 2 h, *then* s.m., Pd(OAc)_2_, S‐Phos, 50°C, 85%.

To our delight, propargylic alcohol **12** proved to be a suitable substrate for S_N_Ar with *O*‐fluoronitrobenzene (**19**), delivering the desired ether product **18** in excellent yield (Scheme 4). The treatment of nitrobenzene **18** with vinylmagnesium bromide afforded the desired indole **17**. Crucial for this Bartoli indole synthesis was the choice of the solvent. While only small amounts of product were formed in pure THF or mixtures of THF with diethyl ether or MTBE, we were able to isolate **17** in a yield of 41% when a 3:1 mixture of THF and dioxane was employed (see SI‐Table 2). Taking the comparatively complex nature of the substrate into consideration, this outcome was deemed satisfactory [38].

We continued our synthesis by selective bromination at the C‐3 position of indole **17** followed by nitrogen protection with a tosyl group [39]. In the following step the resulting brominated and protected substrate was engaged with *N^α^*‐Cbz‐3‐iodo‐l‐alanine methyl ester (prepared in one step) [40] in a Negishi cross‐coupling reaction utilizing conditions previously reported for indole‐based substrates [36], thus delivering the desired fragment **20** in excellent yield. We also evaluated replacing the tosyl group with acetyl protection, which would allow the use of milder deprotection conditions later in the synthesis. Unfortunately, this modification was found to be incompatible with the employed Negishi conditions. Although nosyl protection could offer advantages in terms of deprotection, the tosylated indole was selected as the substrate of choice for its robustness and well‐established compatibility with the Negishi reaction conditions.

**SCHEME 5:**
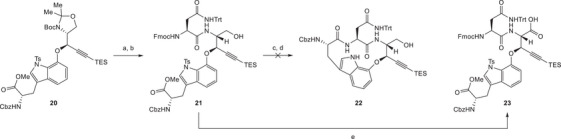
Reagents and conditions: (a) TFA, CH_2_Cl_2_, rt, 30 min; (b) *N* ^α^‐Fmoc‐ *N* ^γ^‐Trt‐l‐asparagine, HATU, DIPEA, DMF, rt, 16 h, 91% over two steps; (c) Me_3_SnOH, DCE, 50°C, 2 d, *then* piperidine, rt, 30 min; (d) various macrolactamization conditions (see SI‐Table 3); (e) TEMPO, PIDA, NaClO_2_, MeCN, aq. phosphate buffer (pH = 6.4), rt, 16 h, 55%.

Next, the oxazolidine moiety and the Boc group of fragment **20** were cleaved off under acidic conditions (Scheme 5) [17]. The resulting primary amine was subjected to an amide coupling reaction with a protected asparagine derivative to afford intermediate **21**. Before tackling the key macrolactamization step, the Fmoc group had to be removed along with hydrolysis of the methyl ester. Initial attempts were hampered by concomitant TES deprotection. Finally, this could be avoided by slow ester hydrolysis with trimethyltin hydroxide [41] followed by Fmoc removal with piperidine. The resulting intermediate was then directly engaged in macrolactamization trials. To our dismay, these remained unsuccessful during studies with different coupling agents, bases, and solvents (see SI‐Table 3). Although no clean products were isolated, we suspected dimerization to be the main side reaction that prohibited any formation of the desired product (**22**). Similar observations were also made by Sarlah and coworkers during their investigations on darobactin A (**1**) [14].

We speculated at this point that the spatial pre‐arrangement of the amine and the carboxylic acid involved in such a macrolactamization approach might be improved by employing a more constrained substrate. For that reason, we turned our attention to an advanced intermediate in which the other macrocycle would already be in place. Therefore, we delayed this crucial macrocylization step to the very end of our synthesis after attachment to the eastern fragment **3** and after closure of the central macrocycle. Hence, we oxidized the primary hydroxy group in **21** to the carboxylic acid, obtaining revised western fragment **23**.

**SCHEME 6:**
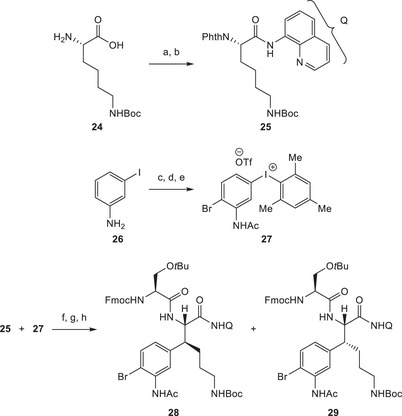
Reagents and conditions: (a) *N*‐ethoxycarbonyl phthalimide, Na_2_CO_3_, H_2_O, rt, 20 h; (b) 8‐aminoquinoline, HATU, DIPEA, DMF; rt, 16 h, 80% over two steps; (c) NBS, benzene, rt, 1 d, 28–47%; (d) Ac_2_O, DMAP, toluene, reflux, 3 h, 98%; (e) *m*CPBA, TfOH, CH_2_Cl_2_, rt, 2 h, *then* mesitylene, rt, 20 h, 96%; (f) Pd(OAc)_2_, AgOAc, 3Å MS, *t*BuOH, 80°C, 48%, d.r. = 1:4 to 1:6; (g) H_2_NNH_2_ ∙ H_2_O, MeOH, rt, 16 h; (h) *N* ^α^‐Fmoc‐ O‐*t*Bu‐l‐serine, HATU, NMM, DMF, rt, 23 h, 85% over two steps, d.r. = 1:4.

As depicted in Scheme 6, the synthesis of the eastern fragment **3** of darobactin A (**1**) commenced with the preparation of substrate **25**. Commercially available *N^ε^*‐Boc‐lysine (**24**) was first protected as a phthalimide, and subsequently an aminoquinoline was installed utilizing slightly altered literature conditions [18]. In addition, iodonium salt **27** was prepared in three steps from 3‐iodoaniline (**26**) following literature precedents [42, 43]. The two substrates were then subjected to the C‐H arylation conditions already employed by Boger and coworkers in their synthesis of streptide [19]. After some adjustments, including the use of molecular sieves, the initially low yields could be improved to an acceptable 48%, with the regioselectivity being dictated by the aminoquinoline directing group. However, analysis by NMR quickly led to the conclusion that a diastereomeric mixture had formed. At this stage, the two isomers were not separable by various methods. Thus, the synthesis continued with phthalimide removal and subsequent coupling with a protected serine derivative. To our delight, at this stage, the two resulting diastereomers, **28** and **29**, were separable by column chromatography. To our dismay, a 4:1 mixture in favor of undesired isomer **29** was obtained. Investigations of different reaction conditions indicated that this ratio could not be improved and was dictated by the stereochemical nature of substrate **25**.

The configuration of **28** and **29** was assigned based on the published data from Boger and coworkers, where they have obtained a crystal structure for a derivative of the favored isomer [19]. Extensive 2D‐NMR analysis, including NOESY, of both **28** and **29** did not result in conclusive evidence. This initial assignment was corroborated later using NOESY analysis of cyclized compounds **35** and **36** (Scheme 8) obtained from the minor diastereoisomer **28**.

To make use of this substrate‐dictated outcome we also investigated the possibility of performing a consecutive C‐H arylation/C‐H alkylation approach, installing the aryl moiety first, followed by the introduction of the lysine alkylamine side chain. While C‐H arylations were successful, C‐H alkylations remained fruitless (see SI‐Scheme 2). Alternative approaches for the construction of the desired arylated lysine motif that we explored included asymmetric cinnamylations [44, 45, 46], asymmetric Strecker reactions [47, 48], asymmetric reductive amination [49], and a conjugate addition‐bromination‐azidation sequence (see SI‐Scheme 3) [50]. As none of these endeavors brought the desired breakthrough, we continued our investigations with intermediate **28** obtained from the initial C‐H arylation approach.

**SCHEME 7:**
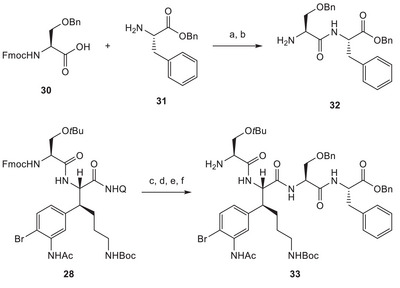
Reagents and conditions: (a) HATU, NMM, DMF, rt, 16 h, quant.; (b) piperidine, DMF, rt, 30 min; (c) O_3_, pyridine, CH_2_Cl_2_, −78°C, 15 min; (d) LiOH ∙ H_2_O, H_2_O_2_, THF/H_2_O, 0°C, 2 h; 33%; (e) **32**, HATU, DIPEA, DMF, rt, 16 h, 88%; (f) piperidine, DMF, rt, 30 min.

Starting from amino acid building blocks **30** and **31**, the suitably protected version of darobactin A's dipeptide side chain **32** was prepared in two steps (Scheme 7). Before coupling this fragment **32** to our previously prepared lysine‐based intermediate **28**, hydrolytic removal of the aminoquinoline was investigated. Albeit being fairly low yielding, ozonolysis followed by hydrolysis with lithium hydroperoxide was identified as the most efficient solution [51]. The corresponding primary amide was isolated as the dominant side product. Alternative methods reported for this conversion included harsh acidic or basic conditions [52, 53, 54, 55] not compatible with substrate **28**. The resulting carboxylic acid was engaged in an amide coupling reaction with side chain fragment **32**. Fmoc deprotection then delivered eastern fragment **33**.

**SCHEME 8:**
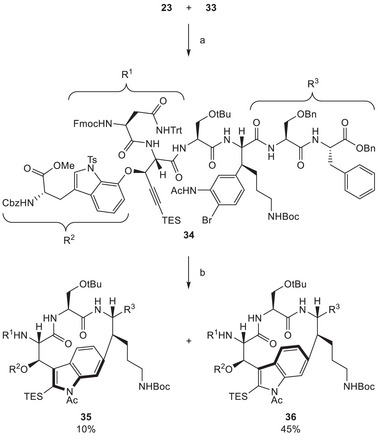
Reagents and conditions: (a) HATU, DIPEA, DMF, rt, 16 h, 70%; (b) Pd(tBu_3_P)_2_, NEt_3_, MeCN, 80°C, 1 h.

With both the western fragment **23** and the eastern fragment **33** in hand, we approached the final steps of our planned synthesis. Joining them through an amide coupling reaction delivered advanced intermediate **34**, comprising the complete carbon backbone of darobactin A (**1**). Therefore, we could focus on the two macrocyclizations and global deprotection (Scheme 8), which were left to achieve. Closure of the eastern ring was accomplished utilizing Larock indole synthesis conditions. After purification we isolated two atropisomeric products in a ratio of approximately 1:4.

Through extensive analysis by 2D‐NMR, we identified atropisomers **35** and **36**, arising from considerable ring strain in this 14‐membered macrocycle (see Si‐Figures 1–6, also see SI‐discussion 1). This phenomenon was later also discussed by Sarlah and Baran [14, 15]. 2D‐NMR analysis additionally confirmed the proposed configuration of compound **28** (scheme 6). To our dismay the desired atropisomer **35** was identified as the minor product of this cyclization. The ratio of the two products could not be altered by employing different catalysts, solvents, or temperatures, and they were found to be not interconvertible.

**SCHEME 9:**
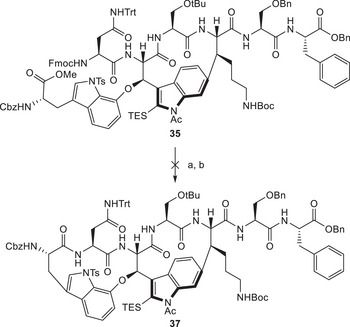
Reagents and conditions: (a) Me_3_SnOH, DCE, 50°C, 2 d, *then* piperidine, rt, 30 min; (b) various macrolactamization conditions.

Nevertheless, we had been able to isolate small amounts of the desired product intermediate **35**, which we again submitted to various macrolactamization conditions after deprotections utilizing the previously established conditions (Scheme 9). Unfortunately, even with this more pre‐organized substrate, these attempts remained futile, and no cyclization could be observed. Recently, Zhao's group reported that ynamines could be used under mild conditions to form peptide bonds [56]. This strategy was applied to a large scope and also for macrolactamization. We were not able to attempt these conditions, but they might be considered for such synthetic challenges. Based on these results, we decided to reorient our synthesis, especially regarding the assembly of the western part of darobactin A (**1**).

**SCHEME 10:**
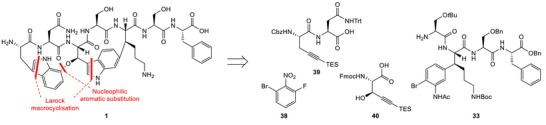
Second‐generation retrosynthetic analysis of darobactin A (**1**).

In light of the two successful total syntheses achieved by Sarlah and Baran in parallel to this study [14, 15], we decided to adjust our synthetic plan accordingly, aiming for a formal synthesis of darobactin A (**1**) and demonstrating the quality of fragment **33** as a plausible precursor. As outlined in Scheme 10, we included a second Larock indole synthesis approach to achieve cyclization of the western ring.

In addition to the already prepared eastern fragment **33**, we proposed fragments **38**, **39**, and **40** as the revised building blocks. Our previous investigations had confirmed that nucleophilic aromatic substitution was a valuable strategy for the construction of the crucial alkyl‐aryl ether bridge; thus, we intended to keep it as a key step. Utilizing the brominated electrophile **38** would deliver the necessary synthetic handle to achieve the proposed second Larock macrocyclization. The alkyne moiety was envisioned to be introduced through fragment **39**, which Sarlah and coworkers had already used in their approach [14].

**SCHEME 11:**
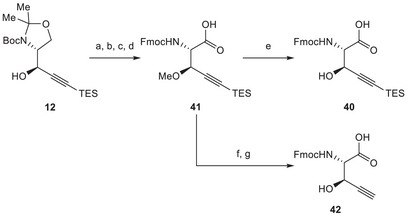
Reagents and conditions: (a) MeI, NaHMDS, THF, −40°C to rt, 1.5 h, 78%; (b) TFA, CH_2_Cl_2_, rt, 30 min; (c) Fmoc‐*O*Su, NaHCO_3_, dioxane/H_2_O, rt, 64 h, 97% over two steps; (d) TEMPO, PIDA, NaClO_2_, MeCN, aq. phosphate buffer (pH = 6.4), rt, 3 h, 85%; (e) BBr_3_, CH_2_Cl_2_, −40°C, 3 h, 53%; (f) TBAF, THF, rt, 2 h, 84%; (g) BBr_3_, CH_2_Cl_2_, −40°C, 1.5 h, 36%.

On the basis of this revised plan, we set out to prepare fragment **40**, starting from propargylic alcohol **12** (Scheme 11). Different protecting groups for the hydroxyl function were evaluated. Ultimately, intermediate protection of the hydroxy group as a methyl ether was found suitable, and we achieved the desired transformations. Oxazolidine cleavage followed by amine reprotection and oxidation delivered intermediate **41**, which was transformed into the desired building block **40** through demethylation. In addition, we prepared its desilylated analogue **42** to investigate the influence of such silyl groups on the atroposelectivity during the Larock macrocyclization.

**SCHEME 12:**
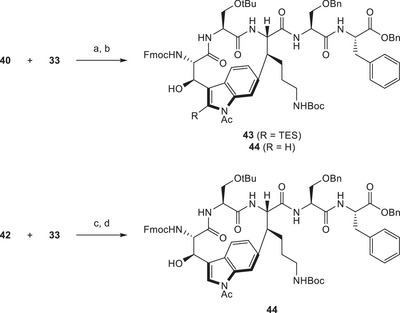
Reagents and conditions: (a) HATU, DIPEA, DMF, rt, 1 d, 67%; (b) Pd(tBu_3_P)_2_, NEt_3_, dioxane, 105°C, 30 min, 35% (combined); (c) HATU, DIPEA, DMF, rt, 16 h, 93%; (d) Pd(tBu_3_P)_2_, NEt_3_, dioxane, 105°C, 30 min, 40%.

Next, we coupled the new building blocks **40** and **42** to our established eastern fragment **33** (Scheme 12). Pleasingly, we could observe excellent atroposelectivity toward the desired product isomers, as only traces of the undesired atropisomer were detected. Reducing the steric bulk at the propargylic position appeared to have a great influence on the described selectivity. The change in solvent to dioxane instead of acetonitrile allowed us to conduct the reaction at higher temperatures, shortening the reaction times, which was desirable as we noted considerable decomposition of products at extended reaction times. When employing the silylated alkyne substrate **40**, we observed partial TES deprotection under the given conditions. Unfortunately, the resulting products, **43** and **44**, were inseparable, and we were unable to achieve clean TES deprotection upon treatment of the product mixture with desilylating agents. On the contrary, directly using desilylated fragment **42** as a substrate allowed us to isolate the desired product **44** in clean form.

**SCHEME 13:**
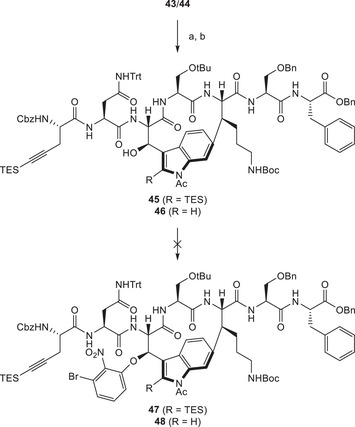
Reagents and conditions: (a) piperidine, DMF, rt, 1 h; (b) **39**, HATU, DIPEA, DMF, rt, 19 h, 93% (combined).

We then focused on the introduction of the aryl building block **38** through nucleophilic aromatic substitution. Because of Fmoc base lability in **43** and **44**, the Fmoc protecting group was removed, first and alkyne‐containing fragment **39** (prepared in four steps [14]) was attached through an amide coupling, delivering a mixture of **45** and **46**. Due to a lack of pure material, we engaged a mixture of **43** and **44** to conduct these investigations. To our dismay, our attempts at performing a nucleophilic substitution with substrates **45** and **46** remained unsuccessful (Scheme 13). We evaluated the use of different bases in this case and mainly observed decomposition of the starting materials. Since investigations into late‐stage Mitsunobu reactions to construct the ether bridge remained equally fruitless, our revised approach had also reached a dead end. As the different strategies evolved closer to already published syntheses [14, 15], we concluded our efforts at this stage.

## 3 Conclusion

In summary, our investigations on darobactin A led to valuable insights for the synthesis of interesting structural motifs. Notably, we were able to prepare darobactin A's western tryptophan moiety, including the adjacent alkyl‐aryl ether bridge, over an unprecedented series of steps, including nucleophilic aromatic substitution, Bartoli indole synthesis, and indole alkylation by Negishi coupling (Scheme 4). This key intermediate ultimately could not be used for the full construction of the western macrocycle, as the presented macrolactamization approach failed in constructing darobactin A's very strained western ring.

The described, highly scalable strategy for the preparation of ether‐bridged tryptophan derivatives might serve as an inspiration for the synthesis of other natural products containing this fascinating motif. In addition, we were also able to access a proposed β‐arylated lysine fragment through C‐H arylation, albeit in disappointingly low yield and selectivity, especially compared to the approaches by Sarlah and Baran, published in the meantime [14, 15]. Nevertheless, this enabled us to study the atroposelectivity of the Larock indole cyclization in darobactin A's eastern macrocycle with different substrates, identifying the substitution at the propargylic position of the substrate as a major factor. We hope that the lessons we have learned during this endeavor will be considered when developing novel strategies for similarly challenging natural products.

## Conflicts of Interest

The authors declare no conflict of interest

## Supporting information



The experimental procedures, NMR spectra, additional figures and tables are available free of charge as the Supporting Information via the Internet. Additional references for the supporting information only [57, 58].
