# Supplementary material for: Synthetic Studies Towards Darobactin A
Source: Chemistry. 2026 Jan 10;32(10):e03359. doi: 10.1002/chem.202503359 (PMC12995848; doi:10.1002/chem.202503359)
Supplement: Supplementary file 1 — The experimental procedures, NMR spectra, additional figures and tables are available free of charge as the Supporting Information via the Internet. Additional references for the supporting information only [57, 58]. [file CHEM-32-e03359-s001.docx]

**Supporting Information**

**Table of content**

Supplementary figures 2

General information 12

Synthetic procedures 14

Reference of the supporting information 53

NMR spectra of synthesized compounds 54

**Supplementary figures**

**SI-Table 1**. Conditions screened for ether formation through a Mitsunobu reaction.

| Entry | Phosphine | Azodicarboxylate | Solvent | Temperature | Yield |
| --- | --- | --- | --- | --- | --- |
| 1 | PPh_3_ | DEAD | THF | rt | n.r.^a^ |
| 2 | PPh_3_ | DIAD | THF | rt | n.r. |
| 3 | PPh_3_ | DEAD | THF | reflux | decomp.^b^ |
| 4 | PPh_3_ | DIAD | THF | reflux | decomp. |
| 5 | PPh_3_ | DIAD | toluene | reflux | decomp. |
| 6 | PPh_3_ | DEAD | CH_2_Cl_2_ | rt | n.r. |
| 7 | PPh_3_ | DEAD | CH_2_Cl_2_ | reflux | decomp. |
| 8 | CMMP | | CH_2_Cl_2_ | reflux | decomp. |

Conversion was followed by UHPLC-MS and TLC; a) no reaction, b) decomposition.

**SI-Scheme 1**. Failed synthetic approaches for the construction of alkyl-aryl ether **15** with stereoinvertive methods (a, b) and stereoretentive methods (c).

**SI-Table 2**. Screening of the Bartoli indole synthesis of indole **17** from nitroarene **18**.

| Entry | X | Equiv. of vinyl magnesium halide | Solvent | Temperature | Reaction time | Yield |
| --- | --- | --- | --- | --- | --- | --- |
| 1 | Br | 5.0 | THF/Et_2_O (3:4) | -40 °C | 2 h | <5% |
| 2 | Br | 5.0 | THF | -78 °C | 5 h | 15% |
| 3 | Br | 10.0 | THF | -78 °C | 6 h | 12% |
| 4 | Cl | 5.0 | THF | -78 °C | 3 h | 16% |
| 5 | Br | 5.0 | THF/DME (1:1) | -40 °C | 3 h | 12% |
| 6 | Br | 5.0 | THF/MTBE (1:1) | -40 °C | 3 h | 13% |
| 7 | Br | 5.0 | THF/dioxane (3:1) | -40 °C | 3 h | 27% |
| 8 | Cl | 5.0 | THF/dioxane (3:1) | -40 °C | 3 h | 10% |
| 9^a^ | Br | 5.0 | THF/dioxane (3:1) | -40 °C | 3 h | 41% |

a) addition of vinyl magnesium halide by syringe pump over 1 h.

**SI-Table 3**. Screening of macrolactamization of the western ring.

| Entry | Starting material | Coupling agent (equiv.) | Base (equiv.) | Solvent | Observations |
| --- | --- | --- | --- | --- | --- |
| 1^a^ | **SI-6** | HATU | DIPEA | DMF | unclear |
| 2^b^ | **SI-6** | DEPBT | NaHCO_3_ | DMF | suspected cyclized dimer |
| 3^a^ | **SI-6** | DEPBT | NEt_3_ | DMF | unclear |
| 4^a^ | **SI-6** | DEPBT | DIPEA | DMF | unclear |
| 5^a^ | **SI-5** | DEPBT | DIPEA | CH_2_Cl_2_ | traces of desired mass detected |

Conversion was followed by UHPLC-MS; starting materials were applied as crudes; a) reaction run at rt, b) reaction run at 50 °C

**SI-Scheme 2.** Investigated sequential C-H arylation/C-H alkylation for the construction of the β-arylated lysine fragment **SI-8**.

**SI-Scheme 3**. Abandoned approaches for the construction of darobactin A's β-arylated lysine motif.

**SI-Scheme 4** (continued). Abandoned approaches for the construction of darobactin A's β-arylated lysine motif.

*
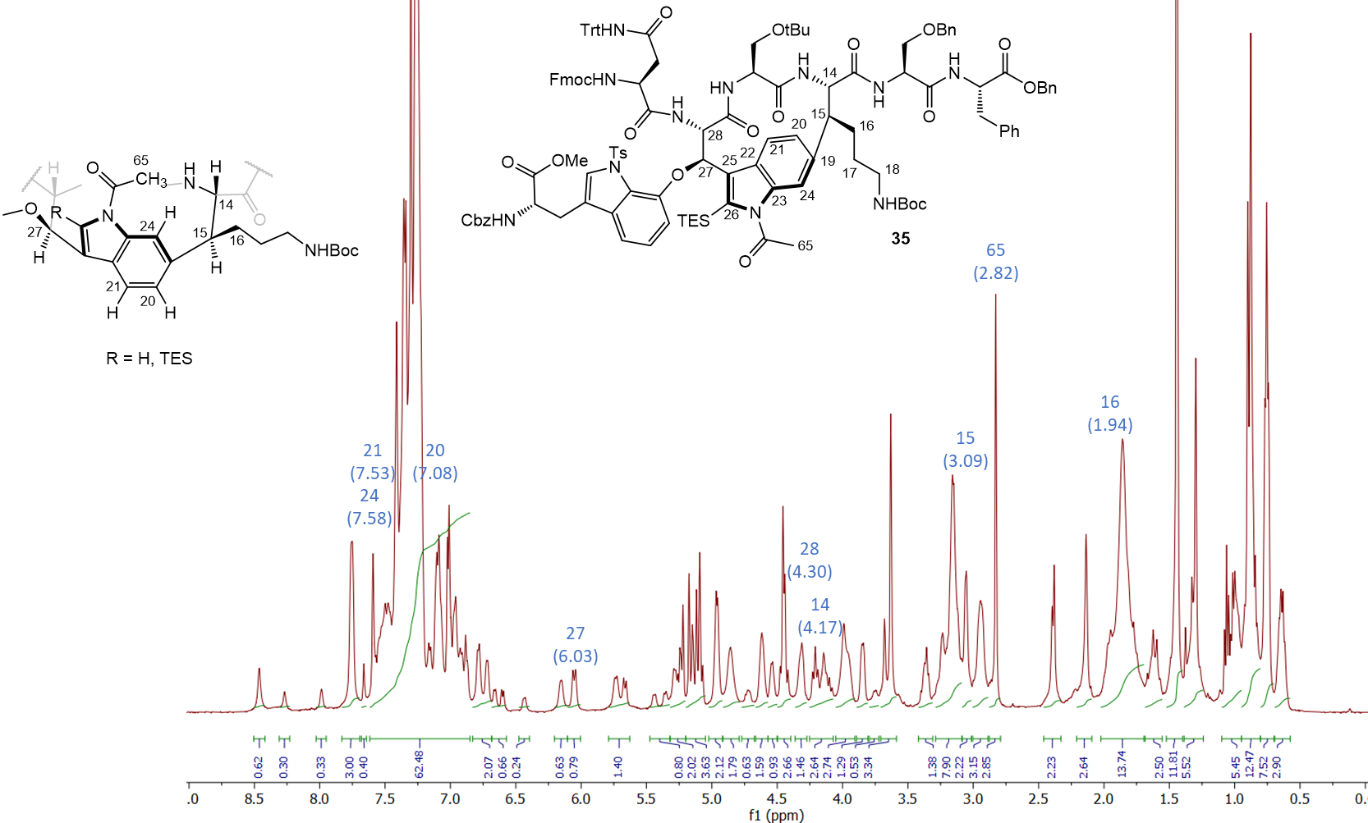
*

**SI-Figure 1**: ^1^H-NMR in CDCl_3_ of compound **35**, key protons are highlighted in blue with their chemical shifts (mixture between R = H or TES)


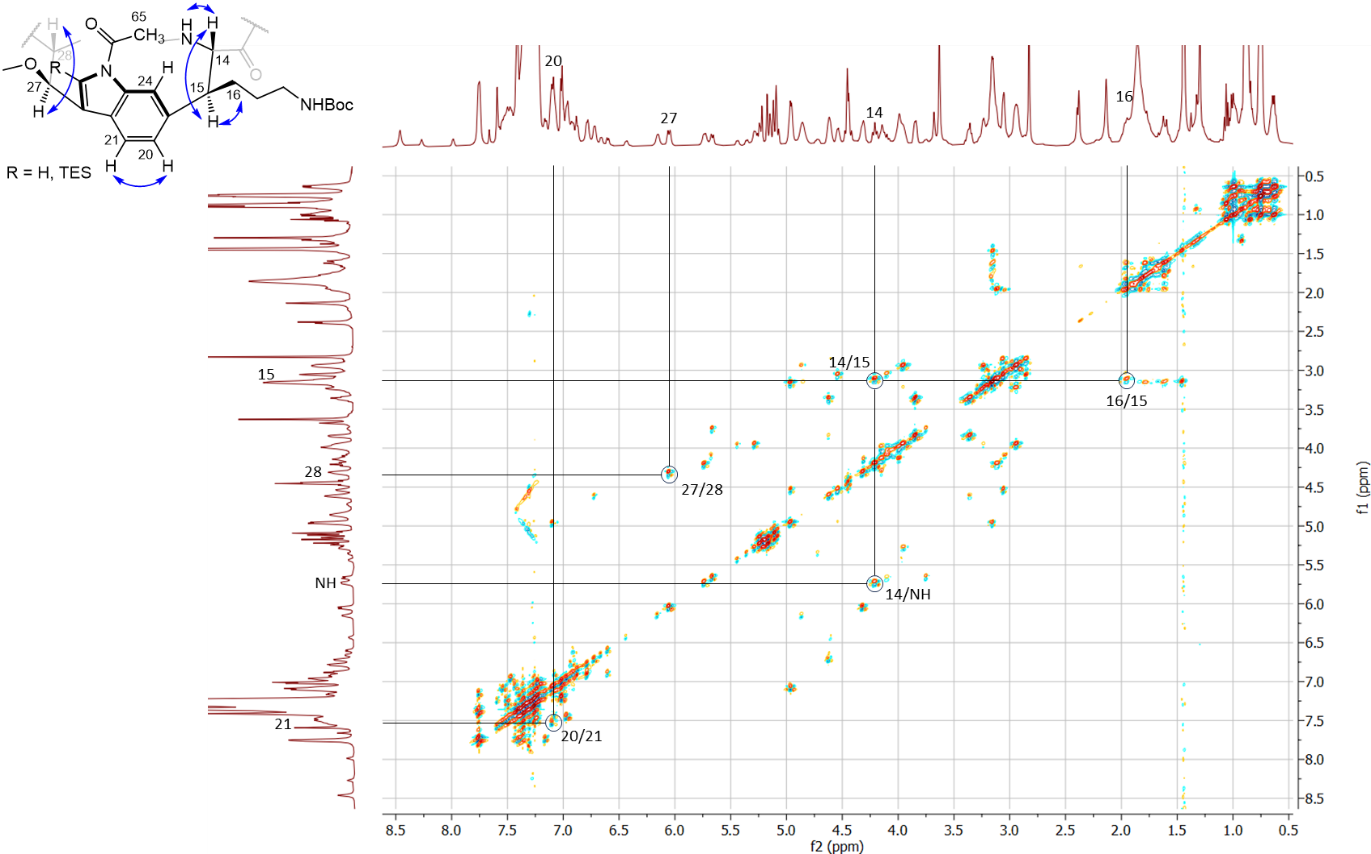


**SI-Figure 2**: COSY-NMR in CDCl_3_ of compound **35**. Blue arrows account for identified correlations.

**
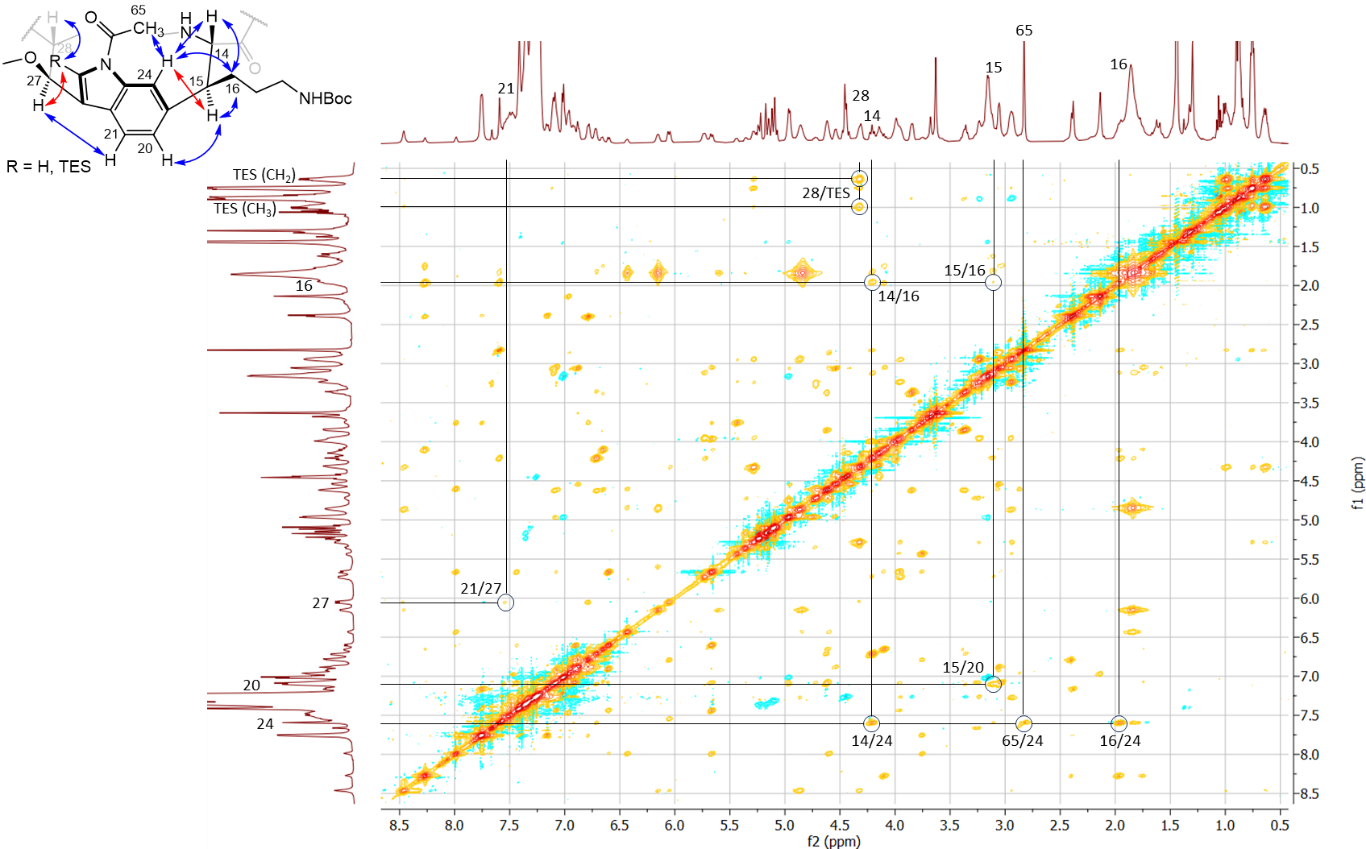
**

**SI-Figure 3**: NOESY-NMR in CDCl_3_ of compound **35.** Blue arrows account for identified correlations. Red arrows indicate correlations which were not observed.

**
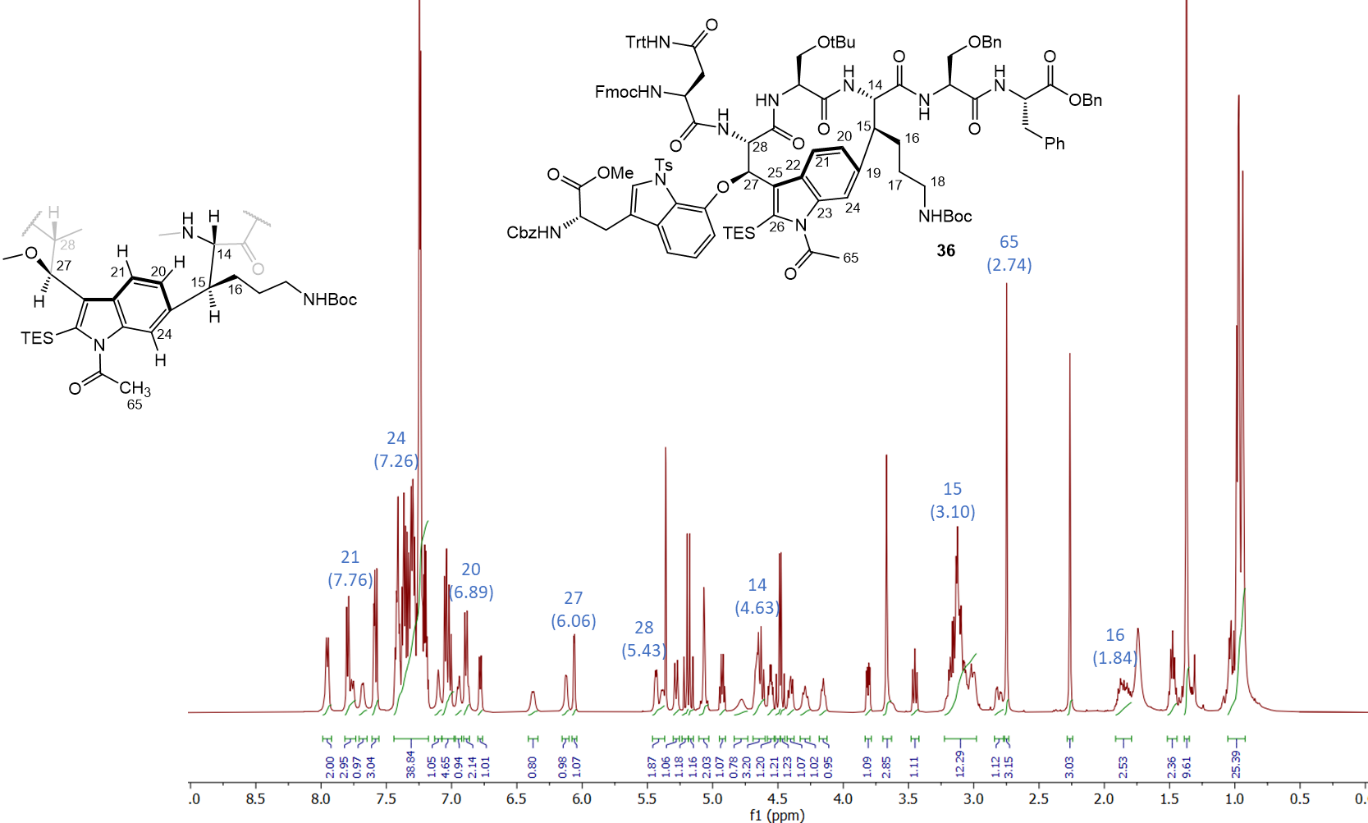
**

**SI-Figure 4**: ^1^H-NMR in CDCl_3_ of compound **36**, key protons are highlighted in blue with their chemical shifts.


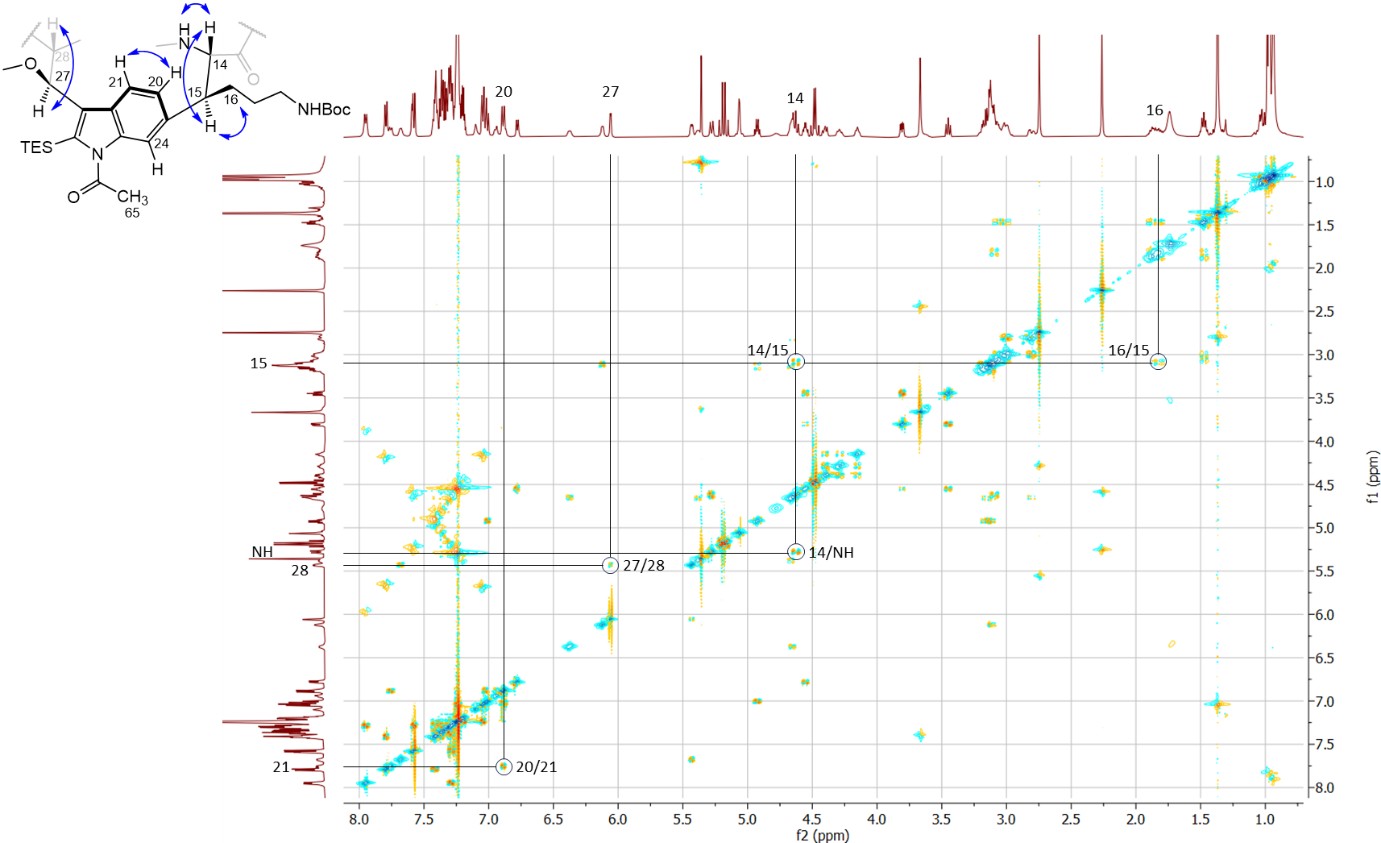


**SI-Figure 5**: COSY-NMR in CDCl_3_ of compound **36**. Blue arrows account for identified correlations.


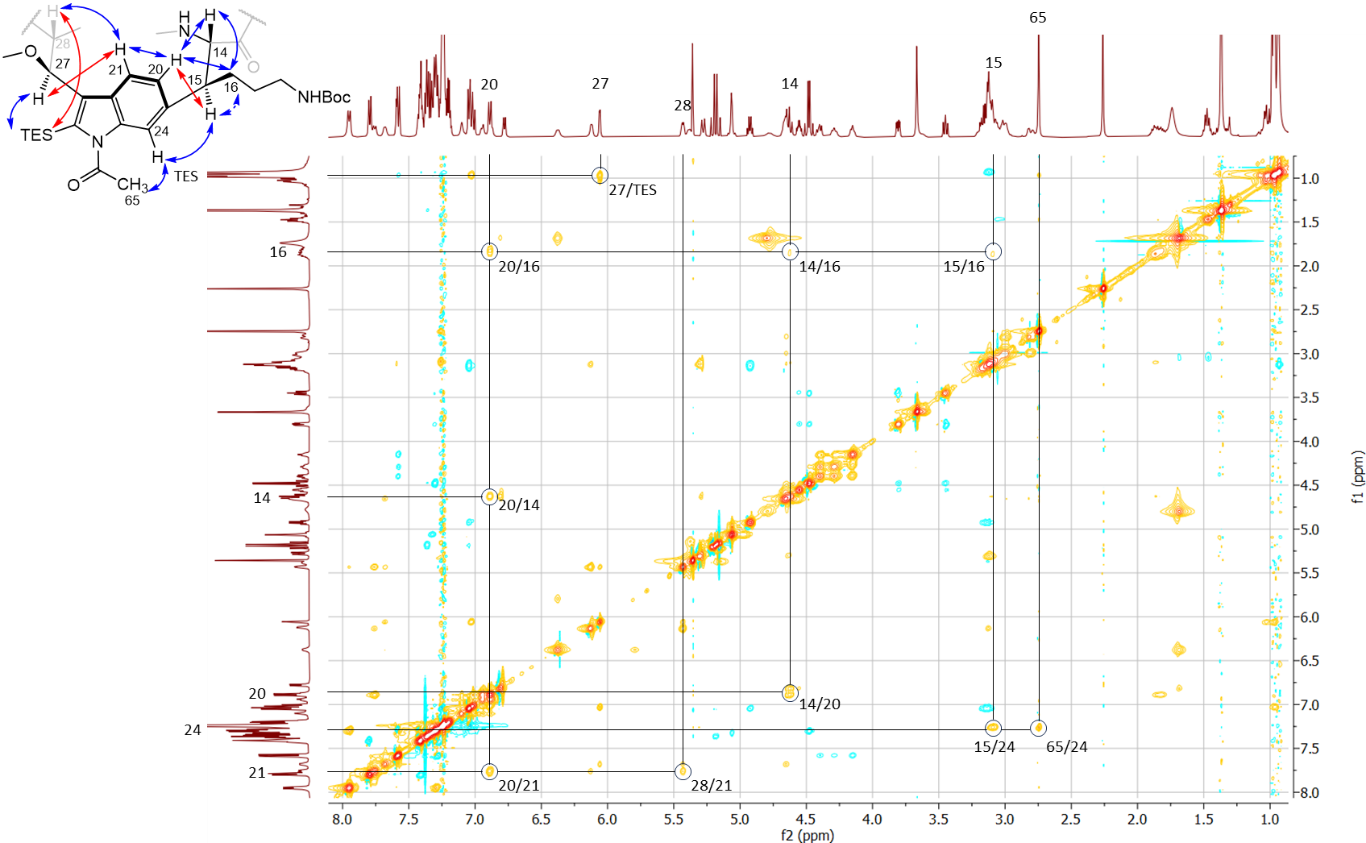


**SI-Figure 6**: NOESY-NMR in CDCl_3_ of compound **36.** Blue arrows account for identified correlations. Red arrows indicate correlations which were not observed.

**SI-discussion 1**

To identify the atropisomers we used COSY- and NOESY-NMR.

The COSY NMR validated the correlations that exist: Between protons H^15^-H^14^ and H^15^-H^16^ (SI-Figure 2 & 5) on the β-arylated lysine; between protons H^20^-H^21^ on the indole ring; and between protons
H^27^-H^28^ from the tryptophane alkyl chain. The three-dimensional orientations were then attributed using NOESY-NMR analysis (SI-Figure 3 & 6). The correlation H^65^-H^24^ can be identified in both **35** and **36** as well as the correlations H^15^-H^16^ and H^16^-H^14^. The proton H^24^ has two key spatial correlations with H^14^ and H^16^ in **35** while it only correlates with H^15^ in 36. Crucially, this correlation with H^15^ is missing in 35. The geometry can be further confirmed with the identified correlation between H^15^ and H^20^ in 35 while H^20^ correlates with H^16^ and H^14^ in **36**. A third set of correlations can be noticed between H^27^-H^21^ and H^28^-TES in 35 where the opposite correlations H^28^-H^21^ and H^27^-TES are visible in 36. Additionally, no correlation is visible in NOESY-NMR between H^14^ and H^15^ in any of the two atropisomers, thus confirming the (*S*) configuration at C^15^.H^15^ in both compounds, confirming the assigned stereochemistry of the β-arylated lysine. In conclusion, these data account for the presented structure for **35** and **36**.

**General information:** All chemicals were purchased from Sigma-Aldrich, Acros, Alfa-Aesar, TCI or Fluka and were used without further purification. All reactions were carried out in oven dried glassware under a nitrogen atmosphere. Solvents applied for chemical transformations were either puriss. quality or HPLC grade solvents. For work-up and purification solvents were distilled from technical grade. All synthetic transformations were monitored by either thin layer chromatography (TLC), ^1^H-NMR spectroscopy, UHPLC-MS. TLC was performed on Merck silica gel 60 F_254_ plates (0.25 mm thickness) precoated with a fluorescent indicator. The developed plates were examined under UV light and stained with cerium ammonium molybdate, *p*-anisaldehyde, or potassium permanganate followed by heating. Concentration under reduced pressure was performed by rotary evaporation *in vacuo* at 40 °C. Column chromatography was performed using silica gel 60 (230–400 mesh) from Sigma-Aldrich a forced flow eluent at 0.2–1.0 bar pressure. Flash column chromatography was performed on Biotage Isolera One, fitted prepacked columns purchased from Macherey-Nagel. All ^1^H-NMR, ^13^C-NMR, ^31^P-NMR, and 2D-NMR spectra were recorded on Bruker 400 MHz (^1^H) & 101 MHz (^13^C) or Bruker 500 MHz (^1^H) & 126 MHz (^13^C) spectrometers at 25 °C unless stated otherwise. Chemical shifts (δ-values) are reported in ppm, spectra were calibrated relative to residual solvent proton chemical shifts (acetone-*d_6_*, δ = 2.05; benzene-*d_6_*, δ = 7.15; CDCl_3_, δ = 7.26; D_2_O, δ = 4.79; DMSO-*d_6_*, δ = 2.50; methanol-*d_4_*, δ = 3.31) and carbon chemical shifts (acetone-*d_6_*, δ = 29.84; benzene-*d_6_*, δ = 128.00; CDCl_3_, δ = 77.16; DMSO-*d_6_*, δ = 39.52; methanol-*d_4_*, δ = 49.00), multiplicity is reported as follows: br. = broad, s = singlet, d = doublet, dd = doublet of doublets, ddd = doublet of doublet of doublets, t = triplet, td = triplet of doublets, dt = doublet of triplets, tt = triplet of triplets, q = quartet, dq = doublet of quartets, m = multiplet or unresolved and coupling constants *J* in Hz. IR spectra were recorded on a Varian 800 FT-IR ATR spectrophotometer with intensities being reported as strong (s), medium (m), and weak (w) and given in cm^–1^. Ultra-high performance liquid chromatography coupled to mass spectrometry (UHPLC-MS): *Ultimate 3000 LC* instrument (*Thermo Fisher Scientific*) coupled to a triple quadrupole *Quantum Ultra EMR* MS (*Thermo Fisher Scientific*) using a reversed-phase column (*Kinetex®* EVO C18; 1.7 mm; 100 Å, 50 x 2.1 nm; *Phenomenex*). The LC was equipped with and *HPG-3400RS* pump, a *WPS-3000TRS* autosampler, a *TCC-3000RS* column oven and a Vanquish *DAD* detector (all *Thermo Fisher Scientific*). The following solvents were applied: H_2_O + 0.1% HCOOH (A), MeCN + 0.1% HCOOH (B). Samples were prepared using HPLC grade solvents (MeCN, MeOH, H_2_O) and filtered over a 4 mm syringe filter, PTFE (hydrophilic), pore size: 0.22 mm obtained from *BGB Analytik AG*. The MS was equipped with an H-ESI II ion source. The source temperature was 250 °C, the capillary temperature 270 °C and capillary voltage 3500 V, and datasets were acquired at resolution 0.7 on Q3 in centroid mode. High performance liquid chromatography (HPLC): *Prominence* modular HPLC instrument (*Shimadzu*) coupled to an *SPD-20A* UV/Vis detector (*Shimadzu*) using a reversed-phase column (*Gemini NX* C18, 3 μm, 10 Å, 150 x 4.6 mm) for analytical HPLC, and a reversed-phase column (*Gemini NX* C18, 5 μm, 110 Å, 250 x 21.2 mm) for preparative HPLC. The LC was equipped with a *CBM-20A* system controller, *LC-20A* solvent delivery unit, a *DGU-20A* degassing unit, *FRC-10A* fraction collector (all *Shimadzu*). The following solvents were used: H_2_O + 0.1% HCOOH (A), MeCN + 0.1% HCOOH (B). All high-resolution mass spectra (HR-ESI & HR-EI) were recorded by the mass spectrometry service at the University of Zurich on a *Dionex Ultimate 3000* UHPLC system (*Thermo Fisher Scientific*, Germering, Germany) connected to a *QExactive* MS with a heated ESI source (*Thermo Fisher Scientific*, Bremen, Germany); on-flow injection of 1 mL sample (*c* = approx. 50 mg mL^–1^ in the indicated solvent) with an *XRS* autosampler (*CTC*, Zwingen, Switzerland); flow rate 120 mL min^–1^; ESI: spray voltage 3.0 kV, capillary temperature 280 °C, sheath gas 30 L min^–1^, aux gas 8 L min^–1^, s-lens RF level 55.0, aux gas temperature 250 °C (N_2_); full scan MS in the alternating (+)/(–)-ESI mode; mass ranges 80–1’200 *m/z*, 133–2’000 *m/z*, or 200–3’000 *m/z* at 70’000 resolution (full width half-maximum); automatic gain control (AGC) target of 3.00∙10^6^; maximum allowed ion transfer time (IT) 30 ms; mass calibration to <2 ppm accuracy with *Pierce®* ESI calibration solutions (*Thermo Fisher Scientific*, Rockford, USA); lock masses: ubiquitous erucamide (*m/z* 338.34174, (+)-ESI) and palmitic acid (*m/z* 255.23295, (–)-ESI) or (for EI) on a *DFS* double-focusing (BE geometry) magnetic sector mass spectrometer (*Thermo Fisher Scientific*, Bremen, Germany). Mass spectra were measured with electron ionization (EI) at 70 eV, solid probe inlet, a source temperature of 200 °C, an acceleration voltage of 5 kV, and a resolution of 10’000. The instrument was scanned between *e.g.* *m/z* 300 and 350 at a scan rate of 100–200 s/decade in the electric scan mode. Perfluorokerosene (*PFK*, *Fluorochem*, Derbyshire, UK) served for calibration. Melting points (M.p.) were determined using a *Büchi B-545* apparatus in open capillaries and are uncorrected. Specific optical rotation was measured on a *JASCO P-2000 Polarimeter*, measured at the indicated temperature in the given solvent.

**Synthetic procedures**

**Synthesis of 11**

To a solution of TES-acetylene (1.00 mL, 5.58 mmol, 1.60 equiv.) in dry THF (20 mL) was slowly added *n*BuLi (1.6 M in hexanes, 3.30 mL, 5.24 mmol, 1.50 equiv.) at –78 °C. After stirring at this temperature for 1 h HMPA (1.20 mL, 6.98 mmol, 2.00 equiv.) and a solution of d-Garner’s aldehyde (**10**, 800 mg, 3.49 mmol, 1.00 equiv.) in dry THF (20 mL) were added dropwise. The resulting mixture was stirred at this temperature for 2 h and then quenched by the addition of sat. aq. NH_4_Cl (50 mL) at 0 °C. The aq. layer was extracted with EtOAc (3 × 100 mL). The combined organic layers were washed with water (100 mL) and brine (100 mL), dried over MgSO_4_, filtered and the solvent was removed under reduced pressure. Purification by silica gel flash column chromatography (EtOAc/hexanes 2% to 22%) delivered alcohol **11** (1.11 g, 3.01 mmol, 86%, d.r. >25:1) as a colorless oil.

R*_f_* = 0.50 (EtOAc/hexanes 1:4).

^1^H-NMR (400 MHz, DMSO-*d_6_*, 80 °C): δ 5.40 (d, *J* = 6.2 Hz, 1H), 4.60 (dd, *J* = 6.4, 3.2 Hz, 1H), 4.05 (d, *J* = 7.1 Hz, 1H), 3.97 (dd, *J* = 8.7, 6.8 Hz, 1H), 3.90 (br. s, 1H), 1.48 (s, 3H), 1.44 (s, 12H), 0.97 (t, *J* = 7.9 Hz, 9H), 0.58 (q, *J* = 7.9 Hz, 6H) ppm.

^13^C-NMR (126 MHz, DMSO-*d_6_*, 80 °C): δ 151.3, 107.6, 93.6, 85.6, 79.0, 63.2, 61.2, 60.4, 27.7, 25.5, 6.7, 3.6 ppm.

(+)-HR-ESI-MS: *m/z* 392.2228 calculated for C_19_H_35_NO_4_SiNa [M+Na]^+^; found 392.2226.

IR (neat): 3385 (w), 2955 (m), 2876 (m), 2169 (w), 1692 (m), 1670 (m), 1458 (w), 1392 (s), 1367 (s), 1245 (m), 1207 (w), 1171 (m), 1116 (m), 1091 (m), 1071 (m), 1019 (m), 973 (w), 947 (w), 846 (w), 809 (w), 770 (w), 728 (m).

${[\alpha]}_{D}^{23}$: + 50.1 (*c* = 1.08, CHCl_3_).

**Synthesis of 12**

To a solution of TES-acetylene (4.96 mL, 27.7 mmol, 1.27 equiv.) in dry THF (75 mL) was added over dropwise by syringe pump (over 1 h) a solution of EtMgBr (3 M in Et_2_O, 8.72 mL, 26.2 mmol, 1.20 equiv.) at 0 °C. The mixture was heated to reflux for 2 h and then cooled to rt. The resulting *Grignard* solution was transferred to a solution of CuI (9.55 g, 50.1 mmol, 2.30 equiv.) in dry THF/Me_2_S (15:1, 80 mL) at −78 °C. The resulting mixture was warmed to −30 °C and stirred at this temperature for 1 h. After cooling back to −78 °C a solution of d-Garner’s aldehyde (**10**, 5.00 g, 21.8 mmol, 1.00 equiv.) in dry THF (20 mL) was added dropwise by syringe pump over 20 min. The mixture was stirred at this temperature for 1 h and then slowly warmed up to rt and stirred for 16 h. The reaction was quenched by the addition of sat. aq. NH_4_Cl (200 mL). The mixture was extracted with Et_2_O (3 × 500 mL). The combined organic layers were washed with brine (500 mL), dried over MgSO_4_, filtered and the solvent removed under reduced pressure. The crude mixture was purified by silica gel flash column chromatography (EtOAc/hexanes 2% to 22%) to afford alcohol **12** (6.37 g, 17.2 mmol, 79%, d.r. >25:1) as a colorless oil.

R*_f_* = 0.20 (EtOAc/hexanes 1:10).

^1^H-NMR (400 MHz, DMSO-*d_6_*, 80 °C): δ 5.49 (d, *J* = 5.5 Hz, 1H), 4.78 (t, *J* = 5.3 Hz, 1H), 4.07 (dd, *J* = 9.1, 3.9 Hz, 1H), 3.98 (dd, *J* = 9.4, 6.7 Hz, 1H), 3.91–3.80 (m, 1H), 1.54 (s, 3H), 1.44 (s, 9H), 1.42 (s, 1H), 0.97 (t, *J* = 7.9 Hz, 9H), 0.57 (q, *J* = 7.8 Hz, 6H) ppm.

^13^C-NMR (126 MHz, DMSO-*d_6_*, 80 °C): δ 151.3, 106.8, 93.5, 86.0, 79.1, 63.7, 61.2, 60.2, 27.7, 25.8, 6.7, 3.6 ppm.

(–)-HR-ESI-MS: *m/z* 404.2029 calculated for C_19_H_35_NO_4_ClSi [M+Cl]^–^; found 404.2033.

IR (neat): 3442 (w), 2955 (w), 2876 (w), 1693 (m), 1458 (w), 1391 (s), 1366 (s), 1248 (m), 1207 (w), 1168 (m), 1085 (m), 1061 (s), 1018 (w), 966 (w), 949 (w), 854 (w), 807 (w), 770 (w), 726 (m), 580 (w).

${[\alpha]}_{D}^{23}$: + 44.2 (*c* = 0.85, CHCl_3_).

**Synthesis of 13**

*Literature procedure.*^[1]^ Boc-Trp-OMe (**SI-23**, 324 mg, 1.02 mmol, 1.00 equiv.), [Ir(cod)OMe]_2_ (16.7 mg, 25.3 mmol, 2.5 mol%) and dtbpy (13.6 mg, 50.5 mmol, 5.0 mol%) were dissolved in dry THF (12 mL). To the resulting red solution was added HBpin (750 mL, 5.05 mmol, 5.00 equiv.) in a single portion at rt. The resulting mixture was stirred at 60 °C for 6 h. After this time the mixture was cooled down to rt and concentrated under reduced pressure. The resulting brown residue was redissolved in AcOH (5.0 mL) and Pd(OAc)_2_ (11.3 mg, 50.5 mmol, 5.0 mol%) was added at rt. The resulting mixture was stirred at this temperature for 16 h and then filtered through a pad of celite, eluting with EtOAc. The filtrate was carefully washed with sat. aq. NaHCO_3_ (3 × 25 mL), dried over MgSO_4_, filtered and the solvent removed under reduced pressure. The crude mixture was purified by silica gel column chromatography (acetone/CH_2_Cl_2_/hexanes 5%/15%/80%) to afford **SI-24** (303 mg, 682 mmol, 68%) as a white solid.

R*_f_* = 0.20 (acetone/CH_2_Cl_2_/hexanes 5:15:80).

^1^H-NMR (400 MHz, CDCl_3_): δ 9.12 (br. s, 1H), 7.67 (d, *J* = 7.9 Hz, 1H), 7.64 (dd, *J* = 7.0, 1.1 Hz, 1H), 7.13 (dd, *J* = 8.0, 7.1 Hz, 1H), 7.06 (s, 1H), 5.05 (d, *J* = 8.0 Hz, 1H), 4.63 (dd, *J* = 13.5, 6.3 Hz, 1H), 3.67 (s, 3H), 3.30 (d, *J* = 5.4 Hz, 2H), 1.42 (s, 9H), 1.39 (s, 12H) ppm.

^13^C-NMR (126 MHz, CDCl_3_): δ 172.9, 155.4, 141.4, 136.2, 129.6, 126.8, 122.9, 122.5, 119.3, 109.7, 84.0, 79.9, 54.4, 52.4, 28.5, 28.1, 25.2 ppm.

(+)-HR-ESI-MS: *m/z* 445.2504 calculated for C_23_H_34_N_2_O_6_B [M+H]^+^; found 445.2506.

IR (neat): 3387 (w), 2979 (w), 1740 (m), 1703 (m), 1593 (w), 1504 (w), 1439 (w), 1371 (m), 1333 (m), 1295 (m), 1268 (w), 1216 (m), 1144 (s), 1131 (s), 1082 (w), 1052 (w), 1015 (w), 968 (w), 850 (w), 805 (w), 780 (w), 759 (w), 683 (m), 566 (w), 495 (w), 472 (w).

${[\alpha]}_{D}^{23}$: + 38.7 (*c* = 1.00, CHCl_3_).

M.p.: 171-6–172.9 °C.

The obtained data are in agreement with the values reported in the literature.^[1]^

Borylated tryptophan **SI-24** (557 mg, 1.25 mmol, 1.00 equiv.) was dissolved in acetone (25 mL). An aq. solution of Oxone^®^ (384 mg, 1.25 mmol, 1.00 equiv. in 25 mL) was added dropwise over the course of 5 min at rt. The resulting mixture was stirred vigorously for 4 h. The reaction was quenched by the addition of sat. aq. Na_2_SO_3_ (10 mL) and extracted with CH_2_Cl_2_ (3 × 100 mL). The combined organic layers were washed with brine (200 mL), dried over MgSO_4_, filtered and the solvent removed under reduced pressure. The crude mixture was purified by silica gel column chromatography (dry loading, EtOAc/pentane 50%) to afford **13** (340 mg, 1.02 mmol, 81%) as a sticky yellowish foam.

R*_f_* = 0.42 (EtOAc/pentane 1:1).

^1^H-NMR (500 MHz, methanol-*d_4_*): δ 7.06–6.98 (m, 2H), 6.83 (t, *J* = 7.7 Hz, 1H), 6.52 (d, *J* = 7.5 Hz, 1H), 4.46–4.37 (m, 1H), 3.65 (s, 3H), 3.21 (dd, *J* = 14.5, 5.8 Hz, 1H), 3.09 (dd, *J* = 14.6, 7.5 Hz, 1H), 1.39 (s, 9H) ppm.

^13^C-NMR (126 MHz, methanol-*d_4_*): δ 174.7, 157.8, 144.6, 130.8, 128.0, 124.0, 120.5, 111.1, 110.9, 106.7, 80.6, 56.1, 52.6, 28.9, 28.7 ppm.

(–)-HR-ESI-MS: *m/z* 333.1456 calculated for C_17_H_21_N_2_O_5_ [M–H]^–^; found 333.1456.

IR (neat): 3363 (w), 1684 (m), 1583 (w), 1512 (w), 1499 (m), 1436 (w), 1392 (w), 1367 (m), 1306 (w), 1268 (m), 1232 (m), 1159 (s), 1093 (w), 1048 (m), 1024 (w), 978 (w), 781 (w), 734 (w), 609 (w), 543 (w).

${[\alpha]}_{D}^{23}$: + 6.0 (*c* = 1.00, CHCl_3_).

The obtained data are in agreement with the values reported in the literature.^[1]^

**Synthesis of SI-1**

Alcohol **11** (88.8 mg, 240 mmol, 1.00 equiv.) was dissolved in dry CH_2_Cl_2_ (5.0 mL) and treated with freshly recrystallized TsCl (137 mg, 720 mmol, 3.00 equiv.) and pyridine (29 mL, 360 mmol, 1.50 equiv.). The resulting mixture was stirred at rt for 7 d and then diluted with CH_2_Cl_2_ (20 mL) and water (20 mL). The aq. layer was extracted with CH_2_Cl_2_ (3 × 20 mL). The combined organic layers were washed with brine (50 mL), dried over MgSO_4_, filtered and the solvent was removed under reduced pressure. Purification by silica gel column chromatography (EtOAc/pentane 0% to 9%) delivered tosyl ether **SI-1** (88.0 mg, 168 mmol, 70%) as a colorless oil.

R*_f_* = 0.25 (EtOAc/pentane 1:10).

^1^H-NMR (400 MHz, DMSO-*d_6_*, 80 °C): δ 7.76 (d, *J* = 8.4 Hz, 2H), 7.46 (d, *J* = 8.4 Hz, 2H), 5.47 (s, 1H), 4.11–3.99 (m, 3H), 2.42 (s, 3H), 1.49 (s, 3H), 1.46 (s, 9H), 1.42 (s, 3H), 0.86 (t, *J* = 7.9 Hz, 9H), 0.47 (q, *J* = 7.9 Hz, 6H) ppm.

^13^C-NMR (126 MHz, DMSO-*d_6_*, 25 °C): δ 151.5 and 150.6 (1C), 145.2 and 145.1 (1C), 133.1 and 133.0 (1C), 130.11 and 130.08 (2C), 127.70 and 127.66 (2C), 99.1 (1C), 94.5 and 94.1 (1C), 92.4 and 92.1 (1C), 80.2 and 79.9 (1C), 70.3 and 69.3 (1C), 63.2 and 62.5 (1C), 60.3 and 59.8 (1C), 27.92 and 27.88 (3C), 26.1, 25.3, and 24.2 (2C), 22.8 and 21.1 (1C), 7.1 (3C), 3.3 (3C) ppm.^[[1]](#footnote-1)^

(+)-HR-ESI-MS: *m/z* 546.2316 calculated for C_26_H_41_NO_6_SSiNa [M+Na]^+^; found 546.2312.

IR (neat): 2957 (w), 2876 (w), 1708 (m), 1689 (m), 1457 (w), 1389 (m), 1366 (s), 1320 (w), 1264 (w), 1247 (w), 1209 (w), 1189 (m), 1177 (s), 1115 (w), 1099 (m), 1080 (m), 1065 (m), 1035 (m), 1019 (w), 954 (w), 929 (m), 884 (w), 852 (m), 813 (w), 765 (w), 729 (m), 679 (m), 666 (m), 564 (w), 551 (m).

${[\alpha]}_{D}^{23}$: + 66.3 (*c* = 1.00, CHCl_3_).

**Synthesis of SI-2**

To a solution of propargylic alcohol **11** (242 mg, 0.655 mmol, 1.00 equiv.) in dry CH_2_Cl_2_ (10 mL) were added MsCl (100 µL, 1.31 mmol, 2.00 equiv.) and NEt_3_ (280 µL, 1.97 mmol, 3.00 equiv.) at rt. After stirring for 1.5 h the mixture was diluted with CH_2_Cl_2_ (50 mL) and washed with aq. HCl (1 M, 2 × 20 mL), sat. aq. NaHCO_3_ (2 × 20 mL) and brine (20 mL). The organic layer was dried over MgSO_4_, filtered and the solvent was removed under reduced pressure to deliver the product **SI-2** (276 mg, 0.616 mmol, 93%) as a yellow oil.^[[2]](#footnote-2)^

R*_f_* = 0.35 (EtOAc/pentane 1:10).

^1^H-NMR (400 MHz, CDCl_3_): δ 5.74 and 5.57 (d, *J* = 2.7 Hz and s, 1H), 4.32–4.24 (m, 1H), 4.22–4.10 (m, 1H), 4.08 (d, *J* = 8.9 Hz, 1H), 3.14, 3.12, and 3.09 (3 × s, 3H), 1.65 and 1.61 (2 × s, 3H), 1.50 (s, 9H), 1.47 and 1.46 (2 × s, 3H), 1.03–0.92 (m, 9H), 0.68–0.55 (m, 6H) ppm.^[[3]](#footnote-3)^

(+)-HR-ESI-MS: *m/z* 470.2003 calculated for C_20_H_37_NO_6_SSiNa [M+Na]^+^; found 470.2004.

**Synthesis of SI-4**

Propargylic alcohol **11** (250 mg, 676 mmol, 1.00 equiv.) was dissolved in dry CH_2_Cl_2_ (5.0 mL) and dicobalt octacarbonyl (254 mg, 744 mmol, 1.10 equiv.) was quickly added. The resulting mixture was stirred at rt for 30 min after which the solvent was removed under reduced pressure. Purification of the crude brown residue by silica gel column chromatography (EtOAc/pentane 0% to 9%) delivered complex **SI-4** (433 mg, 660 mmol, 98%) as a red oil.

R*_f_* = 0.75 (EtOAc/pentane 1:10).

^1^H-NMR: paramagnetic.

^13^C-NMR: paramagnetic.

(–)-HR-ESI-MS: *m/z* 654.0622 calculated for C_25_H_34_NO_10_Co_2_Si [M–H]^–^; found 654.0627.

IR (neat): 2959 (w), 2880 (w), 2088 (m), 2045 (s), 2005 (s), 1693 (m), 1566 (w), 1457 (w), 1376 (m), 1366 (m), 1244 (w), 1207 (w), 1170 (m), 1145 (w), 1102 (m), 1080 (m), 1007 (m), 964 (w), 851 (w), 809 (w), 737 (w), 575 (w), 517 (m), 499 (m), 459 (w).

${[\alpha]}_{D}^{23}$: – 88.0 (*c* = 1.00, CHCl_3_).

**Synthesis of 14**

*Adapted procedure.*^[1]^ To a solution of boronic ester **SI-24** (161 mg, 362 mmol, 1.00 equiv.) in MeOH (5.0 mL) was added a solution of CuCl_2_ dihydrate (207 mg, 1.21 mmol, 3.35 equiv.) in H_2_O (5.0 mL). The resulting mixture was heated to 80 °C for 3 h. The mixture was cooled down to rt, the solvent was removed under reduced pressure and the crude was dried under high vacuum.

The crude mixture was redissolved in dry MeCN (5.0 mL) and treated with Boc_2_O (119 mg, 543 mmol, 1.50 equiv.) and NEt_3_ (75 mL, 540 mmol, 1.50 equiv.). The resulting mixture was stirred at rt for 1 d. The mixture was diluted with water (50 mL) and the aq. layer was extracted with EtOAc (3 × 50 mL). The combined organic layers were washed with aq. HCl (1 M, 50 mL) and brine (50 mL), dried over MgSO_4_, filtered and the solvent was removed under reduced pressure. Purification by silica gel column chromatography (EtOAc/pentane 16%) delivered product **14a** (84.1 mg, 238 mmol, 66%) as a yellow solid.

R*_f_* = 0.15 (EtOAc/pentane 1:5).

^1^H-NMR (500 MHz, CDCl_3_): δ 8.37 (br. s, 1H), 7.48–7.40 (m, 1H), 7.19 (d, *J* = 7.5 Hz, 1H), 7.09–7.00 (m, 2H), 5.08 (d, *J* = 8.1 Hz, 1H), 4.65 (q, *J* = 6.7 Hz, 1H), 3.67 (s, 3H), 3.35–3.19 (m, 2H), 1.43 (s, 9H) ppm.

^13^C-NMR (101 MHz, CDCl_3_): δ 172.7, 155.3, 133.5, 129.3, 124.9, 123.5, 121.7, 120.5, 117.6, 111.6, 80.1, 54.2, 52.4, 28.5, 28.3 ppm.

(–)-HR-ESI-MS: *m/z* 351.1117 calculated for C_17_H_20_N_2_O_4_Cl [M–H]^–^; found 351.1116.

IR (neat): 3298 (w), 1733 (m), 1694 (s), 1528 (m), 1438 (w), 1368 (m), 1349 (m), 1288 (w), 1230 (m), 1162 (s), 1091 (w), 1048 (w), 1016 (w), 898 (w), 857 (w), 775 (m), 730 (w), 601 (w), 521 (w).

${[\alpha]}_{D}^{23}$: + 34.6 (*c* = 1.00, CHCl_3_).

M.p.: 109.8–110.4 °C.

The obtained data are in agreement with the values reported in the literature.^[1]^

*Adapted procedure.*^[1]^ To a solution of boronic ester **SI-24** (187 mg, 420 mmol, 1.00 equiv.) in MeOH (5.0 mL) was added a solution of CuBr_2_ (281 mg, 1.26 mmol, 3.00 equiv.) in H_2_O (5.0 mL). The resulting mixture was heated to 80 °C for 4 h. The mixture was cooled down to rt, the solvent was removed under reduced pressure and the crude was dried under high vacuum.

The crude mixture was redissolved in dry MeCN (5.0 mL) and treated with Boc_2_O (137 mg, 630 mmol, 1.50 equiv.) and NEt_3_ (90 mL, 630 mmol, 1.50 equiv.). The resulting mixture was stirred at rt for 16 h. The mixture was diluted with water (50 mL) and the aq. layer was extracted with EtOAc (3 × 50 mL). The combined organic layers were washed with aq. HCl (1 M, 50 mL) and brine (50 mL), dried over MgSO_4_, filtered and the solvent was removed under reduced pressure. Purification by silica gel column chromatography (EtOAc/pentane 16%) delivered product **14b** (80.6 mg, 203 mmol, 48%) as a yellow solid.

R*_f_* = 0.25 (EtOAc/pentane 1:5).

^1^H-NMR (500 MHz, CDCl_3_): δ 8.25 (br. s, 1H), 7.50 (d, *J* = 7.9 Hz, 1H), 7.34 (d, *J* = 7.6 Hz, 1H), 7.07 (s, 1H), 7.00 (t, *J* = 7.8 Hz, 1H), 5.07 (d, *J* = 8.6 Hz, 1H), 4.64 (d, *J* = 6.6 Hz, 1H), 3.67 (s, 3H), 3.34–3.20 (m, 2H), 1.43 (s, 9H) ppm.

^13^C-NMR (126 MHz, CDCl_3_): δ 172.7, 155.3, 135.0, 129.0, 124.7, 123.4, 121.0, 118.3, 111.9, 104.9, 80.1, 54.2, 52.5, 28.5, 28.4 ppm.

(+)-HR-ESI-MS: *m/z* 419.0577 calculated for C_17_H_21_N_2_O_4_BrNa [M+Na]^+^; found 419.0581.

IR (neat): 1734 (m), 1695 (s), 1565 (w), 1436 (m), 1391 (w), 1299 (m), 1218 (m), 1166 (m), 1062 (m), 1016 (m), 945 (w), 829 (m), 773 (m), 729 (m), 678 (w), 554 (w).

${[\alpha]}_{D}^{23}$: + 26.2 (*c* = 1.00, CHCl_3_).

M.p.: 99.2–101.3 °C.

The obtained data are in agreement with the values reported in the literature.^[1]^

*Literature procedure.*^[1]^ Cu_2_O (8.3 mg, 58 mmol, 0.20 equiv.) was mixed with aq. NH_3_ (25%, 113 mL, 727 mmol, 2.50 equiv.) and stirred for 15 min at rt under air. Boronic ester **SI-24** (130 mg, 291 mmol, 1.00 equiv.), NaI (219 mg, 1.45 mmol, 5.00 equiv.) and MeCN (2.5 mL) were added and stirring at rt under air was continued for 16 h. The mixture was then concentrated under reduced pressure. The residue was taken up in water (50 mL) and the aq. layer was extracted with EtOAc (3 × 100 mL). The combined organic layers were washed with brine (100 mL), dried over MgSO_4_, filtered and the solvent was removed under reduced pressure. Purification by silica gel column chromatography (EtOAc/pentane 17%) delivered product **14c** (54.1 mg, 122 mmol, 42%) as a white solid.

R*_f_* = 0.20 (EtOAc/pentane 1:5).

^1^H-NMR (400 MHz, CDCl_3_): δ 8.13 (s, 1H), 7.53 (dd, *J* = 10.3, 7.7 Hz, 2H), 7.08 (s, 1H), 6.89 (t, *J* = 7.7 Hz, 1H), 5.06 (d, *J* = 8.2 Hz, 1H), 4.64 (dd, *J* = 12.5, 4.9 Hz, 1H), 3.68 (s, 3H), 3.33–3.18 (m, 2H), 1.43 (s, 9H) ppm.

^13^C-NMR (101 MHz, CDCl_3_): δ 172.7, 155.3, 138.1, 131.0, 127.9, 123.2, 121.5, 119.1, 112.1, 80.1, 77.4, 54.2, 52.5, 28.5 ppm.

(+)-HR-ESI-MS: *m/z* 467.0438 calculated for C_17_H_21_N_2_O_4_INa [M+Na]^+^; found 467.0442.

IR (neat): 3385 (w), 2973 (w), 1733 (m), 1694 (s), 1519 (m), 1488 (w), 1436 (m), 1391 (w), 1365 (m), 1346 (m), 1290 (m), 1218 (m), 1205 (m), 1154 (m), 1079 (m), 1051 (w), 1019 (m), 980 (w), 877 (w), 853 (w), 829 (w), 816 (w), 776 (m), 738 (w), 688 (w), 618 (w), 595 (w), 575 (w), 551 (m), 492 (w), 420 (w).

${[\alpha]}_{D}^{23}$: + 41.2, (*c* = 1.05, CHCl_3_).

M.p.: 143.6–144.7 °C.

The obtained data are in agreement with the values reported in the literature.^[1]^

**Synthesis of 18**

Alcohol **12** (6.37 g, 17.3 mmol, 1.50 equiv.) was dissolved in dry THF (150 mL). A solution of NaHMDS (1 M in THF, 23.0 mL, 23.0 mmol, 2.00 equiv.) was slowly added over 10 min at −40 °C. After stirring at this temperature for 30 min 2-fluoronitrobenzene (**19**, 1.22 mL, 11.5 mmol, 1.00 equiv.) was added and the resulting mixture was stirred at −40 °C for 3 h. After warming up to rt the reaction was quenched by the addition of sat. aq. NH_4_Cl (250 mL). The mixture was extracted with EtOAc (3 × 500 mL). The combined organic layers were washed with brine (500 mL), dried over MgSO_4_, filtered and the solvent removed under reduced pressure. The crude mixture was purified by silica gel flash column chromatography (EtOAc/hexanes 2% to 22%) to afford ether **18** (5.29 g, 10.8 mmol, 94%) as a colorless oil

R*_f_* = 0.52 (EtOAc/hexanes 1:8).

^1^H-NMR (400 MHz, DMSO-*d_6_*, 80 °C): δ 7.88 (dd, *J* = 8.1, 1.7 Hz, 1H), 7.67 (ddd, *J* = 8.4, 7.4, 1.7 Hz, 1H), 7.47 (dd, *J* = 8.5, 1.2 Hz, 1H), 7.22 (ddd, *J* = 8.4, 7.4, 1.1 Hz, 1H), 5.54 (br. s, 1H), 4.24 (dt, *J* = 6.8, 3.8 Hz, 1H), 4.18 (dd, *J* = 9.4, 3.4 Hz, 1H), 4.13 (dd, *J* = 9.3, 6.5 Hz, 1H), 1.58 (s, 3H), 1.47 (s, 3H), 1.46 (s, 9H), 0.87 (t, *J* = 7.9 Hz, 9H), 0.52 (q, *J* = 7.9 Hz, 6H) ppm.

^13^C-NMR (101 MHz, DMSO-*d_6_*, 80 °C): δ 148.9, 140.8, 133.7, 124.5, 122.1, 118.0, 100.5, 93.9, 91.9, 79.8, 63.7, 58.2, 27.6 (3C), 25.7 (2C), 6.5 (3C), 3.3 (3C) ppm.

(–)-HR-ESI-MS: *m/z* 525.2193 calculated for C_25_H_38_N_2_O_6_ClSi [M+Cl]^–^; found 525.2197.

IR (neat): 2956 (w), 2876 (w), 1689 (m), 1606 (m), 1586 (w), 1528 (m), 1482 (w), 1456 (w), 1390 (m), 1364 (s), 1268 (m), 1248 (m), 1208 (w), 1165 (m), 1124 (w), 1086 (m), 1063 (m), 1002 (m), 951 (w), 852 (m), 824 (w), 795 (w), 771 (w), 740 (m), 514 (w).

${[\alpha]}_{D}^{23}$: + 107.7 (*c* = 1.00, CHCl_3_).

**Synthesis of 17**

Ether **18** (5.20 g, 10.6 mmol, 1.00 equiv.) was dissolved in dry THF/dioxane (2.5:1, 110 mL) and a solution of vinylmagnesium bromide (0.7 M in THF, 73.1 mL, 53.0 mmol, 5.00 equiv.) was added dropwise by syringe pump over 1.5 h at −40 °C. The resulting mixture was stirred at this temperature for 4 h and then warmed up to rt. The reaction was quenched by the addition of sat. aq. NH_4_Cl (250 mL). The mixture was extracted with EtOAc (3 × 500 mL). The combined organic layers were washed with brine (500 mL), dried over MgSO_4_, filtered and the solvent removed under reduced pressure. The crude mixture was purified by silica gel flash column chromatography (EtOAc/hexanes 1% to 10%) to afford indole **17** (2.01 g, 4.15 mmol, 39%) as a yellowish oil.

R*_f_* = 0.28 (EtOAc/hexanes 1:10).

^1^H-NMR (500 MHz, DMSO-*d_6_*, 80 °C): δ 10.78 (s, 1H), 7.25 (d, *J* = 2.6 Hz, 1H), 7.19 (d, *J* = 7.9 Hz, 1H), 6.90 (t, *J* = 7.7 Hz, 1H), 6.80 (d, *J* = 7.7 Hz, 1H), 6.42 (d, *J* = 2.5 Hz, 1H), 5.54 (br. s, 1H),
4.43–4.35 (m, 1H), 4.29–4.25 (m, 2H), 1.62 (s, 3H), 1.50 (s, 3H), 1.45 (s, 9H), 0.91 (t, *J* = 7.9 Hz, 9H), 0.54 (q, *J* = 7.9 Hz, 6H) ppm.

^13^C-NMR (126 MHz, DMSO-*d_6_*, 80 °C): δ 151.5,^[[4]](#footnote-4)^ 143.2, 129.5, 126.6, 124.6, 118.6, 113.8, 104.6, 102.3, 101.2, 93.8, 90.1, 79.6, 64.0, 58.3, 27.6, 25.6, 6.6, 3.4 ppm.

(–)-HR-ESI-MS: *m/z* 483.2685 calculated for C_27_H_39_N_2_O_4_Si [M–H] ^–^; found 483.2689.

IR (neat): 3375 (w), 2955 (w), 2875 (w), 1690 (m), 1579 (w), 1457 (w), 1392 (m), 1366 (s), 1249 (m), 1167 (m), 1065 (m), 1018 (w), 854 (w), 783 (w), 724 (m).

${[\alpha]}_{D}^{23}$: + 4.6 (*c* = 0.76, CHCl_3_).

**Synthesis of 20**

Indole **17** (550 mg, 1.13 mmol, 1.00 equiv.) was dissolved in dry DMF (20 mL) and cooled to 0 °C. A solution of Br_2_ (58.1 mL, 1.13 mmol, 1.00 equiv.) was added dropwise over 10 min under exclusion from light. The resulting mixture was stirred at this temperature for 2 h and then poured into an ice-cold solution of NaHCO_3_ (142 mg, 1.69 mmol, 1.50 equiv.) and NaHSO_3_ (176 mg, 1.69 mmol, 1.50 equiv.) in water (50 mL). The aq. layer was extracted with EtOAc (3 × 50 mL). The combined organic layers were washed with brine (25 mL), dried over MgSO_4_, filtered and the solvent removed under reduced pressure. The crude mixture was purified by silica gel flash column chromatography (EtOAc/hexanes 2% to 18%) to afford brominated indole **SI-25** (552 mg, 0.980 mmol, 87%) as an orange gum.

R*_f_* = 0.48 (EtOAc/hexanes 1:5).

^1^H-NMR (400 MHz, DMSO-*d_6_*, 80 °C): δ 11.26 (s, 1H), 7.43 (d, *J* = 2.7 Hz, 1H), 7.08 (d, *J* = 7.8 Hz, 1H), 7.03 (t, *J* = 7.7 Hz, 1H), 6.91 (d, *J* = 7.6 Hz, 1H), 5.56 (br. s, 1H), 4.39 (q, *J* = 6.9 Hz, 1H),
4.30–4.24 (m, 2H), 1.62 (s, 3H), 1.50 (s, 3H), 1.44 (s, 9H), 0.89 (t, *J* = 7.9 Hz, 9H), 0.52 (q, *J* = 7.8 Hz, 6H) ppm.

^13^C-NMR (101 MHz, DMSO-*d_6_*, 80 °C): δ 151.3, 143.3, 127.9, 126.2, 124.3, 119.7, 111.6, 105.8, 101.9, 93.8, 90.5, 88.9, 79.6, 68.8, 64.0, 58.3, 27.6 (3C), 25.6 (2C), 6.6 (3C), 3.4 (3C) ppm.

(–)-HR-ESI-MS: *m/z* 561.1790 calculated for C_27_H_38_N_2_O_4_BrSi [M–H] ^–^; found 561.1791.

IR (neat): 3330 (w), 2955 (w), 2875 (w), 1687 (m), 1581 (w), 1516 (w), 1501 (w), 1457 (w), 1392 (m), 1365 (s), 1322 (w), 1253 (m), 1205 (w), 1165 (m), 1086 (m), 1063 (m), 1002 (m), 951 (w), 849 (w), 807 (w), 774 (w), 727 (s), 515 (w).

${[\alpha]}_{D}^{23}$: – 25.5 (*c* = 0.52, CHCl_3_).

Brominated indole **SI-25** (530 mg, 0.940 mmol, 1.00 equiv.) was dissolved in dry CH_2_Cl_2_ (25 mL). TsCl (215 mg, 1.13 mmol, 1.20 equiv.), NaOH (63.9 mg, 1.60 mmol, 1.70 equiv.) and benzyltriethylammonium chloride (42.8 mg, 0.188 mmol, 0.20 equiv.) were added sequentially at 0 °C. The resulting mixture was stirred at rt for 6 h. The reaction was quenched by the addition of sat. aq. NH_4_Cl (25 mL) and the mixture was extracted with CH_2_Cl_2_ (3 × 50 mL). The combined organic layers were washed with brine (50 mL), dried over MgSO_4_, filtered and the solvent removed under reduced pressure. The crude mixture was purified by silica gel flash column chromatography (EtOAc/hexanes 1% to 10%) to afford N-tosylated indole **SI-26** (483 mg, 0.673 mmol, 72%) as a beige foam.

R*_f_* = 0.54 (EtOAc/hexanes 1:10).

^1^H-NMR (400 MHz, DMSO-*d_6_*, 80 °C): δ 7.96 (s, 1H), 7.64 (d, *J* = 8.2 Hz, 2H), 7.37 (d, *J* = 7.7 Hz, 2H), 7.32 (t, *J* = 8.0 Hz, 1H), 7.18 (d, *J* = 8.0 Hz, 1H), 7.15 (d, *J* = 7.8 Hz, 1H), 5.39 (br. s, 1H), 4.22 (dd, *J* = 9.4, 2.8 Hz, 1H), 4.12 (dd, *J* = 9.6, 6.4 Hz, 1H), 4.02 (br. s, 1H), 2.35 (s, 3H), 1.59 (s, 3H), 1.47 (s, 3H), 1.41 (s, 9H), 0.89 (t, *J* = 7.9 Hz, 9H), 0.53 (q, *J* = 8.2 Hz, 6H) ppm.

^13^C-NMR (126 MHz, DMSO-*d_6_*, 80 °C): δ 144.5, 144.4, 135.4, 132.1, 130.5, 128.0, 125.8, 124.8, 123.5, 112.5, 110.6, 101.1, 96.8, 93.5, 91.3, 79.7, 69.5, 64.0, 58.1, 27.6, 25.8, 20.5, 6.5, 3.4 ppm.

(+)-HR-ESI-MS: *m/z* 717.2024 calculated for C_34_H_46_N_2_O_6_BrSSi^+^ [M+H]^+^; found 717.2020.

IR (neat): 2956 (w), 2876 (w), 1687 (m), 1584 (w), 1547 (w), 1490 (w), 1456 (w), 1363 (s), 1336 (w), 1278 (w), 1252 (w), 1223 (w), 1189 (m), 1173 (s), 1121 (m), 1084 (m), 1062 (m), 1007 (m), 982 (m), 948 (w), 851 (w), 809 (w), 777 (w), 725 (s), 691 (m), 657 (m), 624 (w), 602 (w), 568 (s), 541 (m), 525 (w).

${[\alpha]}_{D}^{23}$: + 4.1 (*c* = 0.21, CHCl_3_).

To a solution of Cbz-protected serine **SI-27** (1.06 g, 4.19 mmol, 1.00 equiv.) in dry DMF (10 mL) was added Me(PhO)_3_PI (1.89 g, 4.19 mmol, 1.00 equiv.) in one portion and the resulting mixture was stirred at rt for 6 h. The reaction was quenched by the addition of sat. aq. NH_4_Cl (25 mL). The aq. layer was extracted with EtOAc (3 × 50 mL). The combined organic layers were washed with brine (50 mL), dried over MgSO_4_, filtered and the solvent removed under reduced pressure. The crude mixture was purified by silica gel flash column chromatography (EtOAc/hexanes 2% to 20%) to afford iodide **SI-28** (1.00 g, 2.75 mmol, 66%) as an amorphous white solid.

R*_f_* = 0.52 (EtOAc/hexanes 1:8).

^1^H-NMR (500 MHz, CDCl_3_): δ 7.41–7.30 (m, 5H), 5.63 (d, *J* = 7.6 Hz, 1H), 5.19–5.08 (m, 2H), 4.59 (dt, *J* = 7.8, 3.9 Hz, 1H), 3.80 (s, 3H), 3.64–3.54 (m, 2H) ppm.

^13^C-NMR (126 MHz, CDCl_3_): δ 169.8, 155.6, 136.1, 128.7 (2C), 128.5, 128.3 (2C), 67.5, 54.2, 53.3, 7.5 ppm.

(+)-HR-ESI-MS: *m/z* 385.9860 calculated for C_12_H_14_NO_4_INa^+^ [M+Na]^+^; found 385.9863.

IR (neat): 3333 (w), 3033 (w), 2953 (w), 1717 (s), 1505 (m), 1454 (w), 1438 (w), 1373 (w), 1339 (m), 1296 (m), 1239 (m), 1209 (s), 1119 (w), 1046 (m), 1003 (m), 913 (w), 827 (w), 771 (w), 735 (m), 697 (m), 607 (w).

${[\alpha]}_{D}^{23}$: + 33.2 (*c* = 1.00, CHCl_3_).

M.p.: 71.0–72.4 °C.

The obtained data are in agreement with the values reported in the literature.^[2]^

1,2-dibromoethane (43 mL, 0.50 mmol, 0.80 equiv.) was added to a stirred suspension of zinc dust (328 mg, 5.02 mmol, 8.00 equiv.) in dry DMF (5.0 mL). The resulting mixture was stirred at 50 °C for 30 min. TMSCl (13 mL, 0.10 mmol, 0.16 equiv.) was added at rt and the mixture was stirred vigorously for 30 min. A solution of iodide **SI-28** (911 mg, 2.51 mmol, 4.00 equiv.) in dry DMF (5.0 mL) was added and stirring at rt was continued for 2 h. After letting the reaction mixture stand without stirring for 30 min the supernatant was transferred to a stirred mixture of indole **SI-26** (450 mg, 0.627 mmol, 1.00 equiv.), Pd(OAc)_2_ (14.1 mg, 62.7 mmol, 10 mol%) and SPhos (51.5 mg, 0.125 mmol, 20 mol%) in dry DMF (5.0 mL). The resulting mixture was stirred at 50 °C for 4 h and then filtered through a plug of celite and eluted with EtOAc (100 mL). The filtrate was diluted with water (100 mL), the layers were separated, and the aq. layer was extracted with EtOAc (2 × 100 mL). The combined organic layers were washed with brine (2 × 100 mL), dried over MgSO_4_, filtered and the solvent was removed under reduced pressure. The crude mixture was purified by silica gel flash column chromatography (EtOAc/hexanes 4% to 32%) to afford substituted tryptophan **20** (467 mg, 0.534 mmol, 85%) as an orange oil.

R*_f_* = 0.10 (EtOAc/hexanes 1:5).

^1^H-NMR (500 MHz, DMSO-*d_6_*, 80 °C): δ 7.67 (s, 1H), 7.60 (br. s, 1H), 7.51 (d, *J* = 8.0 Hz, 2H),
7.39–7.16 (m, 9H), 7.07 (d, *J* = 7.4 Hz, 1H), 5.36 (br. s, 1H), 5.02 (s, 2H), 4.42 (ddd, *J* = 9.1, 8.0, 5.2 Hz, 1H), 4.24 (dd, *J* = 9.5, 3.0 Hz, 1H), 4.14 (dd, *J* = 9.5, 6.6 Hz, 1H), 4.00 (br. s, 1H), 3.64 (s, 3H), 3.18 (dd, *J* = 14.9, 5.1 Hz, 1H), 3.13–2.96 (m, 1H), 2.31 (s, 3H), 1.60 (s, 3H), 1.47 (s, 3H), 1.41 (br. s, 9H), 0.90 (t, *J* = 7.9 Hz, 9H), 0.54 (q, *J* = 7.9 Hz, 6H) ppm.

^13^C-NMR (126 MHz, DMSO-*d_6_*, 80 °C): δ 171.5, 155.5, 151.2, 144.6, 143.9, 136.5, 136.1, 133.5, 129.3, 127.9, 127.6, 127.3, 127.0, 125.5, 124.0, 123.8, 116.6, 112.2, 109.2, 101.4, 93.5, 91.0, 79.6, 65.3, 64.1, 58.1, 53.6, 51.5, 27.6, 26.0, 20.5, 6.6, 3.4.

(+)-HR-ESI-MS: *m/z* 896.3583 calculated for C_46_H_59_N_3_O_10_SSiNa [M+Na]^+^; found 896.3578.

IR (neat): 2955 (w), 1702 (m), 1574 (w), 1492 (w), 1456 (w), 1363 (s), 1261 (m), 1212 (m), 1172 (s), 1122 (w), 1088 (m), 1061 (m), 1029 (m), 853 (w), 810 (w), 728 (m), 698 (m), 666 (m), 568 (m), 536 (w), 471 (w).

${[\alpha]}_{D}^{23}$: + 39.9 (*c* = 0.65, CHCl_3_).

**Synthesis of 21**

Oxazolidine **20** (348 mg, 398 mmol, 1.00 equiv.) was dissolved in a mixture of CH_2_Cl_2_ and TFA (5:1, 24 mL) and the resulting mixture was stirred at rt for 30 min. Then it was diluted with water (50 mL) and extracted with CH_2_Cl_2_ (3 × 50 mL). The combined organic layers were washed with brine (50 mL), dried over MgSO_4_, filtered and the solvent was removed under reduced pressure. The obtained crude amine **SI-29** was used in the next step without further purification (assumed quant.).

Crude amine **SI-29** (assumed 292 mg, 398 mmol, 1.00 equiv.) and Fmoc-l-Asn-Trt (**SI-30**, 261 mg, 438 mmol, 1.10 equiv.) were dissolved in dry DMF (15 mL). HATU (166 mg, 438 mmol, 1.10 equiv.) and DIPEA (75 mL, 450 mmol, 1.1 equiv.) were added and the resulting mixture was stirred at rt for 16 h. The reaction was quenched by the addition of sat. aq. NH_4_Cl (200 mL). The aq. layer was extracted with EtOAc (3 × 200 mL). The combined organic layers were washed with brine (200 mL), dried over MgSO_4_, filtered and the solvent was removed under reduced pressure. The crude mixture was purified by silica gel flash column chromatography (EtOAc/hexanes 12% to 100%) to afford amide **21** (477 mg, 3.63 mmol, 91% over two steps) as a beige solid.

R*_f_* = 0.24 (EtOAc/hexanes 1:1).

^1^H-NMR (500 MHz, methanol-*d_4_*): δ 7.77 (d, *J* = 7.6 Hz, 2H), 7.55 (dd, *J* = 7.9, 4.0 Hz, 4H), 7.35 (td, *J* = 7.6, 3.0 Hz, 2H), 7.27–7.13 (m, 25H), 7.06 (dd, *J* = 12.5, 7.5 Hz, 3H), 5.37 (d, *J* = 3.5 Hz, 1H), 5.06–4.97 (m, 2H), 4.63 (dd, *J* = 8.3, 5.1 Hz, 1H), 4.52 (dd, *J* = 9.6, 4.9 Hz, 1H), 4.41 (t, *J* = 8.3 Hz, 1H), 4.29 (dd, *J* = 10.1, 6.4 Hz, 1H), 4.18–4.11 (m, 1H), 4.06 (dd, *J* = 7.6, 5.9 Hz, 1H), 3.68 (s, 3H), 3.63 (q, *J* = 5.8 Hz, 1H), 3.59–3.55 (m, 1H), 3.22 (dd, *J* = 14.8, 4.9 Hz, 1H), 3.06–3.00 (m, 1H),
2.97–2.94 (m, 1H), 2.80–2.75 (m, 1H), 2.18 (s, 3H), 0.84 (t, *J* = 7.9 Hz, 9H), 0.49 (q, *J* = 7.9 Hz, 6H) ppm.

^13^C-NMR (126 MHz, methanol-*d_4_*): δ 173.8, 173.7, 171.6, 158.4, 158.2, 146.8, 146.1, 145.9, 145.6, 144.9, 142.6, 142.4, 138.0, 136.8, 135.8, 130.9, 130.0, 129.6, 129.5, 129.0, 128.8, 128.73, 128.68, 128.21, 128.18, 127.8, 126.7, 126.6, 126.3, 125.7, 120.9, 120.8, 119.4, 113.8, 111.4, 103.3, 91.7, 71.8, 68.7, 68.5, 67.7, 61.2, 56.8, 55.2, 54.0, 52.9, 49.6, 48.1, 38.9, 28.0, 21.5, 7.8, 5.1 ppm.

(–)-HR-ESI-MS: *m/z* 1346.4753 calculated for C_76_H_77_N_5_O_12_SSiCl [M+Cl]^–^; found 1346.4734.

IR (neat): 3357 (w), 2955 (w), 1712 (m), 1492 (m), 1356 (w), 1254 (m), 1190 (w), 1173 (m), 1045 (m), 841 (s), 760 (w), 739 (m), 700 (m), 669 (w), 557 (s).

${[\alpha]}_{D}^{23}$: – 11.9 (*c* = 0.79, MeOH).

M.p.: 92.8–93.6 °C.

**Synthesis of 23**

Alcohol **21** (106 mg, 81.0 mmol, 1.00 equiv.) was dissolved in a mixture of MeCN (4.0 mL) and aq. phosphate buffer (2.0 mL, pH = 6.4, 10 mM) and cooled to 0 °C. PIDA (2.6 mg, 8.1 mmol, 0.10 equiv.), TEMPO (2.5 mg, 16 mmol, 0.20 equiv.) and NaClO_2_ (25.6 mg, 283 mmol, 3.50 equiv.) were added and the resulting mixture was slowly allowed to warm up to rt and stirred for 16 h. The reaction was quenched by the addition of sat. aq. NaHCO_3_ (25 mL). The aq. layer was extracted with Et_2_O (3 × 25 mL). The combined organic layers were dried over MgSO_4_, filtered and the solvent was removed under reduced pressure. The crude mixture was purified by silica gel column chromatography (MeOH/CH_2_Cl_2_ 0% to 10%) to afford carboxylic acid **23** (59.6 mg, 44.9 mmol, 55%) as a white solid.

R*_f_* = 0.27 (MeOH/CH_2_Cl_2_ 10%).

^1^H-NMR (500 MHz, methanol-*d_4_*): δ 7.76 (d, *J* = 7.6 Hz, 2H), 7.63 (d, *J* = 8.1 Hz, 2H), 7.59–7.50 (m, 3H), 7.33 (q, *J* = 8.0 Hz, 2H), 7.29–7.09 (m, 24H), 7.07–6.98 (m, 3H), 5.89 (br. s, 1H), 5.02 (s, 2H), 4.98 (br. s, 1H), 4.82 (dd, *J* = 9.8, 3.5 Hz, 1H), 4.48 (dd, *J* = 9.3, 4.9 Hz, 1H), 4.30 (dd, *J* = 9.6, 5.8 Hz, 1H), 4.09–3.99 (m, 2H), 3.64 (s, 3H), 3.18 (dd, *J* = 15.1, 4.9 Hz, 1H), 3.03 (dd, *J* = 15.3, 3.9 Hz, 1H), 3.00–2.96 (m, 1H), 2.89–2.82 (m, 1H), 2.12 (s, 3H), 0.79 (t, *J* = 7.8 Hz, 9H), 0.45 (q, *J* = 7.9 Hz, 6H) ppm.

^13^C-NMR (126 MHz, methanol-*d_4_*): δ 174.3, 173.6, 171.6, 158.4, 158.2, 146.7, 146.0, 145.9, 145.7, 144.9, 142.6, 142.4, 137.9, 136.0, 135.8, 130.9, 130.0, 129.8, 129.5, 129.0, 128.8, 128.7, 128.5, 128.23, 128.18, 127.7, 127.1, 126.7, 126.3, 125.8, 120.9, 120.8, 119.9, 114.0, 112.3, 102.8, 91.7, 71.8, 71.2, 68.9, 67.7, 59.0, 55.1, 54.3, 52.9, 48.0, 40.4, 27.8, 21.5, 7.8, 5.0 ppm.

(–)-HR-ESI-MS: *m/z* 1324.4779 calculated for C_76_H_74_N_5_O_13_SSi [M–H]^–^; found 1324.4773.

IR (neat): 3354 (w), 2954 (w), 1713 (m), 1490 (m), 1448 (w), 1414 (w), 1358 (w), 1213 (m), 1190 (w), 1174 (m), 1121 (w), 1050 (m), 907 (m), 812 (w), 759 (w), 727 (s), 698 (s), 669 (m), 647 (w), 622 (w), 571 (m), 539 (m).

${[\alpha]}_{D}^{23}$: + 24.7 (*c* = 0.26, CHCl_3_).

M.p.: 132.3–134.0 °C.

**Synthesis of 25**

*Literature procedure.*^[3]^ H-Lys(Boc)-OH (**24**, 21.2 g, 86.0 mmol, 1.00 equiv.) and Na_2_CO_3_ (9.11 g, 86.0 mmol, 1.00 equiv.) were dissolved in water (500 mL) by sonication. The resulting solution was stirred at rt while *N*-ethoxycarbonyl phthalimide (19.4 g, 86.0 mmol, 1.00 equiv.) was added in six portions over 30 min. The resulting mixture was further stirred at this temperature for 20 h. Then it was cooled to 0 °C and the pH was adjusted to 2 by the addition of aq. HCl (1 M, 120 mL). The aq. layer was extracted with EtOAc (2 × 1.0 L). The combined organic layers were washed with brine (1.0 L), dried over MgSO_4_, filtered and the solvent removed under reduced pressure. The resulting crude colorless oil was used in the next step without further purification (assumed quant.).

*Adapted procedure.*^[3]^ Crude **SI-31** (assumed 32.4 g, 86.0 mmol, 1.00 equiv.) was dissolved in dry DMF (150 mL) and cooled to 0 °C. HATU (35.9 g, 94.5 mmol, 1.10 equiv.) and 8-aminoquinoline (13.6 g, 94.5 mmol, 1.10 equiv.) were added. When all the reactants were fully dissolved dry DIPEA (15.6 mL, 94.5 mmol, 1.00 equiv.) was added and the resulting mixture was stirred at this temperature for 1 h and subsequently at rt for 16 h. The reaction was quenched by the addition of sat. aq. NH_4_Cl (1.0 L) at 0 °C. The aq. layer was extracted with EtOAc (2 × 1.5 L). The combined organic layers were washed with brine (1.0 L), dried over MgSO_4_, filtered and the solvent removed under reduced pressure. The crude mixture was purified by silica gel column chromatography (EtOAc/hexane 25% to 50%) and silica gel flash column chromatography (EtOAc/hexanes 2% to 32%) to afford **25** (34.7 g, 69.0 mmol, 80%) as a sticky, white foam.

R*_f_* = 0.15 (EtOAc/hexanes 1:5).

^1^H-NMR (400 MHz, CDCl_3_): δ 10.36 (s, 1H), 8.76–8.67 (m, 2H), 8.17 (dd, *J* = 8.4, 1.1 Hz, 1H), 7.91 (dd, *J* = 5.5, 3.1 Hz, 2H), 7.77 (dd, *J* = 5.5, 3.0 Hz, 2H), 7.53 (d, *J* = 1.1 Hz, 1H), 7.52 (s, 1H), 7.44 (dd, *J* = 8.3, 4.3 Hz, 1H), 5.13 (dd, *J* = 10.9, 5.3 Hz, 1H), 4.56 (br. s, 1H), 3.20–3.05 (m, 2H), 2.67–2.54 (m, 1H), 2.48–2.36 (m, 1H), 1.68–1.44 (m, 4H), 1.40 (s, 9H) ppm.

^13^C-NMR (126 MHz, CDCl_3_): δ 168.3, 167.2, 156.1, 148.1, 138.1, 137.1, 134.4, 133.8, 132.0, 128.2, 127.7, 123.8, 122.2, 121.7, 117.6, 79.2, 55.3, 40.4, 29.6, 28.6, 28.5, 24.0 ppm.

(+)-HR-ESI-MS: *m/z* 503.2289 calculated for C_28_H_31_N_4_O_5_ [M+H]^+^; found 503.2296.

IR (neat): 3345 (w), 2931 (w), 1777 (w), 1711 (s), 1689 (s), 1597 (w), 1526 (s), 1487 (m), 1467 (w), 1426 (w), 1380 (m), 1364 (m), 1326 (m), 1247 (m), 1166 (m), 1102 (w), 1060 (w), 883 (w), 826 (w), 790 (m), 759 (w), 718 (m), 647 (w), 625 (w), 609 (w), 591 (w), 576 (w), 546 (w), 530 (w), 496 (w).

${[\alpha]}_{D}^{23}$: + 3.6 (*c* = 1.00, CHCl_3_).

The obtained data are in agreement with the values reported in the literature.^[3]^

**Synthesis of 27**

*Literature procedure.*^[4]^ A solution of 3-iodoaniline (**26**, 25.2 g, 115 mmol, 1.00 equiv.) in benzene (400 mL) was treated with NBS (20.1 g, 109 mmol, 0.95 equiv.). The resulting mixture was stirred at rt under protection from light for 6 h and then concentrated under reduced pressure. The resulting brown oil was redissolved in Et_2_O (1.0 L) and washed with water (3 × 500 mL) and brine (500 mL). The organic layer was dried over MgSO_4_, filtered and the solvent was removed under reduced pressure. The crude mixture was purified by silica gel flash column chromatography (EtOAc/hexanes 0% to 10%) to afford 2-bromo-5-iodoaniline (**SI-32**, 11.0 g, 36.8 mmol, 32%) as an orange solid. *(Note: this is an exemplary procedure; yields varied between 28-47%)*

R*_f_* = 0.70 (EtOAc/pentane 1:4).

^1^H-NMR (400 MHz, CDCl_3_): δ 7.11–7.08 (m, 2H), 6.91 (dd, *J* = 8.4, 2.0 Hz, 1H), 4.08 (br. s, 2H) ppm.

^13^C-NMR (101 MHz, CDCl_3_): δ 145.5, 134.0, 128.3, 124.2, 109.7, 93.1 ppm.

(+)-HR-ESI-MS: *m/z* 297.8723 calculated for C_6_H_6_NBrI [M+H]^+^; found 297.8722.

IR (neat): 3430 (w), 3308 (w), 2988 (w), 1611 (m), 1578 (m), 1471 (m), 1396 (m), 1295 (w), 1252 (w), 1146 (w), 1051 (m), 1015 (s), 874 (w), 843 (m), 778 (s), 601 (w), 563 (m), 432 (s).

M.p.: 68.2–69.2 °C.

The obtained data are in agreement with the values reported in the literature.^[4]^

To a solution of 2-bromo-5-iodoaniline (**SI-32**, 10.8 g, 36.2 mmol, 1.00 equiv.) in dry toluene (75 mL) were added DMAP (442 mg, 3.62 mmol, 0.10 equiv.) and acetic anhydride (20.4 mL, 217 mmol, 6.00 equiv.). After briefly stirring at rt the mixture was heated to reflux for 20 h. Then it was allowed to cool down to rt and the solvent was removed under reduced pressure. The residue was taken up in EtOAc (500 mL). The organic layer was extracted with sat. aq. NaHCO_3_ (500 mL), water (3 × 500 mL) and brine (500 mL). The organic layer was dried over MgSO_4_, filtered and the solvent removed under reduced pressure. The crude mixture was purified by silica gel flash column chromatography (EtOAc/hexanes 2% to 20%) to afford mono-acetylated aniline **SI-33** (0.78 g, 2.3 mmol, 6%) as a white solid and bis-acetylated aniline **SI-34** (12.7 g, 33.2 mmol, 92%) as a white solid.^[[5]](#footnote-5)^

**Analytical data for SI-33** **(R = H):**

R*_f_* = 0.20 (EtOAc/hexanes 1:10).

^1^H-NMR (500 MHz, CDCl_3_): δ 8.73 (s, 1H), 7.52 (br. s, 1H), 7.29 (dd, *J* = 8.4, 2.1 Hz, 1H), 7.23 (d, *J* = 8.4 Hz, 1H), 2.23 (s, 3H) ppm.

^13^C-NMR (101 MHz, CDCl_3_): δ 168.3, 136.9, 134.2, 133.5, 130.4, 112.9, 93.2, 25.0 ppm.

(+)-HR-ESI-MS: *m/z* 339.8829 calculated for C_8_H_8_NOBrI [M+H]^+^; found 339.8831.

IR (neat): 3267 (w), 2988 (w), 1660 (m), 1564 (m), 1512 (m), 1455 (m), 1388 (s), 1280 (m), 1249 (m), 1073 (m), 1030 (s), 968 (w), 872 (w), 796 (s), 683 (w), 661 (w), 644 (m), 601 (w), 543 (m), 474 (w), 435 (m).

M.p.: 183.0–184.2 °C.

The obtained data are in agreement with the values reported in the literature.^[3]^

**Analytical data for SI-34 (R = Ac):**

R*_f_* = 0.40 (EtOAc/hexanes 1:10).

^1^H-NMR (400 MHz, CDCl_3_): δ 7.61 (dd, *J* = 8.3, 2.2 Hz, 1H), 7.59 (d, *J* = 2.0 Hz, 1H), 7.42 (d, *J* = 8.3 Hz, 1H), 2.30 (s, 6H) ppm.

^13^C-NMR (101 MHz, CDCl_3_): δ 172.0, 140.1, 139.7, 139.4, 135.2, 124.1, 92.7, 26.7 ppm.

(+)-HR-ESI-MS: *m/z* 381.8934 calculated for C_10_H_10_NO_2_BrI [M+H]^+^; found 381.8933.

IR (neat): 1705 (s), 1564 (w), 1459 (w), 1423 (w), 1379 (w), 1360 (m), 1295 (w), 1232 (s), 1202 (s), 1135 (w), 1078 (w), 1044 (w), 1027 (m), 1014 (m), 952 (w), 914 (w), 890 (w), 807 (m), 711 (m), 666 (w), 639 (w), 621 (w), 599 (m), 541 (m), 458 (m), 429 (w).

M.p.: 89.9–91.1 °C.

Freshly dried *m*CPBA (9.46 g, 38.4 mmol, 1.10 equiv.) was dissolved in dry CH_2_Cl_2_ (150 mL). At 0 °C a solution of **SI-33/SI-34** (13.3 g, 34.9 mmol, 1.00 equiv.) in dry CH_2_Cl_2_ (150 mL) was added. Subsequently, TfOH (7.50 mL, 59.3 mmol, 1.70 equiv.) was slowly added resulting in an orange-red solution which was stirred at 0 °C for 15 min and then warmed up to rt. After stirring at this temperature for 1 h mesitylene (5.30 mL, 38.4 mmol, 1.10 equiv.) was added at 0 °C. The resulting mixture was warmed up to rt and stirred for 20 h. Then the solvent was removed under reduced pressure to leave a brown residue which was taken up in Et_2_O (200 mL). The mixture was briefly heated to 40 °C to fully dissolve it and then stored at 4 °C for 20 h. A grey precipitate was observed which was collected by filtration. Washing with ice-cold Et_2_O and drying under high vacuum afforded iodonium salt **27** (20.4 g, 33.5 mmol, 96%) as a grey powder.

^1^H-NMR (400 MHz, DMSO-*d_6_*): δ 9.66 (s, 1H), 8.35 (d, *J* = 2.2 Hz, 1H), 7.77 (d, *J* = 8.6 Hz, 1H), 7.63 (dd, *J* = 8.6, 2.3 Hz, 1H), 7.23 (s, 2H), 2.60 (s, 6H), 2.30 (s, 3H), 2.10 (s, 3H) ppm.

^13^C-NMR (101 MHz, DMSO-*d_6_*): δ 169.1, 143.2, 141.6, 138.8, 135.6, 131.6, 131.4, 129.8, 122.8, 120.7, 119.1, 112.4, 26.3, 23.4, 20.5 ppm.

(+)-HR-ESI-MS: *m/z* 457.9611 calculated for C_17_H_18_NOBrI [M+H]^+^; found 457.9611.

IR (neat): 3408 (w), 1690 (w), 1664 (w), 1563 (w), 1506 (m), 1449 (w), 1390 (m), 1269 (m), 1236 (s), 1221 (s), 1157 (m), 1023 (s), 877 (w), 848 (w), 805 (w), 758 (w), 634 (s), 601 (w), 575 (m), 548 (m), 518 (m), 440 (w).

M.p.: 174.2–175.3 °C.

**Synthesis of 28 & 29**

An oven dried Schlenk tube was charged with a big stirring bar, molecular sieves (3 Å), lysine derivative **25** (2.00 g, 3.98 mmol, 1.00 equiv.), iodonium salt **27** (3.63 g, 5.97 mmol, 1.50 equiv.), AgOAc (1.33 g, 7.96 mmol, 2.00 equiv.) and Pd(OAc)_2_ (313 mg, 1.39 mmol, 0.35 equiv.). Dry, degassed *t*BuOH (40 mL) was added. The resulting mixture was sonicated for 2 min and then heated to 80 °C for 16 h. After cooling down to rt the beige suspension was diluted with CH_2_Cl_2_ (50 mL) and filtered through a pad of celite which was thoroughly rinsed with CH_2_Cl_2_. The solvent of the filtrate was removed under reduced pressure. Purification by silica gel flash column chromatography (EtOAc/hexane 25% to 100%) delivered a mixture of diastereomers **SI-35** (1.27 g, 1.78 mmol, 45%, d.r. 1:5) as a foamy yellowish solid. *(Note: this is an exemplary procedure; yields varied between 40-48%, and d.r. between 1:4 and 1:6)*

For characterization, the two diastereomers were separated by chiral semi-preparative HPLC (Daicel Chemical Industries Inc., CHIRALPAK AD 10 µm, 20 × 250 mm, 2-propanol/hexanes 50%, 9.5 mL/min, t_R_ (**(*S*,*S*)-SI-35**) = 32.5 min, t_R_ (**(*S*,*R*)-SI-35**) = 65.9 min).

**Analytical data for (*S*,*S*)-SI-35 (minor):**

R*_f_* = 0.18 (EtOAc/hexanes 1:1).

^1^H-NMR (400 MHz, CDCl_3_): δ 10.60 (br. s, 1H), 8.89 (d, *J* = 3.4 Hz, 1H), 8.75 (dd, *J* = 6.3, 2.8 Hz, 1H), 8.22–8.15 (m, 2H), 7.73 (dd, *J* = 5.4, 3.1 Hz, 2H), 7.61 (dd, *J* = 5.5, 3.0 Hz, 2H), 7.57–7.52 (m, 2H), 7.48 (dd, *J* = 8.0, 4.0 Hz, 1H), 7.43 (br. s, 1H), 7.34 (d, *J* = 8.1 Hz, 1H), 6.91 (d, *J* = 7.4 Hz, 1H), 5.25 (d, *J* = 11.4 Hz, 1H), 4.79 (br. s, 1H), 4.27 (td, *J* = 12.0, 2.9 Hz, 1H), 3.16–2.97 (m, 2H), 2.20 (s, 3H), 2.10–2.00 (m, 1H), 1.80–1.71 (m, 1H), 1.44–1.37 (m, 2H), 1.33 (s, 9H) ppm.

^13^C-NMR (126 MHz, CDCl_3_): δ 168.4, 167.7, 166.5, 156.3, 148.7, 140.7, 138.5, 136.8, 135.7, 134.2, 133.9, 132.3, 131.4, 128.2, 127.5, 125.6, 123.7, 122.7, 122.2, 121.9, 118.1, 79.1, 60.4, 43.4, 40.0, 31.1, 28.5, 27.4, 24.9 ppm.

(+)-HR-ESI-MS: *m/z* 714.1922 calculated for C_36_H_37_N_5_O_6_Br [M+H]^+^; found 714.1928.

IR (neat): 3321 (w), 2925 (m), 2854 (w), 1776 (w), 1717 (s), 1580 (w), 1529 (s), 1487 (m), 1466 (w), 1425 (m), 1377 (m), 1326 (w), 1244 (w), 1170 (m), 1032 (w), 879 (w), 828 (w), 792 (w), 758 (w), 720 (m), 607 (w), 531 (w).

${[\alpha]}_{D}^{23}$: – 48.6 (*c* = 0.30, CHCl_3_).

**Analytical data for (*S*,*R*)-SI-35 (major):**

R*_f_* = 0.18 (EtOAc/hexanes 1:1).

^1^H-NMR (400 MHz, CDCl_3_): δ 9.91 (br. s, 1H), 8.70 (d, *J* = 4.0 Hz, 1H), 8.53 (d, *J* = 7.0 Hz, 1H), 8.44 (br. s, 1H), 8.09 (d, *J* = 8.5 Hz, 1H), 7.92 (dd, *J* = 5.5, 3.0 Hz, 2H), 7.76 (dd, *J* = 5.6, 3.0 Hz, 2H), 7.50 (br. s, 1H), 7.46–7.37 (m, 4H), 7.09 (d, *J* = 8.1 Hz, 1H), 5.27 (d, *J* = 11.2 Hz, 1H), 4.59 (br. s, 1H), 4.23 (td, *J* = 11.2, 4.0 Hz, 1H), 3.10–3.00 (m, 1H), 3.00–2.90 (m, 1H), 2.19 (s, 3H), 1.75–1.59 (m, 2H), 1.34 (s, 9H), 1.32–1.26 (m, 2H) ppm.

^13^C-NMR (126 MHz, CDCl_3_): δ 168.4, 168.3, 165.9, 156.1, 148.1, 141.1, 138.2, 136.5, 136.3, 134.5, 133.8, 132.9, 131.8, 127.9, 127.4, 125.8, 124.0, 122.1, 121.6, 117.4, 79.1, 60.9, 43.5, 40.0, 29.9, 28.5, 27.0, 24.9 ppm.

(+)-HR-ESI-MS: *m/z* 714.1922 calculated for C_36_H_37_N_5_O_6_Br [M+H]^+^; found 714.1927.

IR (neat): 3313 (w), 2928 (w), 1776 (w), 1714 (s), 1580 (m), 1523 (s), 1487 (m), 1468 (m), 1422 (m), 1381 (s), 1365 (s), 1326 (w), 1247 (m), 1167 (s), 1112 (m), 1083 (m), 1032 (w), 910 (m), 827 (m), 791 (m), 723 (s), 647 (m), 548 (m), 530 (m), 459 (w).

${[\alpha]}_{D}^{23}$: – 0.2 (*c* = 1.00, CHCl_3_).

Phthalimide-protected amine **SI-35** (4.68 g, 6.55 mmol, 1.00 equiv., mixture of diastereomers) was dissolved in a solution of hydrazine hydrate in MeOH (3.0 M, 75 mL) and stirred at rt for 16 h. The reaction was quenched by the addition of sat. aq. NaHCO_3_ (150 mL) and the aq. layer was extracted with EtOAc (3 × 250 mL). The combined organic layers were washed with brine (250 mL), dried over MgSO_4_, filtered and the solvent was removed under reduced pressure. The resulting crude yellow foam was used in the next step without further purification (assumed quant.).

A solution of crude amine **SI-36** (assumed 3.83 g, 6.55 mmol, 1.00 equiv., mixture diastereomers) in dry DMF (60 mL) was treated with Fmoc-Ser(O*t*Bu)-OH (2.51 g, 6.55 mmol, 1.00 equiv.). The resulting mixture was cooled to 0 °C and HATU (2.99 g, 7.86 mmol, 1.20 equiv.), and NMM (1.08 mL, 9.83 mmol, 1.50 equiv.) were added. The reaction mixture was stirred at this temperature for 1 h and at rt for 23 h. The reaction was quenched by the addition of dry MeOH (5.0 mL) at rt and stirred for 30 min. Sat. aq. NH_4_Cl (250 mL) was added, and the aq. layer was extracted with EtOAc (3 × 500 mL). The combined organic layers were washed with brine (500 mL), dried over MgSO_4_, filtered and the solvent was removed under reduced pressure. Purification by silica gel flash column chromatography (EtOAc/hexane 40% to 80%) delivered the products **28** (848 mg, 0.893 mmol, 14%) and **29** (4.40 g, 4.63 mmol, 71%) as foamy white solids. *(Note: this is an exemplary procedure; ratio between* ***28*** *and* ***29*** *were dependent on the diastereomeric ratio of the starting material.)*

**Analytical data for 28** (minor):

R*_f_* = 0.48 (EtOAc/hexanes 2:1).

^1^H-NMR (500 MHz, CDCl_3_): δ 10.25 (s, 1H), 8.76 (d, *J* = 3.0 Hz, 1H), 8.58 (d, *J* = 4.5 Hz, 1H), 8.27 (s, 1H), 8.10 (d, *J* = 8.2 Hz, 1H), 7.70 (d, *J* = 7.6 Hz, 1H), 7.61 (d, *J* = 7.6 Hz, 2H), 7.56 (s, 1H),
7.51–7.46 (m, 1H), 7.44 (d, *J* = 7.6 Hz, 1H), 7.41–7.27 (m, 5H), 7.22 (t, *J* = 7.4 Hz, 1H), 7.18 (t, *J* = 7.5 Hz, 1H), 7.00 (t, *J* = 7.4 Hz, 1H), 6.92 (d, *J* = 8.4 Hz, 1H), 5.28 (dd, *J* = 9.8, 4.1 Hz, 1H), 4.73 (br. s, 1H), 4.69–4.60 (m, 1H), 4.35 (dd, *J* = 10.5, 7.9 Hz, 1H), 4.29 (dd, *J* = 10.5, 7.5 Hz, 1H), 4.17 (t, *J* = 7.7 Hz, 1H), 4.05 (d, *J* = 6.7 Hz, 1H), 3.67–3.60 (m, 1H), 3.57 (dd, *J* = 8.6, 4.4 Hz, 1H), 3.17 (br. s, 1H), 3.07 (dq, *J* = 13.0, 6.6 Hz, 1H), 2.19 (s, 3H), 1.99–1.90 (m, 1H), 1.78 (dtd, *J* = 13.8, 9.1, 5.0 Hz, 1H), 1.61–1.47 (m, 2H), 1.42 (s, 9H), 1.25 (s, 9H) ppm.

^13^C-NMR (126 MHz, CDCl_3_): δ 171.5, 168.2, 168.0, 156.8, 156.1, 148.3, 144.1, 143.8, 141.3, 141.2, 140.1, 138.0, 136.8, 135.6, 133.5, 132.5, 128.0, 127.9, 127.7, 127.6, 127.4, 127.2, 127.02, 126.98, 125.4, 125.2, 122.2, 121.7, 121.2, 120.1, 120.0, 119.9, 117.2, 112.6, 79.1, 73.9, 67.2, 62.8, 57.0, 55.9, 47.1, 40.3, 28.8, 28.6, 27.9, 27.5, 24.9 ppm.

(+)-HR-ESI-MS: *m/z* 949.3494 calculated for C_50_H_58_N_6_O_8_Br [M+H]^+^; found 949.3495.

IR (neat): 3312 (w), 2973 (w), 1675 (s), 1579 (w), 1520 (s), 1486 (s), 1450 (m), 1423 (m), 1390 (w), 1364 (m), 1325 (w), 1246 (m), 1167 (m), 1086 (m), 1034 (w), 984 (w), 880 (w), 827 (w), 791 (w), 759 (m), 740 (m), 639 (w), 621 (w), 586 (w), 544 (w), 459 (w), 426 (w).

${[\alpha]}_{D}^{23}$: – 21.6 (*c* = 1.00, CHCl_3_).

**Analytical data for 29** (major):

R*_f_* = 0.37 (EtOAc/hexanes 2:1).

^1^H-NMR (500 MHz, CDCl_3_): δ 9.96 (br. s, 1H), 8.77 (br. s, 1H), 8.62 (br. s, 1H), 8.28 (s, 1H), 8.14 (d, *J* = 8.3 Hz, 1H), 7.76 (t, *J* = 7.4 Hz, 2H), 7.66 (d, *J* = 7.5 Hz, 1H), 7.61 (d, *J* = 7.6 Hz, 1H), 7.55–7.45 (m, 3H), 7.45–7.34 (m, 5H), 7.34–7.23 (m, 2H), 6.93 (d, *J* = 7.6 Hz, 1H), 6.31 (br. s, 1H), 5.02 (t, *J* = 7.6 Hz, 1H), 4.69 (br. s, 1H), 4.48 (br. s, 1H), 4.40 (qd, *J* = 10.5, 7.3 Hz, 3H), 4.26 (t, *J* = 7.4 Hz, 1H), 3.98–3.92 (m, 1H), 3.61 (t, *J* = 7.8 Hz, 1H), 3.27 (br. s, 1H), 3.16–3.03 (m, 2H), 2.19 (s, 3H), 2.01–1.96 (m, 1H), 1.95–1.88 (m, 1H), 1.49–1.42 (m, 2H), 1.40 (s, 9H), 1.24 (s, 9H) ppm.

^13^C-NMR (126 MHz, CDCl_3_): δ 170.8, 168.1, 168.0, 156.6, 156.0, 148.3, 144.1, 143.9, 141.3, 140.6, 138.2, 135.8, 133.6, 132.5, 127.9, 127.8, 127.7, 127.3, 127.1, 125.4, 125.3, 125.1, 122.3, 122.2, 121.7, 120.0, 117.0, 112.1, 79.0, 74.4, 67.3, 62.0, 59.1, 55.0, 48.0, 47.2, 40.3, 28.5, 28.0, 27.9, 27.52, 24.9 ppm.

(+)-HR-ESI-MS: *m/z* 949.3494 calculated for C_50_H_58_N_6_O_8_Br [M+H]^+^; found 949.3499.

IR (neat): 3314 (w), 2973 (w), 1664 (m), 1579 (w), 1518 (m), 1488 (m), 1450 (w), 1424 (w), 1389 (m), 1366 (m), 1324 (w), 1248 (m), 1166 (m), 1097 (w), 1033 (w), 840 (s), 792 (w), 760 (m), 740 (m), 663 (w), 621 (w), 557 (m), 459 (w), 427 (w).

${[\alpha]}_{D}^{23}$: – 35.7 (*c* = 1.00, CHCl_3_).

**Synthesis of 30**

Boc-l-Ser(Bn)-OH (**SI-37**, 7.14 g, 24.2 mmol, 1.00 equiv.) was dissolved in CH_2_Cl_2_ (30 mL) and treated with TFA (6.0 mL). After stirring at rt for 1 h the solvent was removed under reduced pressure. Recrystallization of the residue from Et_2_O afforded a crude white solid which was directly used in the next step without further purification (assumed quant.).

*Literature procedure.*^[5]^ Crude **SI-38** (assumed 4.72 g, 24.2 mmol, 1.00 equiv.) was dissolved in a mixture of aq. Na_2_CO_3_ (10%, 50 mL) and dioxane (50 mL). FmocCl (6.26 g, 24.2 mmol, 1.00 equiv.) was added at 0 °C. The resulting mixture was stirred at this temperature for 1 h and at rt for 64 h. The reaction was carefully quenched by the addition of aq. HCl (1 M, 100 mL) at 0 °C. The aq. layer was extracted with CH_2_Cl_2_ (3 × 500 mL). The combined organic layers were dried over MgSO_4_, filtered and the solvent was removed under reduced pressure. Recrystallization from Et_2_O/hexane (1:2) afforded product **30** (7.26 g, 17.4 mmol, 72% over two steps) as a white solid.

^1^H-NMR (400 MHz, DMSO-*d_6_*): δ 12.80 (s, 1H), 7.89 (d, *J* = 7.5 Hz, 2H), 7.74 (t, *J* = 8.4 Hz, 2H), 7.42 (t, *J* = 7.4 Hz, 2H), 7.40–7.23 (m, 7H), 4.57–4.45 (m, 2H), 4.32–4.18 (m, 4H), 3.77–3.65 (m, 2H) ppm.

^13^C-NMR (101 MHz, CDCl_3_): δ 174.7, 156.3, 144.0, 143.8, 141.5, 137.2, 128.7, 128.2, 127.9, 127.2, 125.31, 125.26, 120.1, 73.7, 69.6, 67.5, 54.3, 47.2 ppm.

(+)-HR-ESI-MS: *m/z* 418.1649 calculated for C_25_H_24_NO_5_ [M+H]^+^; found 418.1651.

IR (neat): 3385 (w), 2947 (w), 1764 (m), 1694 (m), 1539 (m), 1449 (m), 1392 (w), 1371 (w), 1317 (w), 1233 (m), 1180 (m), 1105 (m), 1086 (m), 1057 (m), 1036 (m), 1018 (m), 956 (w), 905 (w), 836 (w), 758 (m), 739 (s), 699 (m), 644 (m), 620 (w), 593 (w), 543 (m), 477 (w), 430 (w).

${[\alpha]}_{D}^{23}$: + 11.1 (*c* = 1.00, aq. NaOH (1 M)).

M.p.: 140.0–140.6 °C.

The obtained data are in agreement with the values reported in the literature.^[5]^

**Synthesis of *N*-Boc protected 31 (SI-40)**

*Literature procedure.*^[6]^ Boc-l-Phe (**SI-39**, 5.00 g, 18.8 mmol, 1.00 equiv.) and K_2_CO_3_ (13.0 g, 94.0 mmol, 5.00 equiv.) were suspended in acetone (250 mL). BnBr (4.50 mL, 37.6 mmol, 2.00 equiv.) was added and the resulting mixture was stirred at 60 °C for 24 h. After cooling down to rt the solids were removed by filtration and the filtrate was concentrated under reduced pressure. Purification by silica gel flash column chromatography (EtOAc/hexane 1% to 15%) delivered product **SI-40** (6.42 g, 18.1 mmol, 96%) as a white solid.

R*_f_* = 0.25 (EtOAc/hexane 1:10).

^1^H-NMR (400 MHz, CDCl_3_): δ 7.40–7.33 (m, 3H), 7.32–7.27 (m, 2H), 7.25–7.20 (m, 3H), 7.08–7.01 (m, 2H), 5.14 (q, *J* = 12.3 Hz, 2H), 4.97 (d, *J* = 8.4 Hz, 1H), 4.63 (q, *J* = 6.4 Hz, 1H), 3.16–3.01 (m, 2H), 1.41 (s, 9H) ppm.

^13^C-NMR (101 MHz, CDCl_3_): δ 171.9, 155.2, 136.0, 135.3, 129.5, 128.73, 128.69, 128.61, 127.1, 80.1, 67.3, 54.6, 38.4, 28.4 ppm.

(+)-HR-ESI-MS: *m/z* 356.1856 calculated for C_21_H_26_NO_4_ [M+H]^+^; found 356.1856.

IR (CHCl_3_): 3374 (w), 3031 (w), 2977 (w), 2931 (w), 1741 (m), 1713 (s), 1497 (m), 1455 (m), 1391 (w), 1366 (m), 1350 (m), 1250 (m), 1212 (m), 1163 (s), 1080 (w), 1054 (w), 1020 (w), 913 (w), 860 (w), 749 (m), 698 (s), 602 (w), 489 (w).

${[\alpha]}_{D}^{23}$: + 1.4 (*c* = 0.50, CHCl_3_).

M.p.: 66.2–67.1 °C.

The obtained data are in agreement with the values reported in the literature.^[6]^

**Synthesis of *N*-Fmoc protected 32 (SI-41)**

Boc-l-Phe-OBn (**SI-40**, 2.60 g, 7.32 mmol, 1.00 equiv.) was dissolved in CH_2_Cl_2_ (30 mL) and treated with TFA (6.0 mL). After stirring at rt for 30 min the reaction was quenched by the addition of aq. NaOH (1 M, 40 mL). The aq. Layer was extracted with CH_2_Cl_2_ (3 × 100 mL). The combined organic layers were dried over MgSO_4_, filtered and the solvent was removed under reduced pressure delivering crude **31** which was used in the next step without further purification (assumed quant.).

Carboxylic acid **30** (3.06 g, 7.32 mmol, 1.00 equiv.) and amine **31** (1.87 g, 7.32 mmol, 1.00 equiv.) were dissolved in dry DMF (20 mL). HATU (3.34 g, 8.78 mmol, 1.20 equiv.) and NMM (970 µL, 8.78 mmol, 1.20 equiv.) were added at 0 °C. The resulting mixture was stirred at rt for 16 h and quenched by the addition of sat. aq. NH_4_Cl (250 mL). The aq. layer was extracted with EtOAc (3 × 250 mL). The combined organic layers were washed with sat. aq. NaHCO_3_ (250 mL), water (250 mL), and brine (250 mL), dried over MgSO_4_, filtered and the solvent was removed under reduced pressure. Recrystallization from EtOAc/hexane (1:2) delivered product **SI-41** (4.79 g, 7.32 mmol, quant. over two steps) as a white solid.

^1^H-NMR (400 MHz, CDCl_3_): δ 7.71 (d, *J* = 7.5 Hz, 2H), 7.51 (d, *J* = 7.5 Hz, 2H), 7.34 (t, *J* = 7.5 Hz, 2H), 7.32–7.16 (m, 11H), 7.16–6.97 (m, 4H), 6.90 (d, *J* = 7.1 Hz, 2H), 5.60 (d, *J* = 6.7 Hz, 1H), 5.11 (d, *J* = 12.2 Hz, 1H), 5.03 (d, *J* = 12.1 Hz, 1H), 4.88–4.78 (m, 1H), 4.47–4.36 (m, 2H), 4.31 (d, *J* = 7.1 Hz, 2H), 4.14 (t, *J* = 7.3 Hz, 1H), 3.86–3.78 (m, 1H), 3.43 (t, *J* = 8.3 Hz, 1H), 3.08 (dd, *J* = 13.9, 5.8 Hz, 1H), 2.99 (dd, *J* = 13.9, 6.0 Hz, 1H) ppm.

^13^C-NMR (101 MHz, CDCl_3_): δ 171.0, 169.8, 156.1, 144.0, 143.8, 141.43, 141.42, 137.3, 135.6, 135.2, 129.4, 128.8, 128.69, 128.66, 128.14, 128.07, 127.9, 127.2, 125.2, 120.1, 73.6, 69.7, 53.7, 47.2, 37.8 ppm.

(+)-HR-ESI-MS: *m/z* 655.2803 calculated for C_41_H_39_N_2_O_6_^+^ [M+H]^+^; found 655.2812.

IR (neat): 3290 (w), 2971 (w), 1722 (m), 1652 (s), 1514 (m), 1448 (w), 1386 (m), 1349 (w), 1235 (m), 1190 (m), 1107 (m), 1066 (m), 1043 (m), 949 (w), 915 (w), 839 (s), 756 (m), 737 (m), 700 (m), 666 (w), 621 (w), 599 (w), 588 (w), 558 (m), 513 (w), 460 (w), 441 (w), 426 (w).

${[\alpha]}_{D}^{23}$: – 11.3 (*c* = 0.96, CHCl_3_).

**Synthesis of *N*-Fmoc protected 33 (SI-44)**

Quinolinamide **28** (597 mg, 628 µmol, 1.00 equiv.) was dissolved in dry CH_2_Cl_2_ (10 mL) and dry pyridine (150 µL, 1.88 mmol, 3.00 equiv.) was added. The resulting mixture was cooled to –78 °C and the reaction flask was purged with N_2_ and O_2_. Then ozone was passed through the stirred mixture for 15 min. The flask was flushed with O_2_ and N_2_ and the mixture was warmed up to rt. The solvent was removed under reduced pressure.

The residue was redissolved in a mixture of water and THF (1:4, 10 mL) and aq. H_2_O_2_ (30%, 590 µL, 6.28 µmol, 10.0 equiv.) was added at rt. The resulting mixture was cooled to 0 °C and LiOH∙H_2_O (158 mg, 3.77 mmol, 6.00 equiv.) was added. The mixture was stirred at this temperature for 2 h, after which the pH was adjusted to 2 by the addition of aq. H_2_SO_4_ (1 M) and the reaction was quenched by the addition of sat aq. Na_2_SO_3_ (10 mL). After warming up to rt the aq. layer was acidified to pH 2 again by the addition of aq. H_2_SO_4_ (1 M). The aq. layer was extracted with EtOAc (3 × 50 mL). The combined organic layers were washed with brine (50 mL), dried over MgSO_4_, filtered and the solvent was removed under reduced pressure. Purification by silica gel column chromatography (MeOH/CH_2_Cl_2_ 0% to 5%, + 0.2% AcOH) followed by co-evaporation with toluene for the removal of AcOH delivered carboxylic acid **SI-42** (169 mg, 205 µmol, 33%) as a colorless gum and primary amide **SI-43** (141 mg, 171 µmol, 27%) as a colorless gum. *(Notes: removal of the Fmoc protecting group was observed in some cases during hydrolysis of the ozonolyzed intermediate with LiOOH. In these cases, reprotection of the primary amine was achieved by treatment of the reaction mixture with Fmoc-Osu (1.00 equiv.) and stirring for 16 h at rt. Since the separation of* ***SI-42*** *and* ***SI-43*** *can be quite challenging, mixed fractions can be engaged in the next step as well, but it must be noted that the yield of the next step will drop significantly.)*

**Analytical data for SI-42**:

R*_f_* = 0.21 (MeOH/CH_2_Cl_2_ 5%).

^1^H-NMR (500 MHz, methanol-*d_4_*): δ 7.77 (d, *J* = 7.7 Hz, 2H), 7.75–7.72 (m, 1H), 7.64 (d, *J* = 7.6 Hz, 1H), 7.61 (d, *J* = 7.5 Hz, 1H), 7.52 (d, *J* = 8.3 Hz, 1H), 7.41 (d, *J* = 9.4 Hz, 1H), 7.36 (t, *J* = 7.5 Hz, 2H), 7.28–7.20 (m, 2H), 6.93 (d, *J* = 8.2 Hz, 1H), 4.92 (br. s, 1H), 4.43–4.37 (m, 1H), 4.34 (br. s, 1H), 4.24–4.14 (m, 2H), 3.83 (dd, *J* = 8.9, 4.0 Hz, 1H), 3.58 (dd, *J* = 9.2, 4.1 Hz, 1H), 3.06–2.93 (m, 2H), 2.20 (s, 3H), 1.89–1.79 (m, 1H), 1.77–1.68 (m, 1H), 1.49–1.34 (m, 2H, therein: 1.41 (s, 9H)), 1.22 (s, 9H) ppm.

^13^C-NMR (126 MHz, methanol-*d_4_*): δ 173.3, 172.9, 171.2, 158.53, 158.46, 145.5, 145.1, 142.6, 142.5, 140.7, 137.1, 133.7, 129.2, 128.8, 128.7, 128.2, 128.1, 127.0, 126.6, 126.3, 120.9, 120.8, 116.9, 79.8, 74.8, 68.5, 63.4, 57.5, 56.7, 48.7, 48.3, 41.1, 30.1, 28.8, 28.6, 27.8, 23.7 ppm.

(–)-HR-ESI-MS: *m/z* 821.2767 calculated for C_41_H_50_N_4_O_9_Br [M–H]^–^; found 821.2763.

IR (neat): 3313 (w), 2974 (w), 2163 (w), 2051 (w), 1981 (w), 1678 (s), 1579 (w), 1517 (s), 1450 (m), 1420 (m), 1365 (m), 1248 (m), 1167 (m), 1085 (w), 1034 (w), 871 (w), 759 (m), 740 (m), 621 (w), 545 (w), 428 (w).

${[\alpha]}_{D}^{23}$: + 56.0 (*c* = 1.00, acetone).

**Analytical data for SI-43**:

R*_f_* = 0.29 (MeOH/CH_2_Cl_2_ 5%).

^1^H-NMR (500 MHz, methanol-*d_4_*): δ 7.79 (d, *J* = 7.6 Hz, 2H), 7.67 (d, *J* = 6.4 Hz, 2H), 7.64 (d, *J* = 7.6 Hz, 1H), 7.50 (d, *J* = 8.2 Hz, 1H), 7.38 (tt, *J* = 7.5, 1.6 Hz, 2H), 7.28 (q, *J* = 7.0 Hz, 2H), 6.96 (d, *J* = 8.2 Hz, 1H), 6.49 (br. s, 1H), 4.79 (d, *J* = 5.4 Hz, 1H), 4.32 (dd, *J* = 10.4, 7.2 Hz, 1H), 4.27 (dd, *J* = 10.4, 7.3 Hz, 1H), 4.25–4.17 (m, 2H), 3.93–3.63 (m, 1H), 3.54–3.49 (m, 1H), 3.24–3.19 (m, 1H), 3.02–2.93 (m, 2H), 2.19 (s, 3H), 1.85–1.76 (m, 1H), 1.76–1.67 (m, 1H), 1.40 (s, 9H), 1.38–1.29 (m, 2H), 1.19 (s, 9H) ppm.

^13^C-NMR (126 MHz, methanol-*d_4_*): δ 174.5, 172.7, 171.4, 158.6, 158.5, 145.4, 145.1, 142.59, 142.55, 140.9, 137.1, 133.7, 129.1, 128.79, 128.78, 128.20, 128.17, 127.6, 126.5, 126.3, 120.9, 117.0, 79.9, 74.8, 68.4, 63.2, 57.5, 57.3, 48.7, 48.3, 41.1, 30.2, 28.8, 28.7, 27.7, 23.6 ppm.

(+)-HR-ESI-MS: *m/z* 844.2892 calculated for C_41_H_52_N_5_O_8_BrNa [M+Na]^+^; found 844.2905.

IR (neat): 3315 (w), 2975 (w), 2163 (w), 1674 (s), 1580 (w), 1520 (s), 1451 (w), 1420 (w), 1365 (m), 1249 (m), 1168 (m), 1085 (w), 1034 (w), 760 (w), 741 (m), 621 (w), 545 (w), 462 (w), 428 (w).

${[\alpha]}_{D}^{23}$: + 61.6 (*c* = 1.00, acetone).

To a solution of **SI-41** (413 mg, 630 µmol, 1.00 equiv.) in dry DMF (5.0 mL) was added piperidine (60 µL, 60 µmol, 0.95 equiv.). After stirring at rt for 30 min the reaction was quenched by the addition of sat. aq. NaHCO_3_ (30 mL). The aq. layer was extracted with EtOAc (3 × 50 mL). The combined organic layers were washed with aq. HCl (1 M, 50 mL), brine (50 mL), dried over MgSO_4_, filtered and the solvent was removed under reduced pressure delivering crude **32** which was used in the next step without further purification (assumed quant.).

Carboxylic acid **SI-42** (519 mg, 630 µmol, 1.00 equiv.) and crude amine **32** (assumed 272 mg, 630 µmol, 1.00 equiv.) were suspended in dry DMF (15 mL). HATU (309 mg, 813 µmol, 1.30 equiv.) and dry DIPEA (550 µL, 3.15 mmol, 5.00 equiv.) were added at 0 °C. The resulting mixture was stirred at 0 °C for 30 min and at rt for 16 h. The reaction was quenched by the addition of sat. aq. NH_4_Cl (25 mL). The aq. layer was extracted with EtOAc (3 × 50 mL). The combined organic layers were washed with brine (50 mL), dried over MgSO_4_, filtered and the solvent was removed under reduced pressure. Purification by silica gel column chromatography (MeOH/CH_2_Cl_2_ 0% to 5%) delivered product **SI-44** (686 mg, 554 µmol, 88%) as a white foam.

R*_f_* = 0.30 (MeOH/CH_2_Cl_2_ 2%).

^1^H-NMR (500 MHz, acetone-*d_6_*): δ 8.74 (s, 1H), 8.00 (s, 1H), 7.84 (d, *J* = 7.5 Hz, 1H), 7.83 (d, *J* = 7.5 Hz, 1H), 7.76 (d, *J* = 7.7 Hz, 1H), 7.74 (d, *J* = 8.3 Hz, 1H), 7.66 (d, *J* = 7.5 Hz, 1H), 7.63 (d, *J* = 7.8 Hz, 1H), 7.53 (d, *J* = 8.6 Hz, 1H), 7.43–7.11 (m, 21H), 6.87 (d, *J* = 8.3 Hz, 1H), 5.98–5.92 (m, 1H), 5.12 (s, 2H), 5.00 (d, *J* = 4.0 Hz, 1H), 4.81 (t, *J* = 6.3 Hz, 1H), 4.60–4.54 (m, 1H), 4.41 (d, *J* = 12.1 Hz, 1H), 4.37 (d, *J* = 12.0 Hz, 1H), 4.35–4.28 (m, 2H), 4.24–4.15 (m, 2H), 3.88 (dd, *J* = 8.8, 3.9 Hz, 1H), 3.66–3.61 (m, 1H), 3.58 (dd, *J* = 8.8, 4.0 Hz, 1H), 3.53–3.47 (m, 1H), 3.35–3.28 (m, 1H), 3.17–3.07 (m, 2H), 3.07–2.98 (m, 2H), 2.27 (s, 3H), 1.87 (ddt, *J* = 12.1, 9.8, 6.0 Hz, 1H), 1.72 (dtd, *J* = 14.2, 9.3, 5.3 Hz, 1H), 1.52–1.41 (m, 2H), 1.38 (s, 9H), 1.23 (s, 9H) ppm.

^13^C-NMR (126 MHz, acetone-*d_6_*): δ 171.7, 171.0, 170.5, 170.2, 169.4, 157.4, 156.6, 145.4, 144.8, 142.0, 140.9, 139.1, 137.5, 136.8, 136.5, 133.0, 130.2, 129.31, 129.28, 129.2, 129.1, 129.0, 128.7, 128.6, 128.5, 128.3, 128.02, 127.95, 127.6, 126.6, 126.3, 125.1, 120.7, 114.3, 78.4, 74.0, 73.5, 70.7, 67.6, 67.4, 63.3, 57.1, 56.2, 54.6, 53.4, 48.9, 47.9, 40.8, 30.1, 28.7, 28.6, 27.7, 24.3 ppm.

(+)-HR-ESI-MS: *m/z* 1237.4856 calculated for C_67_H_78_N_6_O_12_Br [M+H]^+^; found 1237.4844.

IR (neat): 3301 (w), 2922 (w), 1661 (s), 1579 (w), 1516 (s), 1452 (m), 1421 (w), 1390 (w), 1365 (m), 1247 (m), 1168 (m), 1081 (m), 1029 (m), 848 (w), 739 (s), 698 (m), 621 (w), 545 (w), 459 (w), 426 (w).

${[\alpha]}_{D}^{23}$: + 2.7 (*c* = 1.00, acetone).

**Synthesis of 34**

To a solution of **SI-44** (138 mg, 111 µmol, 1.00 equiv.) in dry DMF (2.5 mL) was added piperidine (11 µL, 110 µmol, 1.00 equiv.) and the resulting mixture was stirred at rt for 30 min. The reaction was quenched by the addition of sat. aq. NaHCO_3_ (10 mL) and the aq. layer was extracted with EtOAc (3 × 20 mL). The combined organic layers were washed with brine (20 mL), dried over MgSO_4_, filtered and the solvent was removed under reduced pressure. The resulting crude amine **33** was used in the next step without further purification (assumed quant.).

To a solution of carboxylic acid **23** (41.8 mg, 31.5 µmol, 1.00 equiv.) and crude amine **33** (assumed 32.0 mg, 31.5 µmol, 1.00 equiv.) in dry DMF (1.0 mL) were added HATU (14.4 mg, 37.8 µmol, 1.20 equiv.) and DIPEA (11 µL, 63 µmol, 2.00 equiv.) at 0 °C. The resulting mixture was stirred at this temperature for 30 min and at rt for 16 h. The reaction was quenched by the addition of sat. aq. NH_4_Cl (20 mL) and the aq. layer was extracted with EtOAc (3 × 50 mL). The combined organic layers were washed with brine (50 mL), dried over MgSO_4_, filtered and the solvent was removed under reduced pressure. Purification by silica gel column chromatography (MeOH/CH_2_Cl_2_ 0% to 5%) delivered product **34** (51.1 mg, 22.0 µmol, 70%) as an off-white solid.

R*_f_* = 0.40 (MeOH/CH_2_Cl_2_ 5%).

^1^H-NMR (500 MHz, methanol-*d_4_*): δ 7.78 (d, *J* = 7.6 Hz, 2H), 7.63–7.54 (m, 4H), 7.47 (s, 1H), 7.45 (dd, *J* = 8.1, 4.1 Hz, 2H), 7.35 (td, *J* = 7.6, 2.5 Hz, 2H), 7.33–7.13 (m, 35H), 7.10–7.04 (m, 4H), 7.00 (d, *J* = 8.0 Hz, 2H), 6.94 (d, *J* = 7.6 Hz, 1H), 6.87 (d, *J* = 8.4 Hz, 1H), 5.84 (br. s, 1H), 5.25–5.15 (m, 1H), 5.07 (s, 2H), 5.01 (s, 2H), 4.78–4.70 (m, 2H), 4.65 (dd, *J* = 9.4, 3.7 Hz, 1H), 4.43 (dd, *J* = 9.3, 5.0 Hz, 2H), 4.36–4.26 (m, 4H), 4.17–4.13 (m, 1H), 4.08 (d, *J* = 9.0 Hz, 1H), 3.63 (s, 3H), 3.55–3.46 (m, 1H), 3.42 (d, *J* = 5.2 Hz, 2H), 3.38 (dd, *J* = 9.4, 5.7 Hz, 1H), 3.18–3.00 (m, 5H), 2.98–2.90 (m, 3H), 2.84 (dd, *J* = 14.8, 9.5 Hz, 1H), 2.14 (s, 3H), 2.10 (s, 3H), 1.80–1.69 (m, 1H), 1.70–1.59 (m, 1H), 1.38 (s, 9H), 1.32–1.28 (m, 2H), 0.95 (s, 9H), 0.82 (t, *J* = 7.9 Hz, 9H), 0.48 (q, *J* = 7.9 Hz, 6H) ppm.

^13^C-NMR (126 MHz, methanol-*d_4_*): δ 174.4, 173.6, 172.4, 171.9, 171.8, 171.5, 171.3, 170.2, 158.6, 158.4, 146.5, 146.3, 145.8, 144.7, 142.6, 142.4, 141.1, 139.1, 138.0, 137.7, 137.0, 136.1, 135.6, 133.9, 130.9, 130.6, 130.4, 130.0, 129.60, 129.57, 129.54, 129.51, 129.42, 129.37, 129.0, 128.88, 128.85, 128.8, 128.72, 128.70, 128.4, 128.3, 128.2, 128.0, 127.9, 126.9, 126.7, 126.3, 126.0, 121.0, 120.9, 114.1, 112.6, 102.3, 92.3, 79.8, 74.8, 74.1, 71.9, 70.6, 70.1, 69.1, 68.1, 67.7, 62.5, 58.5, 57.8, 55.9, 55.4, 55.1, 54.8, 54.4, 52.9, 48.0, 41.1, 39.9, 38.5, 30.8, 30.3, 28.8, 28.6, 27.8, 23.7, 21.5, 7.9, 5.0 ppm.

(+)-HR-ESI-MS: *m/z* 2344.8740 calculated for C_128_H_140_N_11_O_22_BrSSiNa [M+Na]^+^; found 2344.8768.

IR (CHCl_3_): 3346 (w), 3030 (w), 2955 (w), 2874 (w), 1673 (s), 1577 (w), 1495 (m), 1450 (m), 1421 (m), 1365 (m), 1251 (m), 1214 (m), 1190 (m), 1175 (m), 1090 (w), 1043 (w), 740 (m), 699 (m), 668 (w), 571 (w), 542 (w), 459 (w), 415 (w), 404 (w).

${[\alpha]}_{D}^{23}$: + 22.9 (*c* = 1.00, CHCl_3_).

**Synthesis of 35 & 36**

Pd(P*t*Bu_3_)_2_ (5.7 mg, 11.2 µmol, 1.1 equiv.) was suspended in dry and degassed MeCN (5 mL) in a Schlenk tube under N_2_. **34** (23.8 mg, 10.2 µmol, 1.0 equiv.) was separately dissolved in dry and degassed MeCN (5 mL) under N_2_. Dry Et_3_N (8 µL, 56.9 µmol, 5.6 equiv.) was added to **34**, and this mixture was finally transferred to the Schlenk tube with the stirring mixture of catalyst. The resulting mixture was stirred for 1 h at 80 °C. After this time the mixture was cooled down to rt, filtered and washed with DCM. The filtrate was concentrated under reduced pressure and the crude was purified by preparative HPLC (MeCN/H_2_O/FA 80:20:0.1 to 100:0:0.1) to afford **35** (2.3 mg, 1.0 µmol, 10 %) and **36** (10.2 mg, 4.5 µmol, 45 %) as yellow oils. *(Note: a mixture of the desired product* ***35*** *and desilylated product was obtained. These two compounds were not separable by silica gel column chromatography nor prep-HPLC and therefore only HRMS is described for these compounds. 1D and 2D NMR spectra of the mixture are available in the NMR spectra section.)*

**Analytical data for 35 (mixture between TES protected and deprotected compounds)**

R*_f_* = 0.36 (MeOH/CH_2_Cl_2_ 3%)

(+)-HR-ESI-MS: *m/z* 1121.9866 calculated for C_128_H_141_N_11_O_22_SSi [M+2H]^2+^; found 1121.9873 (TES protected compound)

(+)-HR-ESI-MS: *m/z* 1064.9433 calculated for C_122_H_127_N_11_O_22_S [M+2H]^2+^; found 1064.9448 (deprotected compound)

**Analytical data for 36**

R*_f_* = 0.38 (MeOH/CH_2_Cl_2_ 3%)

^1^H-NMR (500 MHz, CD_2_Cl_2_) *δ:* 7.95 (d, *J* = 7.9 Hz, 2H), 7.84–7.73 (m, 3H), 7.68 (br s, 1H), 7.62–7.54 (m, 3H), 7.47–7.16 (m, 38H), 7.10 (br s, 1H), 7.08–6.98 (m, 5H), 6.98–6.92 (m, 1H), 6.92–6.83 (m, 2H), 6.78 (d, *J* = 7.4 Hz, 1H), 6.38 (d, *J* = 6.9 Hz, 1H), 6.12 (d, *J* = 3.7 Hz, 1H), 6.06 (d, *J* = 2.8 Hz, 1H), 5.48–5.37 (m, 2H), 5.28 (d, *J* = 10.6 Hz, 1H), 5.24–5.19 (m, 2H), 5.12–5.02 (m, 2H), 4.97–4.89 (m,1H), 4.78 (brs, 1H), 4.72–4.59 (m, 3H), 4.56 (td, *J* = 7.2, 4.0 Hz, 1H), 4.53–4.44 (m, 2H), 4.40 (dd, *J* = 10.5, 7.0 Hz, 1H), 4.34–4.22 (m, 1H), 4.22–4.09 (m, 1H), 3.81 (dd, *J* = 9.3, 3.7 Hz, 1H), 3.67 (s, 3H), 3.45 (t, *J* = 7.9 Hz, 1H), 3.27–2.93 (m, 12H), 2.86–2.77 (m, 1H), 2.75 (s, 3H), 2.26 (s, 3H), 1.94–1.77 (m, 2H), 1.54–1.43 (m, 2H), 1.37 (s, 9H), 1.12–0.82 (m, 24H) ppm

^13^C-NMR (126 MHz, CD_2_Cl_2_) *δ*: 171.7, 171.0, 170.7, 170.2, 169.7, 169.3, 169.1, 169.0, 167.1, 155.7, 155.5, 145.3, 144.9, 144.5, 144.2, 143.7, 141.2, 141.1, 137.6, 137.3, 137.2, 136.5, 135.7, 135.3, 135.0, 134.6, 130.2, 129.4, 129.1, 128.7, 128.6, 128.5, 128.5, 128.5, 128.1, 127.9, 127.8, 127.6, 127.6, 127.1, 126.9, 125.2, 125.0, 124.0, 123.2, 121.9, 119.8, 117.1, 116.7, 112.5, 111.1, 78.6, 73.4, 73.2, 70.6, 69.1, 67.5, 67.3, 66.8, 61.4, 61.3, 58.3, 54.6, 54.0, 53.5, 52.6, 52.3, 52.1, 49.5, 47.1, 40.0, 38.5, 37.6, 29.5, 28.5, 28.1, 27.7, 27.0, 26.3, 21.4, 8.2, 6.4 ppm.

IR (neat): 3356 (br), 2954 (br), 1692 (s), 1493 (s), 1451 (m), 1366 (m), 1314 (m), 1215 (m), 1189 (m), 1174 (m), 1117 (m), 1092 (m), 1027 (w), 812 (w), 741 (m), 699 (m), 668 (m), 573 (w).

${[\alpha]}_{D}^{22}$: + 113.3 (*c* = 1.00, CHCl_3_)

(+)-HR-ESI-MS: *m/z* 1121.9866 calculated for C_128_H_141_N_11_O_22_SSi [M+2H]^2+^; found 1121.9869 (TES protected compound)

**Synthesis of 41**

Propargylic alcohol **12** (2.38 g, 6.44 mmol, 1.00 equiv.) was dissolved in dry THF (50 mL) and cooled to −40 °C. NaHMDS (2 M in THF, 4.85 mL, 9.66 mmol, 1.50 equiv.) was added and the resulting mixture was stirred at this temperature for 1 h. Iodomethane (600 mL, 9.66 mmol, 1.50 equiv.) was added and the mixture was allowed to slowly warm up to rt and stirred for 1.5 h. The reaction was quenched by the addition of water (250 mL). The aq. layer was extracted with EtOAc (3 × 500 mL). The combined organic layers were washed with brine (500 mL), dried over MgSO_4_, filtered and the solvent removed under reduced pressure. The crude mixture was purified by silica gel flash column chromatography (EtOAc/hexane 0% to 5%) to afford **SI-45** (1.93 g, 5.03 mmol, 78%) as a colorless oil.

R*_f_* = 0.38 (EtOAc/hexanes 1:10).

^1^H-NMR (500 MHz, DMSO-*d_6_*, 80 °C): δ 4.48 (br. s, 1H), 4.11–3.83 (m, 3H), 3.36 (s, 3H), 1.55 (s, 3H), 1.44 (s, 9H), 1.43 (s, 3H), 0.98 (t, *J* = 7.9 Hz, 9H), 0.59 (q, *J* = 7.9 Hz, 6H) ppm.

^13^C-NMR (126 MHz, DMSO-*d_6_*, 80 °C): δ 151.1, 102.9, 93.4, 89.1, 79.3, 71.4, 63.8, 58.3, 56.1, 27.6, 25.7, 6.7, 3.5 ppm.

(+)-HR-ESI-MS: *m/z* 384.2565 calculated for C_20_H_38_NO_4_Si [M+H]^+^; found 384.2563.

IR (neat): 2955 (w), 2876 (w), 1695 (s), 1458 (w), 1387 (s), 1365 (s), 1258 (m), 1172 (m), 1118 (m), 1097 (s), 1061 (m), 1018 (w), 977 (w), 855 (w), 802 (w), 738 (m).

${[\alpha]}_{D}^{23}$: + 76.0 (*c* = 0.80, CHCl_3_).

Methyl ether **SI-45** (1.93 g, 5.03 mmol, 1.00 equiv.) was dissolved in CH_2_Cl_2_ (50 mL) and TFA (10 mL) and the resulting mixture was stirred at rt for 30 min. The mixture was then diluted with water (100 mL) and the aq. layer was extracted with CH_2_Cl_2_ (3 × 150 mL). The combined organic layers were washed with brine (150 mL), dried over MgSO_4_, filtered and the solvent removed under reduced pressure. The resulting crude **SI-46** was used in the next step without further purification (assumed quant.).

Amine **SI-46** (assumed 1.22 g, 5.03 mmol, 1.00 equiv.) was dissolved in a mixture of water/dioxane (1:1, 30 mL) and NaHCO_3_ (1.06 g, 12.6 mmol, 2.50 equiv.) was added at rt. The resulting mixture was cooled to 0 °C and a solution of Fmoc-OSu (1.70 g, 5.03 mmol, 1.00 equiv.) in dioxane (10 mL) was added dropwise. The mixture was then allowed to warm up to rt and stirred for 64 h. The reaction was quenched by the addition of sat. aq. NH_4_Cl (100 mL). The aq. layer was extracted with EtOAc (3 × 250 mL). The combined organic layers were washed with an aq. solution of HCl (1 M, 250 mL) and brine (250 mL), dried over MgSO_4_, filtered and the solvent removed under reduced pressure. Purification by silica gel flash column chromatography (EtOAc/hexane 12% to 30%) delivered product **SI-47** (2.28 g, 4.90 mmol, 97%) as a viscous, colorless oil.

R*_f_* = 0.70 (EtOAc/hexane 1:1).

^1^H-NMR (500 MHz, DMSO-*d_6_*): δ 7.86 (d, *J* = 7.5 Hz, 2H), 7.70 (dd, *J* = 7.6, 4.7 Hz, 2H), 7.42 (t, *J* = 7.4 Hz, 2H), 7.32 (t, *J* = 7.4 Hz, 2H), 4.39–4.19 (m, 3H), 4.26 (d, *J* = 5.6 Hz, 1H), 3.71 (p, *J* = 6.0 Hz, 1H), 3.59 (dd, *J* = 10.9, 5.4 Hz, 1H), 3.52 (dd, *J* = 10.9, 6.5 Hz, 1H), 3.33 (s, 3H), 0.97 (t, *J* = 7.8 Hz, 9H), 0.58 (q, *J* = 7.8 Hz, 6H) ppm.

^13^C-NMR (126 MHz, DMSO-*d_6_*): δ 155.5, 143.6, 143.5, 140.4, 127.2, 126.6, 124.8, 124.7, 119.6, 103.8, 88.3, 70.2, 65.4, 59.9, 56.0, 55.5, 46.5, 6.7, 3.6 ppm.

(+)-HR-ESI-MS: *m/z* 466.2408 calculated for C_27_H_36_NO_4_Si [M+H]^+^; found 466.2410.

IR (neat): 3432 (w), 2954 (w), 2875 (w), 1706 (s), 1511 (m), 1450 (m), 1414 (w), 1329 (w), 1236 (m), 1158 (w), 1087 (m), 1019 (m), 758 (m), 739 (s), 621 (w).

${[\alpha]}_{D}^{23}$: – 2.4 (*c* = 1.00, CHCl_3_).

To a solution of alcohol **SI-47** (2.28 g, 4.90 mmol, 1.00 equiv.) in a mixture of MeCN (48 mL) and aq. phosphate buffer (pH = 6.4, 10 mM, 24 mL) were added TEMPO (153 mg, 0.98 mmol, 0.20 equiv.), and PIDA (158 mg, 0.49 mmol, 0.10 equiv.) at rt. The resulting mixture was cooled to 0 °C and NaClO_2_ (1.55 g, 17.2 mmol, 3.50 equiv.) was added. The reaction mixture was allowed to slowly warm up to rt and stirred for 3 h. The reaction was quenched by the addition of sat. aq. NH_4_Cl (120 mL). The aq. layer was extracted with EtOAc (3 × 200 mL). the combined organic layers were washed with brine (200 mL), dried over MgSO_4_, filtered and the solvent removed under reduced pressure. The crude mixture was purified by silica gel column chromatography (EtOAc/hexane 50% + 0.2% AcOH) and the product containing fractions were co-evaporated with toluene to remove residual AcOH. The product **41** (1.99 g, 4.14 mmol, 85%) was obtained as a colorless oil.

R*_f_* = 0.20 (EtOAc/hexane 1:1 + 0.2% AcOH).

^1^H-NMR (400 MHz, methanol-*d_4_*): δ 7.81 (d, *J* = 7.5 Hz, 2H), 7.70 (d, *J* = 7.5 Hz, 2H), 7.40 (td, *J* = 7.5, 0.7 Hz, 2H), 7.31 (tt, *J* = 7.4, 1.4 Hz, 2H), 4.60 (d, *J* = 3.3 Hz, 1H), 4.58–4.52 (m, 1H), 4.34–4.28 (m, 2H), 4.25 (dd, *J* = 8.4, 6.0 Hz, 1H), 3.43 (s, 3H), 0.95 (t, *J* = 7.9 Hz, 9H), 0.56 (q, *J* = 7.9 Hz, 6H) ppm.

^13^C-NMR (126 MHz, methanol-*d_4_*): δ 172.5, 158.8, 145.3, 145.2, 142.6, 142.5, 128.8, 128.2, 126.43, 126.41, 120.9, 103.2, 90.8, 73.0, 68.6, 59.5, 57.3, 48.3, 7.8, 5.1 ppm.

(+)-HR-ESI-MS: *m/z* 480.2201 calculated for C_27_H_34_NO_5_Si [M+H]^+^; found 480.2206.

IR (neat): 2955 (m), 1728 (s), 1514 (m), 1450 (w), 1374 (w), 1328 (w), 1240 (m), 1098 (m), 1058 (s), 974 (w), 758 (m), 737 (s), 621 (w), 539 (w), 427 (w).

${[\alpha]}_{D}^{23}$: – 29.2 (*c* = 1.00, MeOH).

**Synthesis of 40**

Methyl ether **41** (498 mg, 1.04 mmol, 1.00 equiv.) was dissolved in dry CH_2_Cl_2_ (50 mL) and cooled to –70 °C. BBr_3_ (500 µL, 5.20 mmol, 5.00 equiv.) was added dropwise and the resulting mixture was slowly allowed to warm up to –40 °C and stirred for 3 h. The reaction was quenched by the addition of sat. aq. NaHCO_3_ (20 mL) at 0 °C. The aq. layer was extracted with CH_2_Cl_2_ (2 × 50 mL). The combined organic layers were washed with an aq. soln. of H_2_SO_4_ (0.1 M, 50 mL) and brine (50 mL), dried over MgSO_4_, filtered and the solvent was removed under reduced pressure. Purification by prep-HPLC (MeCN/H_2_O 50% to 100% + 0.1% FA, 45 min) delivered product **40** (254 mg, 0.546 mmol, 53%) as a white powder.

^1^H-NMR (500 MHz, methanol-*d_4_*): δ 7.81 (d, *J* = 7.5 Hz, 2H), 7.69 (d, *J* = 7.5 Hz, 2H), 7.40 (t, *J* = 7.5 Hz, 2H), 7.32 (t, *J* = 7.6 Hz, 2H), 4.90 (d, *J* = 3.3 Hz, 1H), 4.47 (d, *J* = 3.2 Hz, 1H), 4.38–4.29 (m, 2H), 4.25 (t, *J* = 7.1 Hz, 1H), 0.96 (t, *J* = 7.9 Hz, 9H), 0.56 (q, *J* = 7.9 Hz, 6H) ppm.

^13^C-NMR (126 MHz, methanol-*d_4_*): δ 172.5, 158.7, 145.3, 145.2, 142.58, 142.57, 128.8, 128.2, 126.34, 126.33, 120.9, 106.2, 88.3, 68.5, 63.9, 60.7, 48.3, 7.7, 5.1 ppm.

(–)-HR-ESI-MS: *m/z* 464.1899 calculated for C_26_H_30_NO_5_Si [M–H]^–^; found 464.1896.

IR (neat): 2954 (w), 2874 (w), 1705 (m), 1516 (w), 1450 (w), 1412 (w), 1330 (w), 1234 (m), 1166 (w), 1056 (m), 1033 (m), 1019 (m), 1007 (m), 976 (w), 757 (m), 726 (s), 681 (w), 621 (w), 596 (w), 537 (w).

${[\alpha]}_{D}^{23}$: – 3.9 (*c* = 1.00, MeOH).

M.p.: 60.3–61.3 °C.

**Synthesis of 42**

Silylated alkyne **41** (2.30 g, 4.80 mmol, 1.00 equiv.) was dissolved in dry THF (20 mL) and cooled to 0 °C. A solution of TBAF (1 M in THF, 9.60 mL, 9.60 mmol, 2.00 equiv.) was added dropwise and the resulting mixture was slowly allowed to warm up to rt and stirred for 2 h. The reaction was quenched by the addition of sat. aq. NH_4_Cl (50 mL) and the aq. layer was extracted with EtOAc (3 × 100 mL). The combined organic layers were washed with brine (100 mL), dried over MgSO_4_, filtered and the solvent was removed under reduced pressure. Purification by silica gel column chromatography (EtOAc/hexane 9% to 50% + 0.2% AcOH) followed by co-evaporation with toluene to remove residual AcOH delivered product **SI-48** (1.47 g, 4.02 mmol, 84%) as a white solid.

R*_f_* = 0.24 (EtOAc/hexane 1:1 + 0.2% AcOH).

^1^H-NMR (500 MHz, methanol-*d_4_*): δ 7.80 (d, *J* = 7.5 Hz, 2H), 7.69 (d, *J* = 7.5 Hz, 2H), 7.39 (t, *J*= 7.5 Hz, 2H), 7.31 (t, *J* = 7.4 Hz, 2H), 4.60–4.58 (m, 1H), 4.54 (d, *J* = 3.4 Hz, 1H), 4.42–4.22 (m, 3H), 3.43 (s, 3H), 2.96 (d, *J* = 2.0 Hz, 1H) ppm.

^13^C-NMR (101 MHz, methanol-*d_4_*): δ 172.2, 158.9, 145.2, 142.5, 128.8, 128.2, 126.4, 120.9, 79.7, 77.5, 72.4, 68.4, 59.3, 57.4, 48.3 ppm.

(+)-HR-ESI-MS: *m/z* 388.1155 calculated for C_21_H_19_NO_5_Na [M+Na]^+^; found 388.1157.

IR (neat): 3404 (w), 2971 (w), 1755 (w), 1718 (s), 1536 (m), 1450 (w), 1391 (w), 1325 (w), 1302 (w), 1237 (m), 1207 (m), 1099 (m), 1082 (s), 1044 (m), 1028 (m), 848 (w), 770 (w), 754 (m), 739 (s), 727 (m), 684 (m), 621 (w), 594 (w), 538 (m), 426 (w).

${[\alpha]}_{D}^{23}$: – 8.1 (*c* = 0.92, CHCl_3_).

M.p.: 130.5–131.4 °C.

Methyl ether **SI-48** (1.44 g, 3.94 mmol, 1.00 equiv.) was dissolved in dry CH_2_Cl_2_ (60 mL) and cooled to –70 °C. BBr_3_ (1.87 mL, 19.7 mmol, 5.00 equiv.) was added dropwise and the resulting mixture was slowly allowed to warm up to –40 °C and stirred for 1.5 h. The reaction was quenched by the addition of CH_2_Cl_2_ (50 mL) and water (0.25 mL) at 0 °C. The mixture was dried over MgSO_4_, filtered and the solvent was removed under removed pressure. Purification by silica gel column chromatography (EtOAc/hexane 10% to 100% + 0.2% AcOH) followed by co-evaporation with toluene to remove residual AcOH deliver product **42** (503 mg, 1.43 mmol, 36%) as a white foam.

R*_f_* = 0.16 (EtOAc/hexane 1:1 + 0.2% AcOH).

^1^H-NMR (400 MHz, methanol-*d_4_*): δ 7.78 (d, *J* = 7.5 Hz, 2H), 7.68 (d, *J* = 7.5 Hz, 2H), 7.38 (t, *J* = 7.4 Hz, 2H), 7.30 (t, *J* = 7.5 Hz, 2H), 4.89–4.82 (m, 1H), 4.46 (d, *J* = 3.4 Hz, 1H), 4.41–4.29 (m, 2H), 4.24 (t, *J* = 7.1 Hz, 1H), 2.87 (td, *J* = 8.3, 2.0 Hz, 1H) ppm.

^13^C-NMR (126 MHz, methanol-*d_4_*): δ 172.5, 158.8, 145.21, 145.17, 142.5, 128.8, 128.2, 126.34, 126.29, 120.9, 82.8, 75.3, 68.3, 63.3, 60.5, 48.3 ppm.

(+)-HR-ESI-MS: *m/z* 374.0999 calculated for C_20_H_17_NO_5_Na [M+Na]^+^; found 374.0999.

IR (neat): 3285 (w), 2512 (w), 1693 (s), 1524 (w), 1477 (w), 1450 (m), 1432 (m), 1338 (m), 1293 (m), 1249 (m), 1229 (m), 1189 (w), 1104 (w), 1057 (m), 972 (w), 758 (m), 739 (s), 650 (m), 568 (w), 536 (w), 425 (w).

${[\alpha]}_{D}^{23}$: – 9.7 (*c* = 1.00, MeOH).

**Synthesis of 43 & 44**

To a solution of carboxylic acid **40** (22.4 mg, 48.1 µmol, 1.50 equiv.) and crude amine **33** (assumed 32.7 mg, 32.2 µmol, 1.00 equiv.) in dry DMF (1.0 mL) were added HATU (24.4 mg, 64.2 µmol, 2.00 equiv.) and DIPEA (27 µL, 160 µmol, 5.00 equiv.) at 0 °C. The resulting mixture was stirred at rt for 1 d. The reaction was quenched by the addition of sat. aq. NH_4_Cl (25 mL) and the aq. layer was extracted with EtOAc (3 × 50 mL). The combined organic layers were washed with brine (50 mL), dried over MgSO_4_, filtered and the solvent was removed under reduced pressure. Purification by silica gel column chromatography (MeOH/CH_2_Cl_2_ 0% to 3%) delivered product **SI-49** (31.4 mg, 21.5 µmol, 67%) as an orange gum.

R*_f_* = 0.10 (MeOH/CH_2_Cl_2_ 3%).

^1^H-NMR (500 MHz, methanol-*d_4_*): δ 7.80 (d, *J* = 7.6 Hz, 2H), 7.67 (dd, *J* = 7.6, 3.2 Hz, 2H), 7.62 (br. s, 1H), 7.39 (t, *J* = 7.4 Hz, 2H), 7.17–7.18 (m, 17H), 7.12 (dd, *J* = 7.5, 1.9 Hz, 2H), 6.84 (d, *J* = 8.5 Hz, 1H), 5.06 (q, *J* = 12.1 Hz, 2H), 4.66 (d, *J* = 2.7 Hz, 1H), 4.58 (br. s, 1H), 4.56 (t, *J* = 5.5 Hz, 1H), 4.48 (d, *J* = 11.8 Hz, 1H), 4.42 (d, *J* = 12.0 Hz, 1H), 4.31–4.28 (m, 1H), 4.20 (t, *J* = 7.2 Hz, 1H), 3.78 (dd, *J* = 9.1, 4.0 Hz, 1H), 3.64 (dd, *J* = 9.9, 5.7 Hz, 1H), 3.58 (dd, *J* = 9.8, 5.1 Hz, 1H), 3.53 (dd, *J* = 9.1, 3.9 Hz, 1H), 3.19 (dd, *J* = 9.7, 4.6 Hz, 1H), 3.14 (dd, *J* = 13.7, 6.2 Hz, 1H), 3.06 (dd, *J* = 13.8, 7.2 Hz, 1H), 3.00–2.91 (m, 2H), 2.22 (s, 3H), 1.83–1.74 (m, 1H), 1.73–1.66 (m, 1H), 1.40 (s, 9H), 1.36–1.30 (m, 2H), 1.18 (s, 9H), 0.93 (t, *J* = 7.9 Hz, 9H), 0.53 (q, *J* = 7.9 Hz, 6H) ppm.

^13^C-NMR (126 MHz, methanol-*d_4_*): δ 172.4, 172.0, 171.9, 171.53, 171.45, 158.6, 145.2, 142.6, 139.1, 137.7, 136.9, 133.8, 130.4, 129.64, 129.55, 129.44, 129.38, 129.0, 128.83, 128.78, 128.2, 128.0, 126.4, 121.0, 117.3, 79.8, 74.9, 74.2, 70.8, 68.7, 68.1, 64.5, 62.8, 60.7, 55.4, 54.4, 48.3, 38.9, 38.5, 30.0, 28.8, 27.8, 23.7, 7.9, 5.2 ppm.^[[6]](#footnote-6)^

(+)-HR-ESI-MS: *m/z* 1484.5860 calculated for C_78_H_96_N_7_O_14_BrSiNa [M+Na]^+^; found 1484.5846.

IR (CHCl_3_): 3313 (w), 3064 (w), 2954 (w), 2874 (w), 1674 (s), 1579 (w), 1521 (s), 1453 (m), 1421 (w), 1392 (w), 1366 (m), 1249 (m), 1174 (m), 1102 (w), 1030 (w), 757 (w), 740 (m), 699 (m), 621 (w), 465 (w), 425 (w), 409 (w).

${[\alpha]}_{D}^{23}$: + 12.5 (*c* = 0.20, CHCl_3_).

Pd(*t*Bu_3_P)_2_ (49.6 mg, 97.1 µmol, 3.53 equiv.) – issued from a glove box – was suspended in dry and degassed dioxane (4.0 mL) in a Schlenk tube. Compound **SI-49** (40.3 mg, 27.5 µmol, 1.00 equiv.) was separately dissolved in dry and degassed dioxane (6.0 mL) and treated with NEt_3_ (67 µL, 480 µmol, 17.5 equiv.). This mixture was then transferred to the Schlenk tube. This final mixture was heated to 105 °C for 30 min. After cooling down to rt the solvent was removed under reduced pressure. Purification by silica gel column chromatography (MeOH/CH_2_Cl_2_ 1% to 2%) delivered a mixture of product **43** and desilylated product **44** (combined: 13.3 mg, 9.6 µmol, 35%). *(Note:* ***43*** *and* ***44*** *were not separable by silica gel column chromatography nor prep-HPLC and therefore no analytics are shown here for these compounds.)*

**Synthesis of 44**

To a solution of amine **33** (assumed: 113 mg, 111 µmol, 1.00 equiv.) and carboxylic acid **42** (58.5 mg, 167 µmol, 1.50 equiv.) in dry DMF (5.0 mL) were added HATU (84.4 mg, 222 µmol, 2.00 equiv.) and DIPEA (97 µL, 560 µmol, 5.00 equiv.) at 0 °C. The resulting mixture was stirred at rt for 16 h and the reaction was quenched by the addition of sat. aq. NH_4_Cl (50 mL). The aq. layer was extracted with EtOAc (3 × 50 mL). The combined organic layers were washed with brine (50 mL), dried over MgSO_4_, filtered and the solvent was removed under reduced pressure. Purification by silica gel column chromatography (MeOH/CH_2_Cl_2_ 0% to 5%) delivered product **SI-50** (140 mg, 104 µmol, 93%) as an orange gum.

R*_f_* = 0.40 (MeOH/ CH_2_Cl_2_ 5%).

^1^H-NMR (500 MHz, methanol-*d_4_*): δ 7.78 (d, *J* = 7.6 Hz, 2H), 7.65 (d, *J* = 6.4 Hz, 2H), 7.61 (s, 1H), 7.46–7.33 (m, 3H), 7.33–7.16 (m, 15H), 7.14–7.08 (m, 2H), 6.86 (d, *J* = 8.4 Hz, 1H), 6.44 (t, *J* = 5.8 Hz, 1H), 5.15–5.01 (m, 2H), 4.87 (br. s, 1H), 4.80 (t, *J* = 6.8 Hz, 1H), 4.72 (dd, *J* = 4.0, 2.2 Hz, 1H), 4.58 (d, *J* = 3.9 Hz, 1H), 4.56 (t, *J* = 5.6 Hz, 1H), 4.48 (d, *J* = 4.7 Hz, 1H), 4.44 (d, *J* = 10.9 Hz, 2H), 4.37 (dd, *J* = 10.4, 7.2 Hz, 1H), 4.33–4.25 (m, 1H), 4.21 (t, *J* = 7.2 Hz, 1H), 3.74 (dd, *J* = 9.1, 4.0 Hz, 1H), 3.64 (dd, *J* = 10.0, 5.8 Hz, 1H), 3.59 (dd, *J* = 9.9, 5.6 Hz, 1H), 3.50 (dd, *J* = 9.1, 3.9 Hz, 1H), 3.18 (dt, *J* = 10.5, 5.4 Hz, 1H), 3.13 (dd, *J* = 13.8, 6.4 Hz, 1H), 3.06 (dd, *J* = 13.8, 7.2 Hz, 1H), 2.99–2.90 (m, 2H), 2.70 (d, *J* = 2.2 Hz, 1H), 2.21 (s, 3H), 1.81–1.74 (m, 1H), 1.72–1.63 (m, 1H), 1.39 (s, 9H),
1.36–1.28 (m, 2H), 1.16 (s, 9H) ppm.

^13^C-NMR (126 MHz, methanol-*d_4_*): δ 172.4, 172.0, 171.9, 171.52, 171.49, 158.5, 158.4, 145.24, 145.17, 142.6, 140.5, 139.1, 137.7, 136.9, 133.9, 130.4, 129.62, 129.56, 129.54, 129.43, 129.38, 129.0, 128.81, 128.77, 128.2, 128.0, 127.9, 127.5, 126.4, 126.3, 120.9, 82.6, 79.9, 75.9, 74.8, 74.2, 70.8, 68.4, 68.1, 63.6, 62.7, 60.4, 57.5, 55.8, 55.4, 54.4, 48.9, 48.3, 41.1, 38.4, 30.1, 28.8, 28.5, 27.8, 23.6 ppm.

(+)-HR-ESI-MS: *m/z* 1370.4995 calculated for C_72_H_82_N_7_O_14_BrNa [M+Na]^+^; found 1370.4994.

IR (neat): 3301 (m), 3064 (w), 2973 (w), 2930 (w), 1669 (s), 1580 (m), 1521 (s), 1453 (m), 1421 (w), 1391 (m), 1365 (m), 1250 (m), 1174 (m), 1102 (m), 846 (w), 759 (w), 741 (m), 699 (m), 621 (w).

${[\alpha]}_{D}^{23}$: – 8.0 (*c* = 0.50, MeOH).

Pd(*t*Bu_3_P)_2_ (21.0 mg, 41.1 µmol, 3.00 equiv.) – issued from a glove box – was suspended in dry and degassed dioxane (1.0 mL) in a Schlenk tube. Compound **SI-50** (18.5 mg, 13.7 µmol, 1.00 equiv.) was separately dissolved in dry and degassed dioxane (4.0 mL) and treated with NEt_3_ (29 µL, 210 µmol, 15.0 equiv.). This mixture was then transferred to the Schlenk tube. This final mixture was heated to 105 °C for 30 min. After cooling down to rt the solvent was removed under reduced pressure. Purification by silica gel column chromatography (MeOH/CH_2_Cl_2_ 1% to 2%) and prep-HPLC (MeCN/H_2_O 50% to 80% + 0.1% FA, 25 min) delivered **44** (3.9 mg, 3.1 µmol, 22%) as a grey powder. (*Note: this is an exemplary procedure, yields ranged from 10 to 40%)*

R*_f_* = 0.45 (MeOH/ CH_2_Cl_2_ 10%).

^1^H-NMR (500 MHz, methanol-*d_4_*): δ 8.07 (d, *J* = 8.1 Hz, 1H), 7.80 (d, *J* = 7.6 Hz, 2H), 7.65 (d, *J* = 7.6 Hz, 2H), 7.44–7.35 (m, 5H), 7.35–7.24 (m, 11H), 7.24–7.20 (m, 3H), 7.18–7.13 (m, 2H), 7.10 (br. s, 1H), 6.77 (d, *J* = 7.7 Hz, 1H), 6.34 (s, 1H), 6.00 (d, *J* = 2.1 Hz, 1H), 5.98 (d, *J* = 6.0 Hz, 1H), 5.80 (d, *J* = 8.1 Hz, 1H), 5.11 (d, *J* = 2.9 Hz, 2H), 4.76–4.65 (m, 1H), 4.62–4.57 (m, 1H), 4.47 (d, *J* = 11.9 Hz, 1H), 4.42 (d, *J* = 11.9 Hz, 1H), 4.38 (dd, *J* = 10.5, 7.2 Hz, 1H), 4.31 (dd, *J* = 10.4, 7.0 Hz, 1H), 4.27–4.18 (m, 2H), 4.14–4.10 (m, 1H), 4.07 (ddd, *J* = 12.5, 4.6, 2.3 Hz, 1H), 3.69–3.64 (m, 1H), 3.63–3.56 (m, 1H), 3.14 (dd, *J* = 5.2, 2.8 Hz, 1H), 3.12–3.04 (m, 3H), 3.01 (t, *J* = 8.7 Hz, 1H), 2.13 (s, 3H), 2.00–1.94 (m, 1H), 1.93–1.87 (m, 1H), 1.63–1.50 (m, 2H), 1.39 (s, 9H), 0.87 (s, 9H) ppm.

^13^C-NMR (126 MHz, methanol-*d_4_*): δ 172.5, 171.8, 171.7, 171.5, 167.2, 166.2, 163.2, 163.0, 145.2, 142.8, 142.6, 139.1, 137.7, 136.9, 135.6, 130.9, 130.8, 130.6, 130.5, 129.6, 129.4, 128.9, 128.8, 128.6, 128.2, 128.0, 126.3, 125.2, 124.2, 121.9, 121.3, 121.0, 119.4, 117.1, 107.4, 103.1, 89.7, 75.7, 74.6, 74.3, 71.8, 68.7, 68.2, 65.5, 55.3, 40.4, 38.5, 36.5, 31.7, 30.8, 30.6, 30.5, 30.4, 30.3, 30.24, 30.15, 28.8, 28.1, 27.5, 26.9, 25.1, 24.0, 21.0, 14.4, 11.4 ppm.

(+)-HR-ESI-MS: *m/z* 1290.5734 calculated for C_72_H_81_N_7_O_14_Na [M+Na]^+^; found 1290.5723.

IR (neat): 3355 (w), 2928 (w), 1678 (s), 1630 (m), 1490 (m), 1448 (m), 1365 (w), 1205 (s), 1189 (s), 1175 (s), 1135 (m), 1089 (m), 1039 (m), 839 (w), 801 (w), 758 (w), 724 (m), 698 (m), 668 (m), 621 (w), 571 (m), 539 (w), 427 (w).

${[\alpha]}_{D}^{23}$: + 6.4 (*c* = 1.00, MeOH).

**Synthesis of 39**

To a solution of TES-acetylene (**SI-51**, 2.87 mL, 16.0 mmol, 1.00 equiv.) in dry acetone (50 mL) were added AgNO_3_ (272 mg, 1.60 mmol, 0.10 equiv.) and NBS (3.13 g, 17.6 mmol, 1.10 equiv.) successively, each in a single portion at rt. The resulting mixture was stirred for 4 h and then quenched by the addition of ice water (100 mL). The aq. layer was extracted with pentane (3 × 100 mL). The combined organic layers were washed with brine (100 mL), dried over MgSO_4_, filtered and the solvent removed under reduced pressure. The resulting crude liquid was loaded on a small silica gel column and eluted with pure pentane. The elute was concentrated under reduced pressure. The resulting colorless liquid was used in the next step without further purification (assumed quant.).

*Literature procedure.*^[7]^ To a suspension of Zinc dust (1.30 g, 19.8 mmol, 3.60 equiv.) in dry DMF (6.0 mL) was added 1,2-dibromoethane (95 mL, 1.1 mmol, 0.20 equiv.). The resulting mixture was stirred at 80 °C for 1 h. After cooling down to rt TMSCl (70 mL, 0.55 mmol, 0.10 equiv.) was added and the suspension was stirred at rt for 30 min. A solution of iodide **SI-53** (2.00 g, 5.51 mmol, 1.00 equiv.) in dry DMF (4.0 mL) was added over 2 min which resulted in an exothermic reaction. The mixture was stirred for 10 min and then left to stand without stirring for 10 min. The supernatant was transferred to a cooled (−20 °C) solution of CuCN (444 mg, 4.96 mmol, 0.90 equiv.) and LiCl (420 mg, 9.91 mmol, 1.80 equiv.) in dry DMF (10 mL). After stirring for 15 min neat alkyne **SI-52** (1.69 g, 7.71 mmol, 1.40 equiv.) was added dropwise. The resulting mixture was slowly allowed to warm up to rt over 3 h and further stirred at rt for 16 h. The reaction was quenched by the addition of water (100 mL) and extracted with Et_2_O (4 × 100 mL). The combined organic layers were washed with brine (100 mL), dried over MgSO_4_, filtered and the solvent removed under reduced pressure. The crude mixture was purified by silica gel flash column chromatography (EtOAc/cyclohexane 10% to 20%) to afford **SI-54** (1.45 g, 3.86 mmol, 70%) as a yellow oil.

R*_f_* = 0.40 (EtOAc/hexanes 1:5).

^1^H-NMR (500 MHz, CDCl_3_): δ 7.40–7.30 (m, 5H), 5.58 (d, *J* = 8.3 Hz, 1H), 5.13 (s, 2H), 4.53 (dt, *J* = 8.9, 4.8 Hz, 1H), 3.76 (s, 3H), 2.84 (dd, *J* = 17.0 Hz, 4.6 Hz, 1H), 2.78 (dd, *J* = 17.1, 5.1 Hz, 1H), 0.96 (t, *J* = 7.9 Hz, 9H), 0.56 (q, *J* = 7.9 Hz, 6H) ppm.

^13^C-NMR (126 MHz, CDCl_3_): δ 170.9, 155.7, 136.3, 128.7, 128.3, 128.2, 101.5, 86.1, 67.2, 52.8, 52.7, 24.4, 7.5, 4.5 ppm.

(+)-HR-ESI-MS: *m/z* 376.1939 calculated for C_20_H_30_NO_4_Si^+^ [M+H]^+^; found 376.1936.

IR (neat): 3333 (w), 2954 (w), 2875 (w), 2176 (w), 1726 (s), 1506 (m), 1456 (w), 1438 (w), 1346 (m), 1213 (s), 1047 (m), 1017 (m), 737 (m), 698 (m), 601 (w).

${[\alpha]}_{D}^{23}$: + 82.5 (*c* = 0.60, CHCl_3_).

The obtained data are in agreement with the values reported in the literature.^[7]^

To a solution of methyl ester **SI-54** (1.02 g, 2.72 mmol, 1.00 equiv.) in THF (20 mL) and water (10 mL) was added LiOH (98.0 mg, 4.08 mmol, 1.50 equiv.) at 0 °C. The resulting mixture was allowed to warm up to rt and stirred for 16 h. The reaction was quenched by the addition of aq. HCl (1 M) to reach pH = 4. The aq. layer was extracted with EtOAc (3 × 200 mL). The combined organic layers were washed with brine (200 mL), dried over MgSO_4_, filtered and the solvent removed under reduced pressure. The resulting crude **SI-55** was used in the next step without further purification (assumed quant.).

*Literature procedure.*^[7]^ To a solution of H-Asn(Trt)-OMe (1.06 g, 2.72 mmol, 1.00 equiv.) in dry DMF (15 mL) was added a solution of carboxylic acid **SI-55** (assumed 983 mg, 2.72 mmol, 1.00 equiv.) in dry DMF (15 mL) and the resulting mixture was cooled to 0 °C. DIPEA (1.14 mL, 6.53 mmol, 2.40 equiv.), HOAt (444 mg, 3.26 mmol, 1.20 equiv.) and EDC∙HCl (626 mg, 3.26 mmol, 1.20 equiv.) were added and the mixture was allowed to warm up to rt and stirred for 2 d. The reaction was quenched by the addition of aq. HCl (1 M, 50 mL) and the aq. layer was extracted with EtOAc (3 × 150 mL). The combined organic layers were washed with brine (150 mL), dried over MgSO_4_, filtered and the solvent was removed under reduced pressure. The crude mixture was purified by silica gel flash column chromatography (EtOAc/cyclohexane 12% to 100%) to afford **SI-56** (612 mg, 0.836 mmol, 31%) as an off-white solid.

R*_f_* = 0.58 (EtOAc/hexanes 1:1).

^1^H-NMR (500 MHz, CDCl_3_): δ 7.40–7.21 (m, 14H), 7.15 (d, *J* = 7.2 Hz, 7H), 6.69 (s, 1H), 5.44 (d, *J* = 7.9 Hz, 1H), 5.10 (d, *J* = 12.3 Hz, 1H), 5.06 (d, *J* = 12.4 Hz, 1H), 4.79 (dt, *J* = 8.8, 4.3 Hz, 1H), 4.29 (q, *J* = 6.8 Hz, 1H), 3.65 (s, 3H), 3.06 (dd, *J* = 15.8, 4.2 Hz, 1H), 2.76 (td, *J* = 16.9, 5.3 Hz, 2H), 2.65 (dd, *J* = 16.8, 7.0 Hz, 1H), 0.94 (t, *J* = 7.9 Hz, 9H), 0.54 (q, *J* = 7.9 Hz, 6H) ppm.

^13^C-NMR (126 MHz, CDCl_3_): δ 171.0, 169.9, 169.3, 155.9, 144.4, 136.3, 128.8, 128.6, 128.3, 128.18, 128.15, 127.3, 102.1, 85.9, 71.1, 67.3, 53.6, 52.9, 49.3, 38.4, 24.3, 7.6, 4.5 ppm.

(+)-HR-ESI-MS: *m/z* 732.3463 calculated for C_43_H_50_N_3_O_6_Si [M+H]^+^; found 732.3468.

IR (neat): 3294 (w), 3033 (w), 2951 (w), 2874 (w), 2179 (w), 1741 (m), 1661 (s), 1529 (m), 1491 (m), 1444 (m), 1327 (w), 1258 (m), 1233 (m), 1019 (m), 904 (w), 850 (w), 767 (w), 725 (m), 696 (s), 637 (m), 623 (m), 569 (w), 501 (w), 458 (w).

${[\alpha]}_{D}^{23}$: + 22.5 (*c* = 0.92, CHCl_3_).

M.p.: 151.2–152.4 °C.

The obtained data are in agreement with the values reported in the literature.^[7]^

Methyl ester **SI-56** (26.0 mg, 35.5 µmol, 1.00 equiv.) was dissolved in a mixture of THF and water (3:1, 0.4 mL) and cooled to 0 °C. An aq. soln. of LiOH (1 M, 89 µL, 89 µmol, 2.50 equiv.) was added dropwise and the resulting mixture was stirred at this temperature for 1 h. The reaction was quenched by the addition of aq. HCl (1 M) to reach pH 4. The aq. layer was extracted with EtOAc (3 × 15 mL). The combined organic layers were washed with brine (15 mL), dried over MgSO_4_, filtered and the solvent was removed under reduced pressure. The resulting crude product **39** was used in the next step without further purification (assumed quant.).

**Synthesis of 45 & 46**

A mixture of **43** and **44** (30.0 mg, 21.7 µmol, 1.00 equiv.) was dissolved in dry DMF (0.5 mL) and treated with piperidine (2.2 µL, 2.2 µmol, 1.00 equiv.). the resulting mixture was stirred at rt for 1 h and then quenched by the addition of sat. aq. NaHCO_3_ (10 mL). The aq. layer was extracted with EtOAc (3 × 15 mL). The combined organic layers were washed with brine (15 mL), dried over MgSO_4_, filtered and the solvent was removed under reduced pressure. The resulting crude mixture of **SI-57** and **SI-58** was used in the next step without further purification (assumed quant.).

To a solution of crude carboxylic acid **39** (assumed: 25.5 mg, 35.5 µmol, 1.50 equiv.) and a crude mixture of amines **SI-57** and **SI-58** (assumed: 25.2 mg, 21.7 µmol, 1.00 equiv.) in dry DMF (1.0 mL) were added HATU (16.5 mg, 43.4 µmol, 2.00 equiv.) and DIPEA (18 µL, 110 µmol, 5.00 equiv.) at 0 °C. The resulting mixture was stirred at rt for 19 h. The reaction was quenched by the addition of sat. aq. NH_4_Cl (25 mL) and the aq. layer was extracted with EtOAc (3 × 50 mL). The combined organic layers were washed with brine (50 mL), dried over MgSO_4_, filtered and the solvent was removed under reduced pressure. Purification by silica gel column chromatography (EtOAc/hexane 0% to 100%) delivered a mixture of products **45** and **46** (combined: 37.7 mg, 20.3 µmol, 93%) as an orange gum. *(Note:* ***45*** *and* ***46*** *were not separable by silica gel column chromatography nor prep-HPLC and therefore no analytics are shown here for these compounds.)*

**Reference of the Supporting Information**

[1] R. P. Loach, O. S. Fenton, K. Amaike, D. S. Siegel, E. Ozkal, M. Movassaghi, *J. Org. Chem.* **2014**, *79*, 11254–11263.

[2] Y. Hattori, T. Asano, M. Kirihata, Y. Yamaguchi, T. Wakamiya, *Tetrahedron Lett.* **2008**, *49*, 4977–4980.

[3] N. A. Isley, Y. Endo, Z.-C. Wu, B. C. Covington, L. B. Bushin, M. R. Seyedsayamdost, D. L. Boger, *J. Am. Chem. Soc.* **2019**, *141*, 17361–17369.

[4] J. Garfunkle, F. S. Kimball, J. D. Trzupek, S. Takizawa, H. Shimamura, M. Tomishima, D. L. Boger, *J. Am. Chem. Soc.* **2009**, *131*, 16036–16038.

[5] F. Marchiori, G. Borin, A. Calderan, G. Chessa, *Int. J. Pept. Protein Res.* **1987**, *30*, 822–831.

[6] T. P. Boyle, J. B. Bremner, Z. Brkic, J. A. V. Coates, N. K. Dalton, J. Deadman, P. A. Keller, J. Morgan, S. G. Pyne, D. I. Rhodes, M. J. Robertson, *Peptidic Compounds*, **2006**, WO2006074501A1.

[7] M. Nesic, D. B. Ryffel, J. Maturano, M. Shevlin, S. R. Pollack, D. R. Jr. Gauthier, P. Trigo-Mouriño, L.-K. Zhang, D. M. Schultz, J. M. McCabe Dunn, L.-C. Campeau, N. R. Patel, D. A. Petrone, D. Sarlah, *J. Am. Chem. Soc.* **2022**, *144*, 14026–14030.

^1^H-NMR (400 MHz, 25 °C) in DMSO-*d_6_*


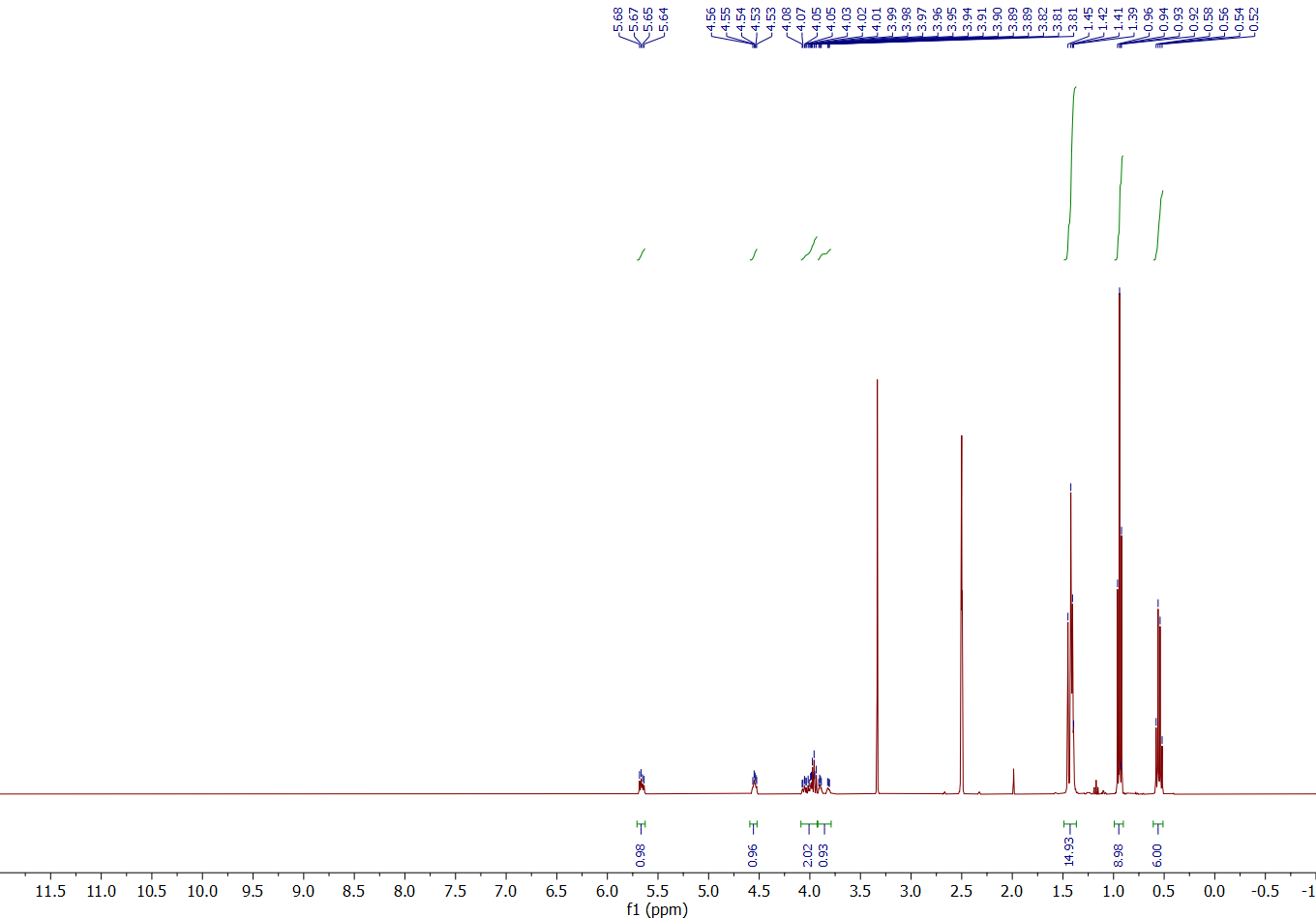


^1^H-NMR (400 MHz, 80 °C) in DMSO-*d_6_*


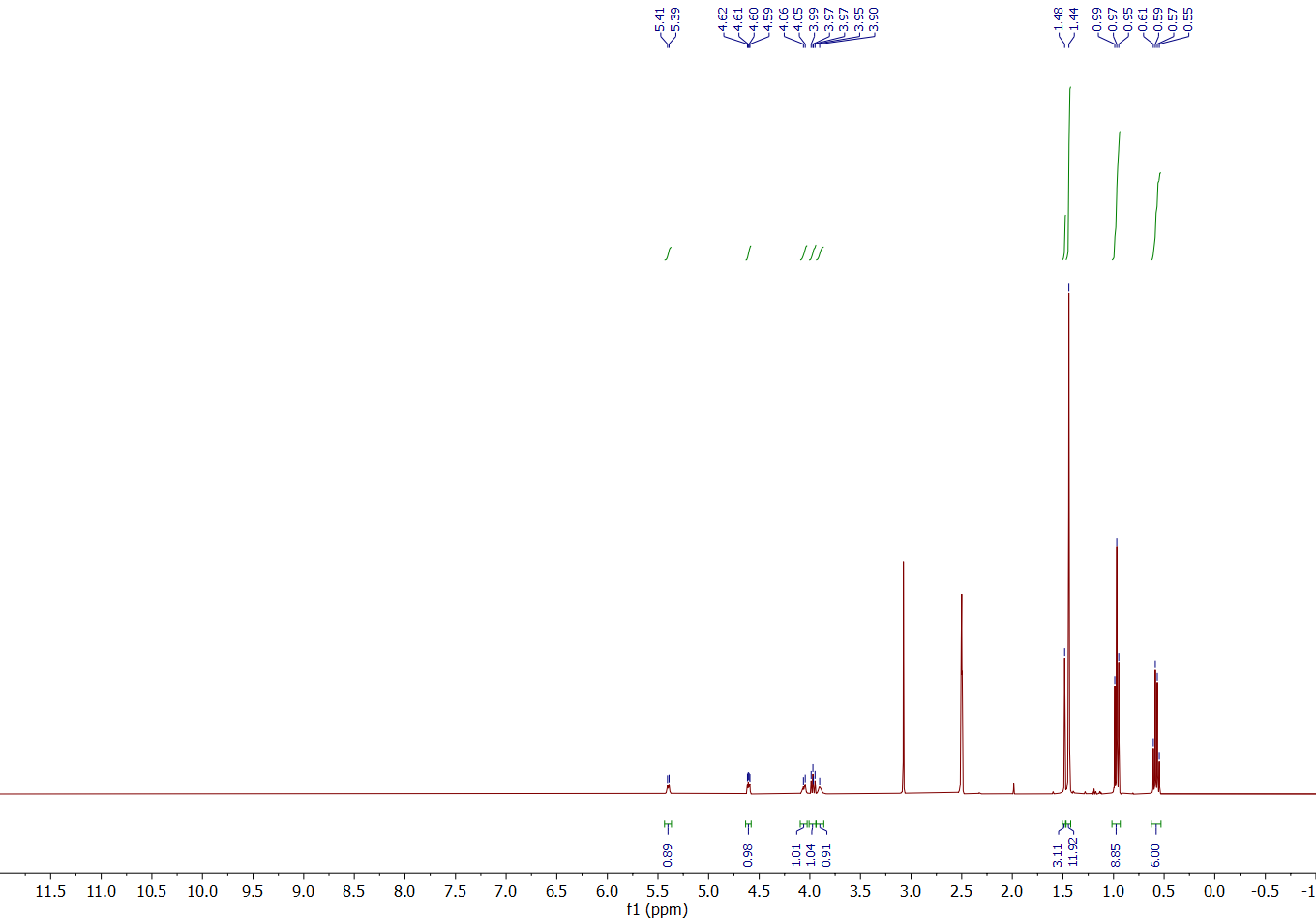


^13^C-NMR (126 MHz, 25 °C) in DMSO-*d_6_*


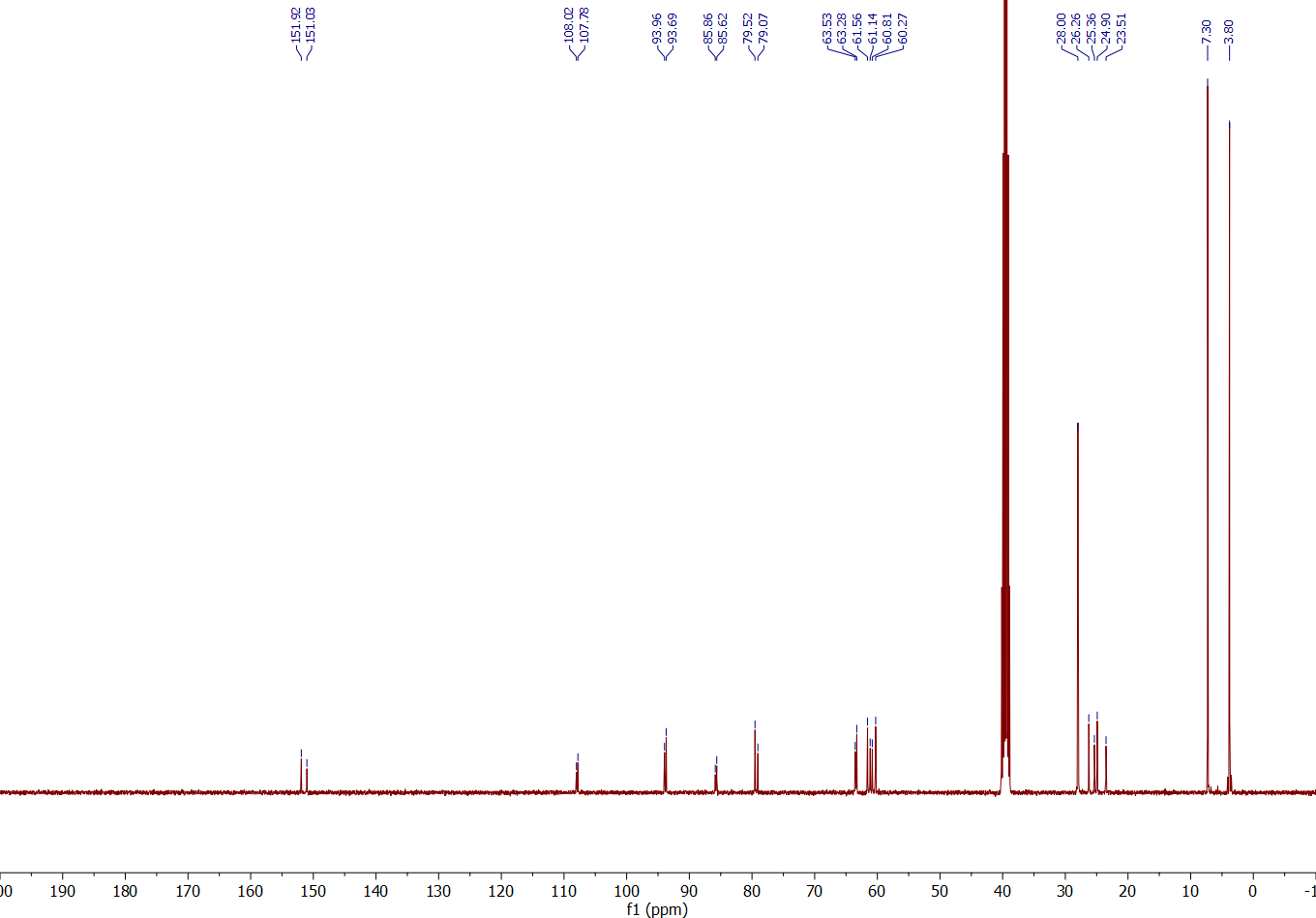


^13^C-NMR (126 MHz, 80 °C) in DMSO-*d_6_*


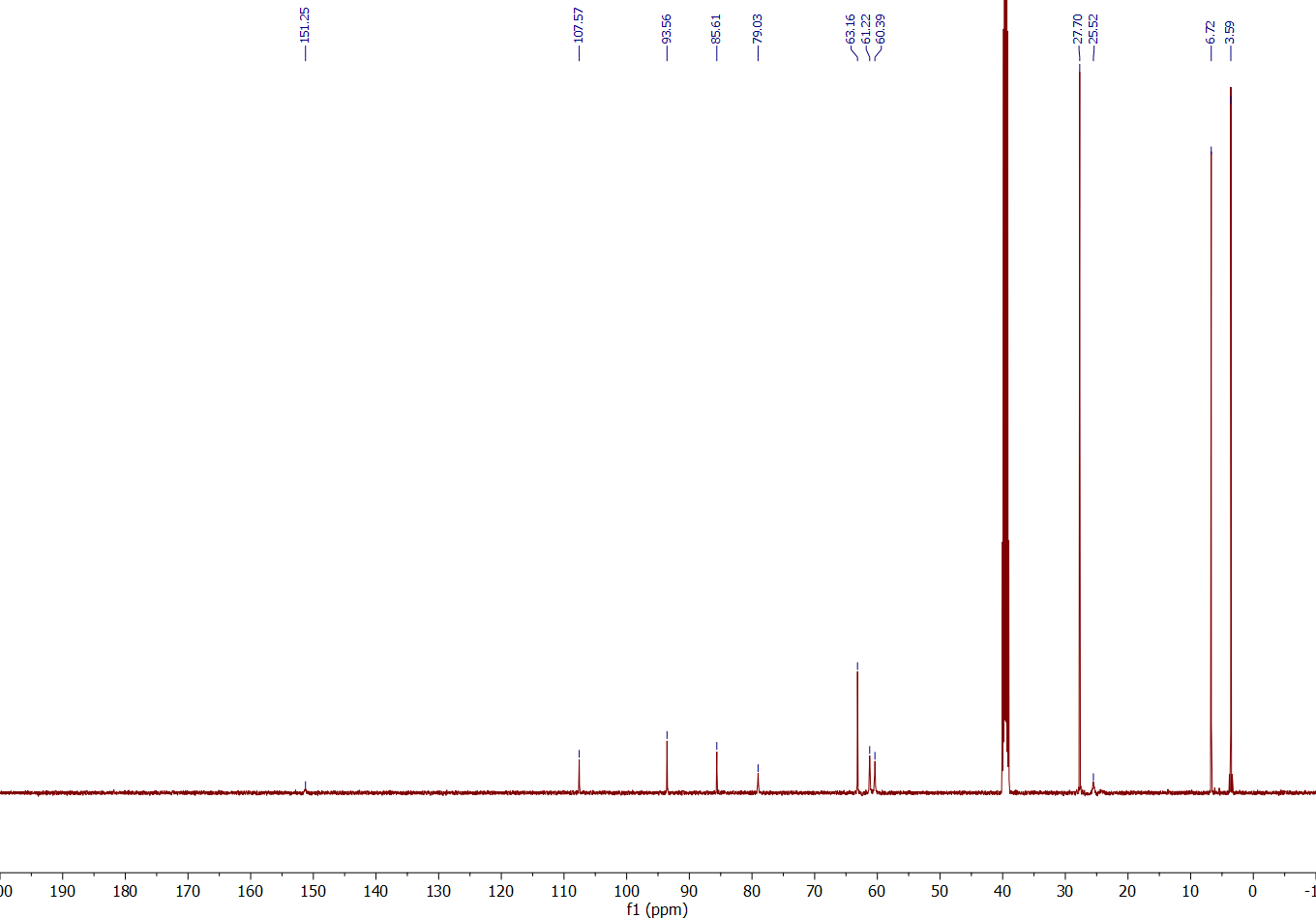


^1^H-NMR (400 MHz, 25 °C) in DMSO-*d_6_*


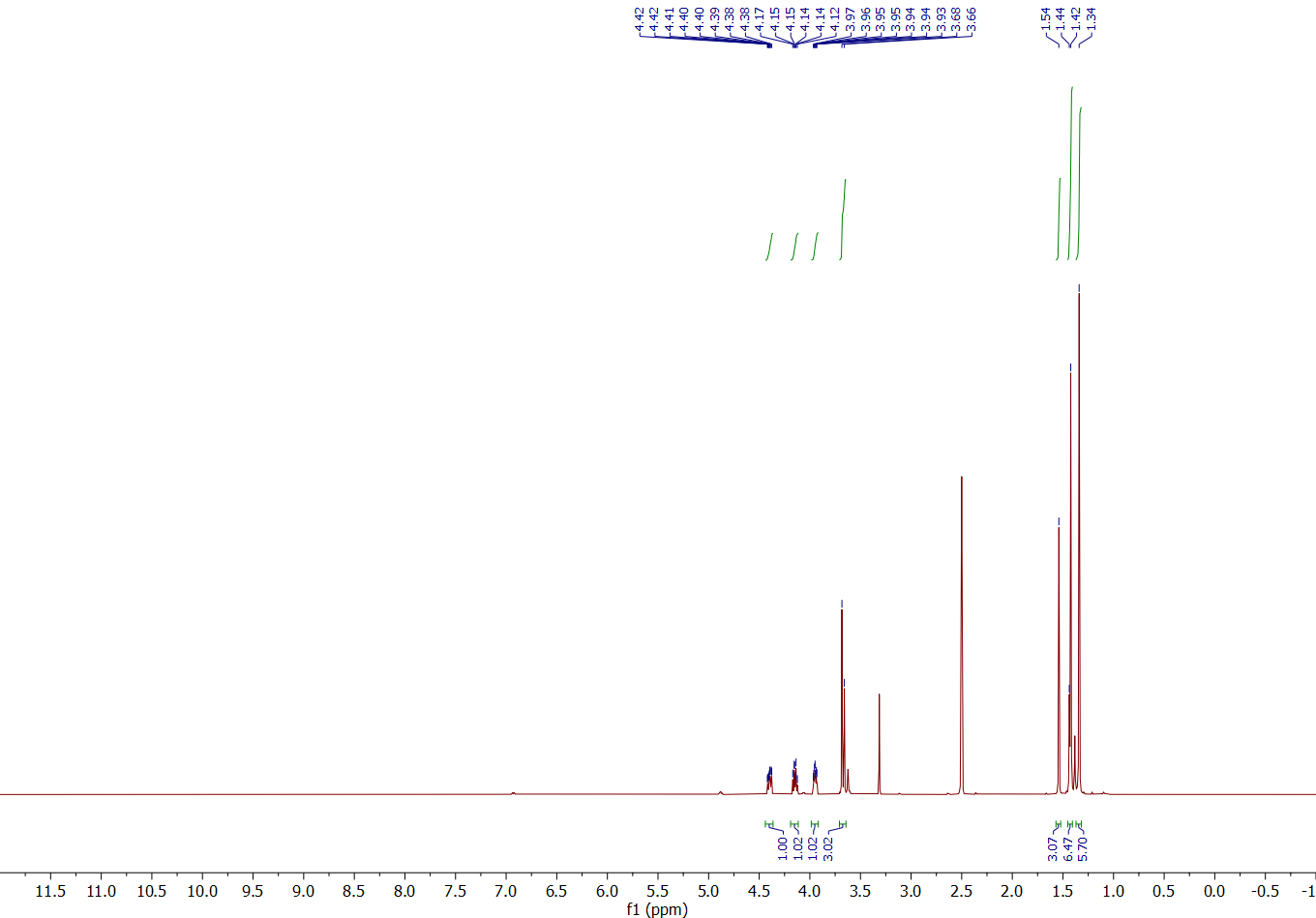

^1^H-NMR (400 MHz, 80 °C) in DMSO-*d_6_*


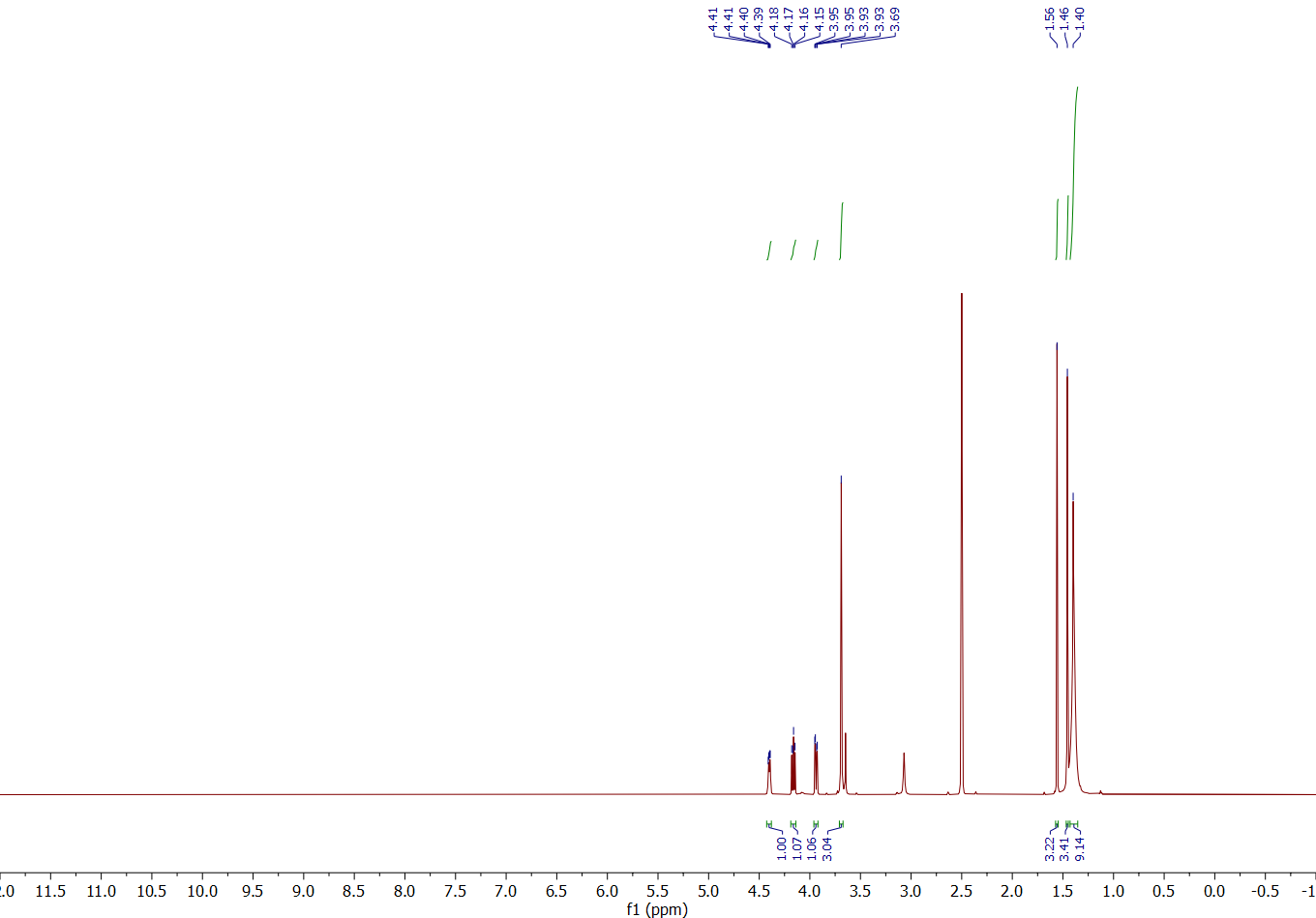


^13^C-NMR (126 MHz, 25 °C) in DMSO-*d_6_*


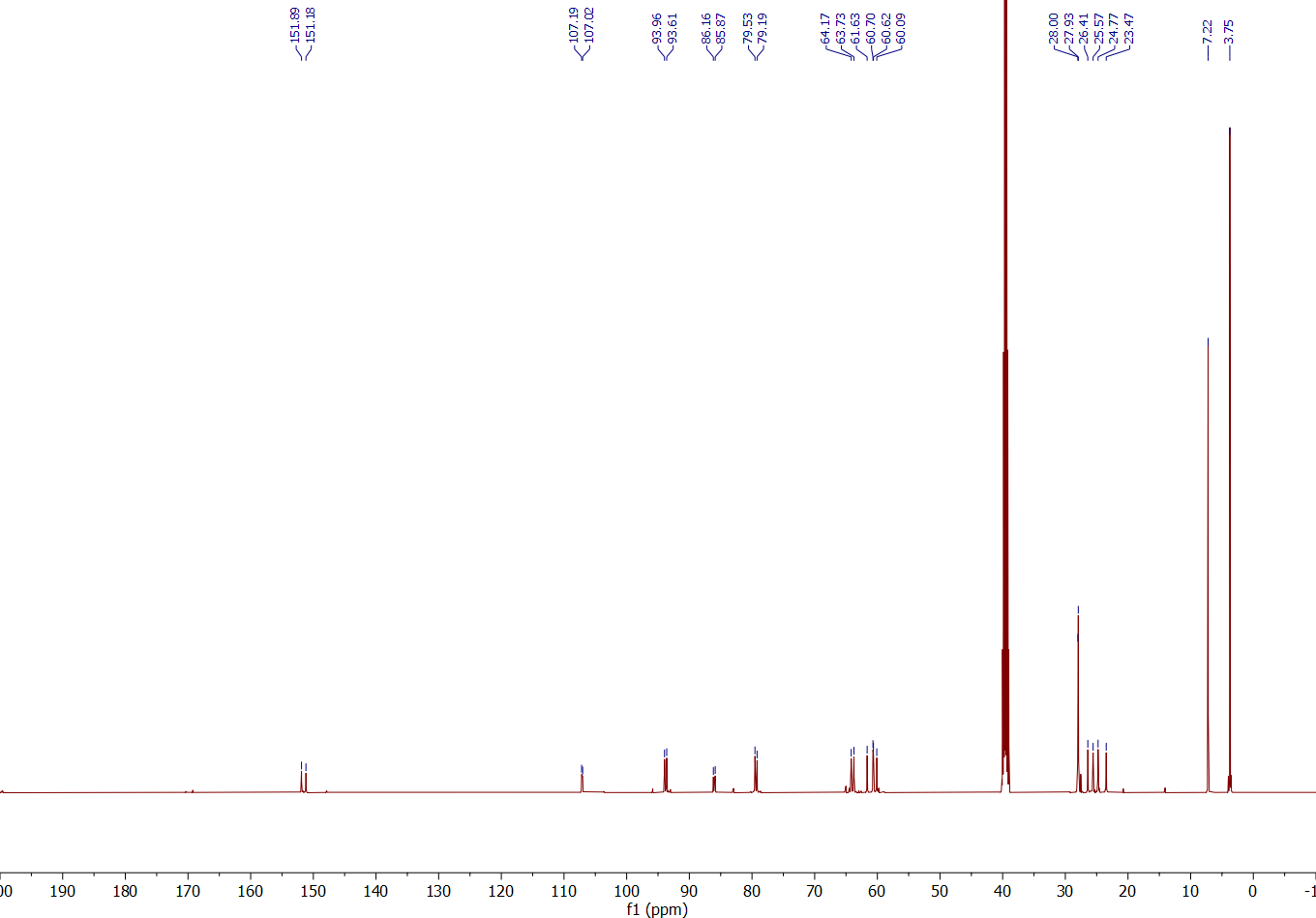


^13^C-NMR (126 MHz, 80 °C) in DMSO-*d_6_*


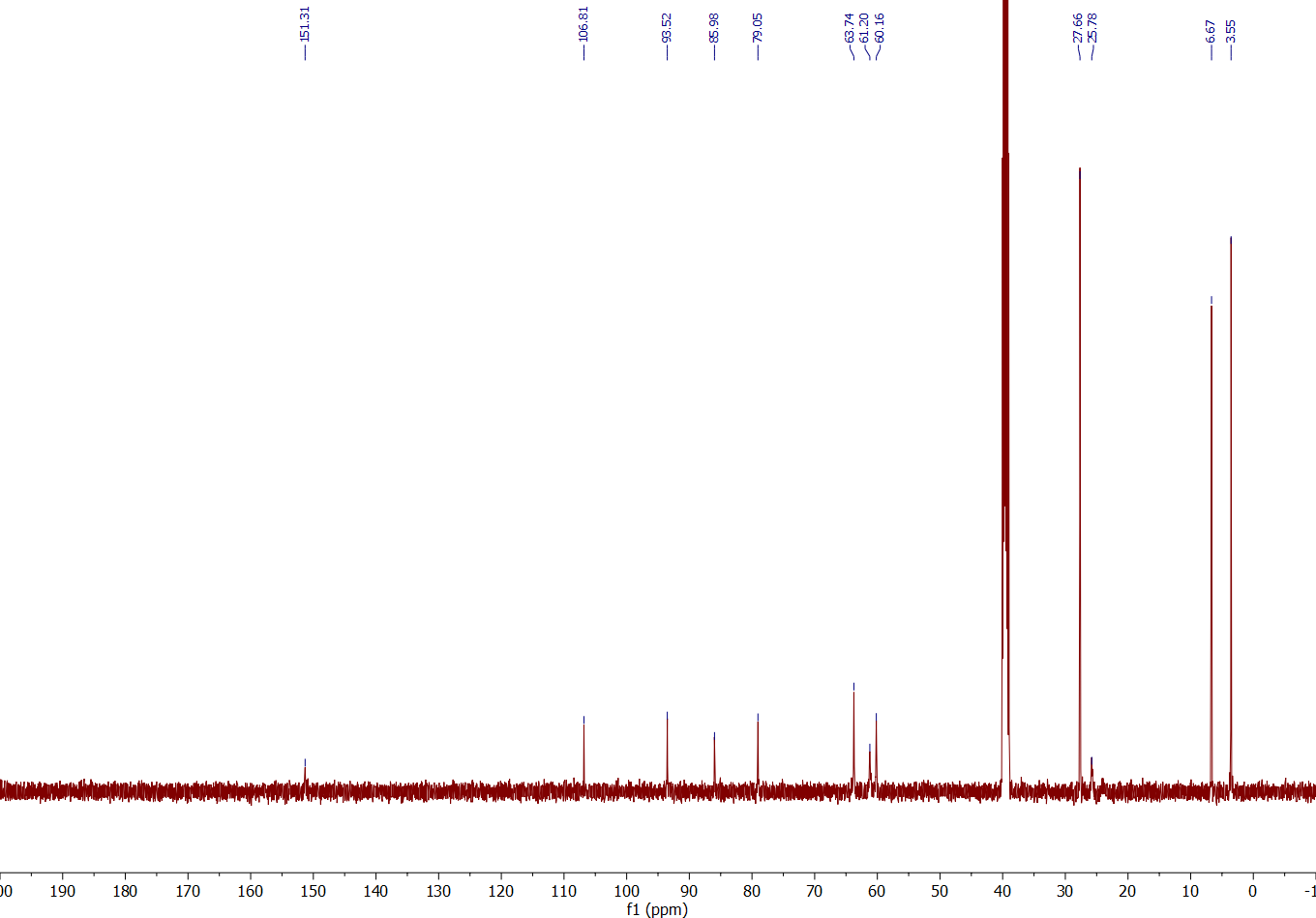


^1^H-NMR (400 MHz) in CDCl_3_


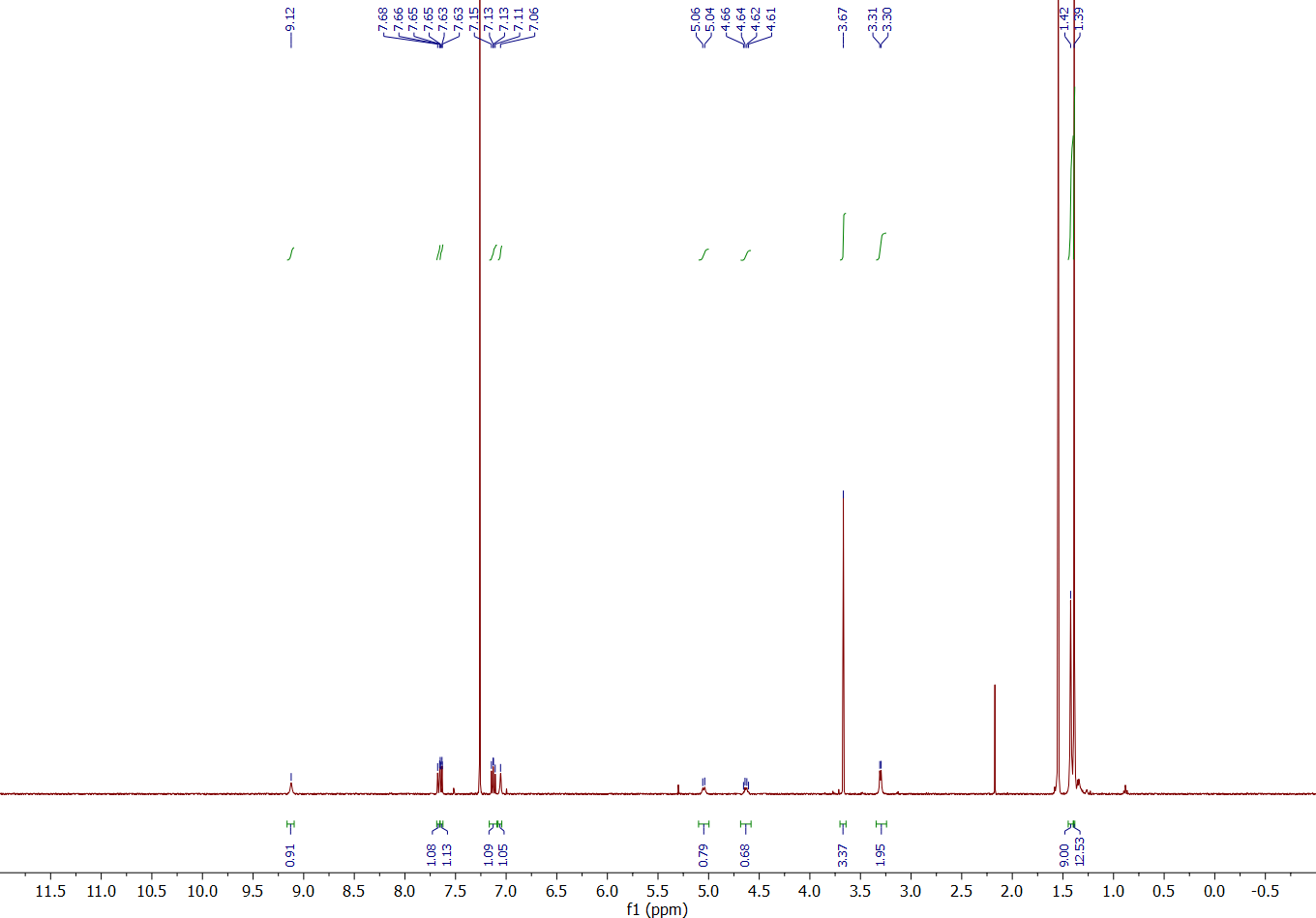


^13^C-NMR (126 MHz) in CDCl_3_


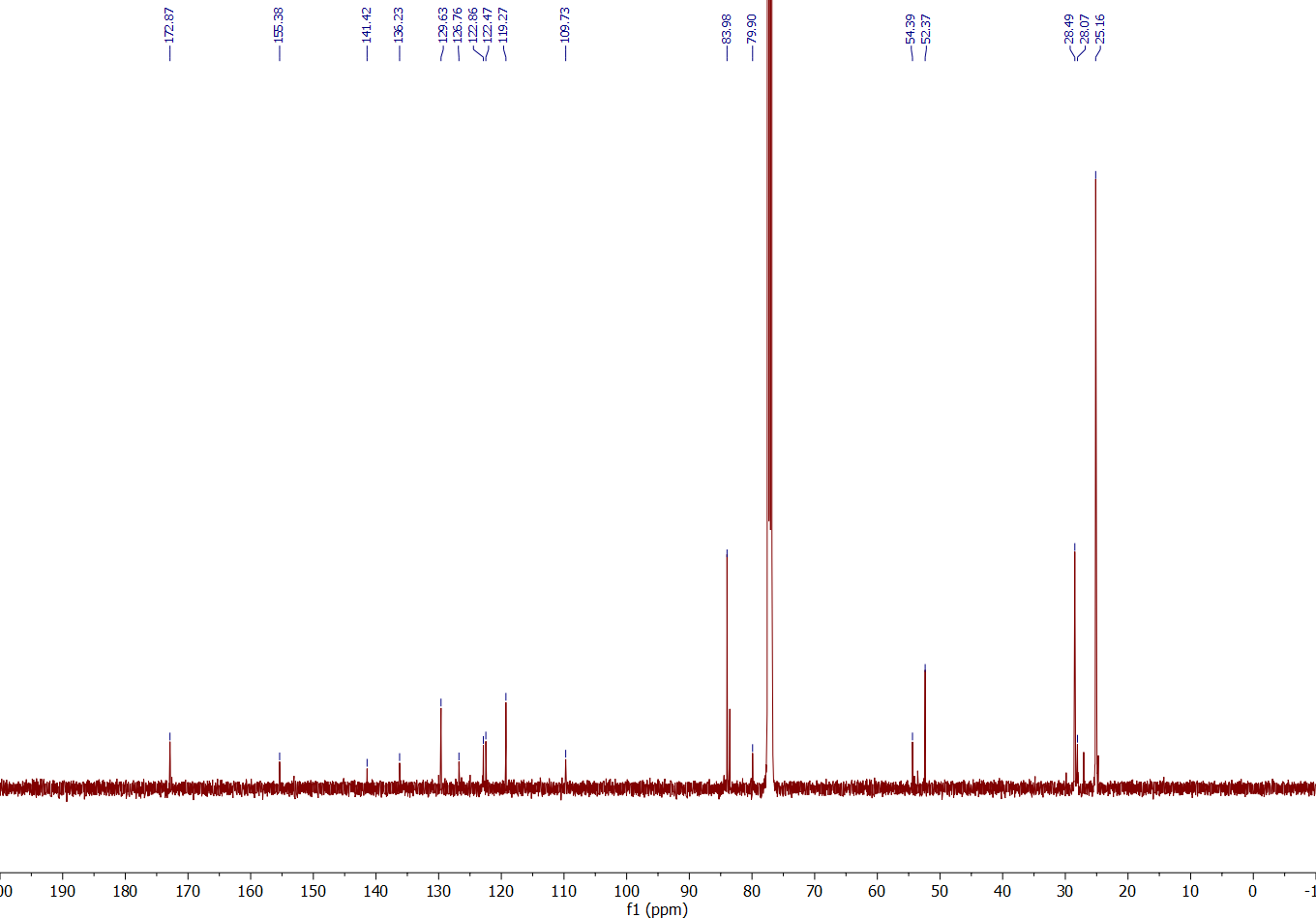


^1^H-NMR (500 MHz) in methanol-*d_4_*


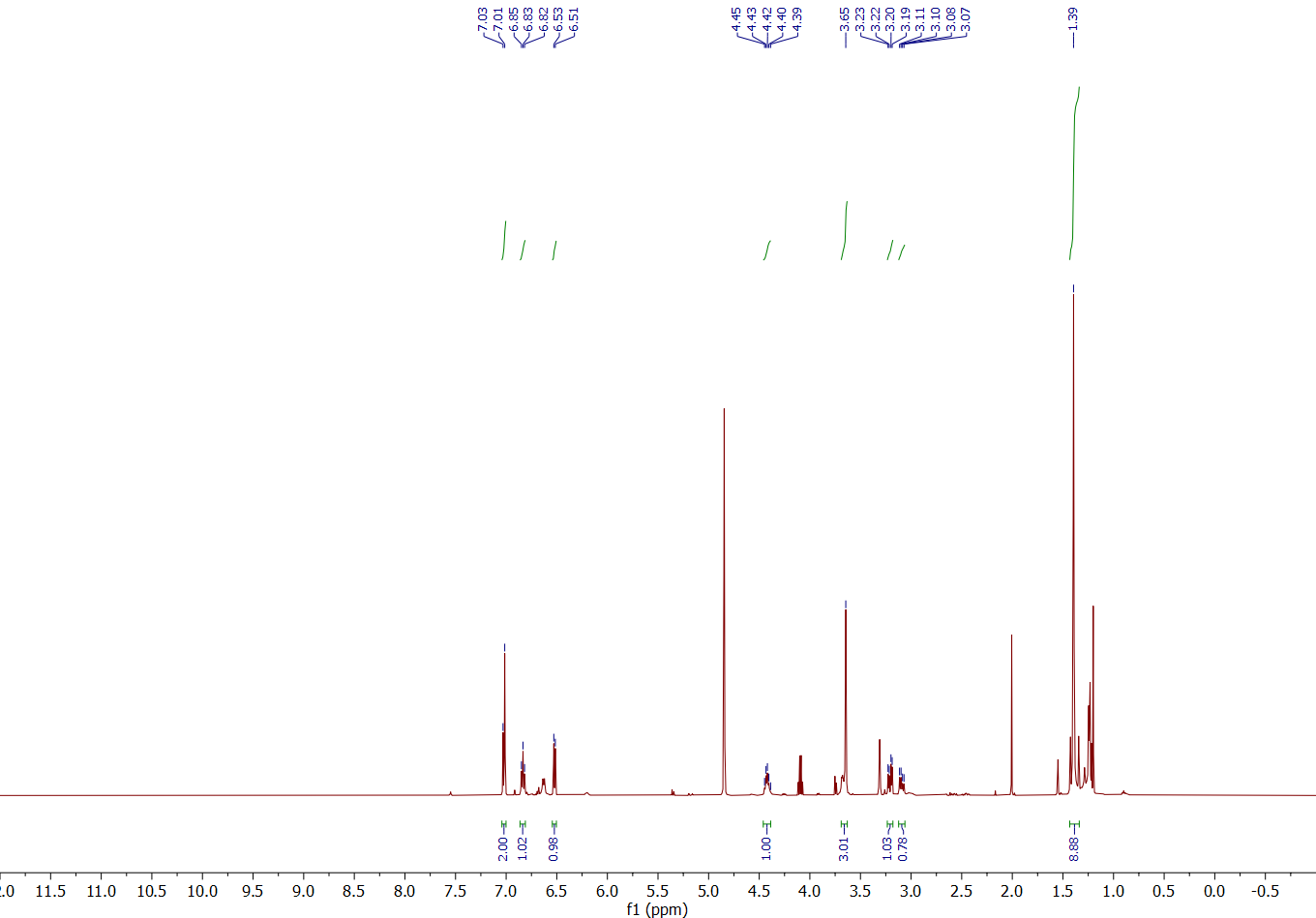


^13^C-NMR (126 MHz) in methanol-*d_4_*


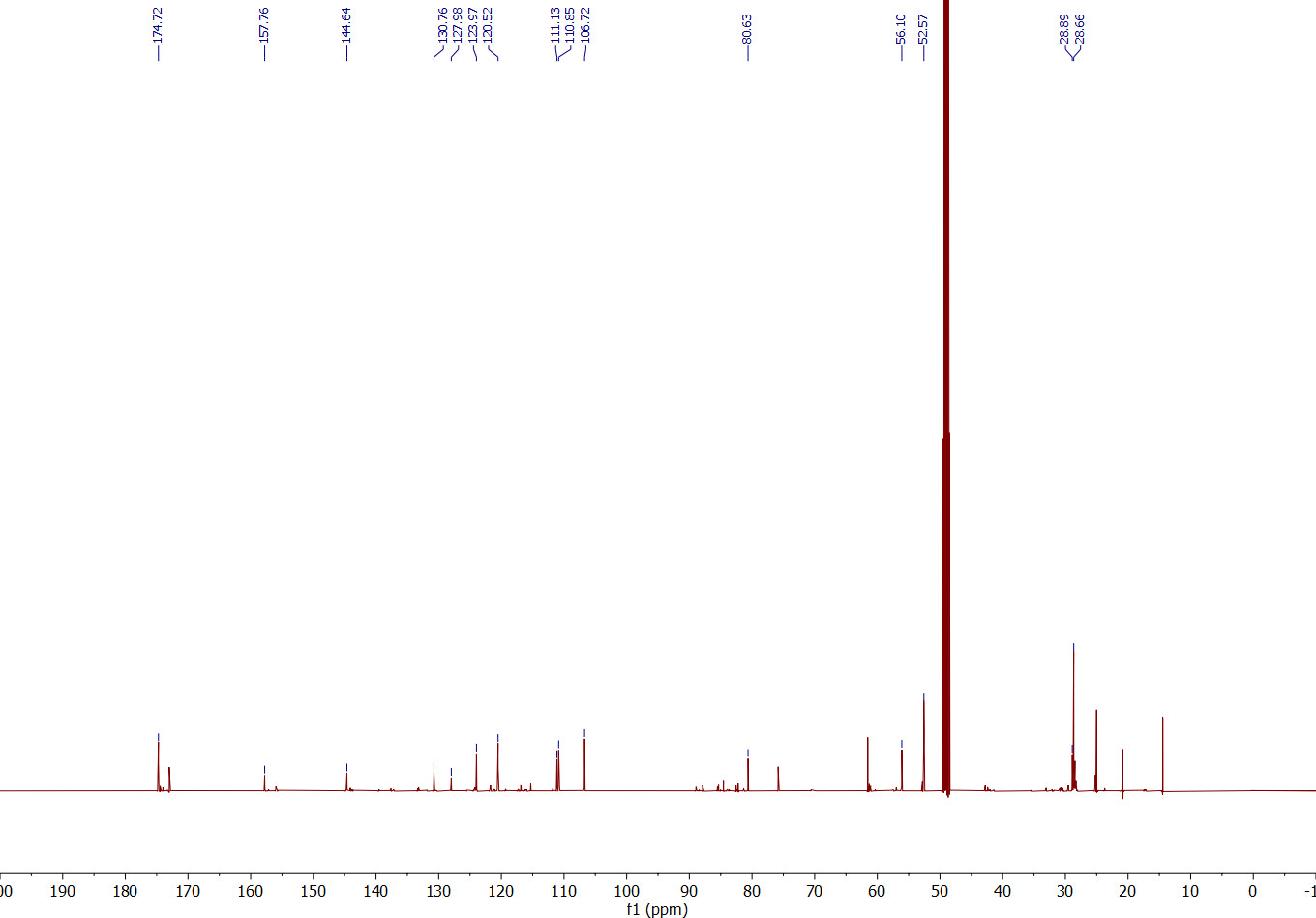


^1^H-NMR (400 MHz, 25 °C) in DMSO-*d_6_*


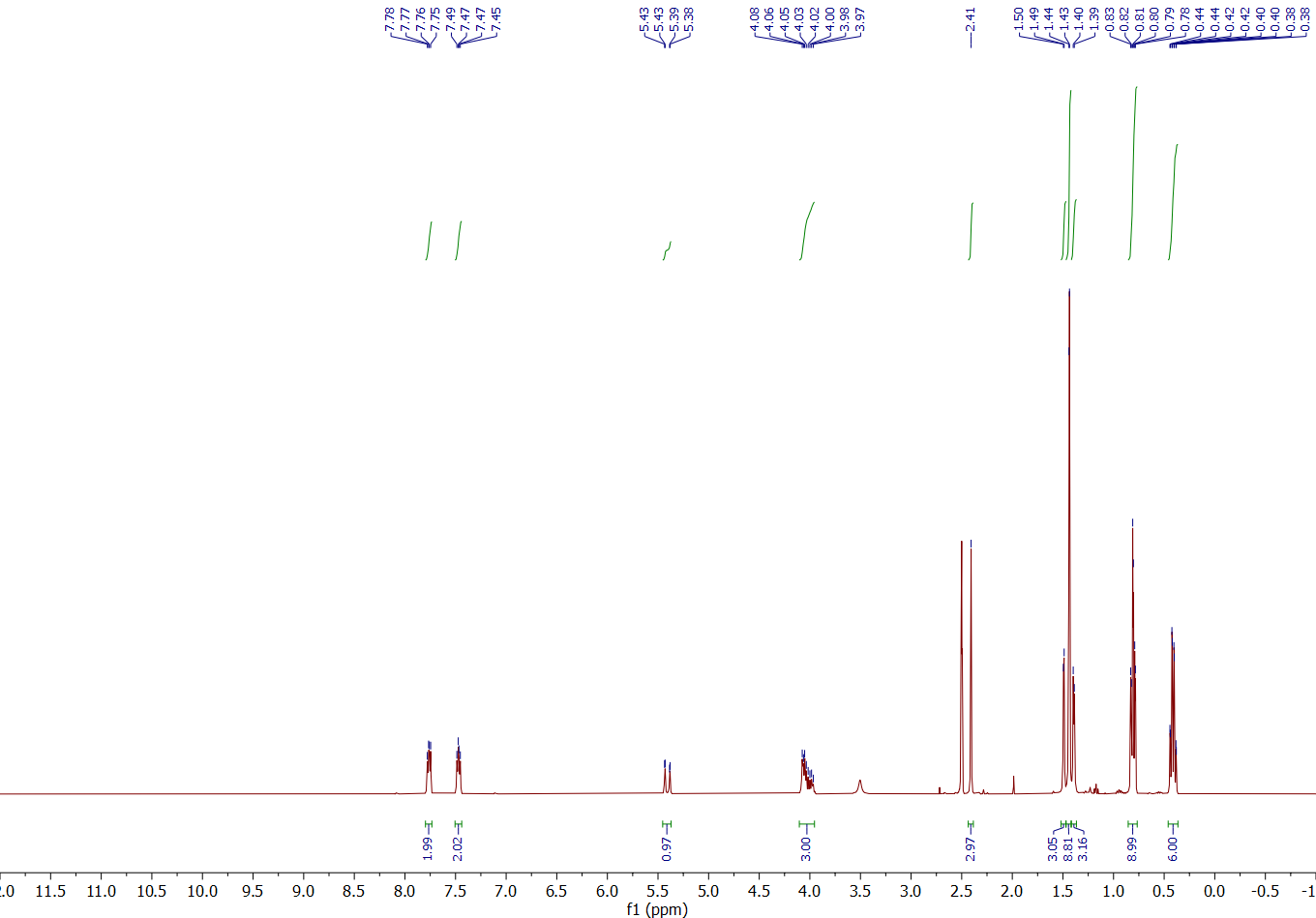


^1^H-NMR (400 MHz, 80 °C) in DMSO-*d_6_*


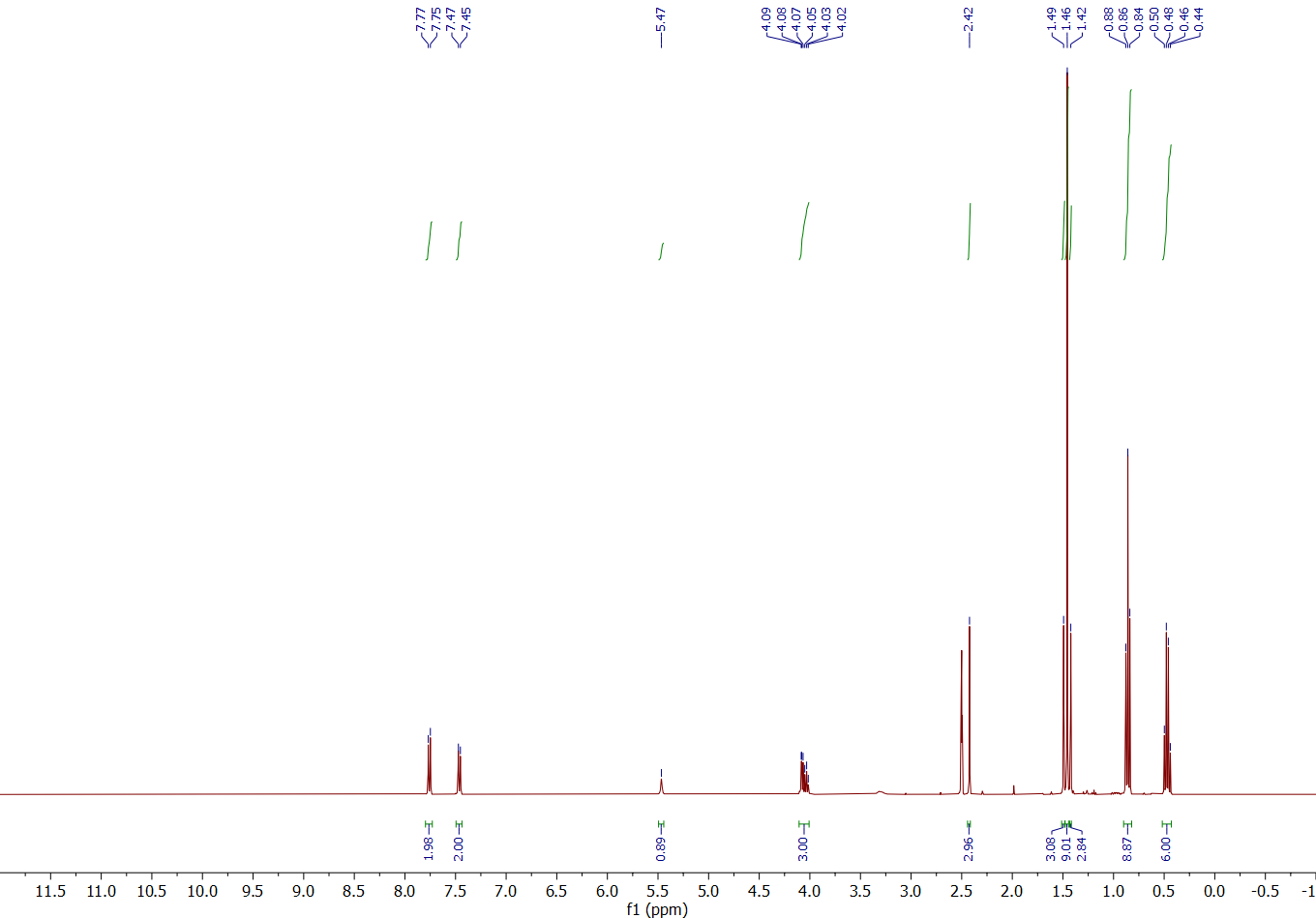


^13^C-NMR (126 MHz, 25 °C) in DMSO-*d_6_*


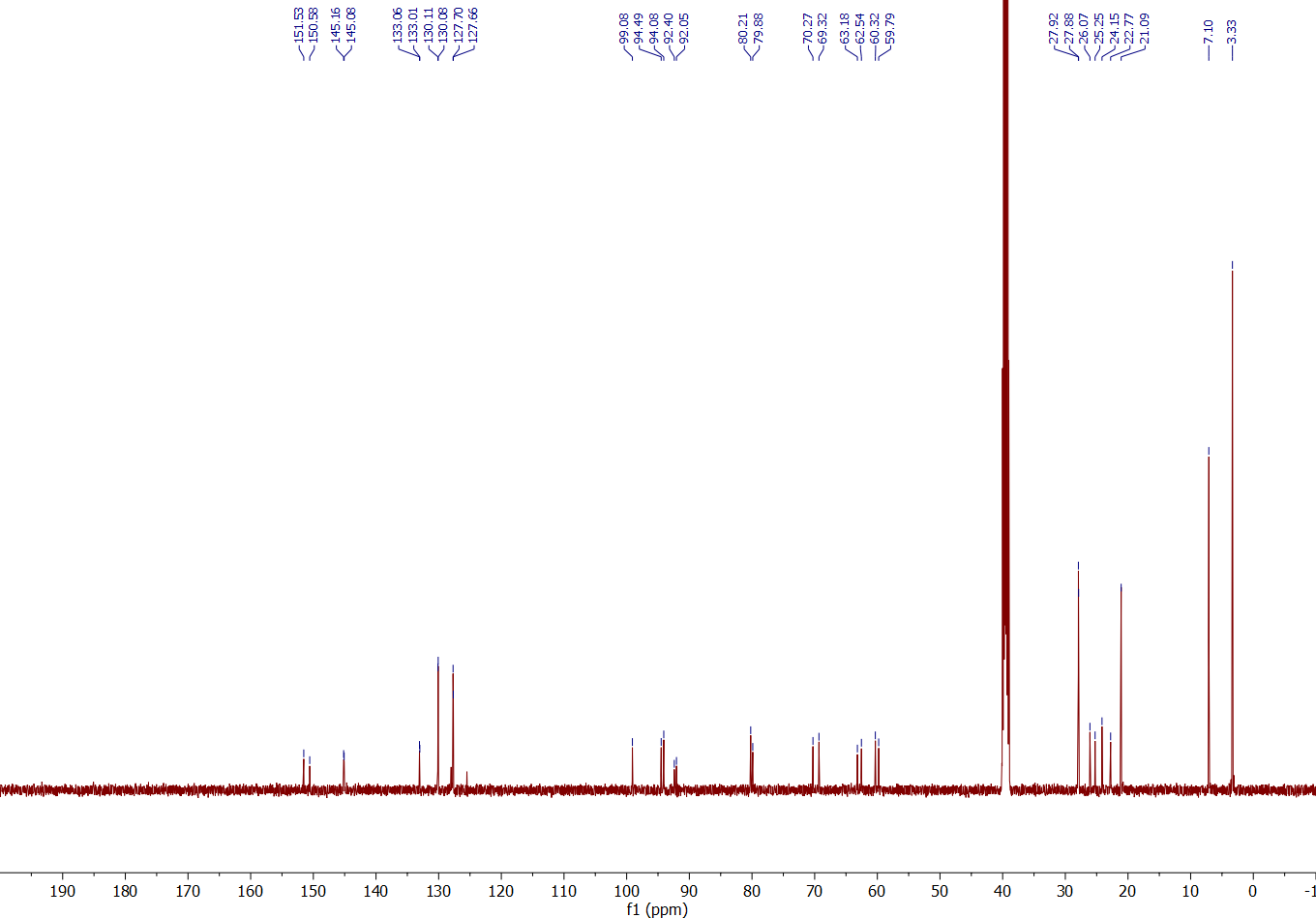


^1^H-NMR (400 MHz) in CDCl_3_


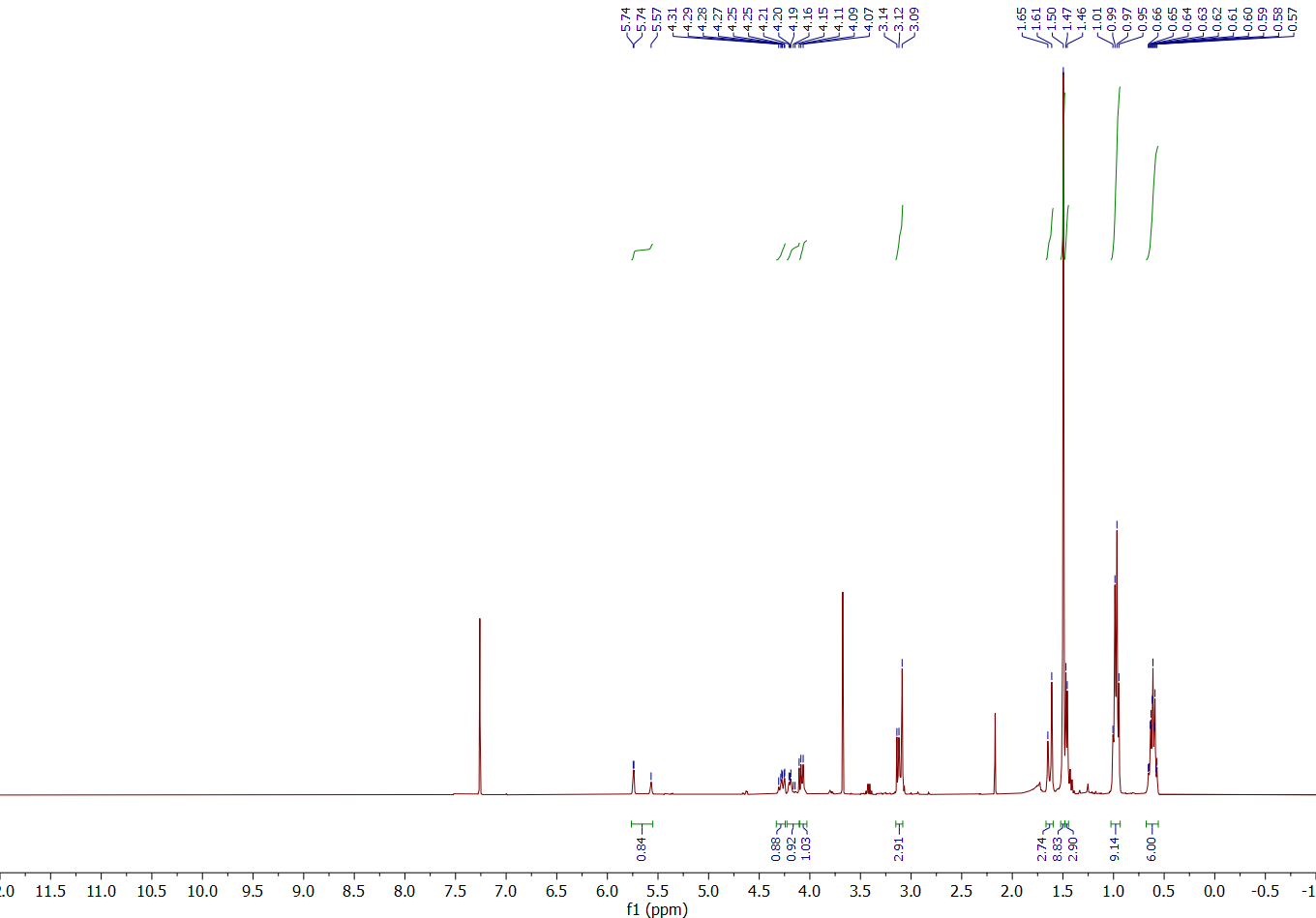


^1^H-NMR (500 MHz) in CDCl_3_

^
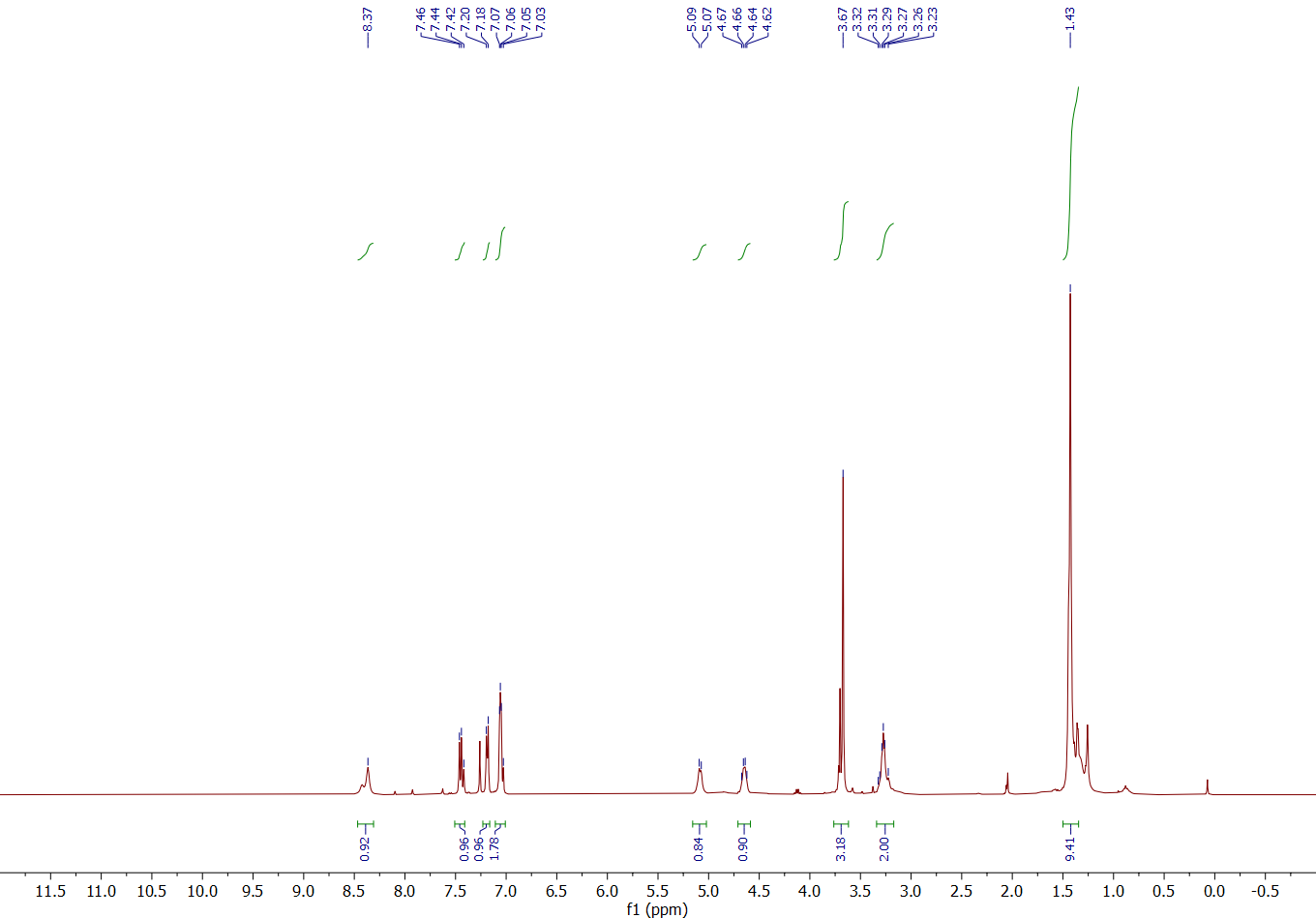
^

^13^C-NMR (101 MHz) in CDCl_3_

^
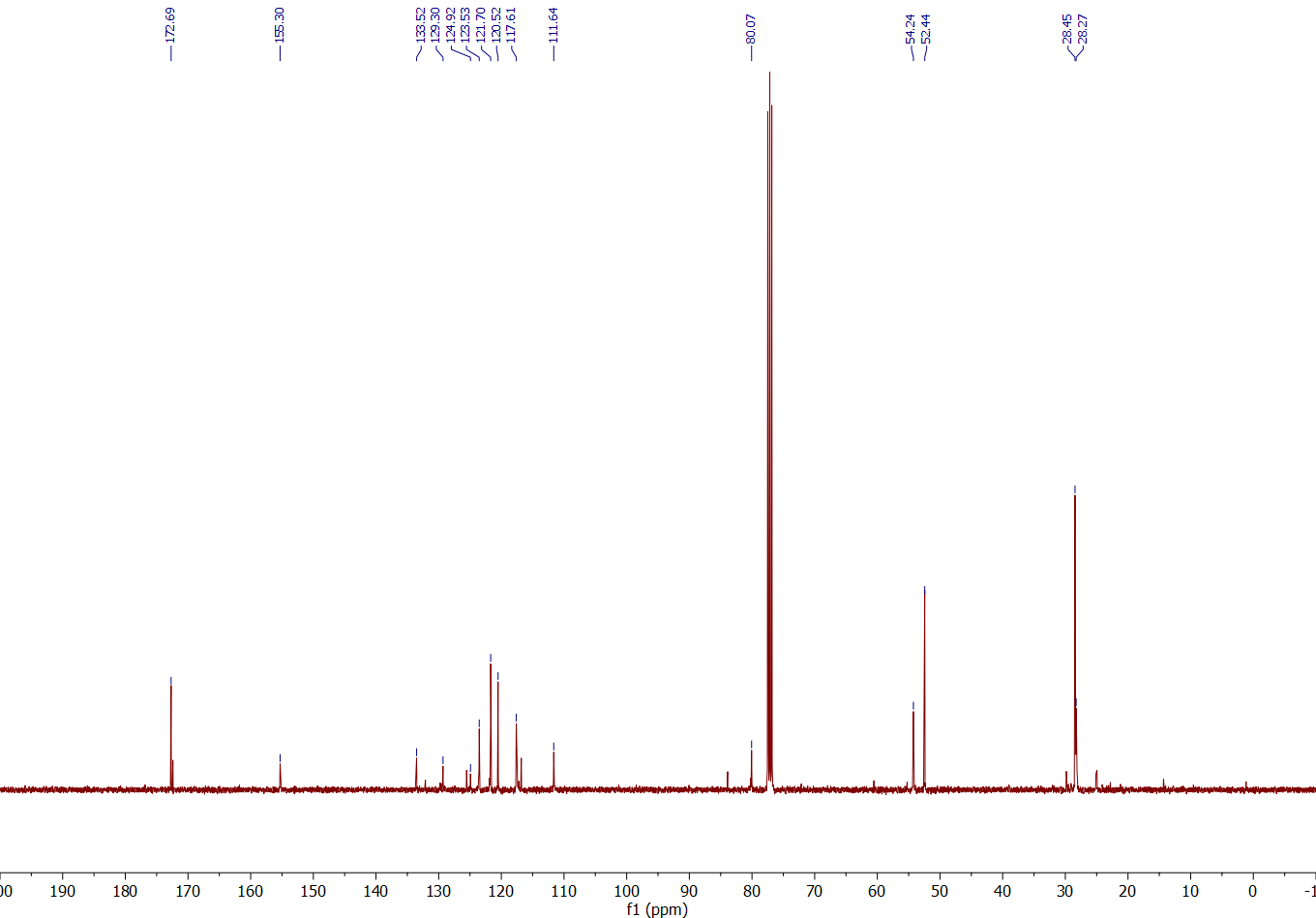
^

^1^H-NMR (500 MHz) in CDCl_3_

^
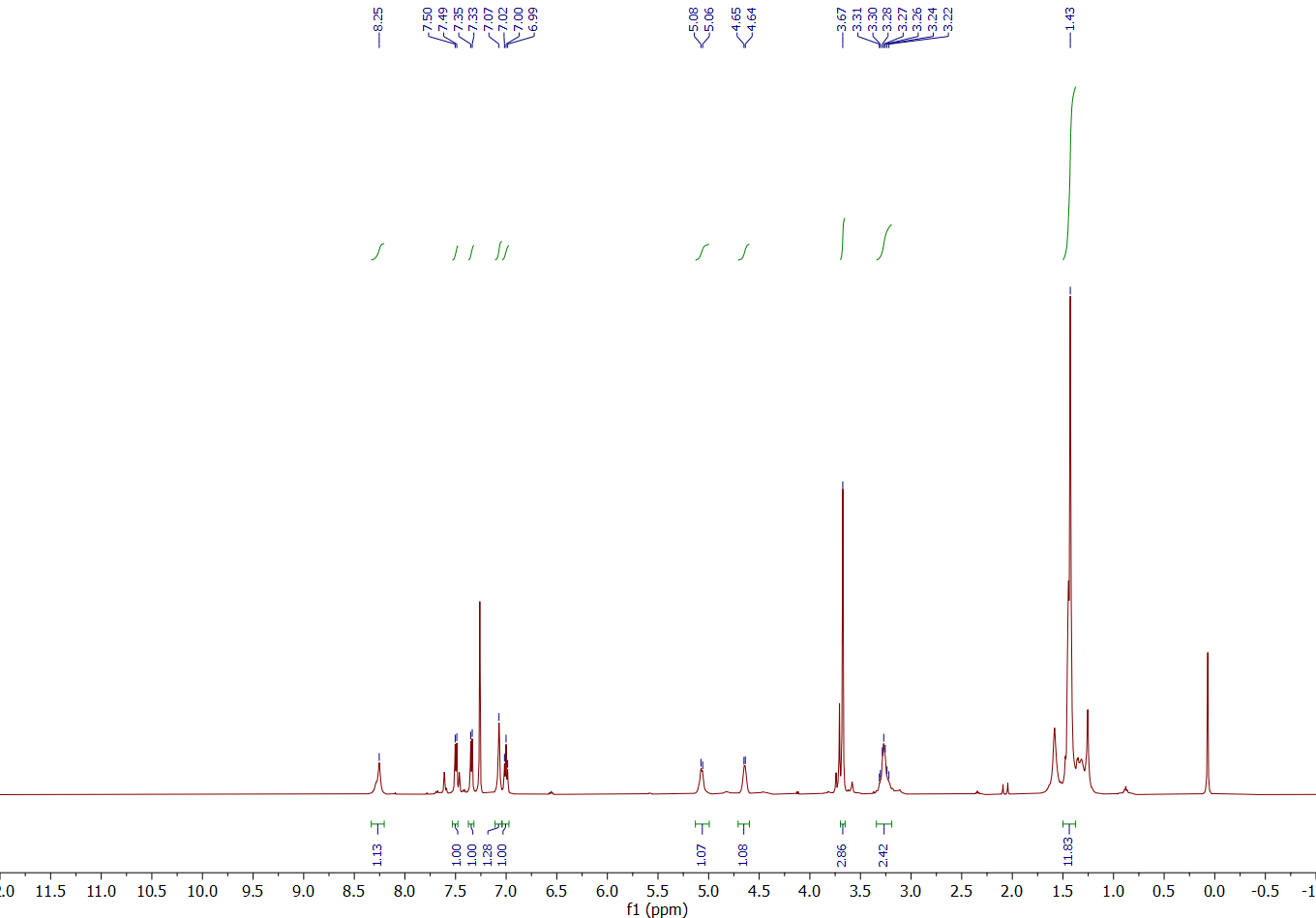
^

^13^C-NMR (126 MHz) in CDCl_3_

^
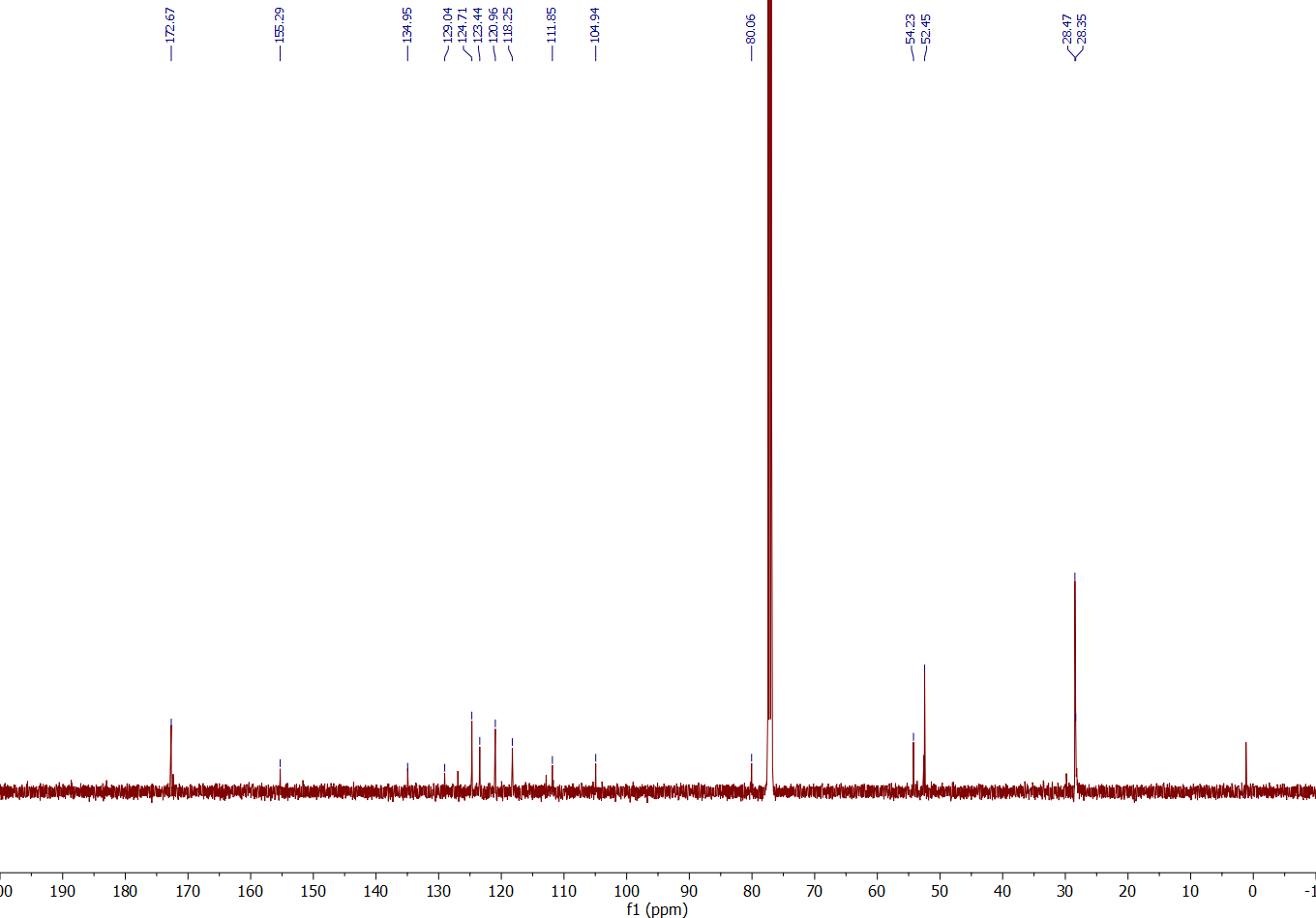
^

^1^H-NMR (400 MHz) in CDCl_3_


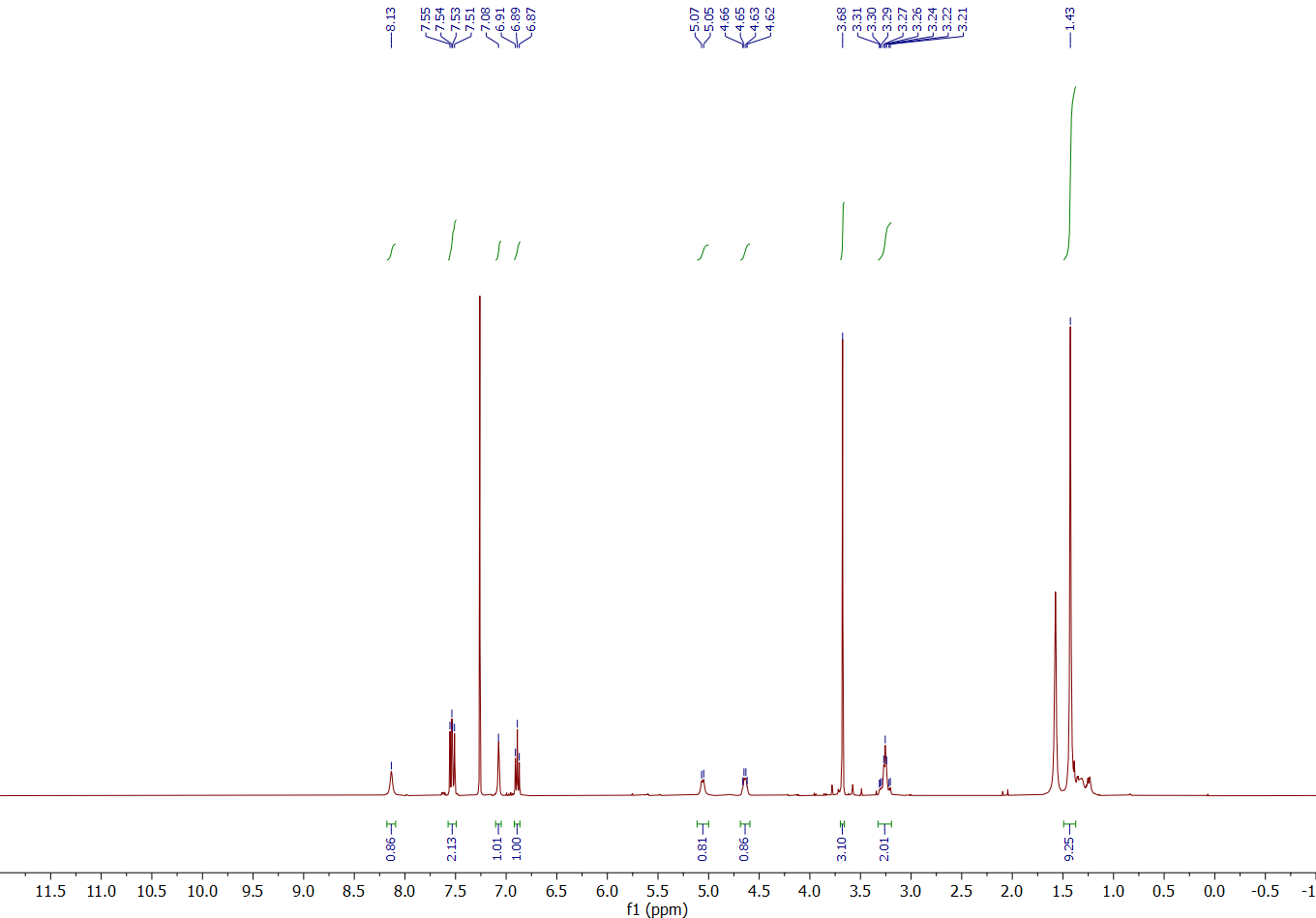


^13^C-NMR (101 MHz) in CDCl_3_


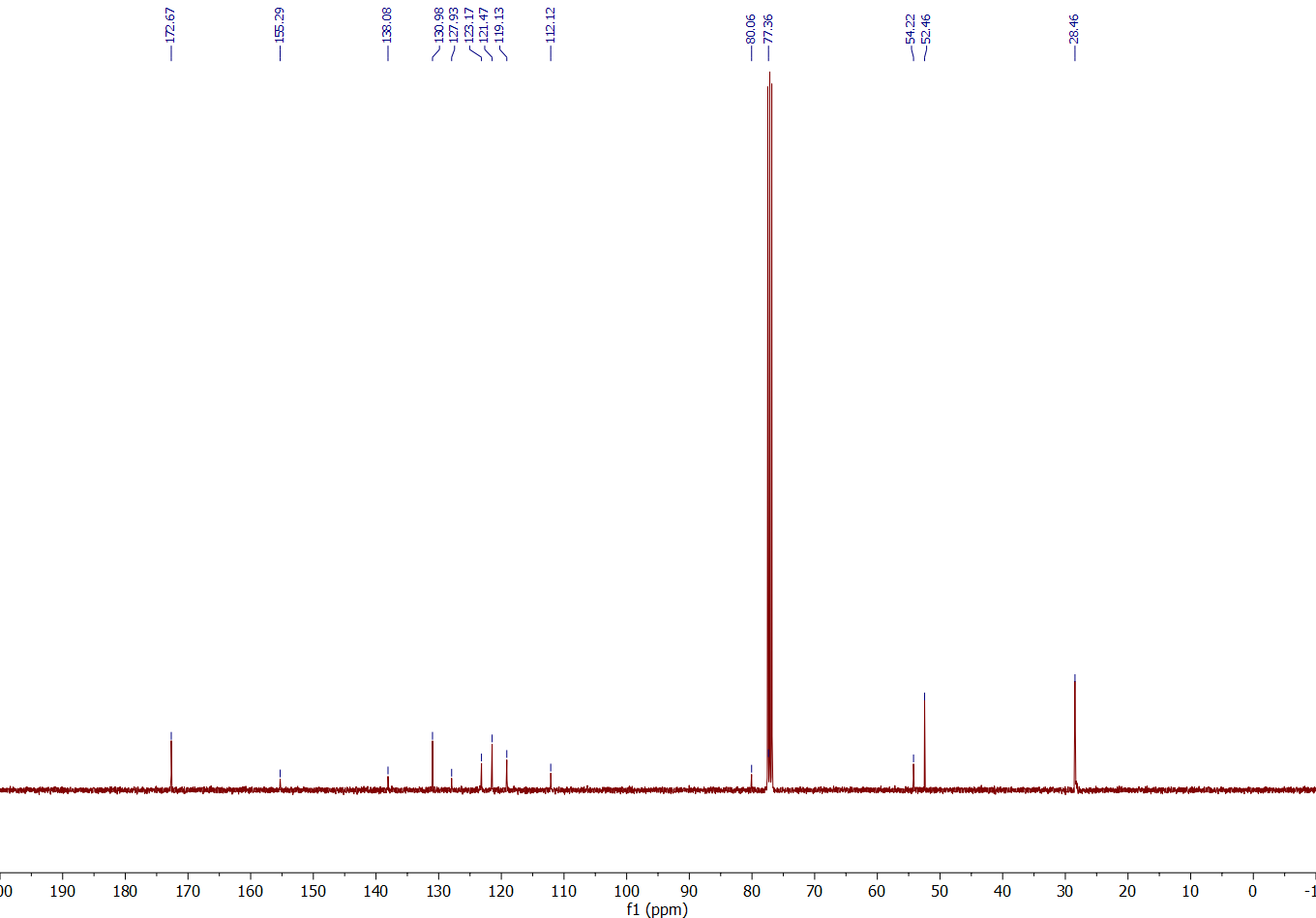


^1^H-NMR (400 MHz, 25 °C) in DMSO-*d_6_*


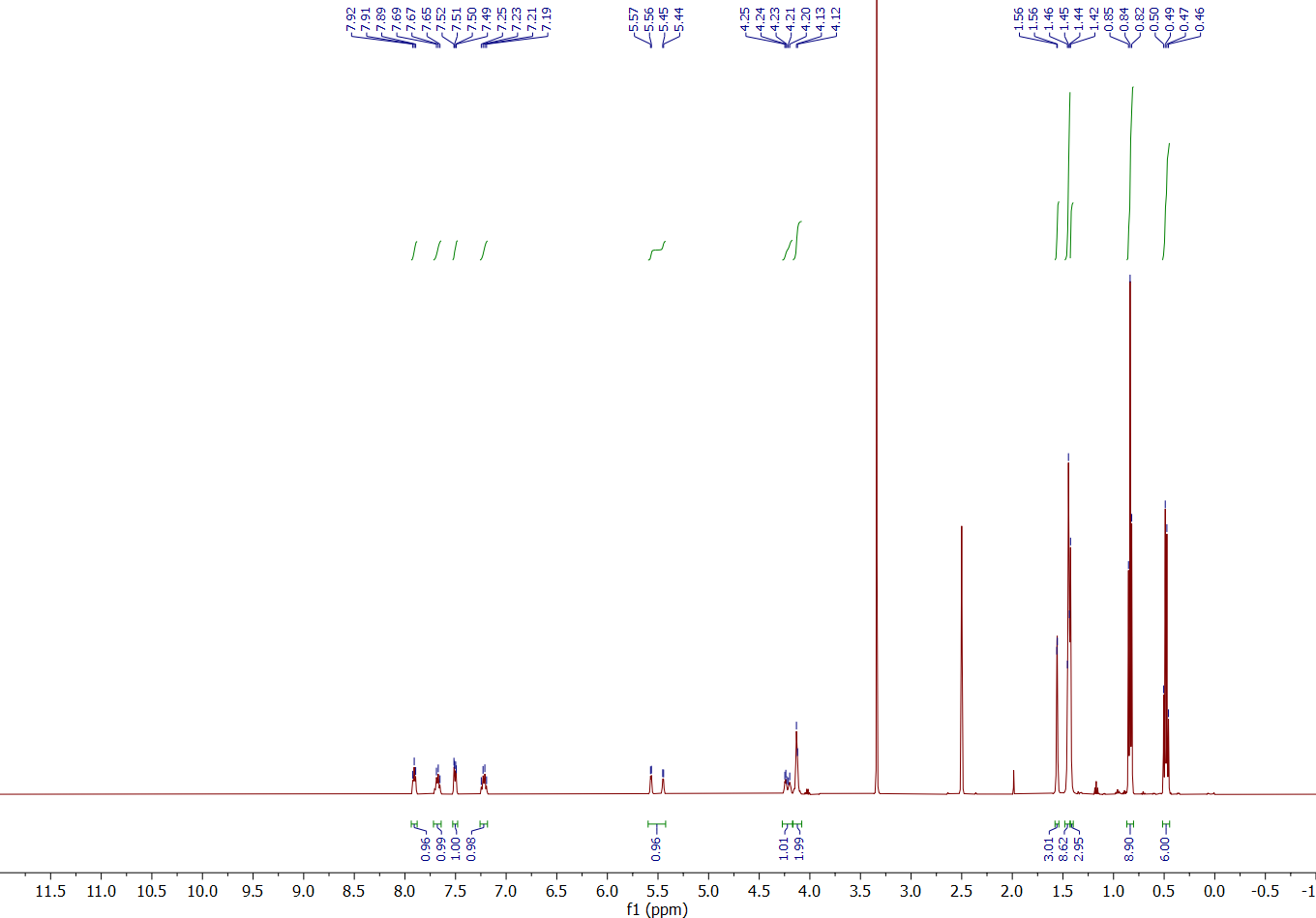


^1^H-NMR (400 MHz, 80 °C) in DMSO-*d_6_*


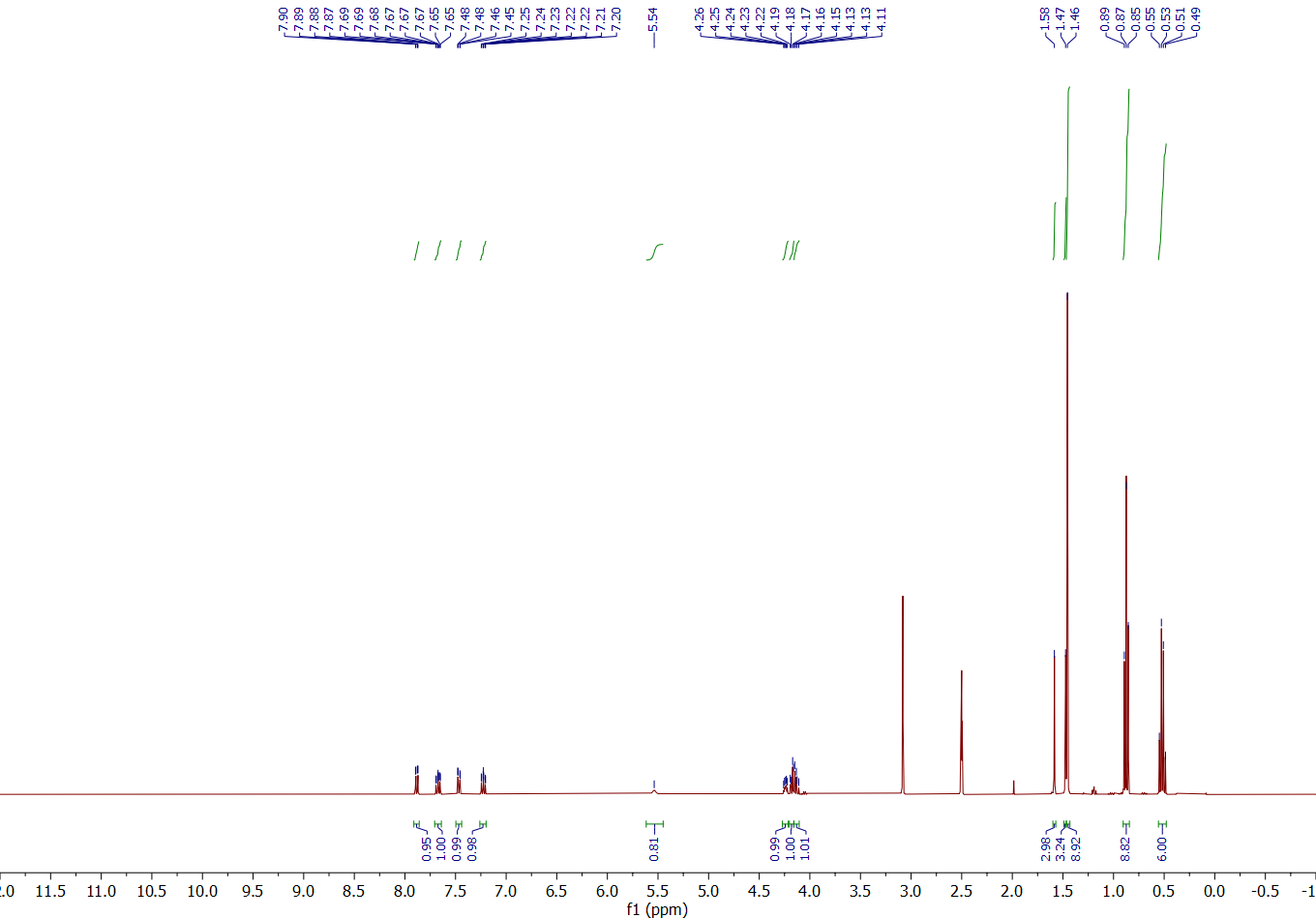


^13^C-NMR (101 MHz, 25 °C) in DMSO-*d_6_*


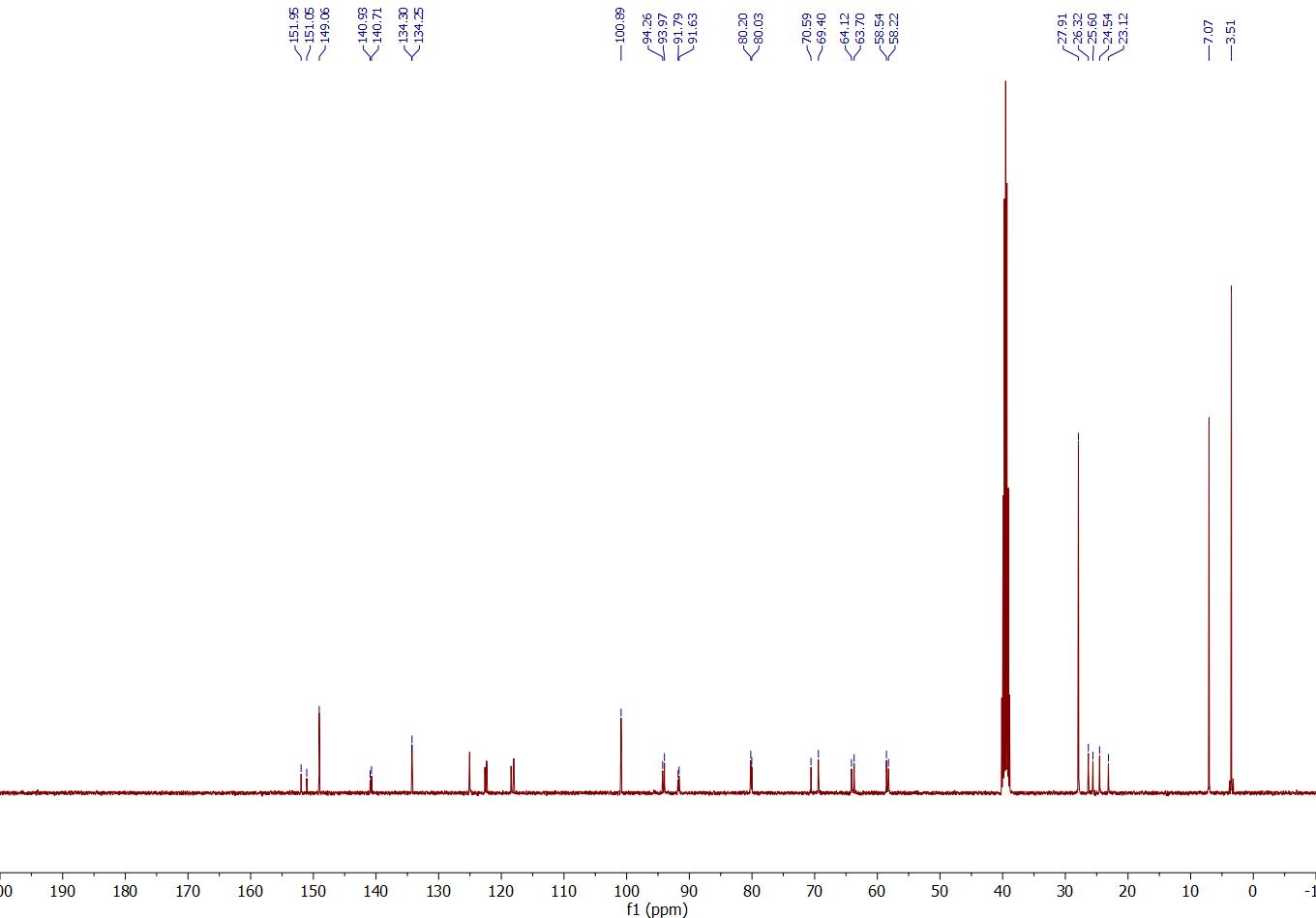


^13^C-NMR (101 MHz, 80 °C) in DMSO-*d_6_*


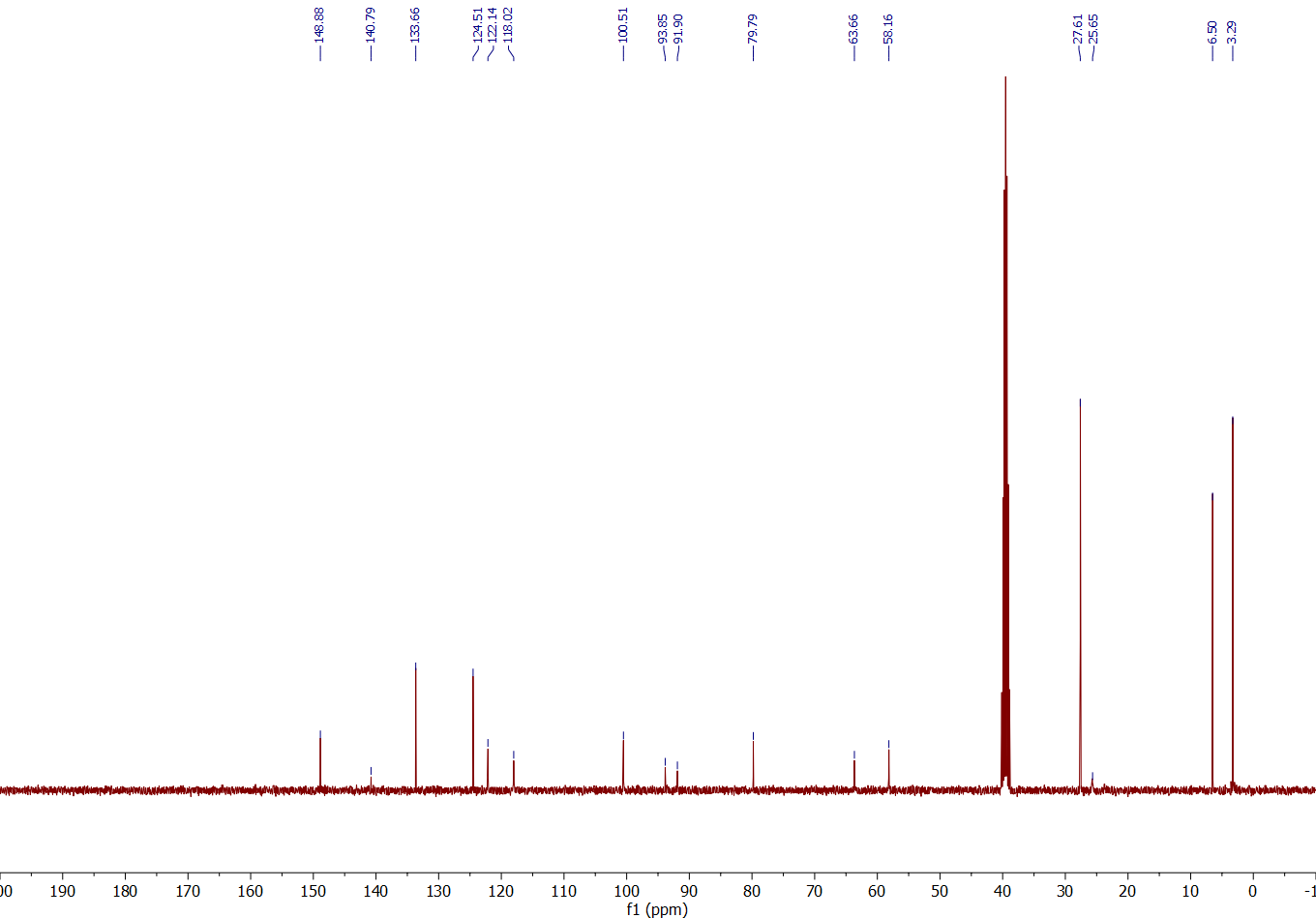


^1^H-NMR (500 MHz, 25 °C) in DMSO-*d_6_*


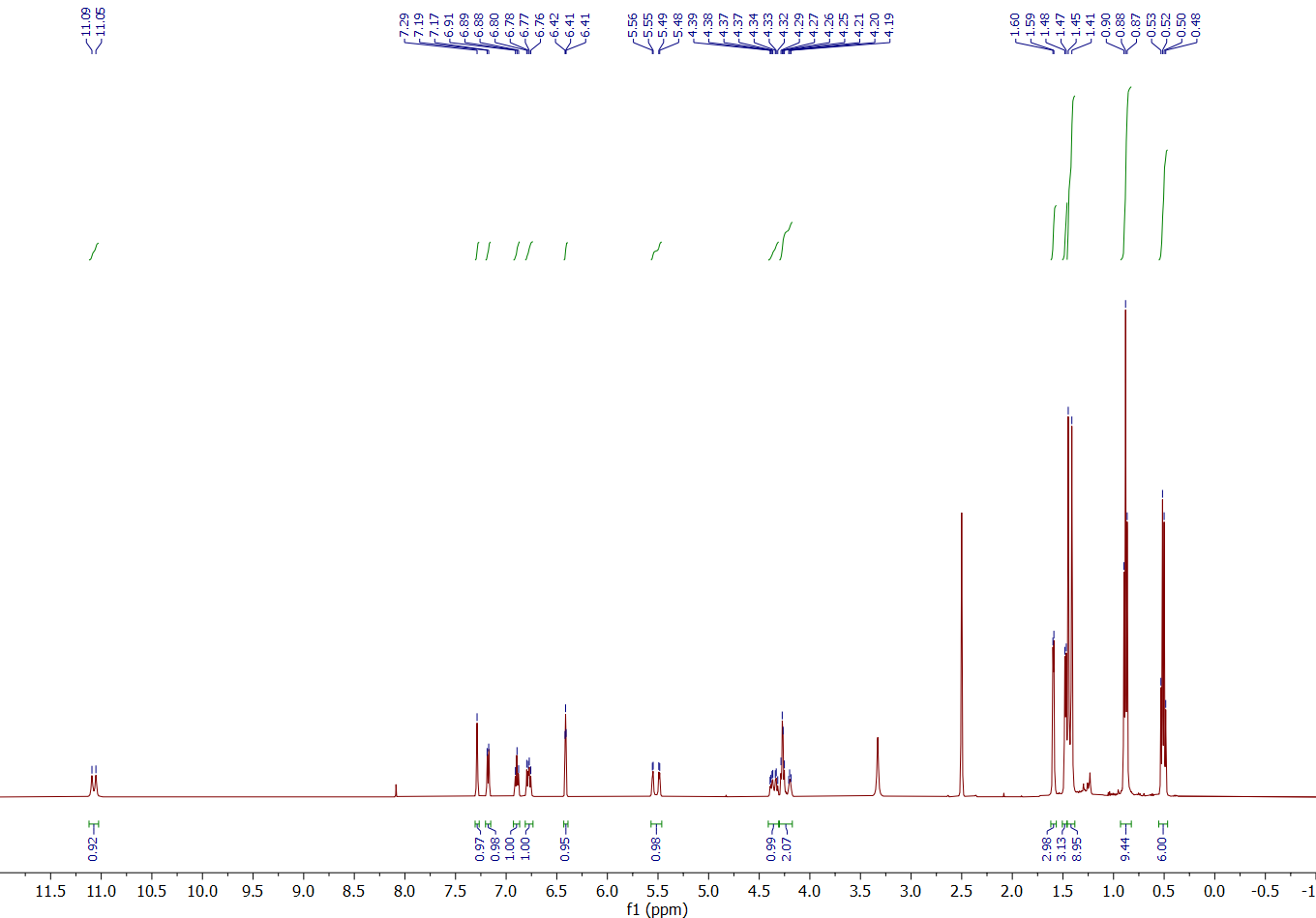


^1^H-NMR (500 MHz, 80 °C) in DMSO-*d_6_*


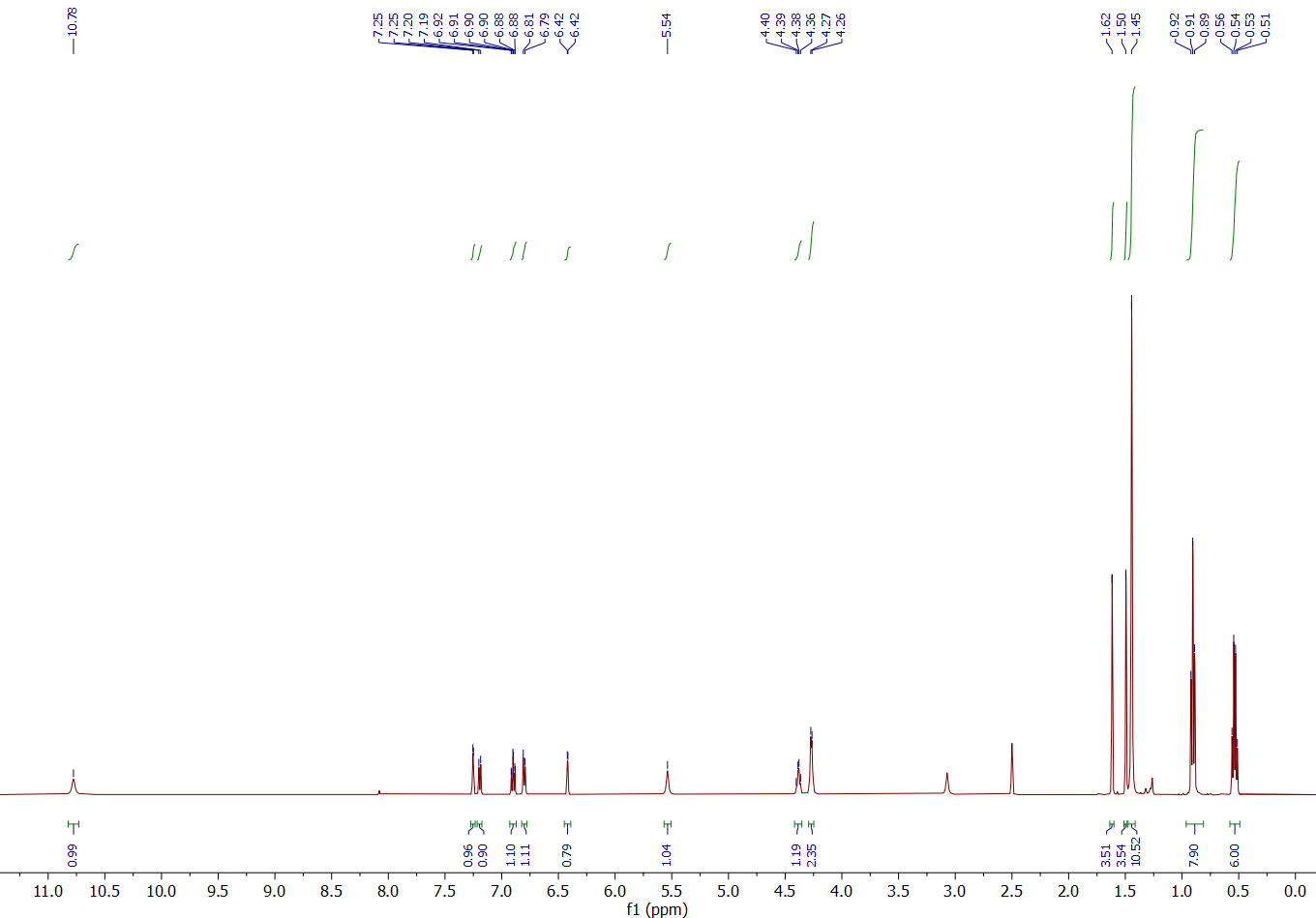


^13^C-NMR (126 MHz, 25 °C) in DMSO-*d_6_*


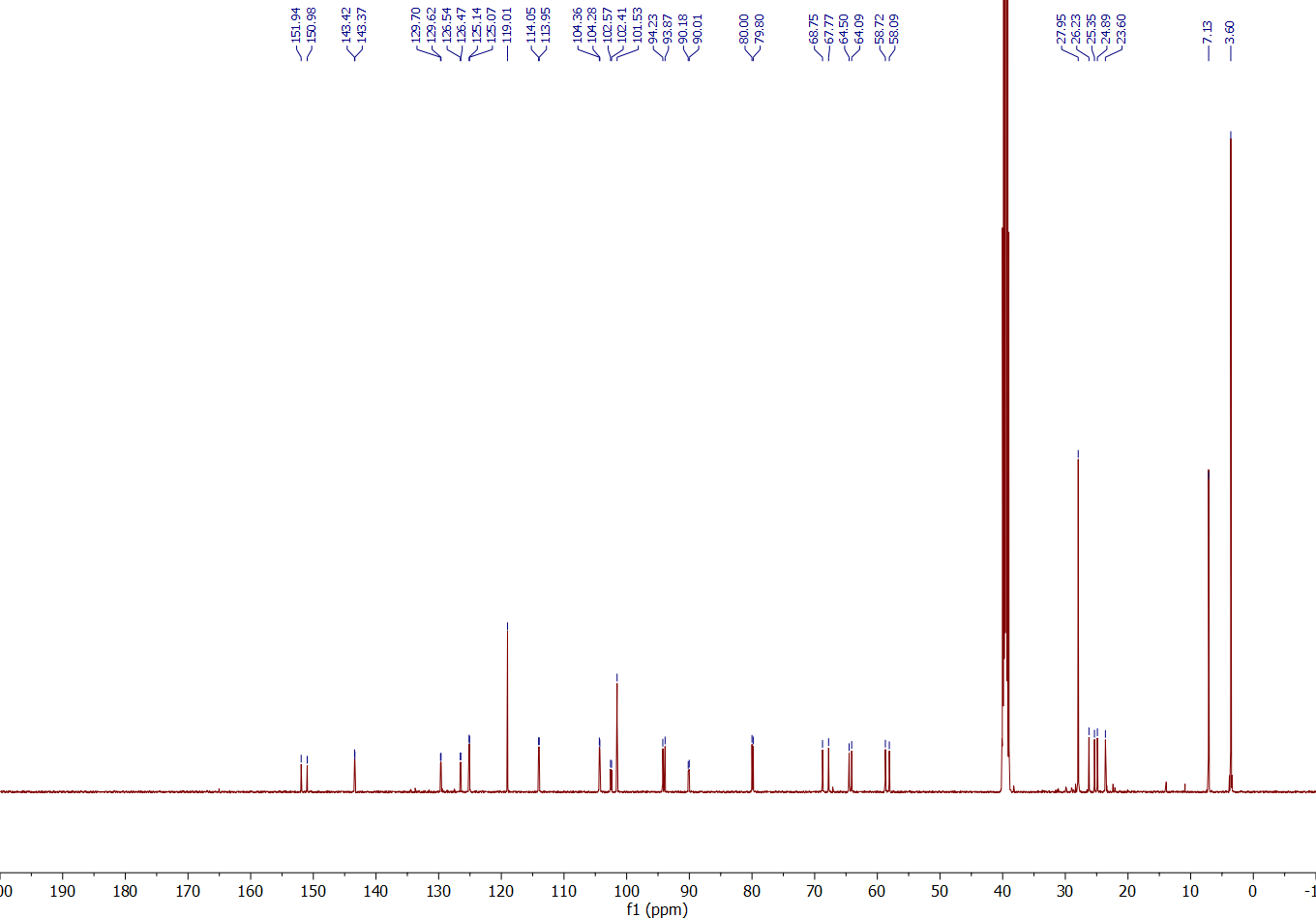


^13^C-NMR (126 MHz, 80 °C) in DMSO-*d_6_*


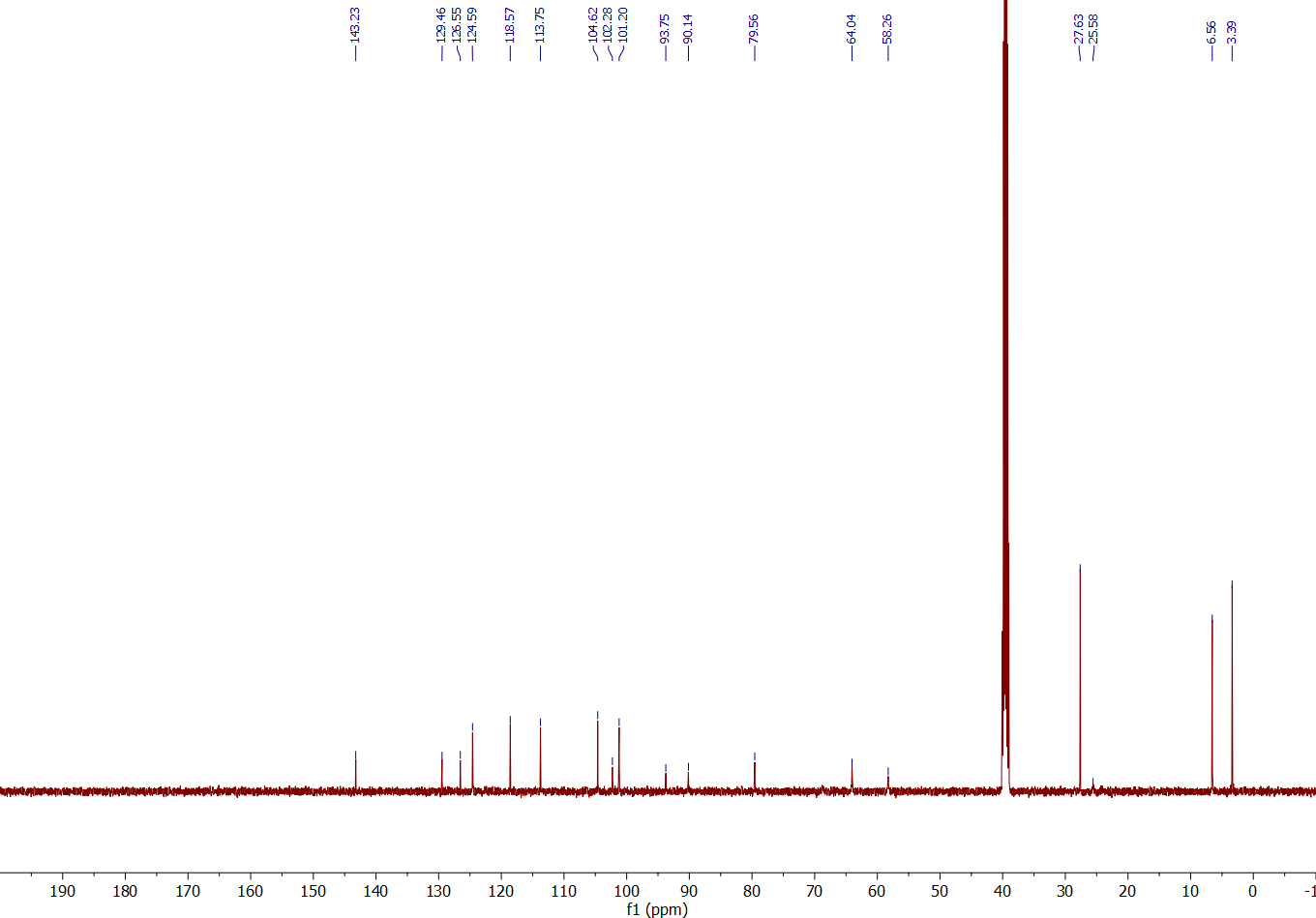


^1^H-NMR (400 MHz, 25 °C) in DMSO-*d_6_*


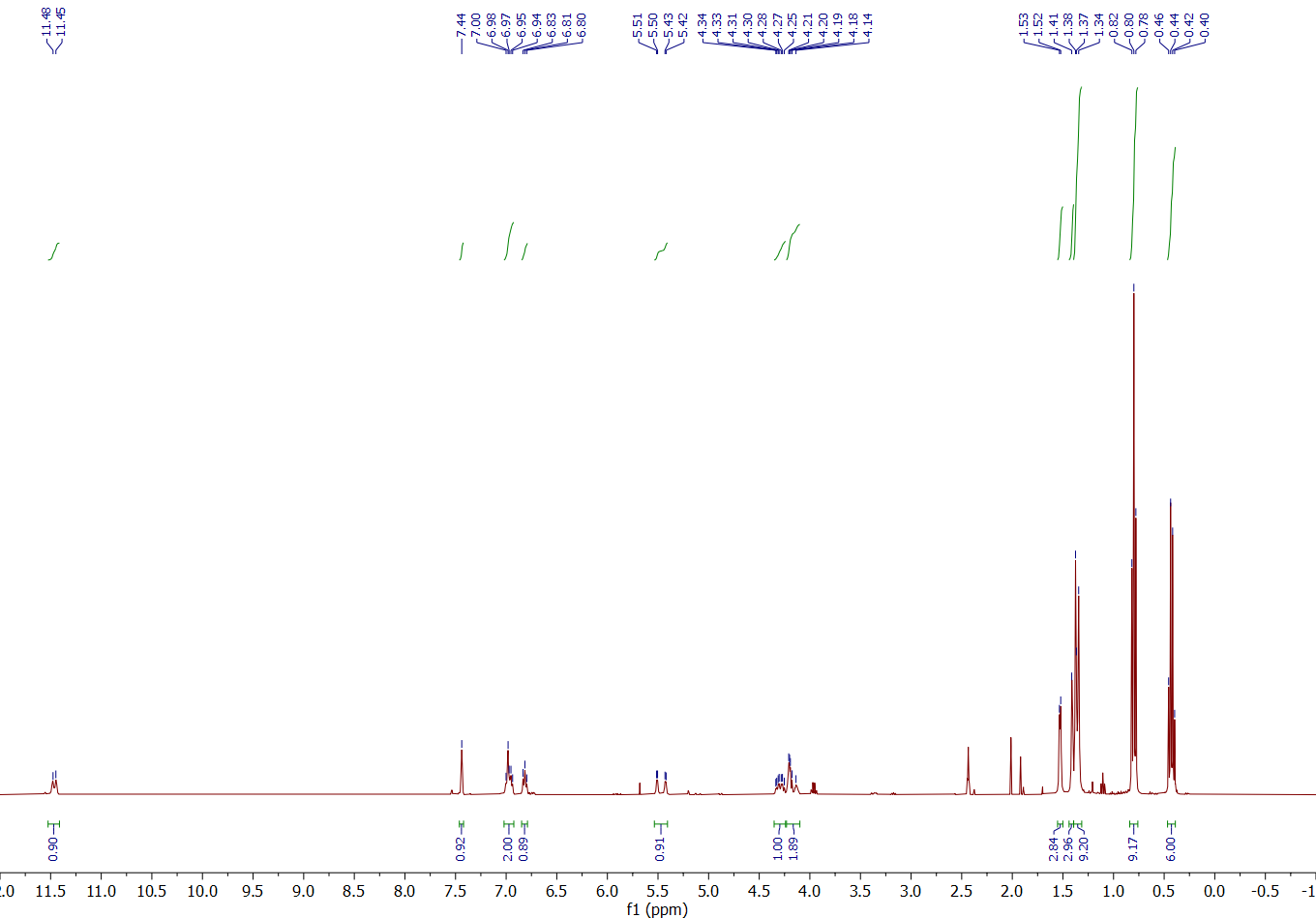


^1^H-NMR (400 MHz, 80 °C) in DMSO-*d_6_*


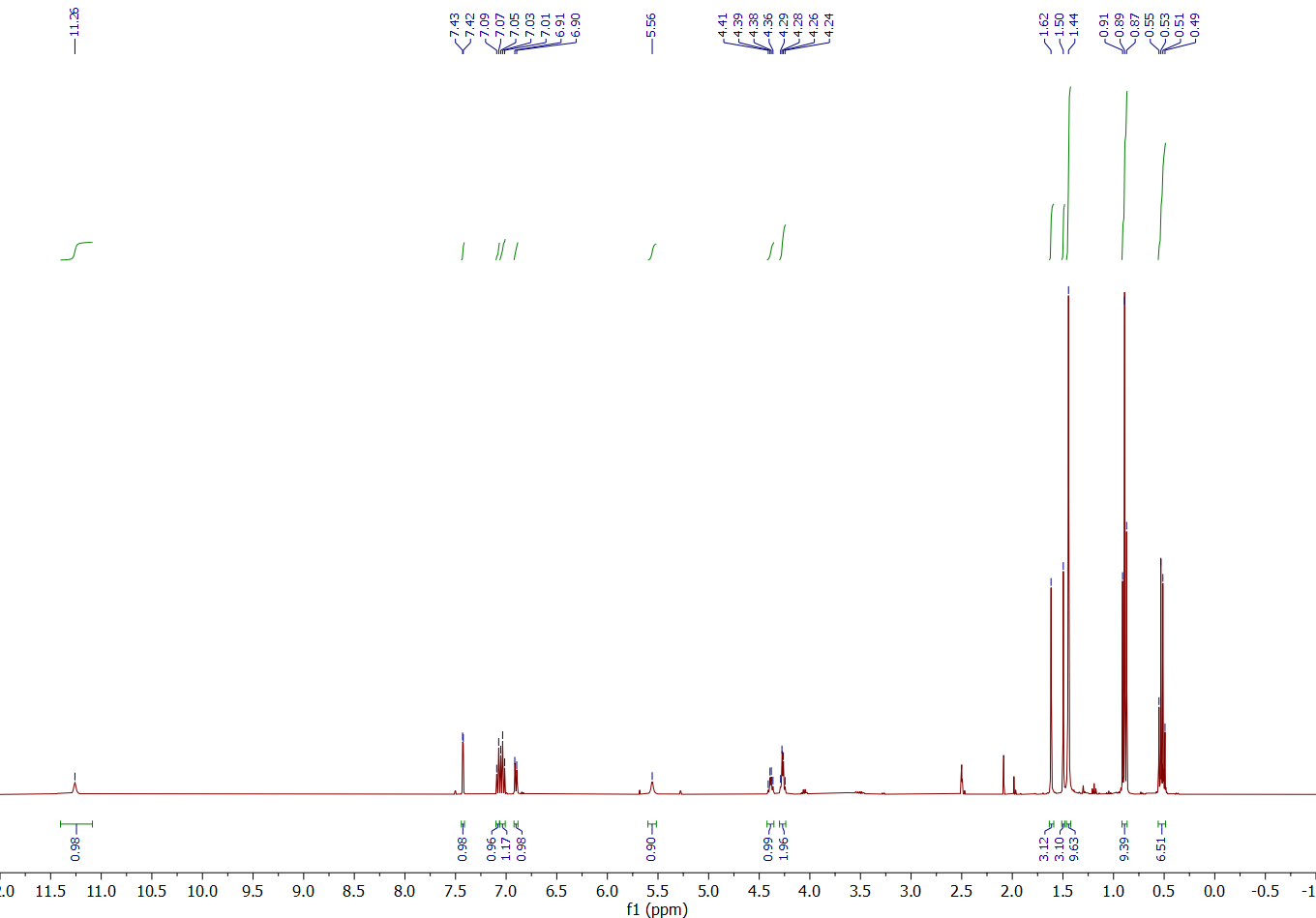


^13^C-NMR (101 MHz, 25 °C) in DMSO-*d_6_*


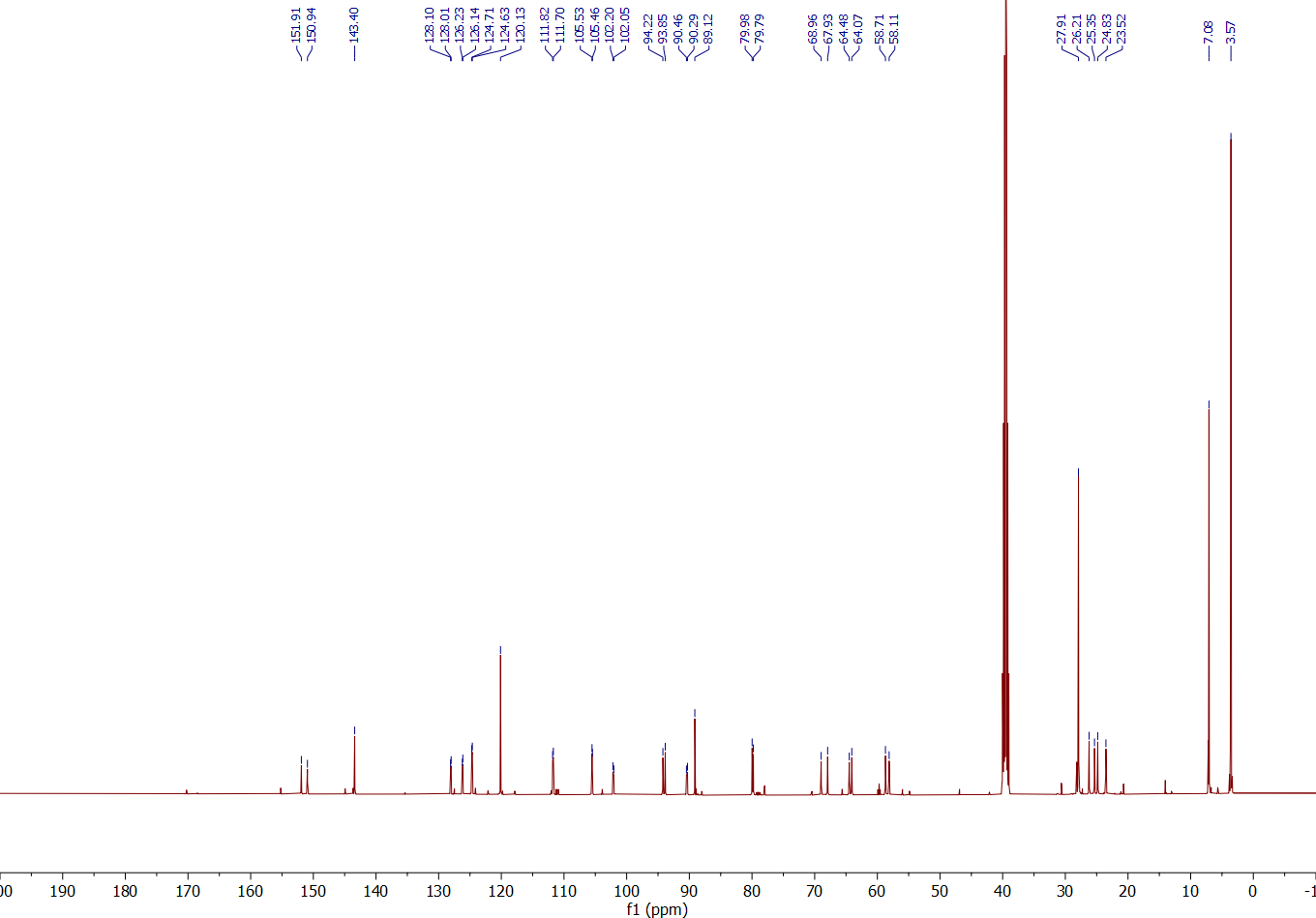


^13^C-NMR (101 MHz, 80 °C) in DMSO-*d_6_*


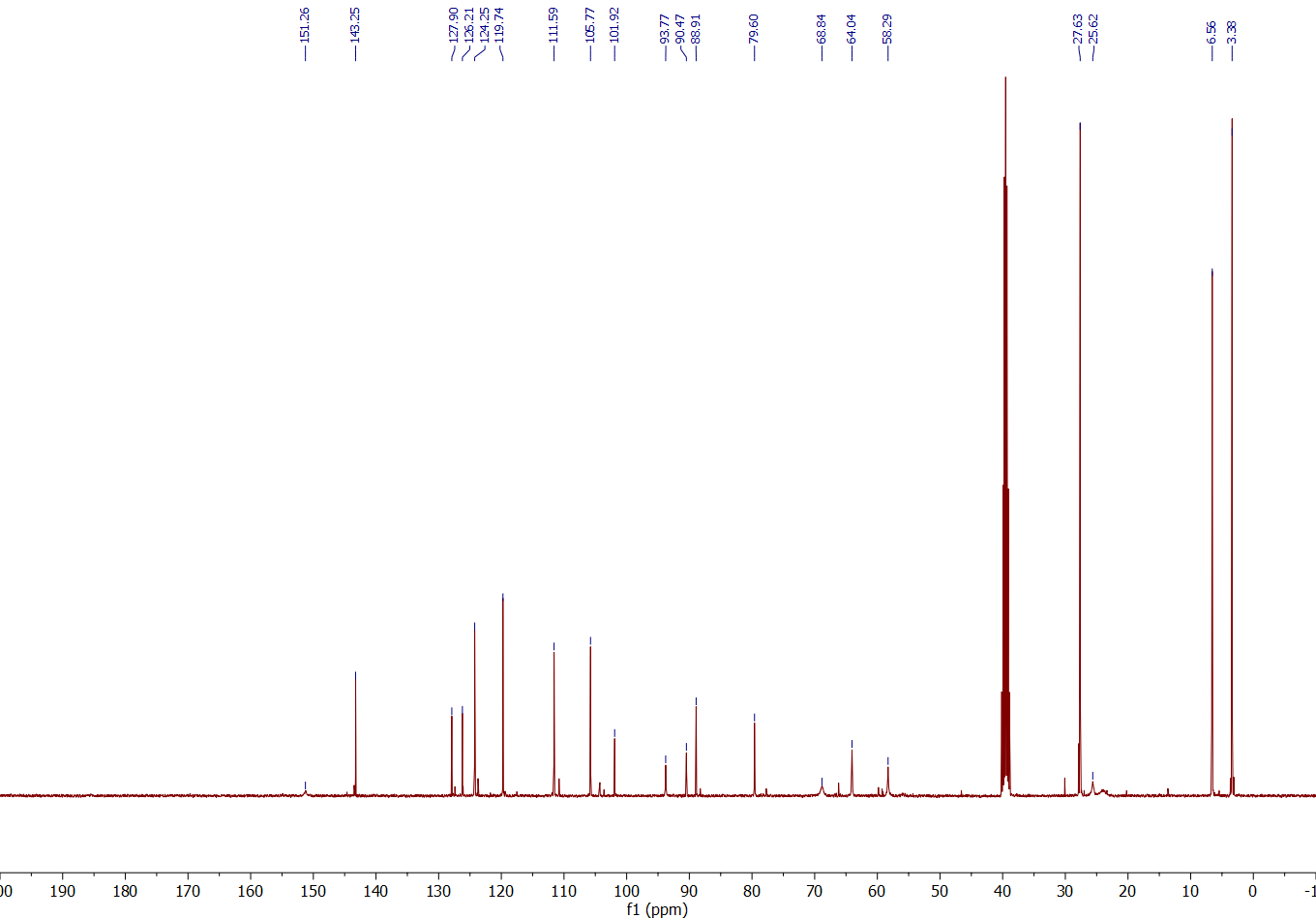


^1^H-NMR (400 MHz, 25 °C) in DMSO-*d_6_*


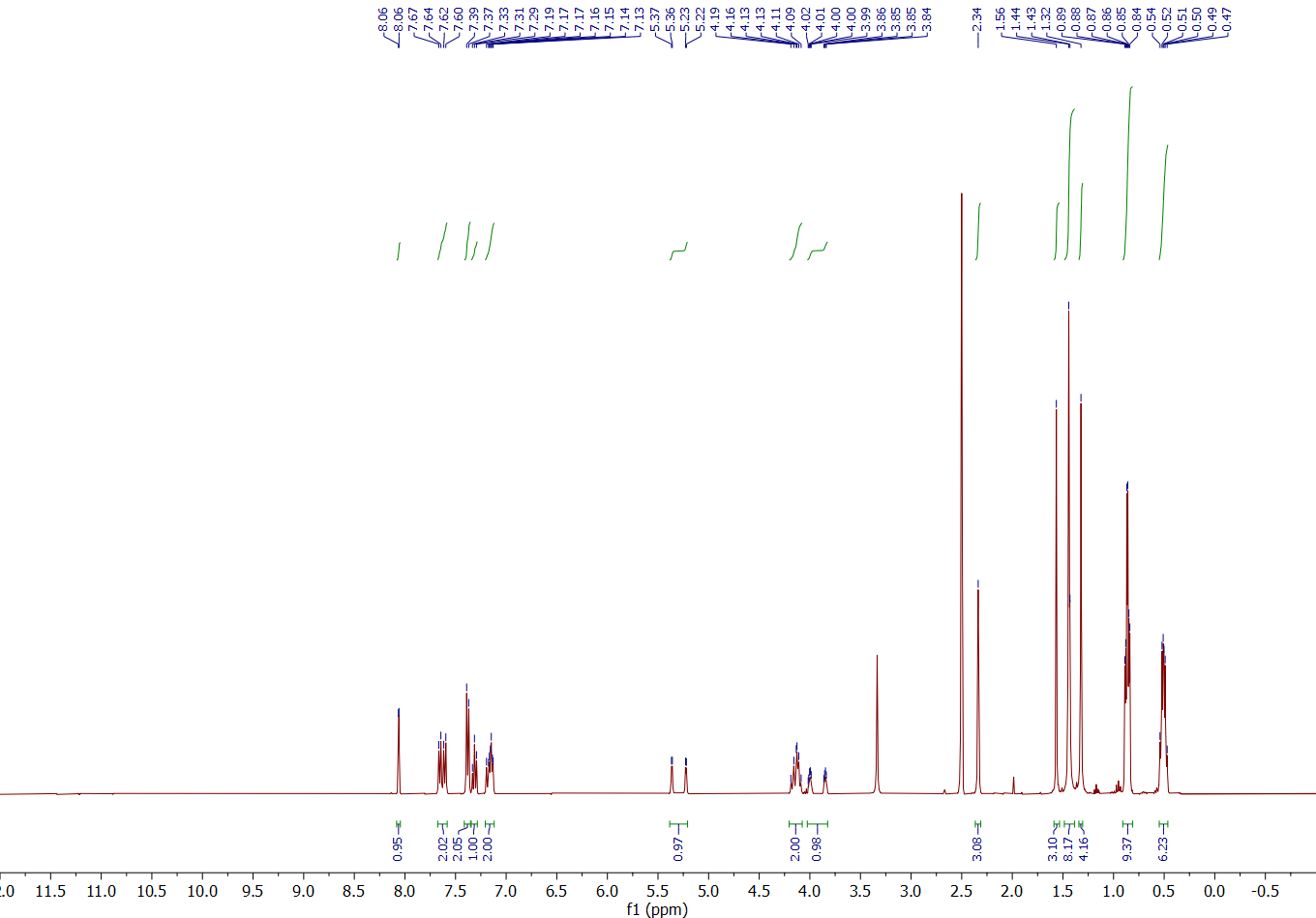


^1^H-NMR (400 MHz, 80 °C) in DMSO-*d_6_*


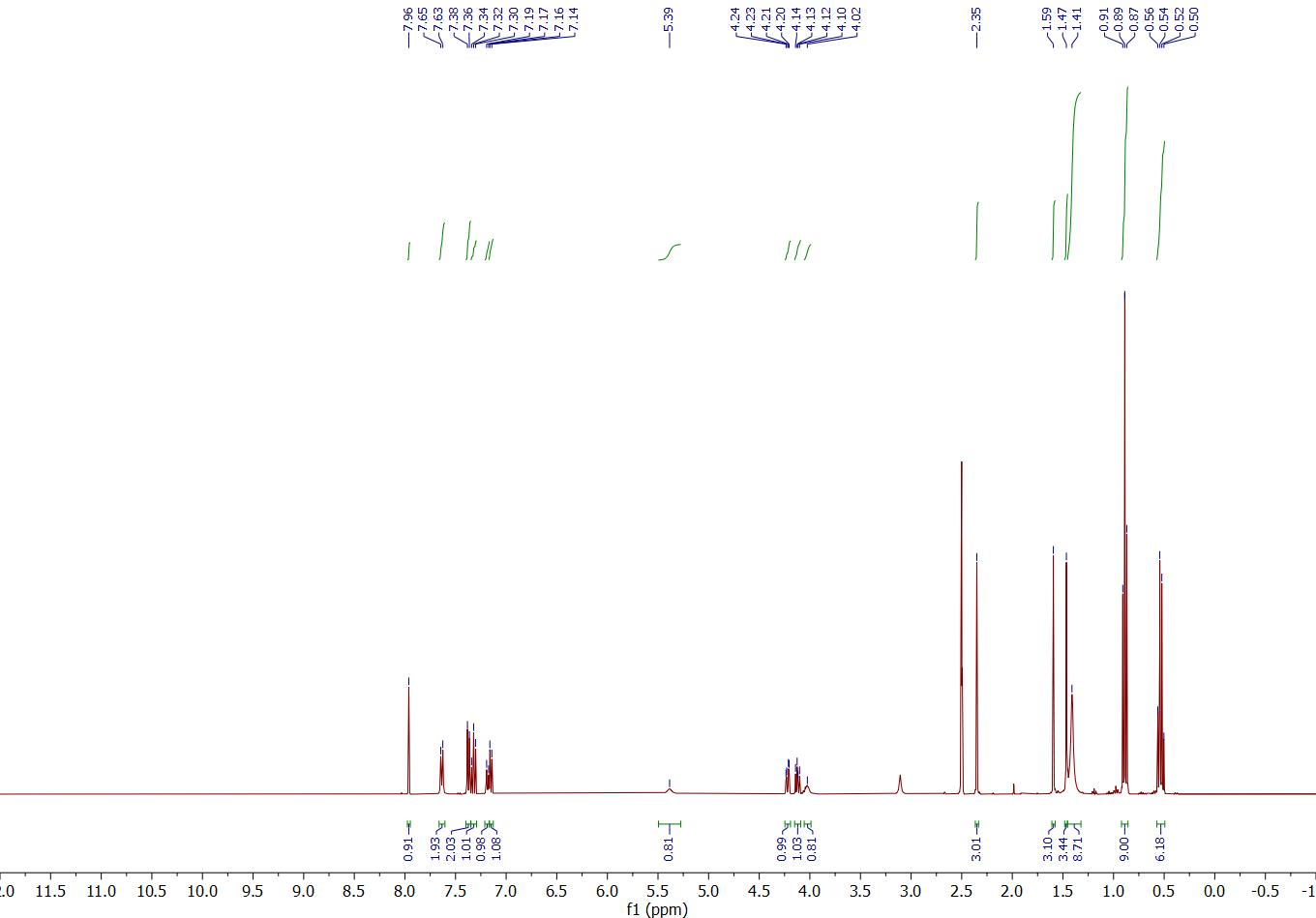


^13^C-NMR (126 MHz, 25 °C) in DMSO-*d_6_*


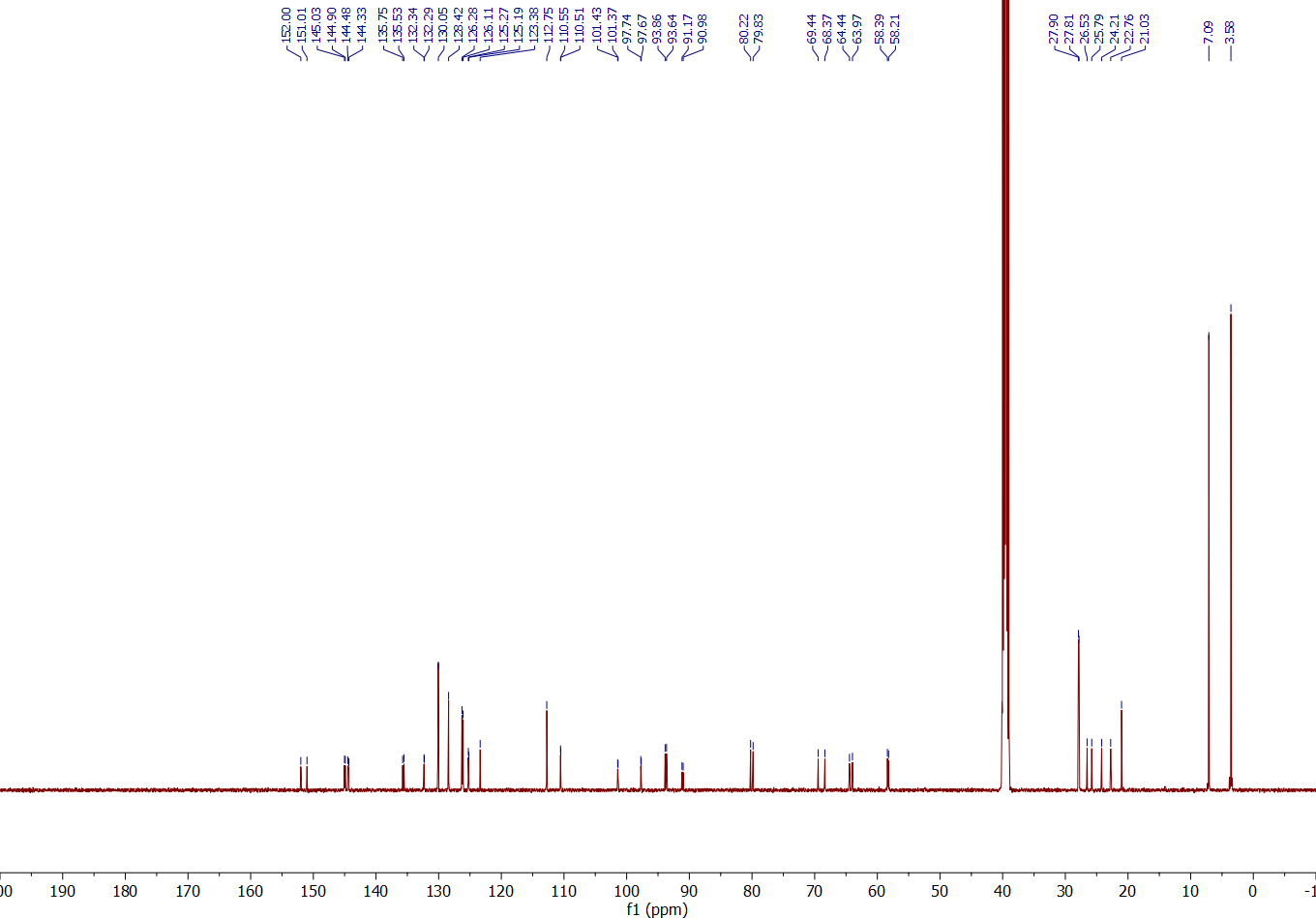


^13^C-NMR (126 MHz, 80 °C) in DMSO-*d_6_*


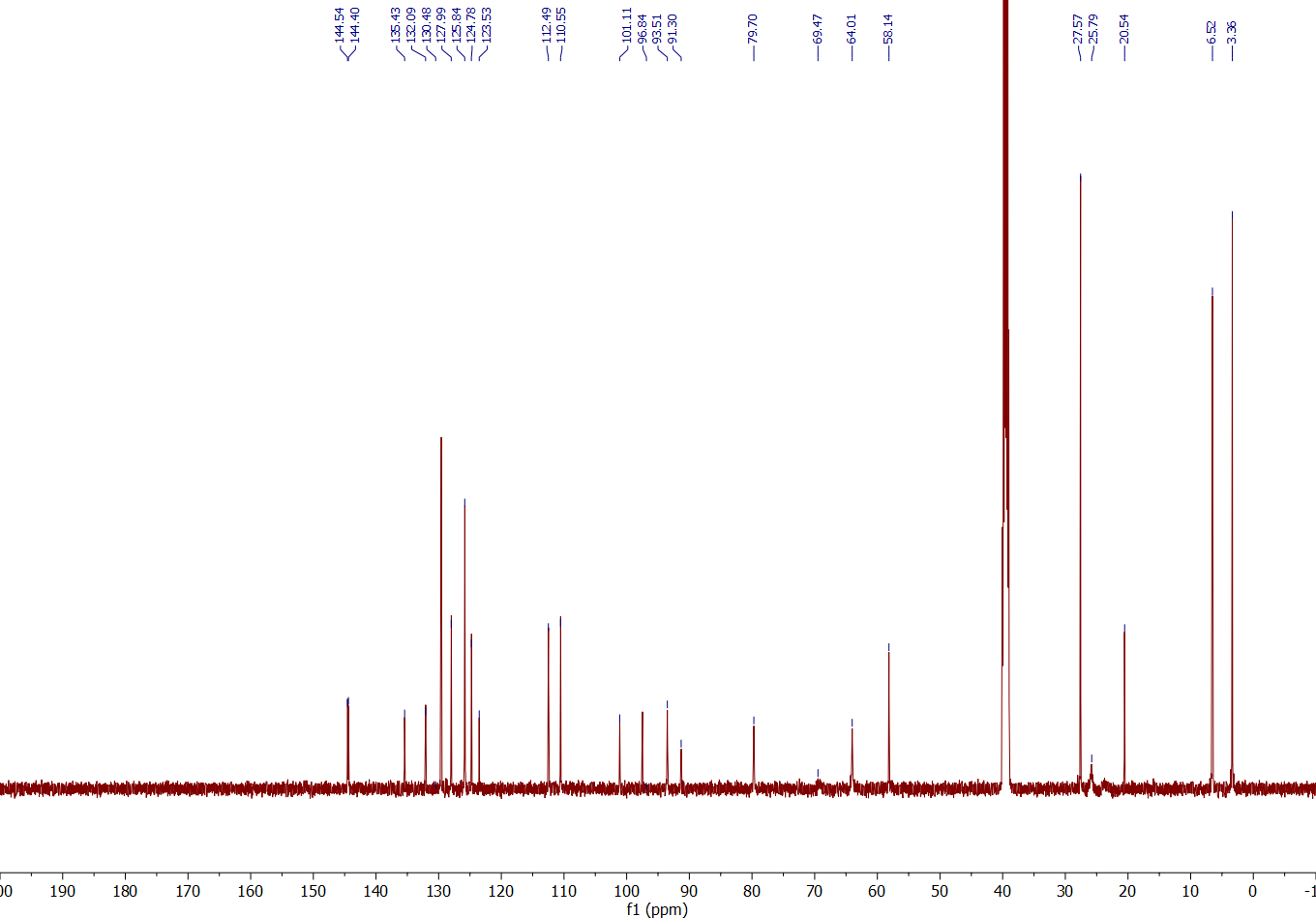


^1^H-NMR (500 MHz) in CDCl_3_


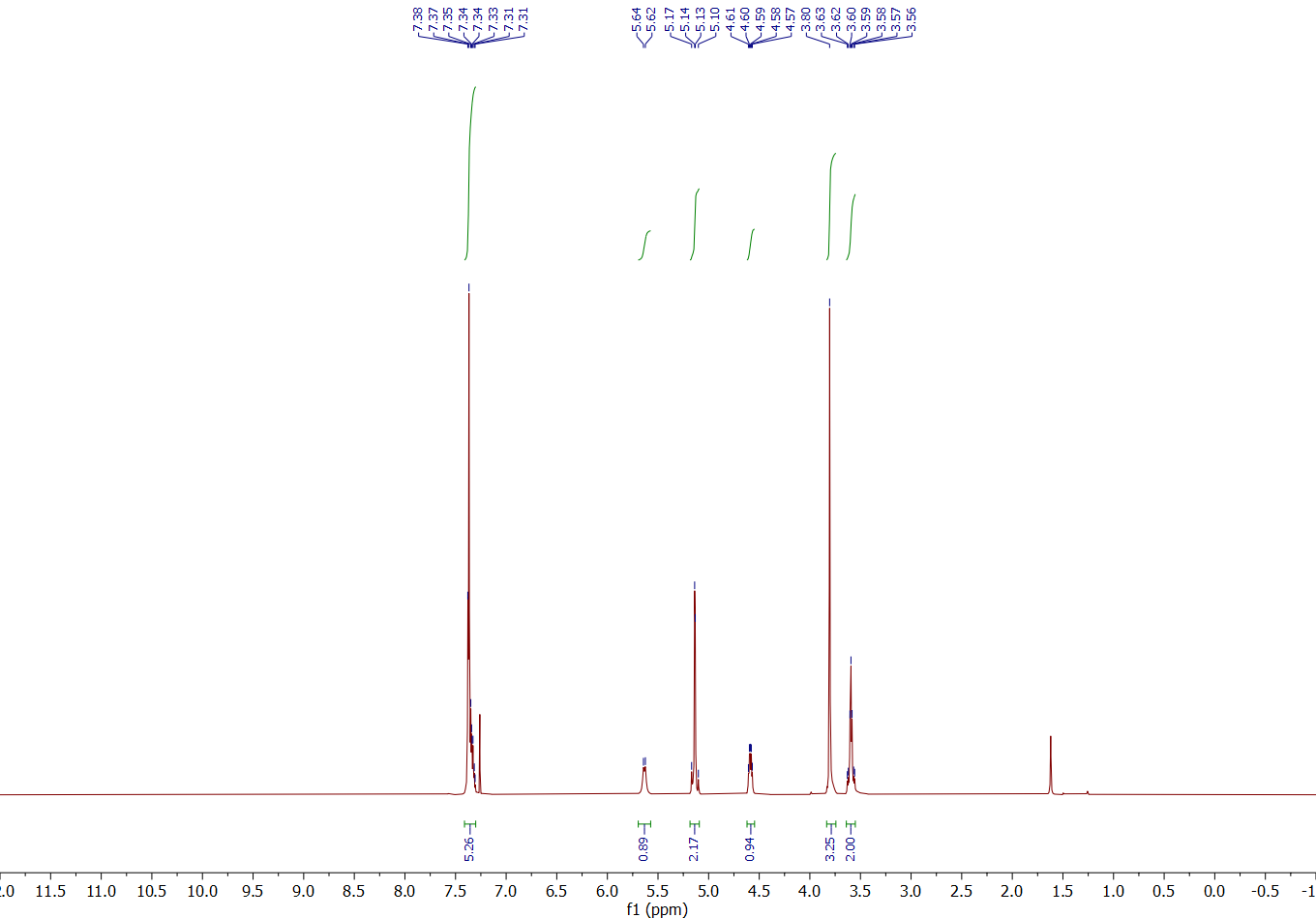


^13^C-NMR (126 MHz) in CDCl_3_


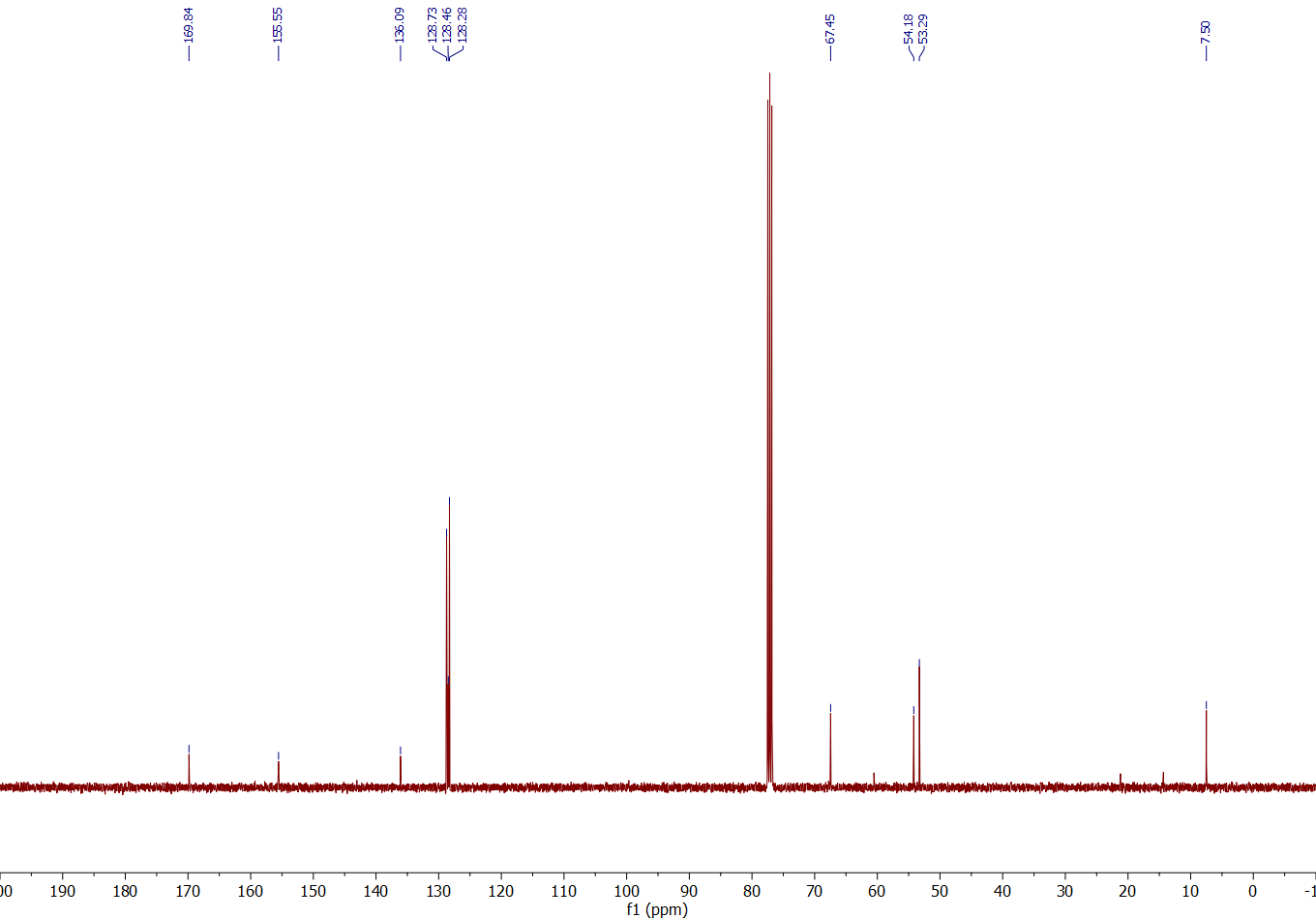


^1^H-NMR (500 MHz, 25 °C) in DMSO-*d_6_*


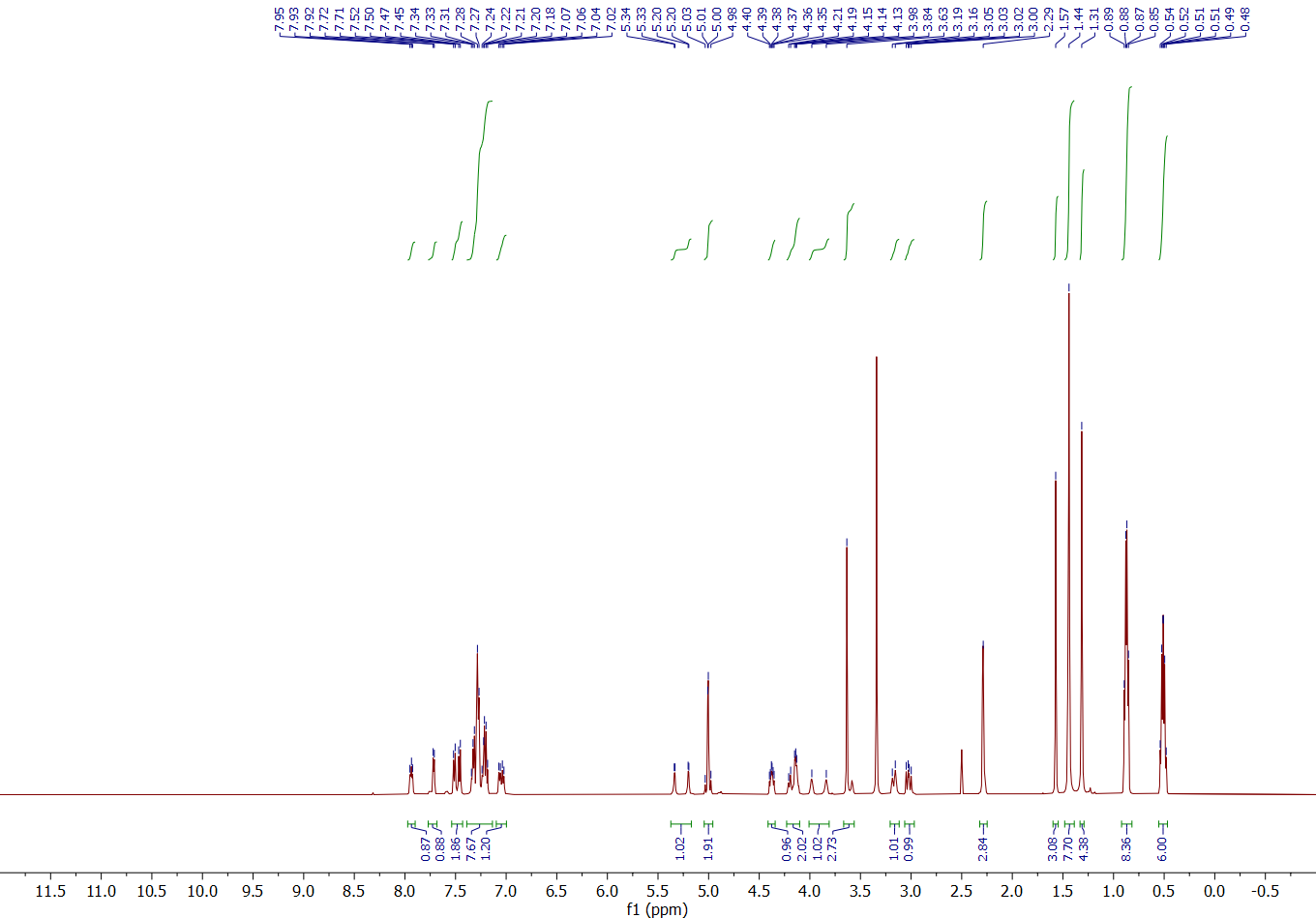


^1^H-NMR (500 MHz, 80 °C) in DMSO-*d_6_*


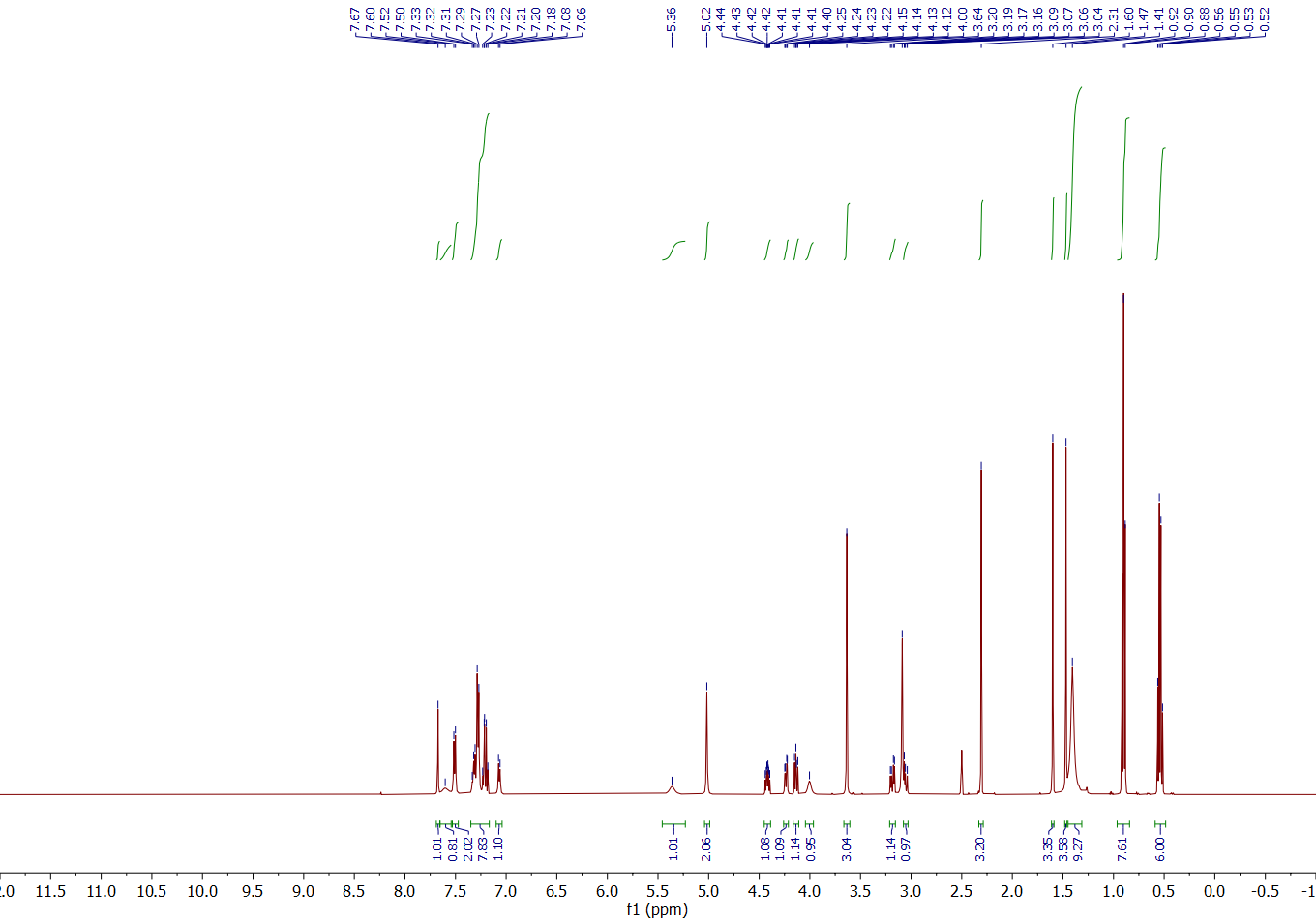


^13^C-NMR (126 MHz, 25 °C) in DMSO-*d_6_*


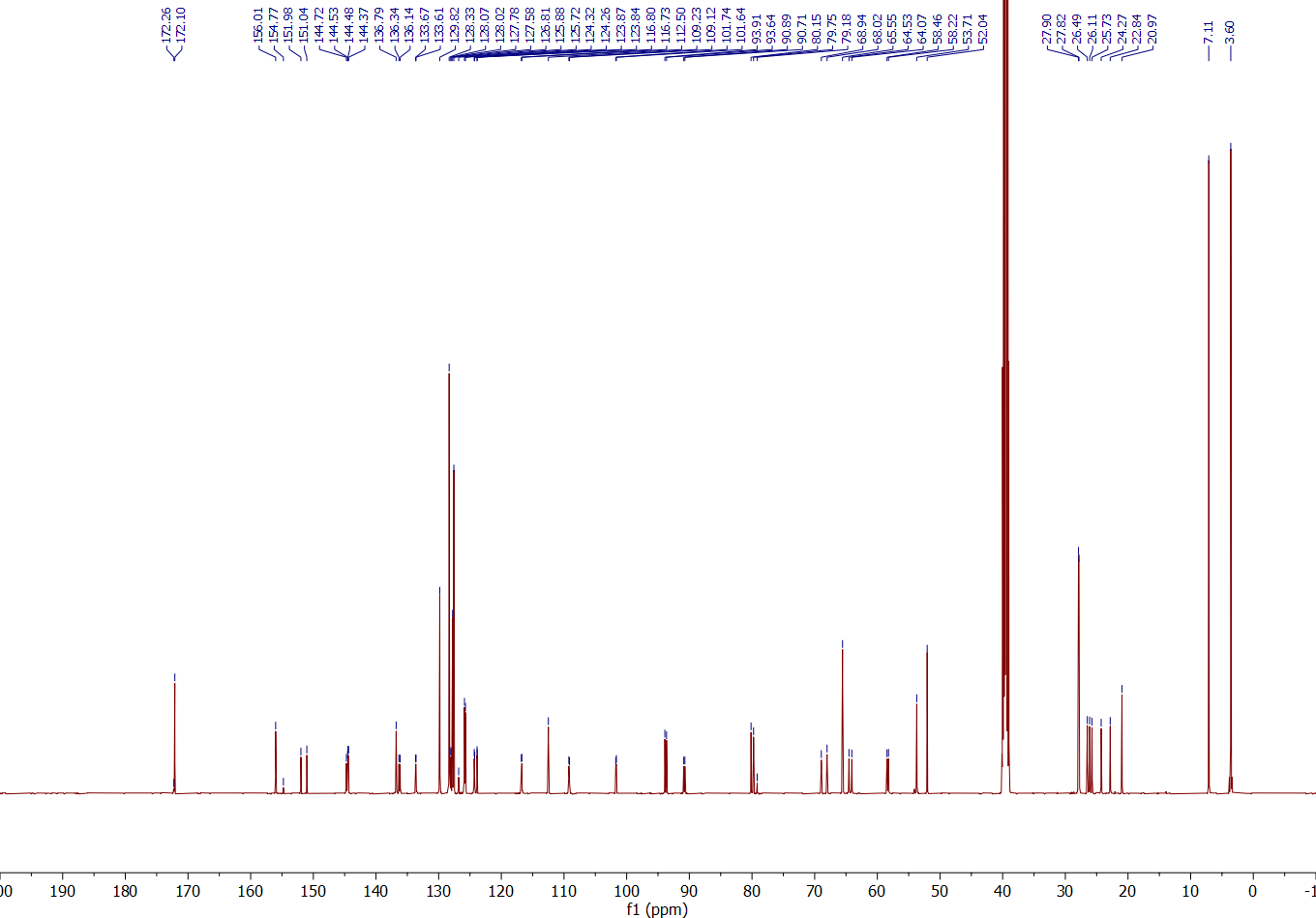


^13^C-NMR (126 MHz, 80 °C) in DMSO-*d_6_*


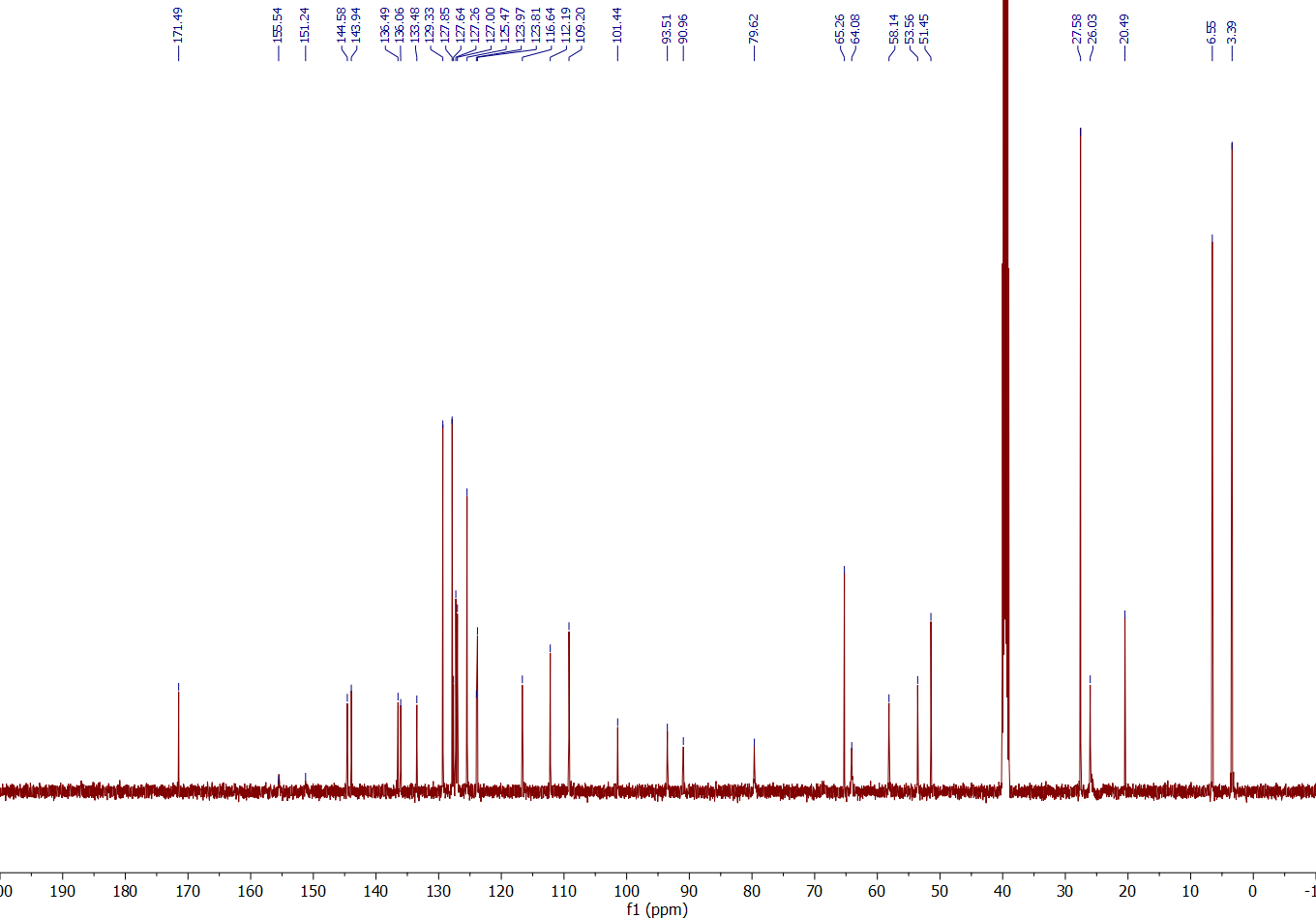


^1^H-NMR (500 MHz) in methanol-*d_4_*


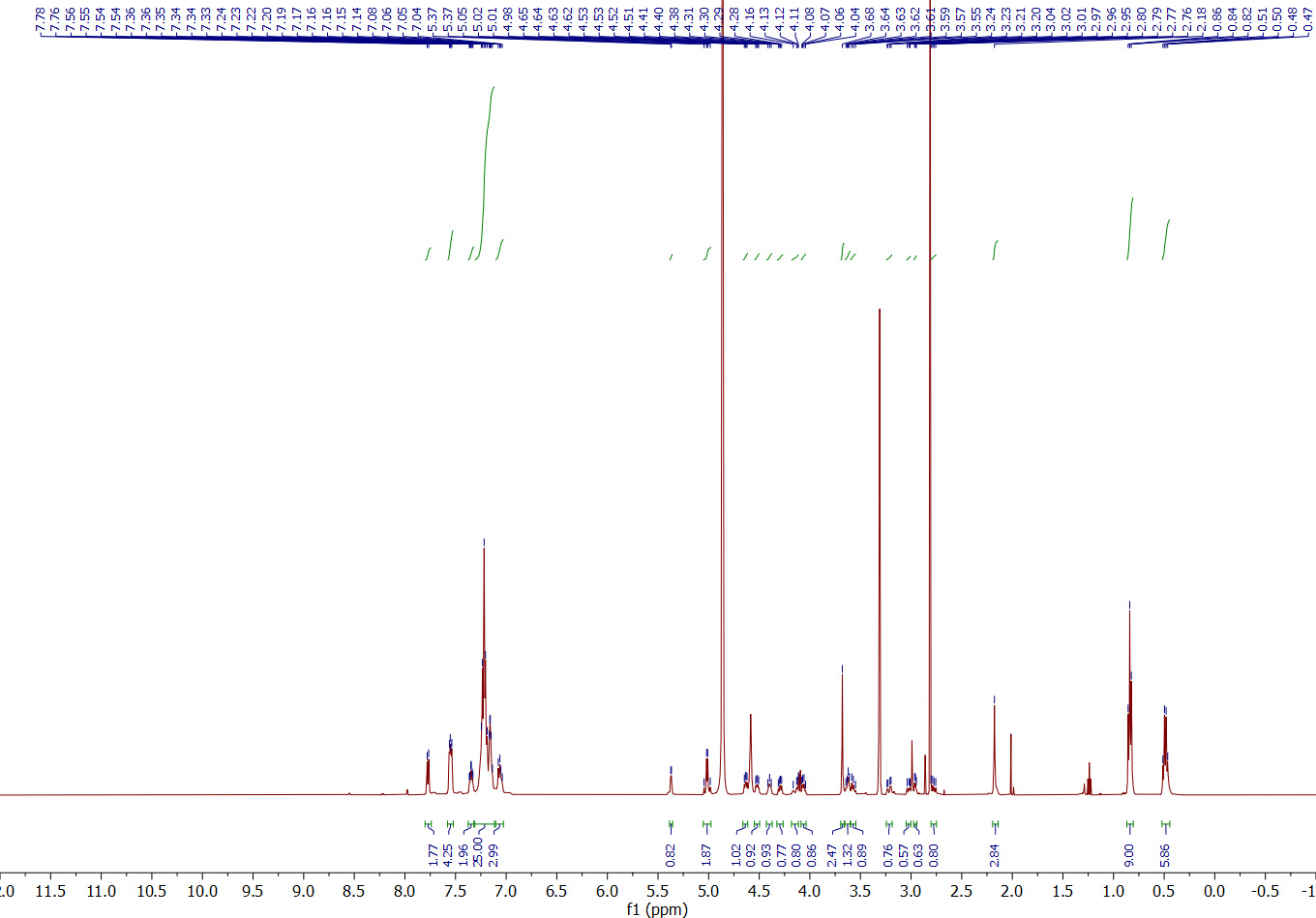


^13^C-NMR (126 MHz) in methanol-*d_4_*


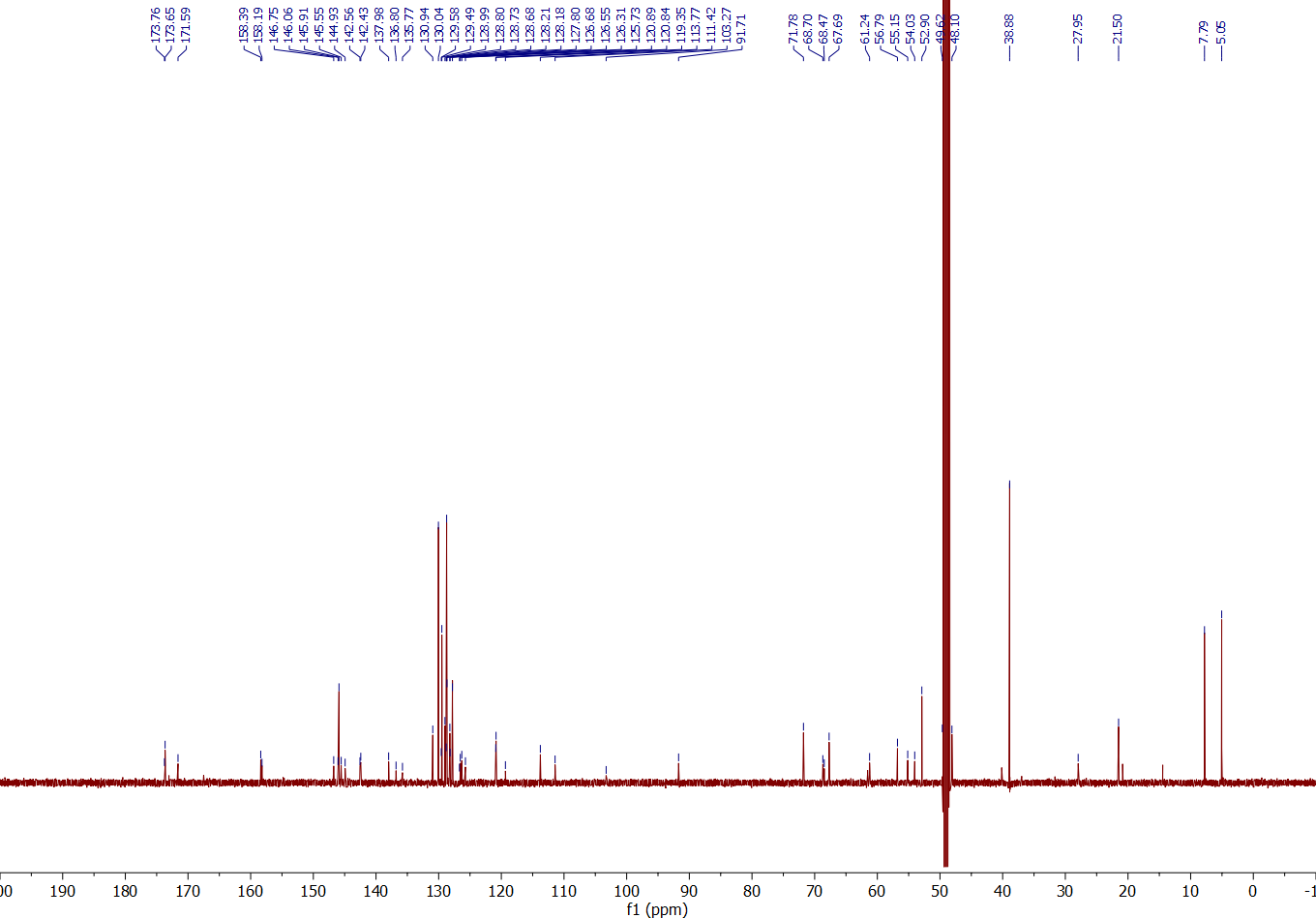


^1^H-NMR (500 MHz) in methanol-*d_4_*


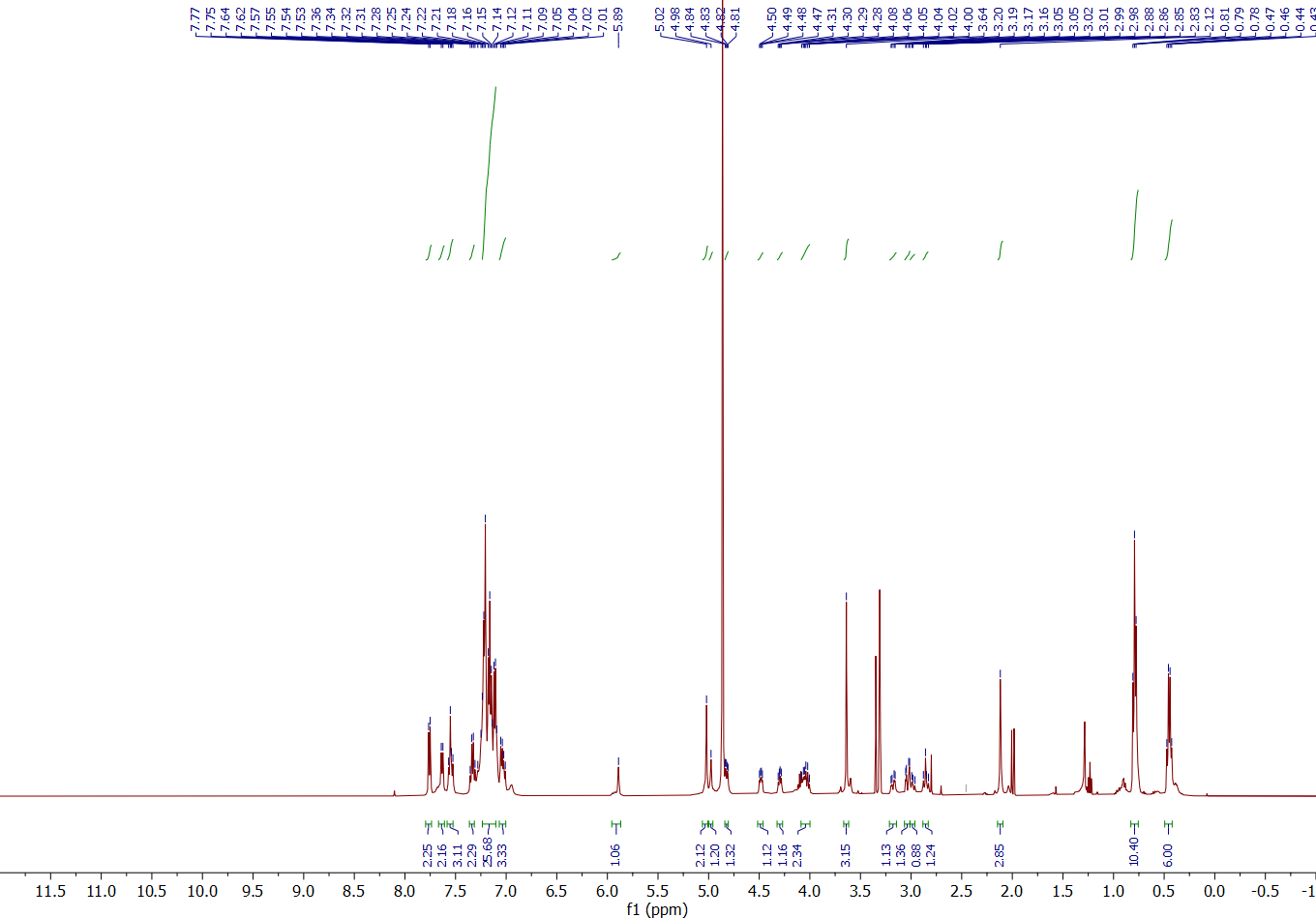


^13^C-NMR (126 MHz) in methanol-*d_4_*


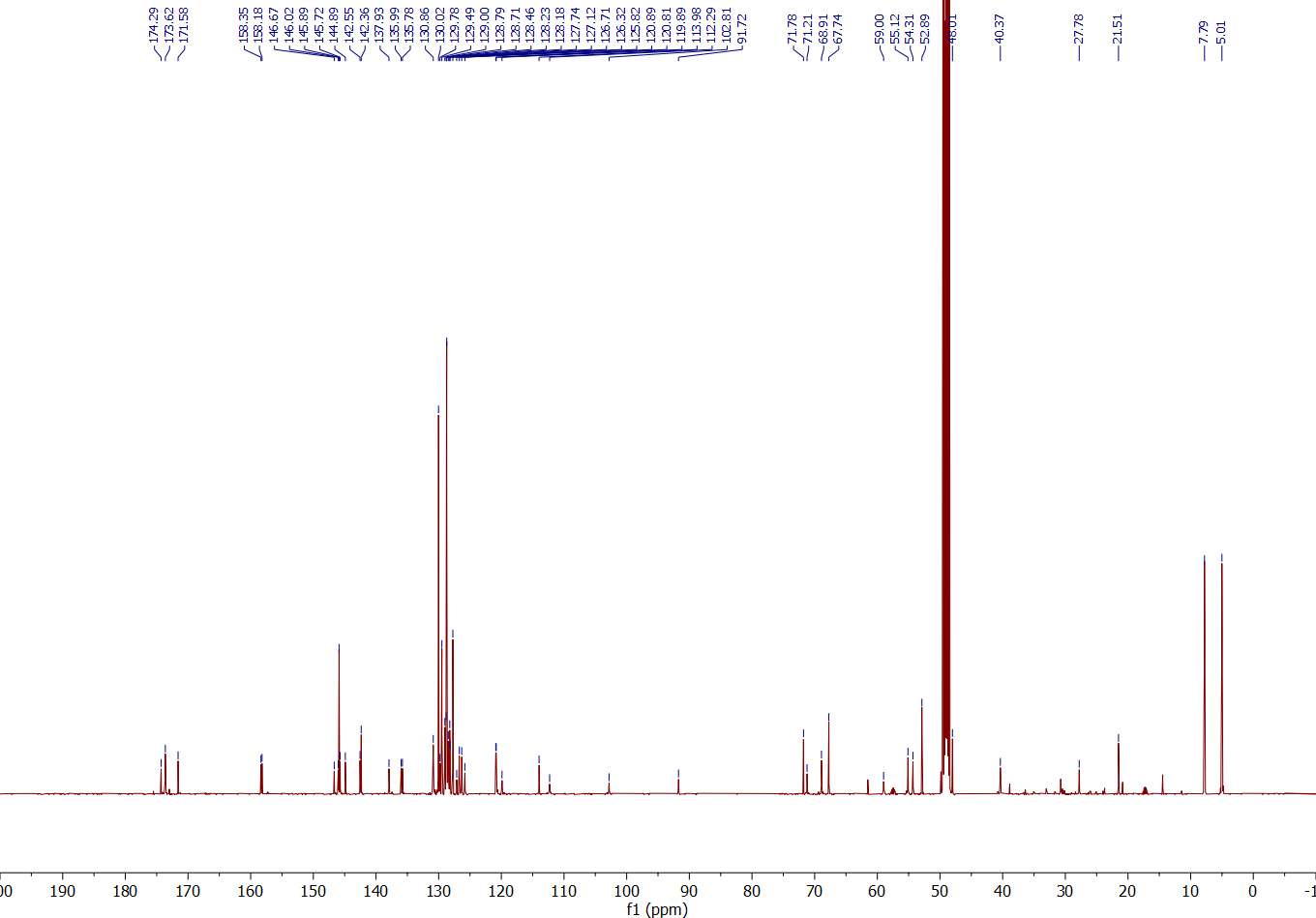


^1^H-NMR (400 MHz) in CDCl_3_


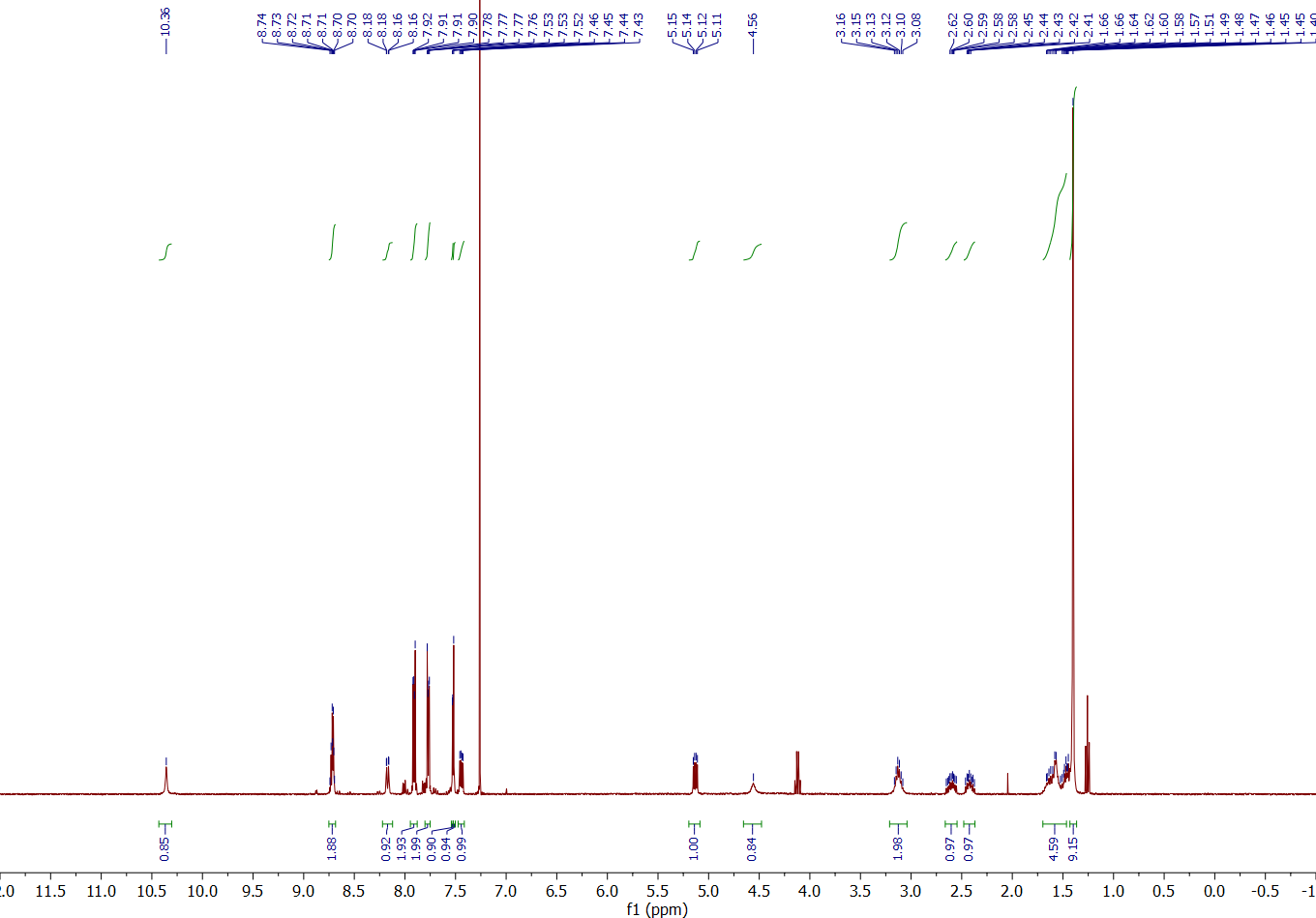


^13^C-NMR (126 MHz) in CDCl_3_


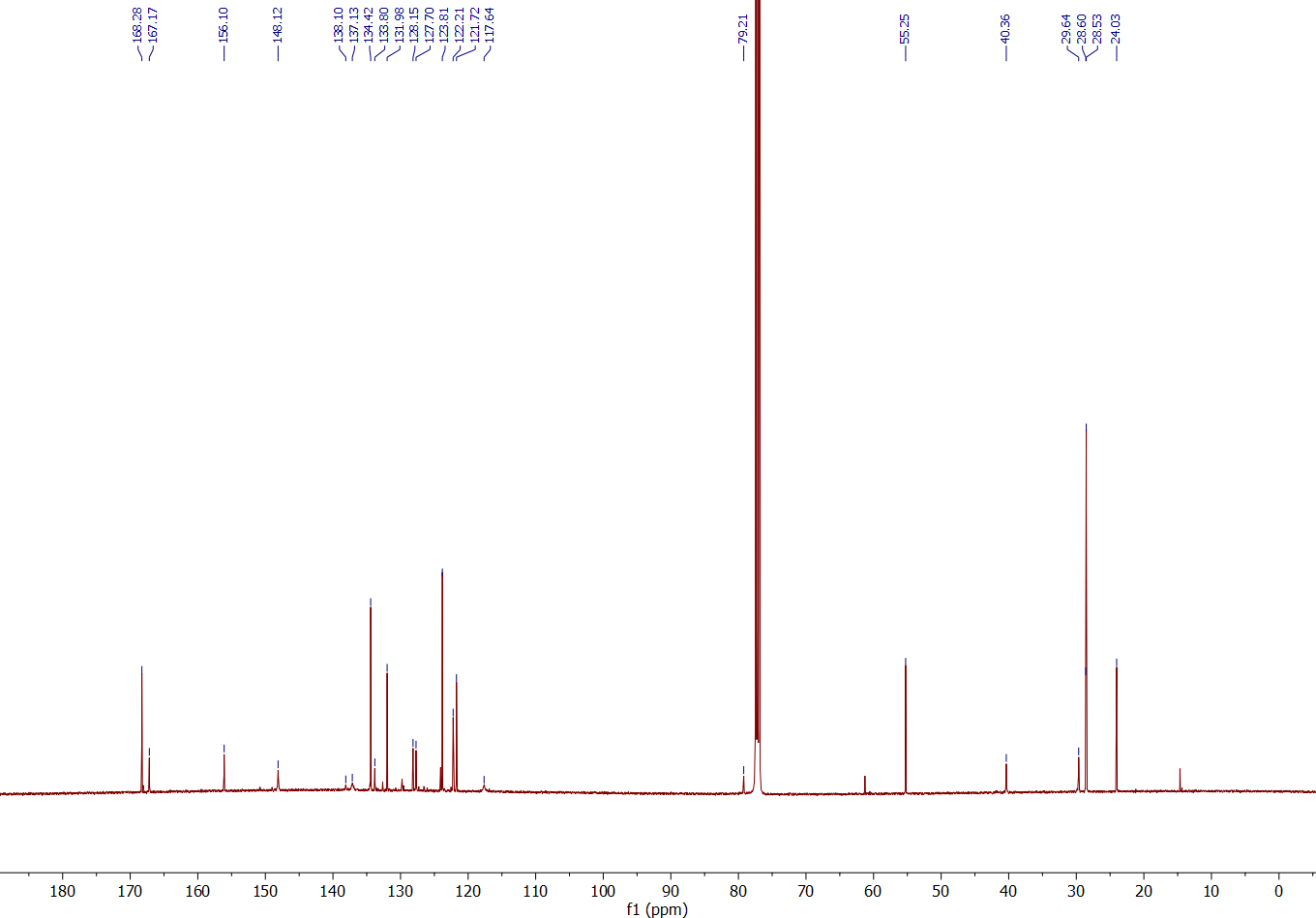


^1^H-NMR (400 MHz) in CDCl_3_


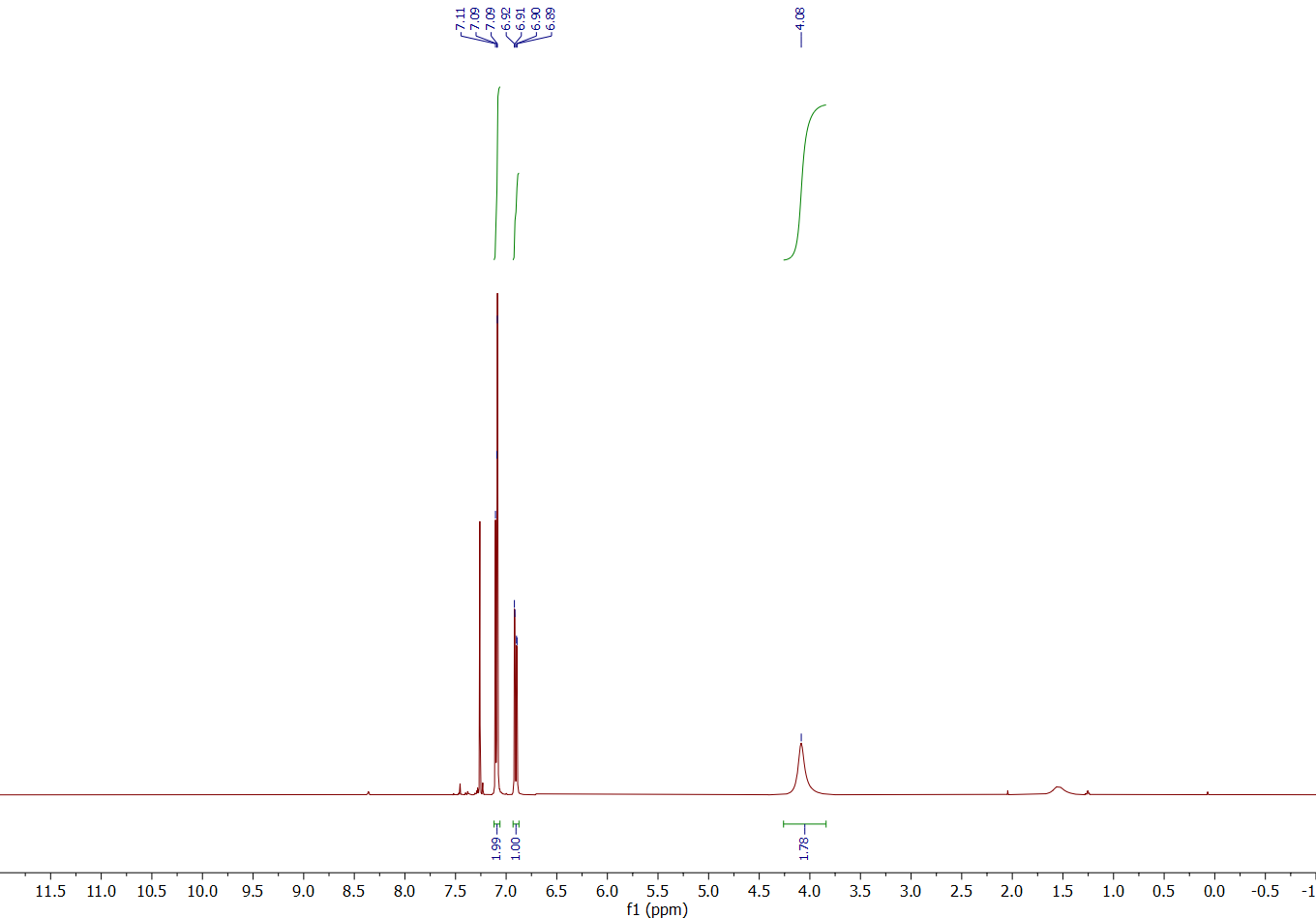


^13^C-NMR (101 MHz) in CDCl_3_


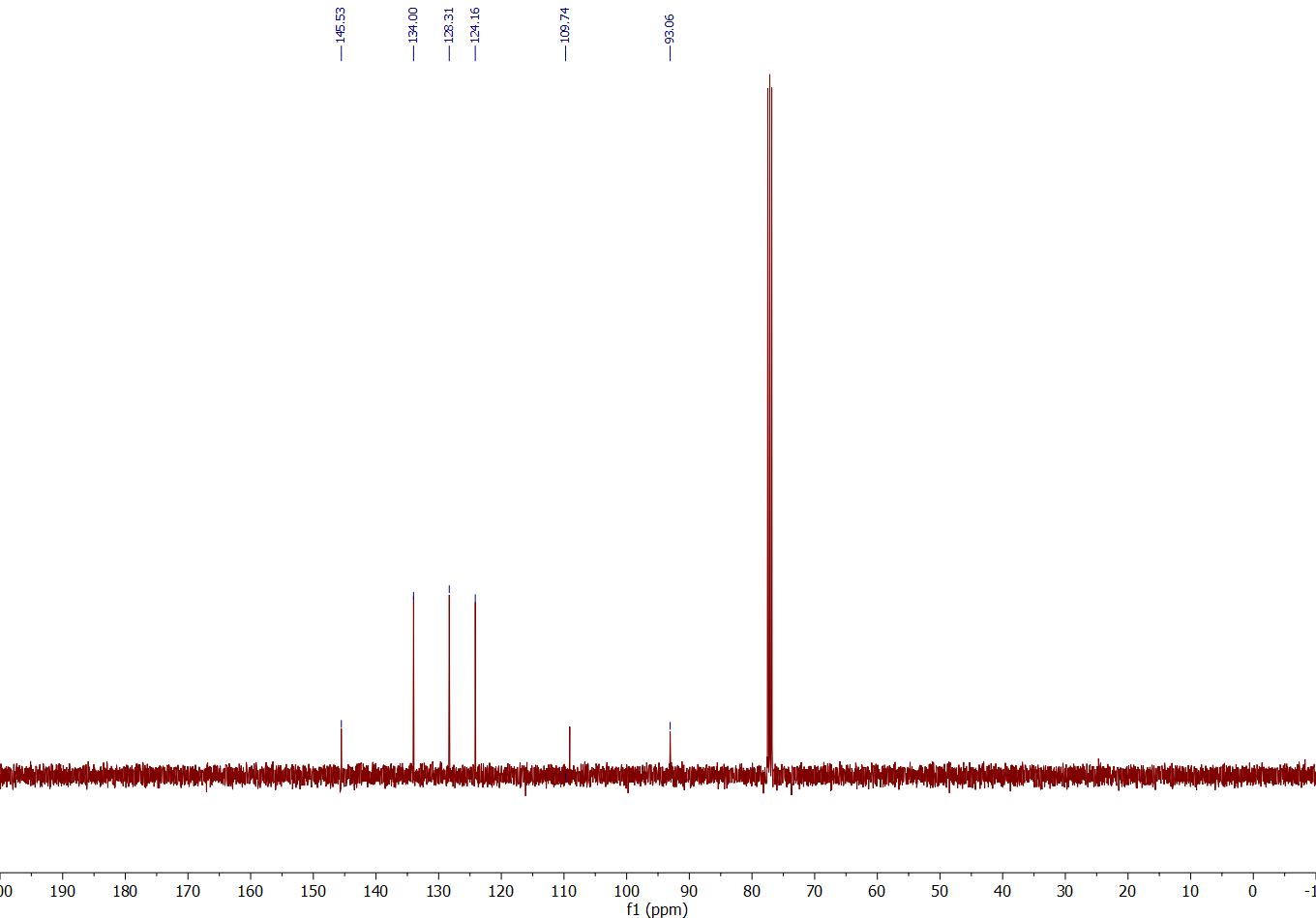


^1^H-NMR (500 MHz) in CDCl_3_


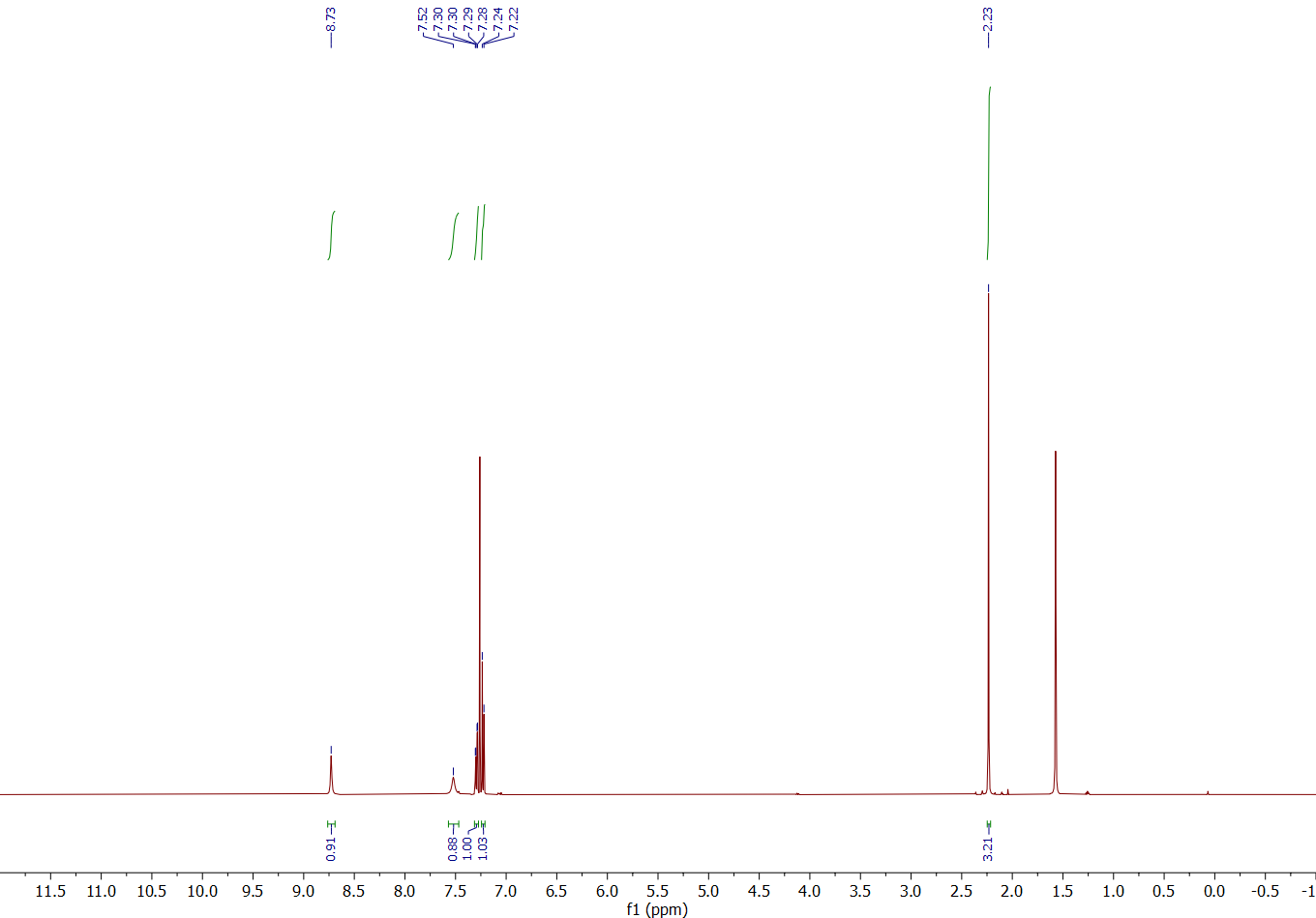


^13^C-NMR (101 MHz) in CDCl_3_


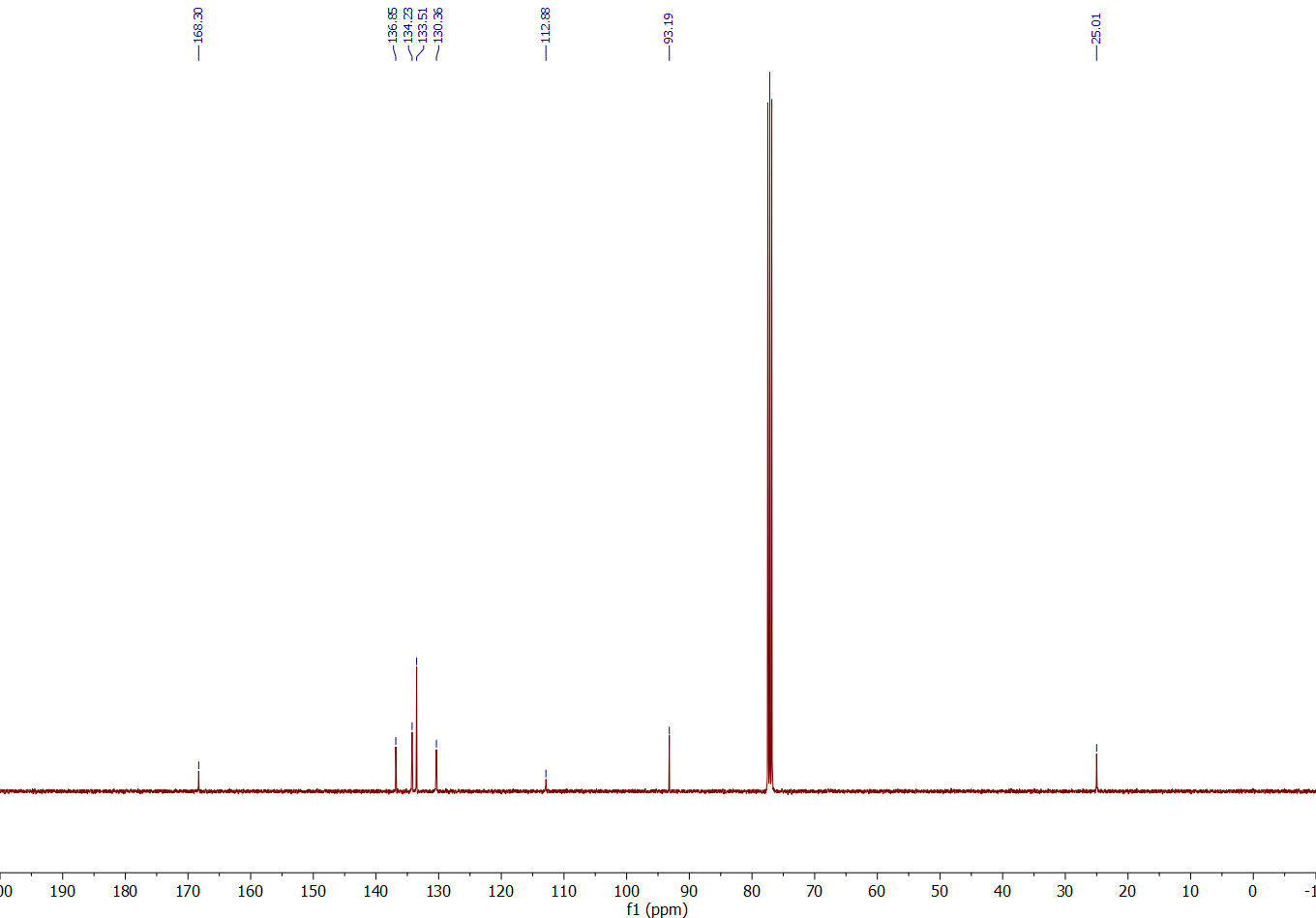


^1^H-NMR (400 MHz) in CDCl_3_


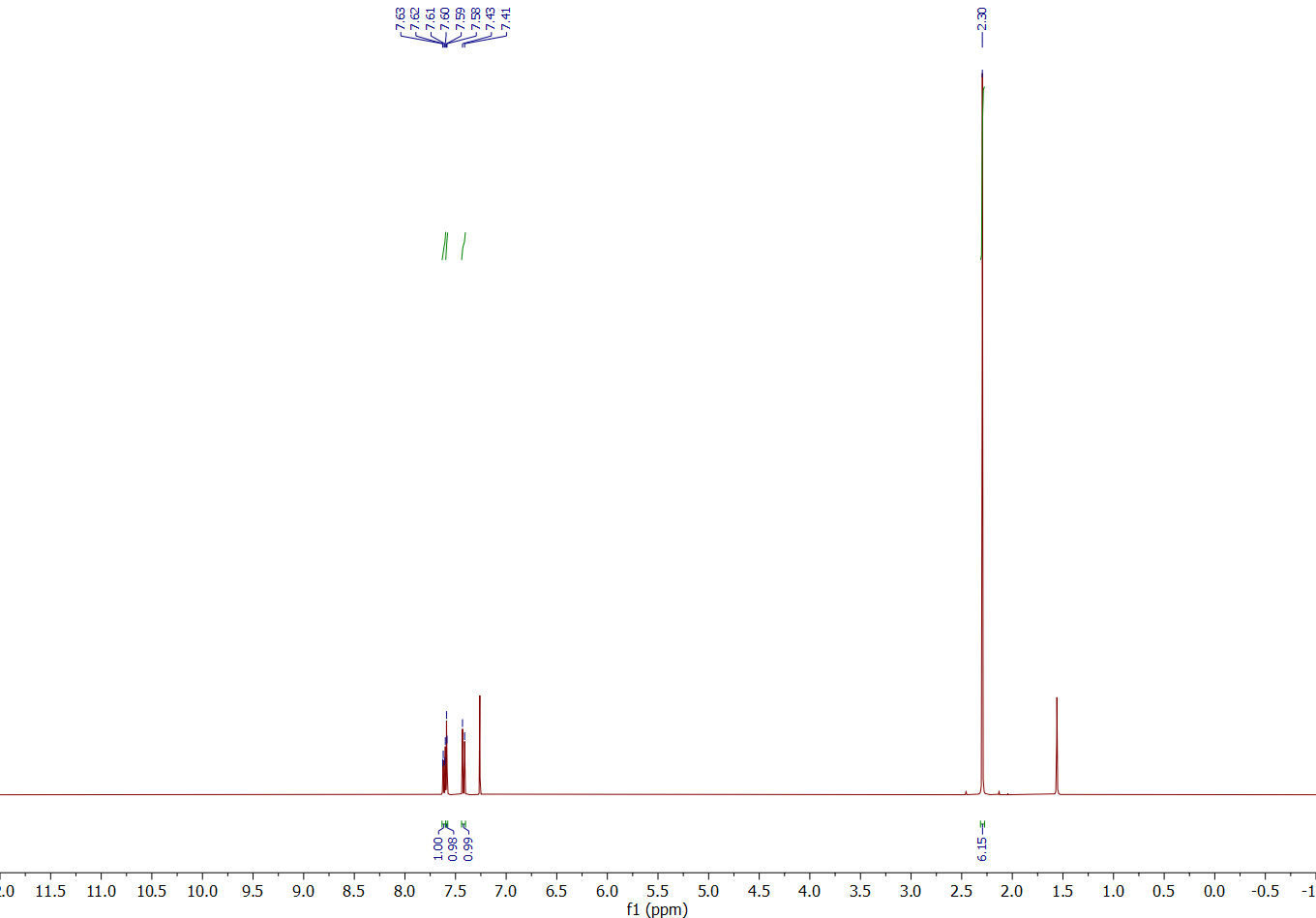


^13^C-NMR (101 MHz) in CDCl_3_


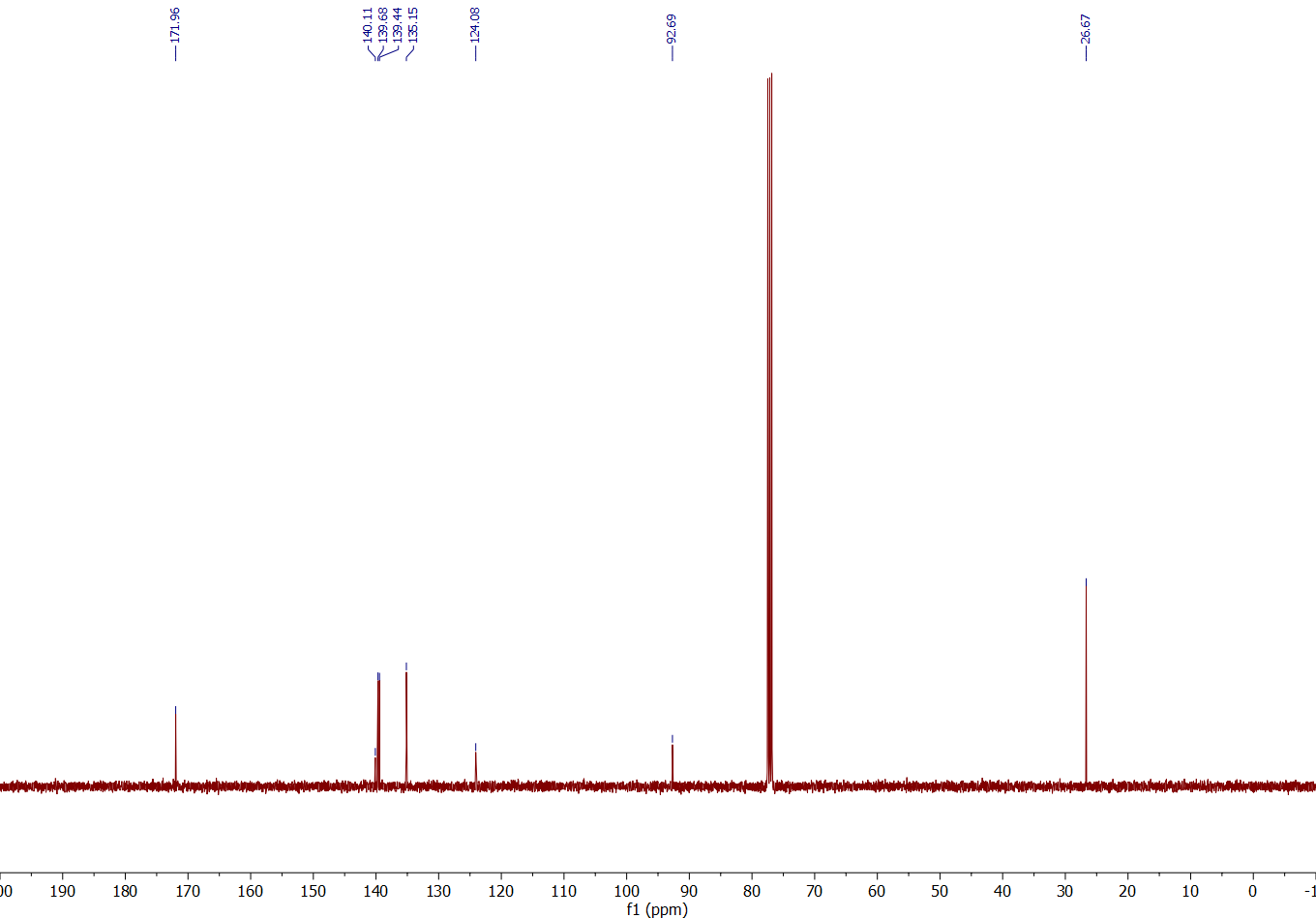


^1^H-NMR (400 MHz) in DMSO-*d_6_*


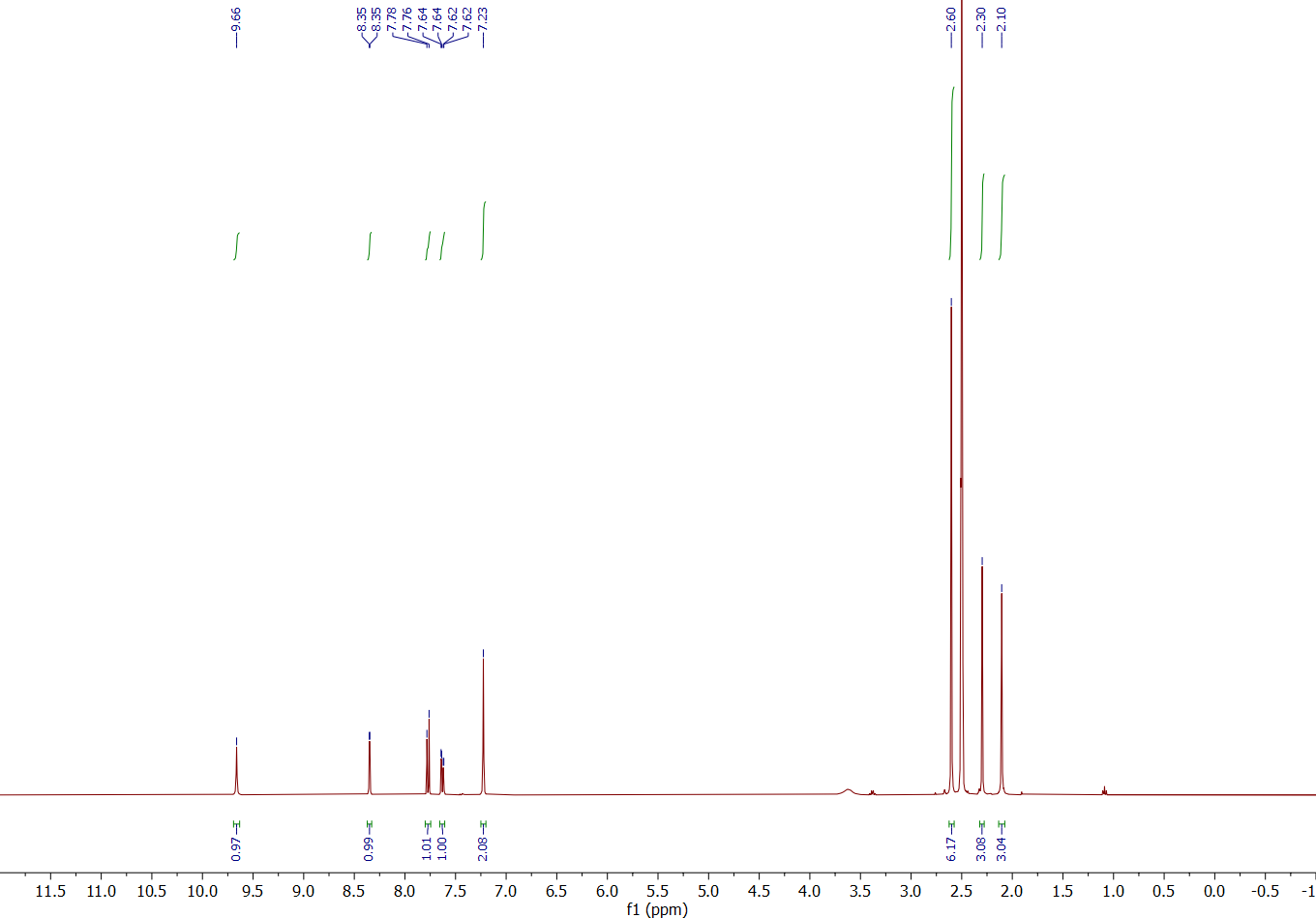


^13^C-NMR (101 MHz) in DMSO-*d_6_*


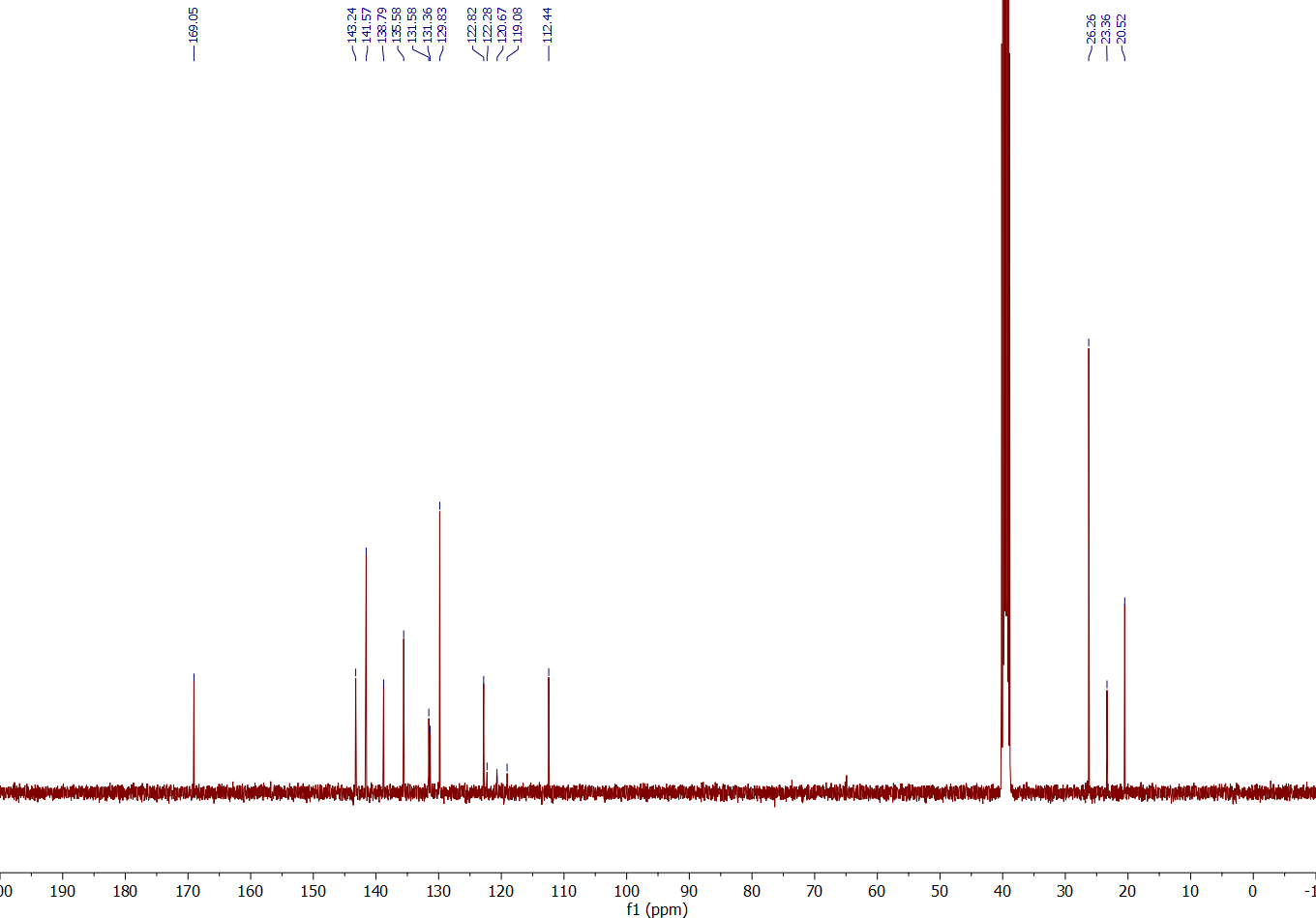


^1^H-NMR (400 MHz) in CDCl_3_


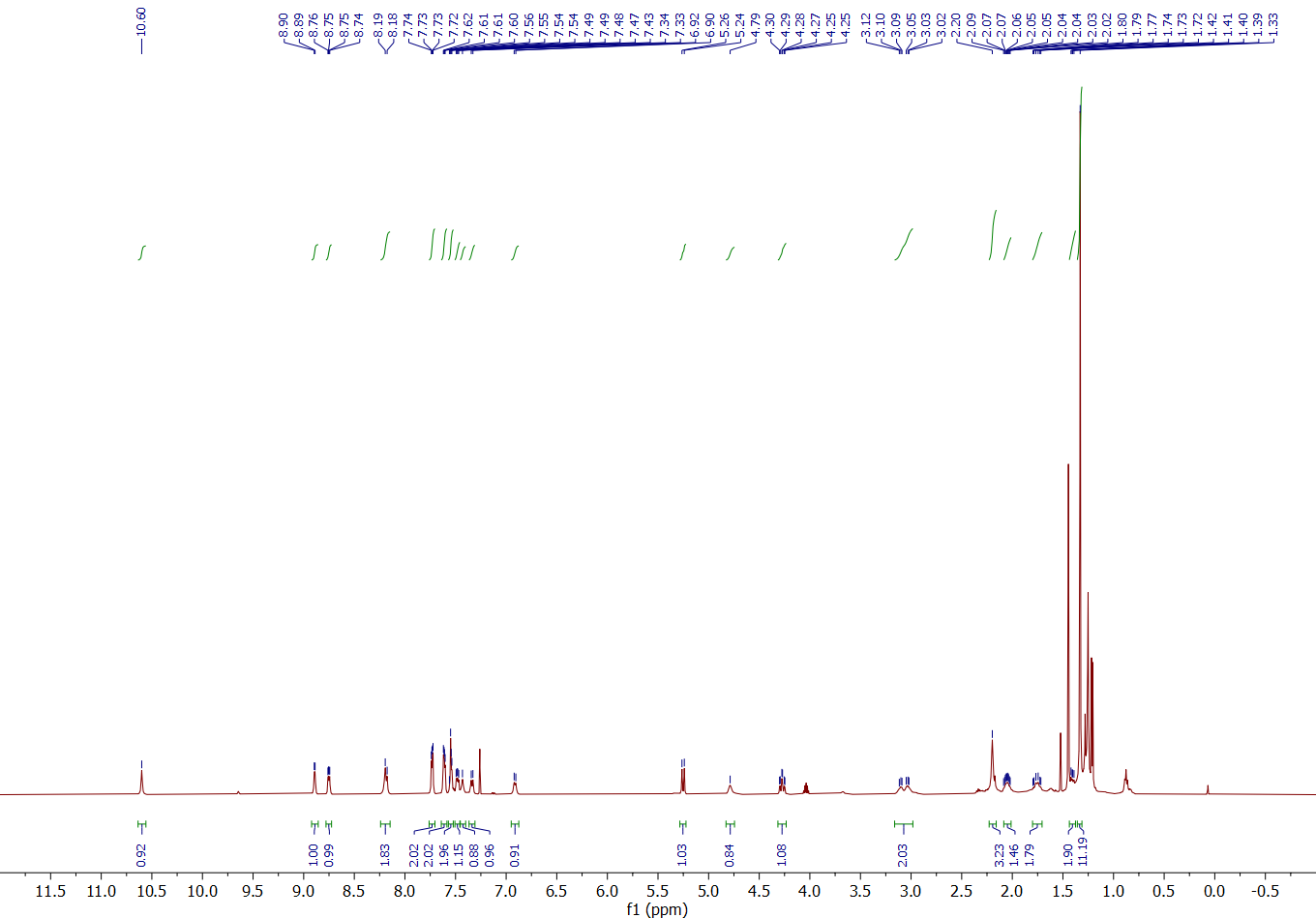


^13^C-NMR (126 MHz) in CDCl_3_


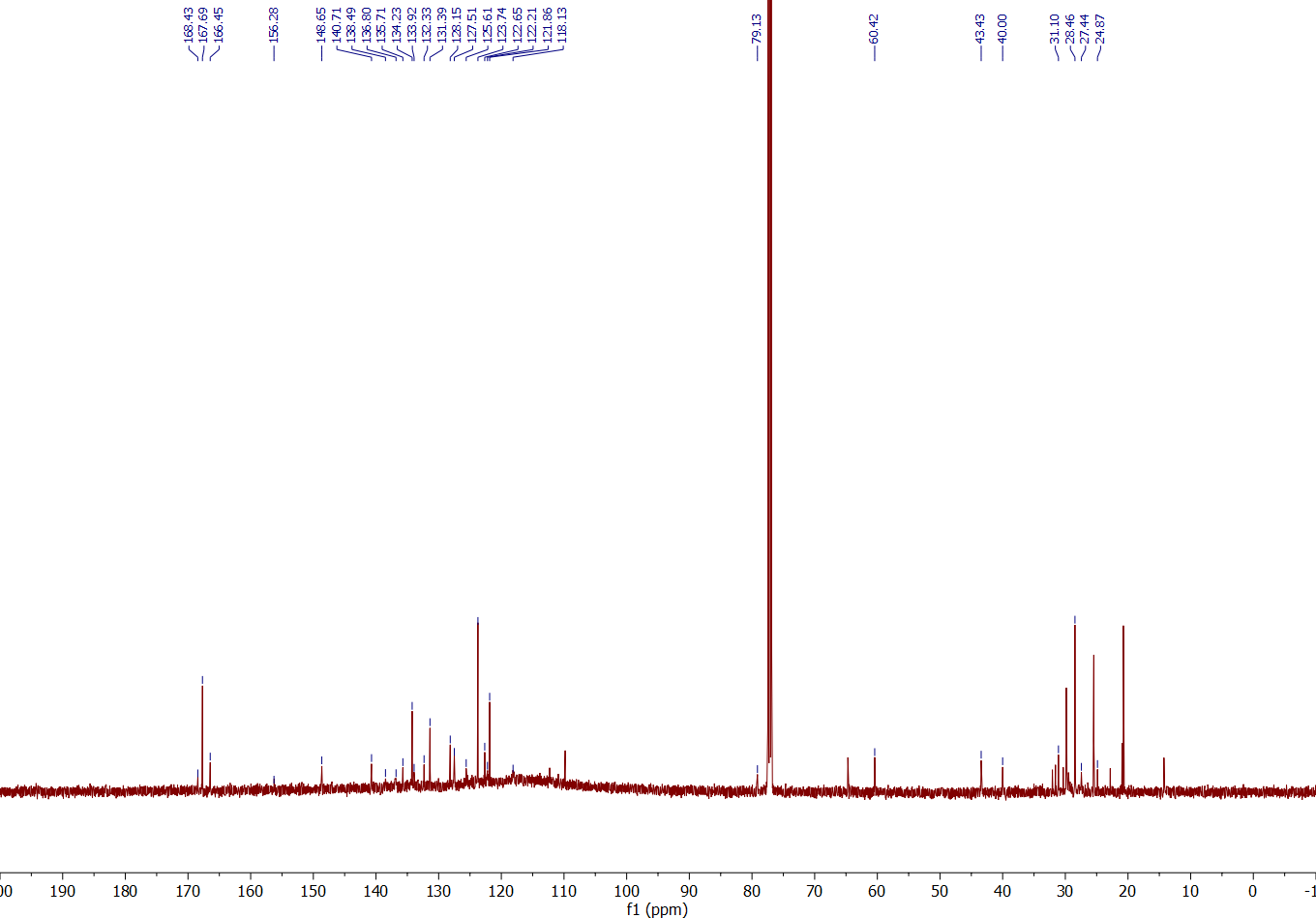


^1^H-NMR (400 MHz) in CDCl_3_


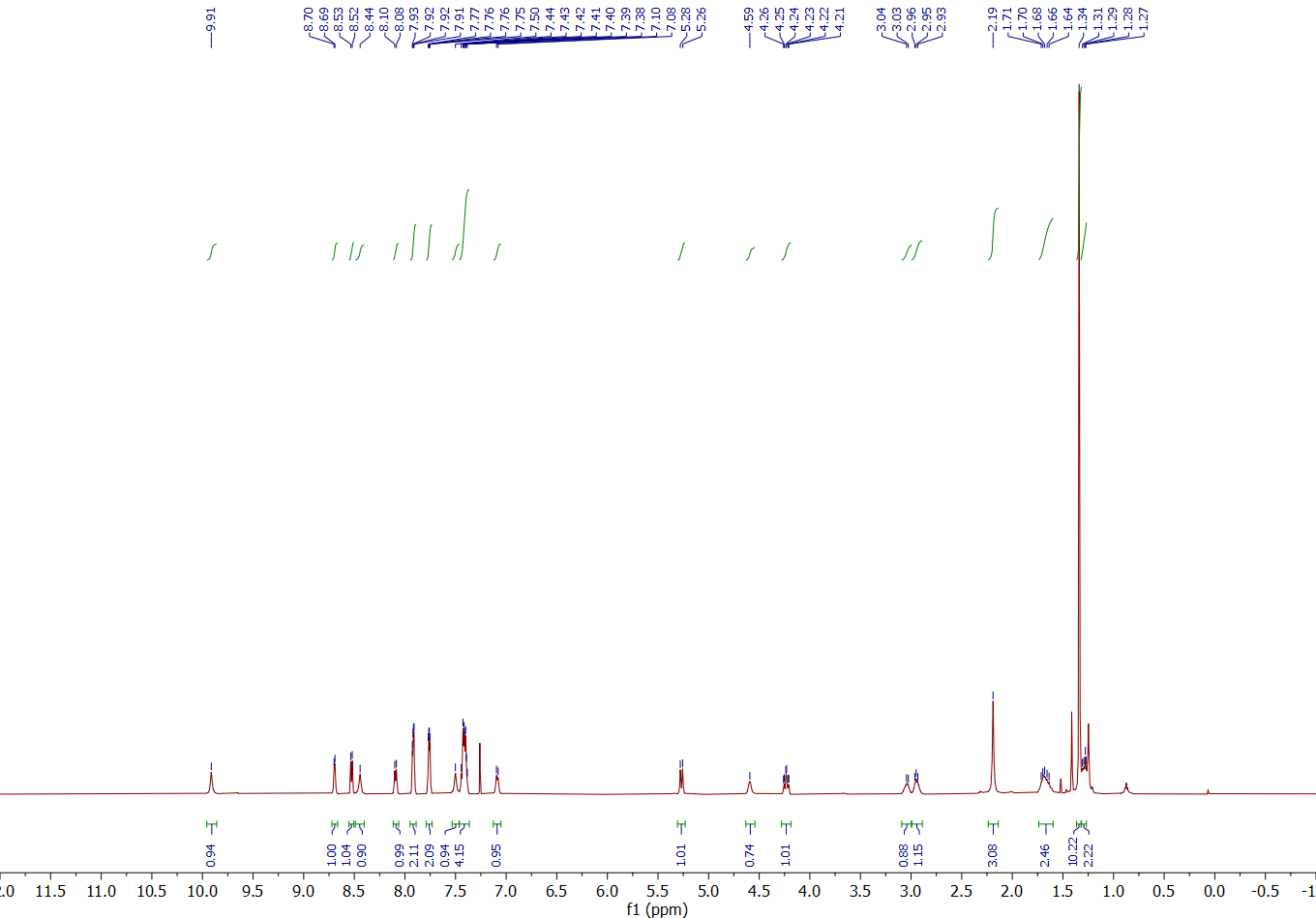


^13^C-NMR (126 MHz) in CDCl_3_


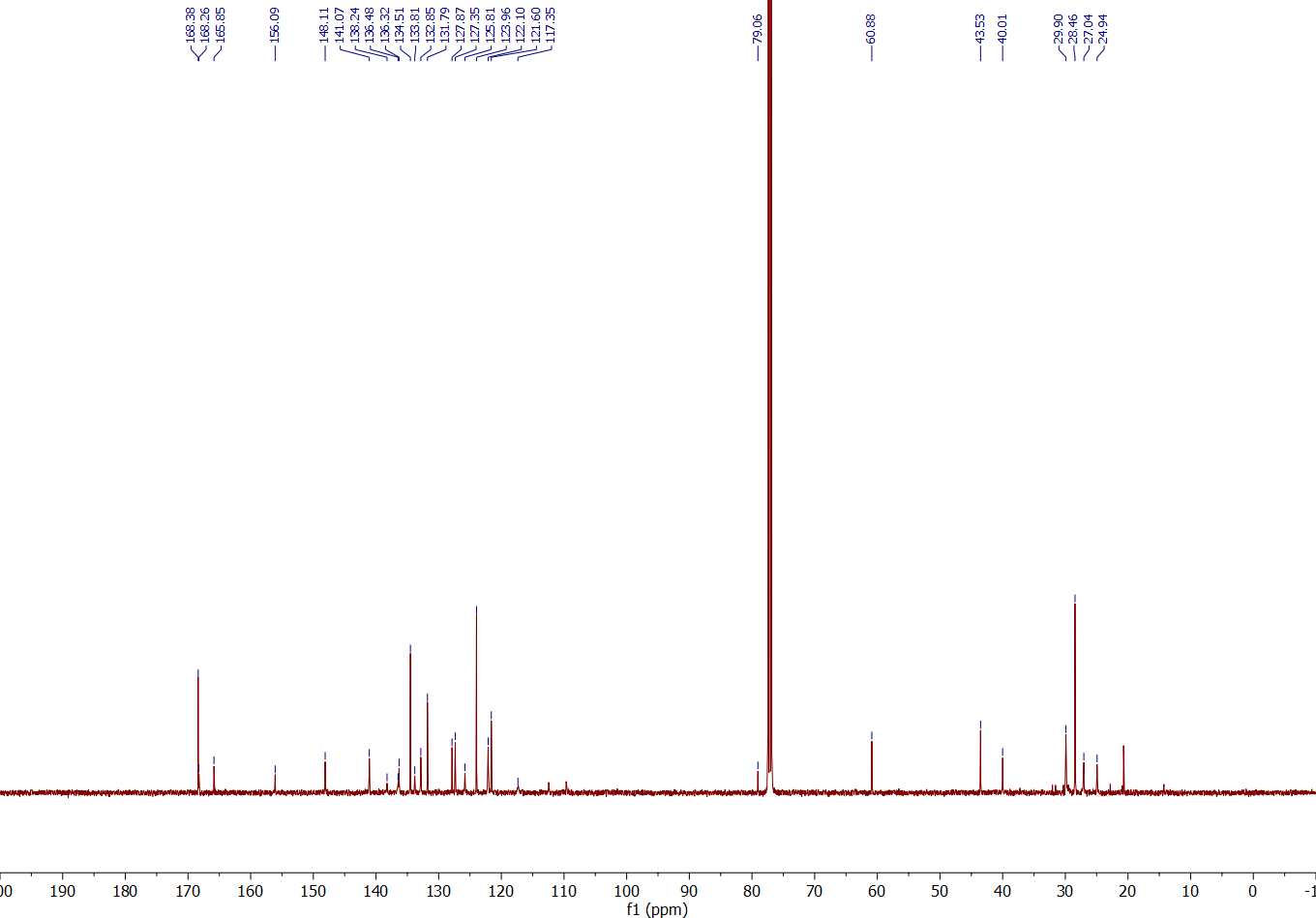


^1^H-NMR (500 MHz) in CDCl_3_


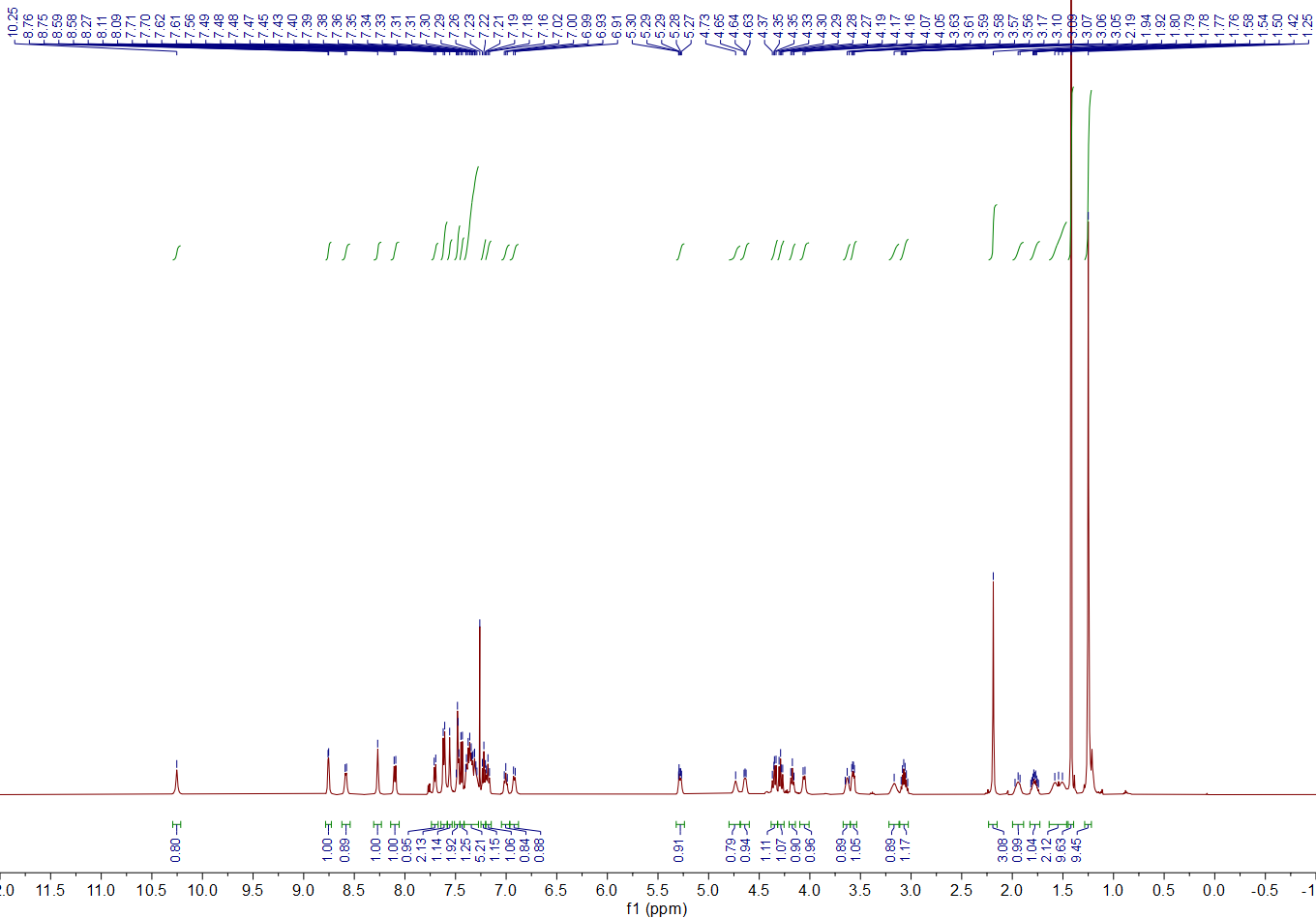


^13^C-NMR (126 MHz) in CDCl_3_


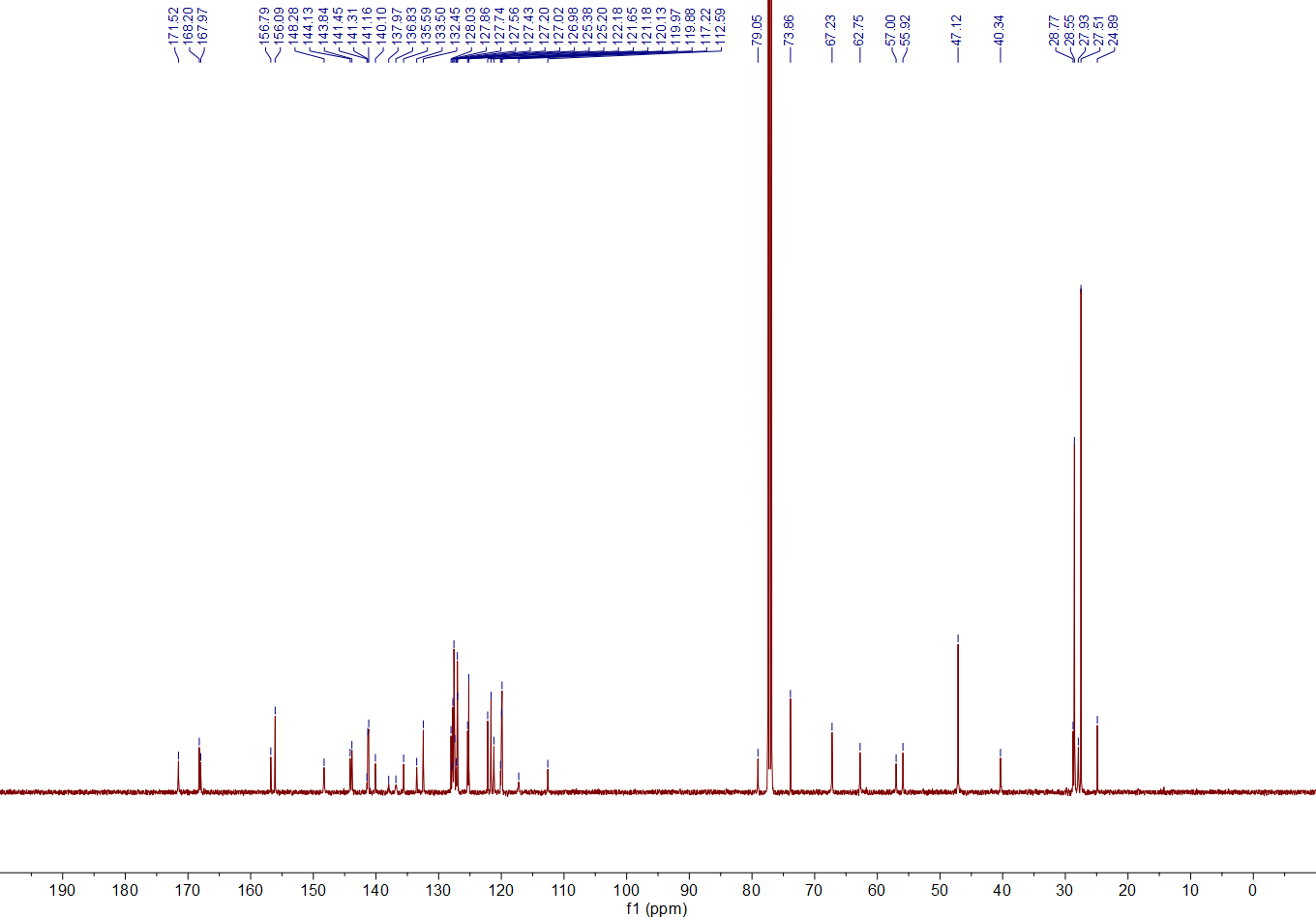


COSY-NMR for compound **28** in CDCl_3_


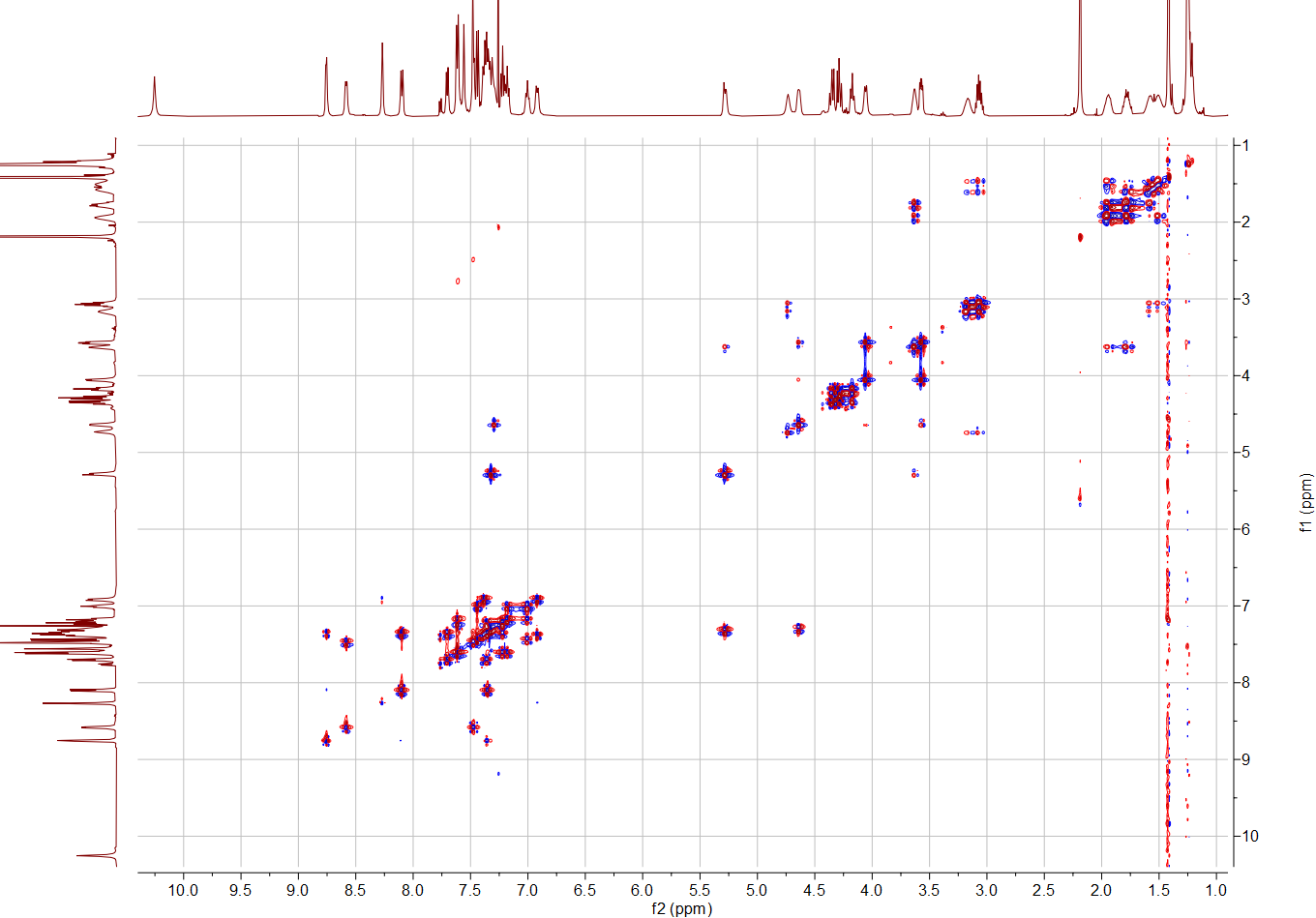


HSQC-NMR for compound **28** in CDCl_3_


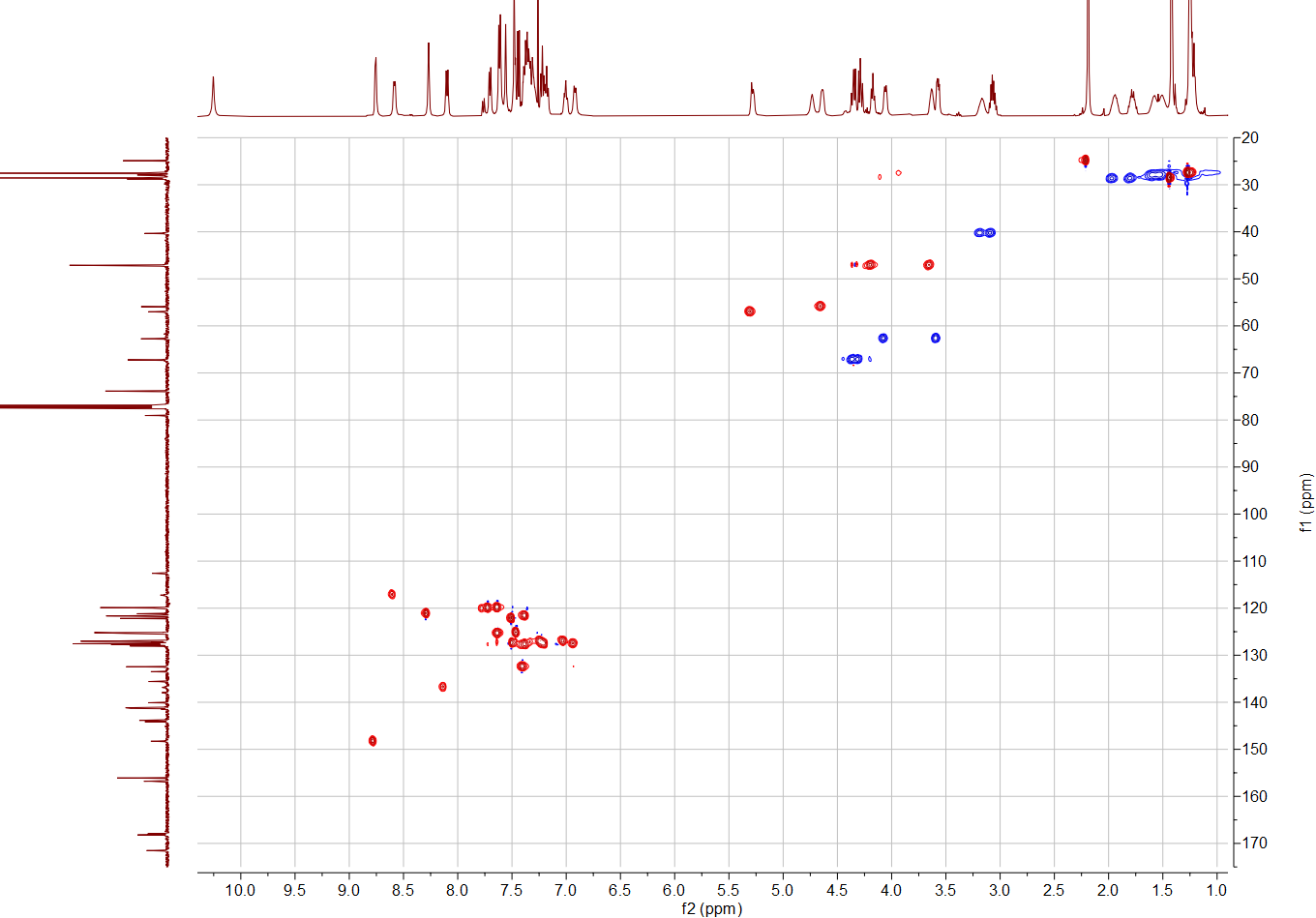


HMBC-NMR for compound **28** in CDCl_3_


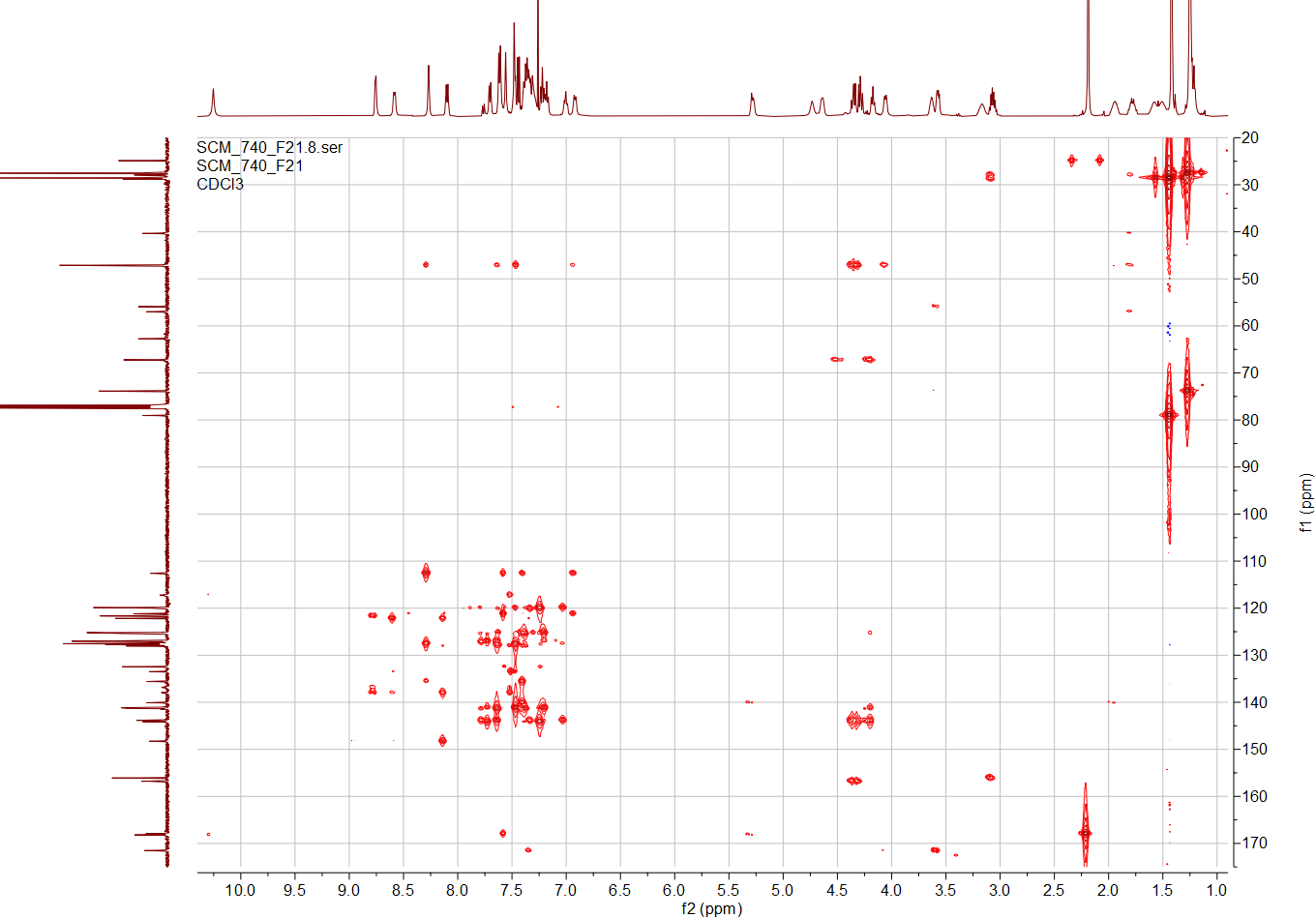


NOESY-NMR for compound **28** in CDCl_3_


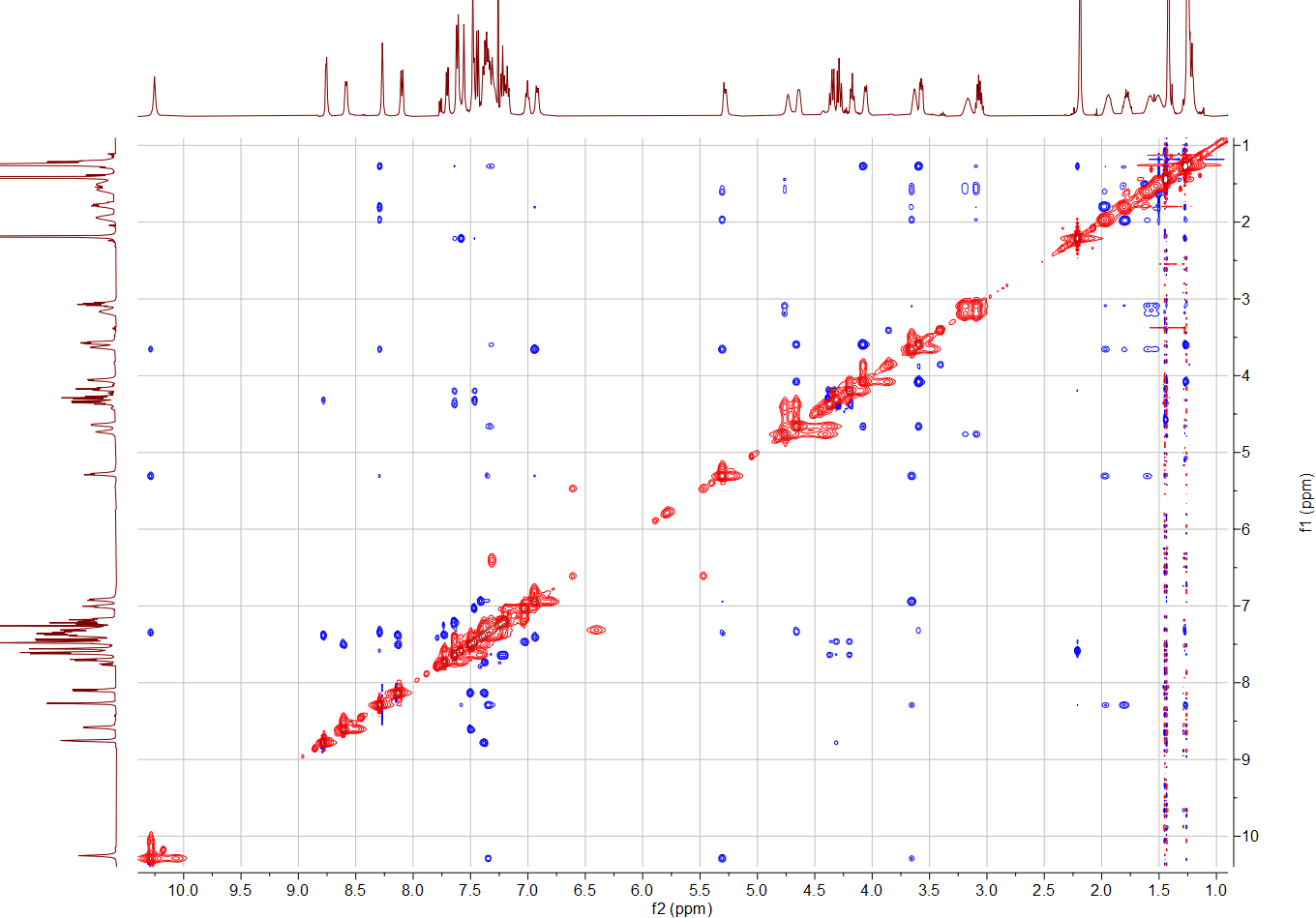


^1^H-NMR (500 MHz) in CDCl_3_


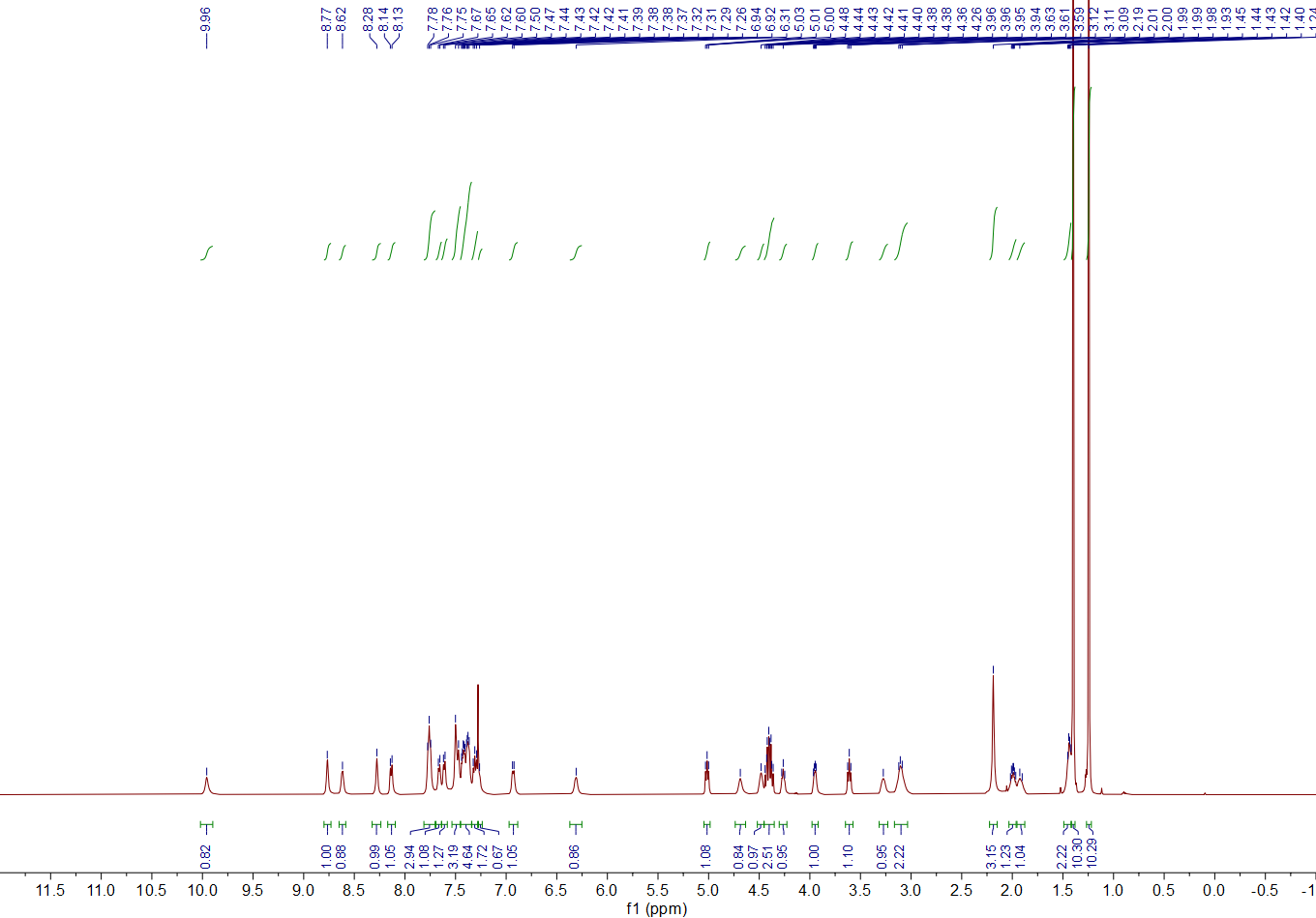


^13^C-NMR (126 MHz) in CDCl_3_


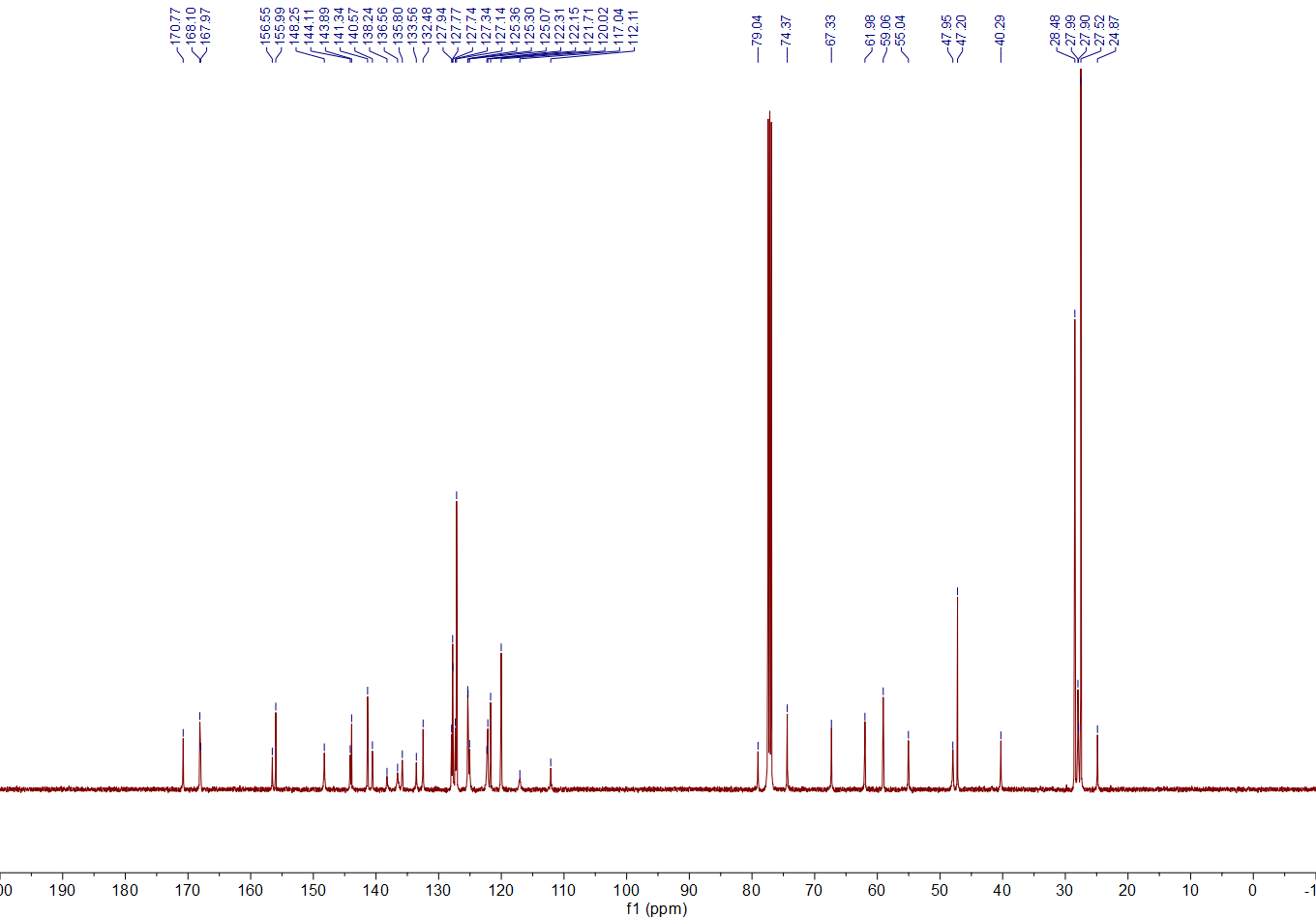


COSY-NMR for compound **29** in CDCl_3_


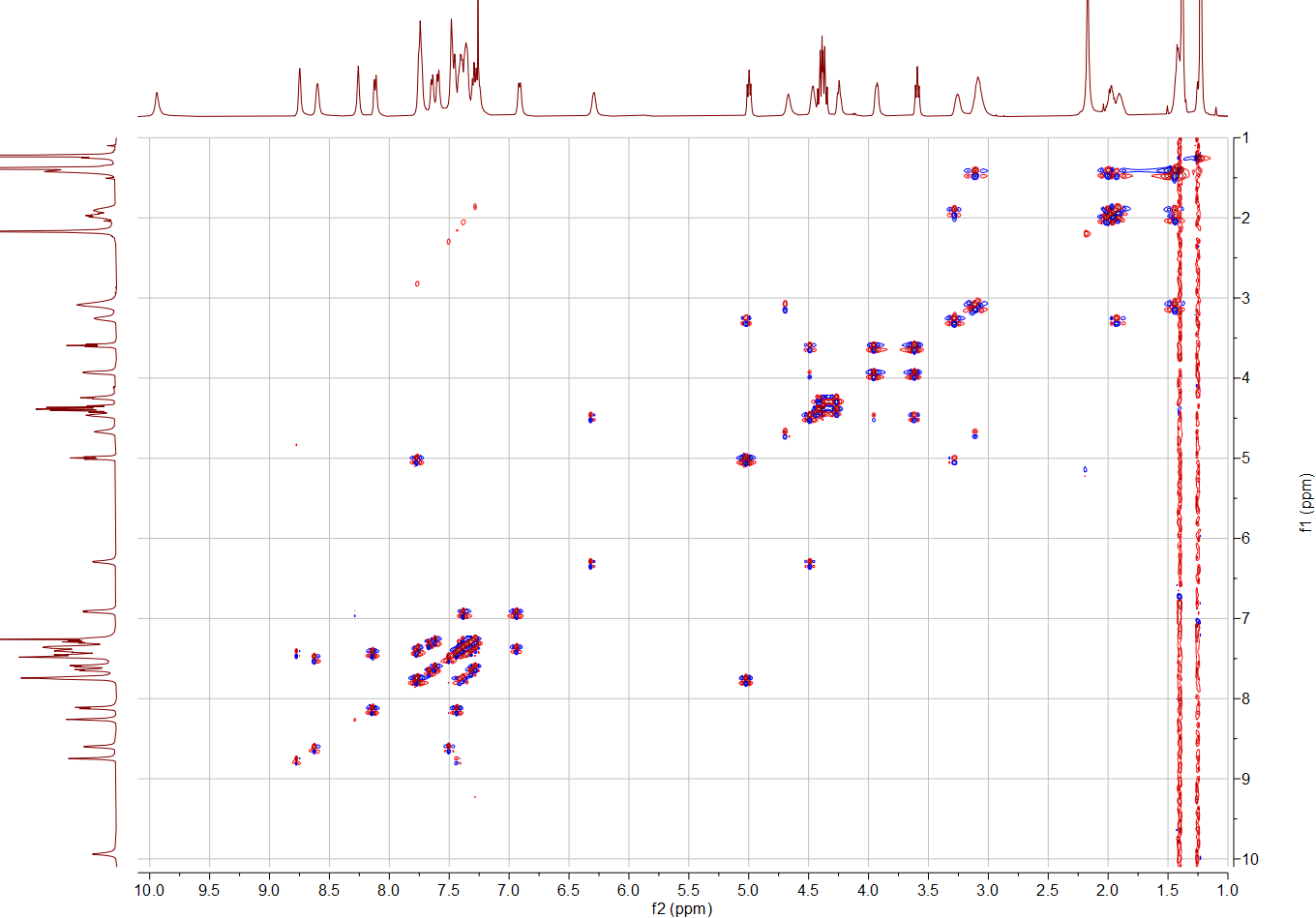


HSQC-NMR for compound **29** in CDCl_3_


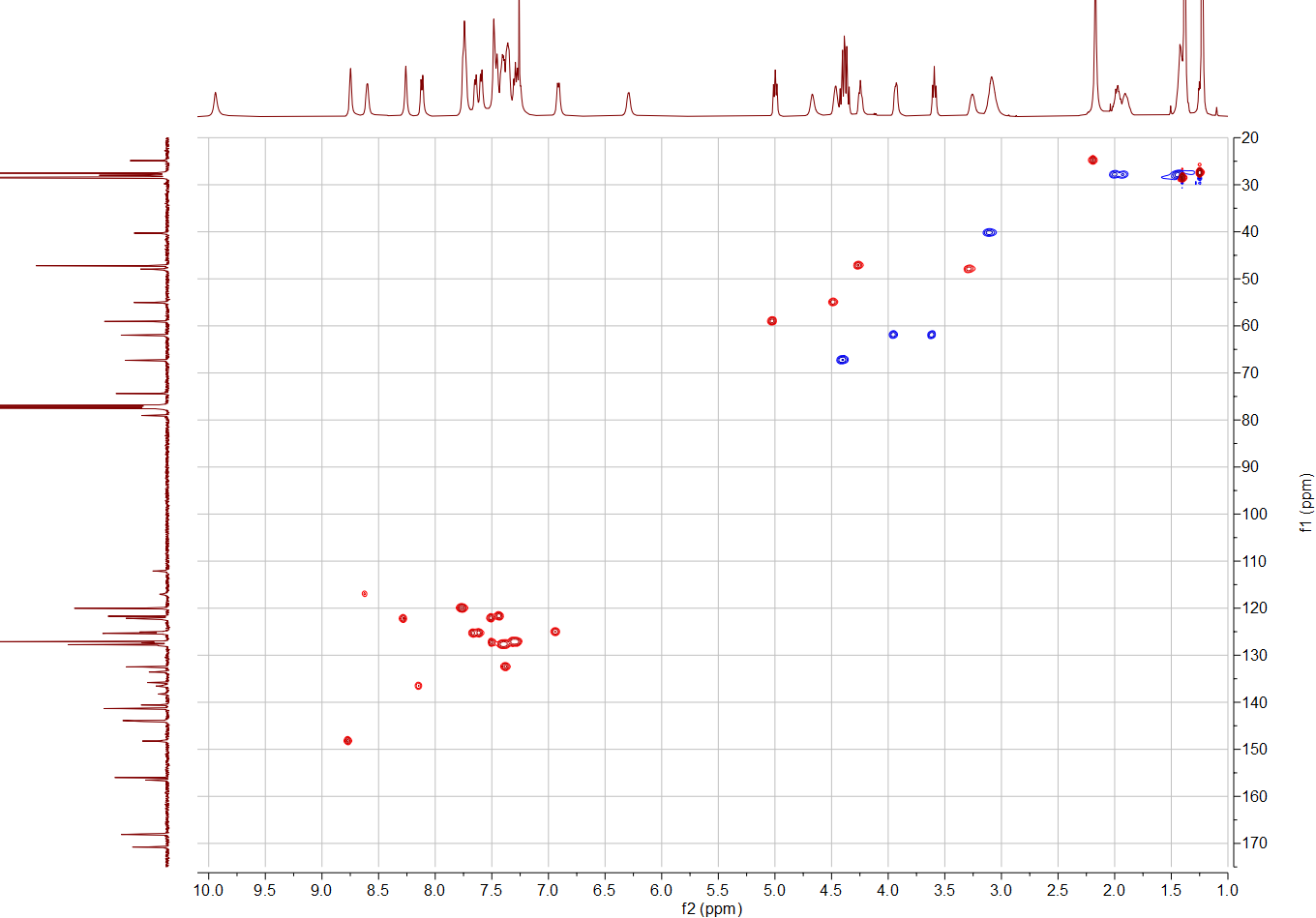


HMBC-NMR for compound **29** in CDCl_3_


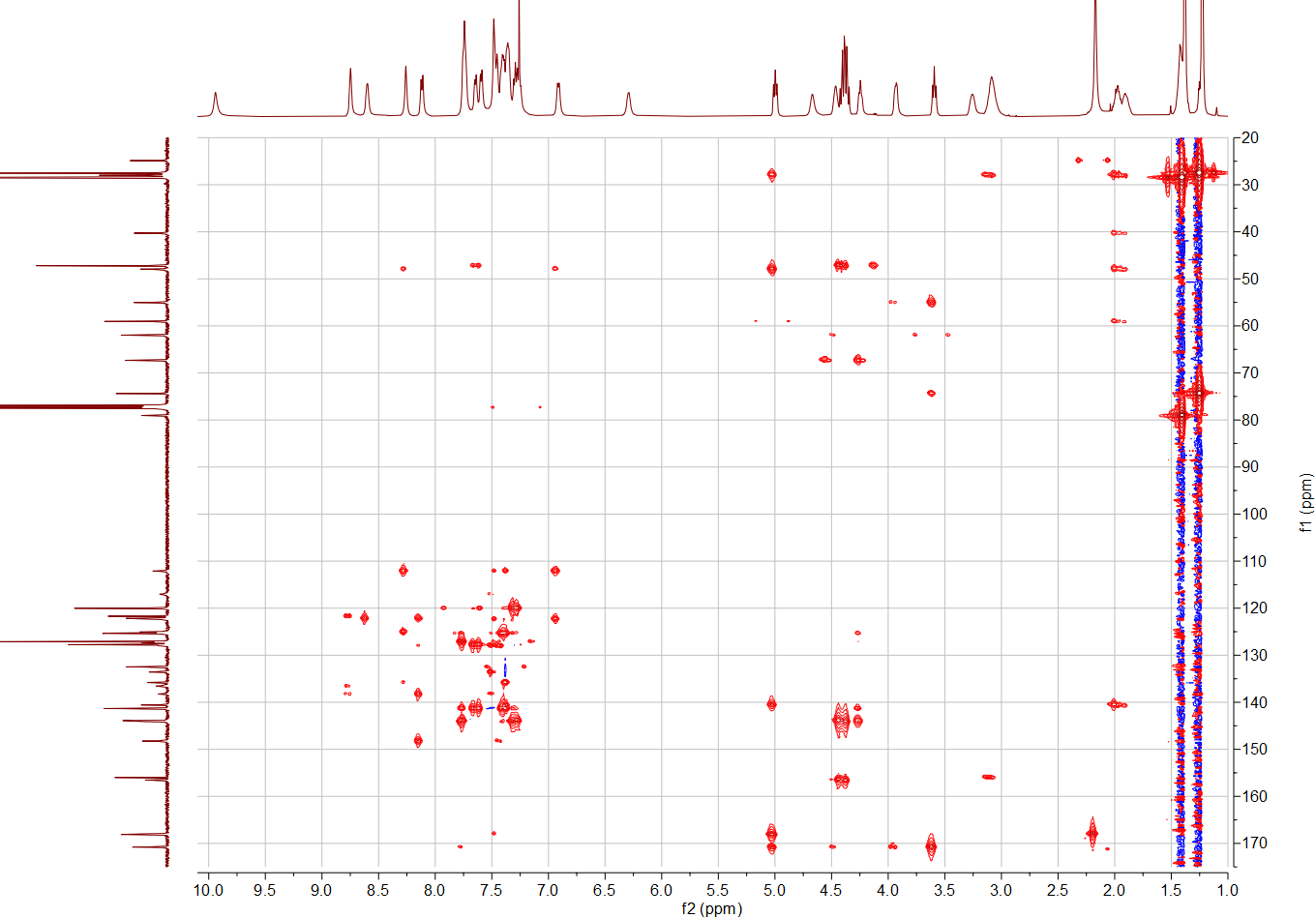


NOESY-NMR for compound **29** in CDCl_3_


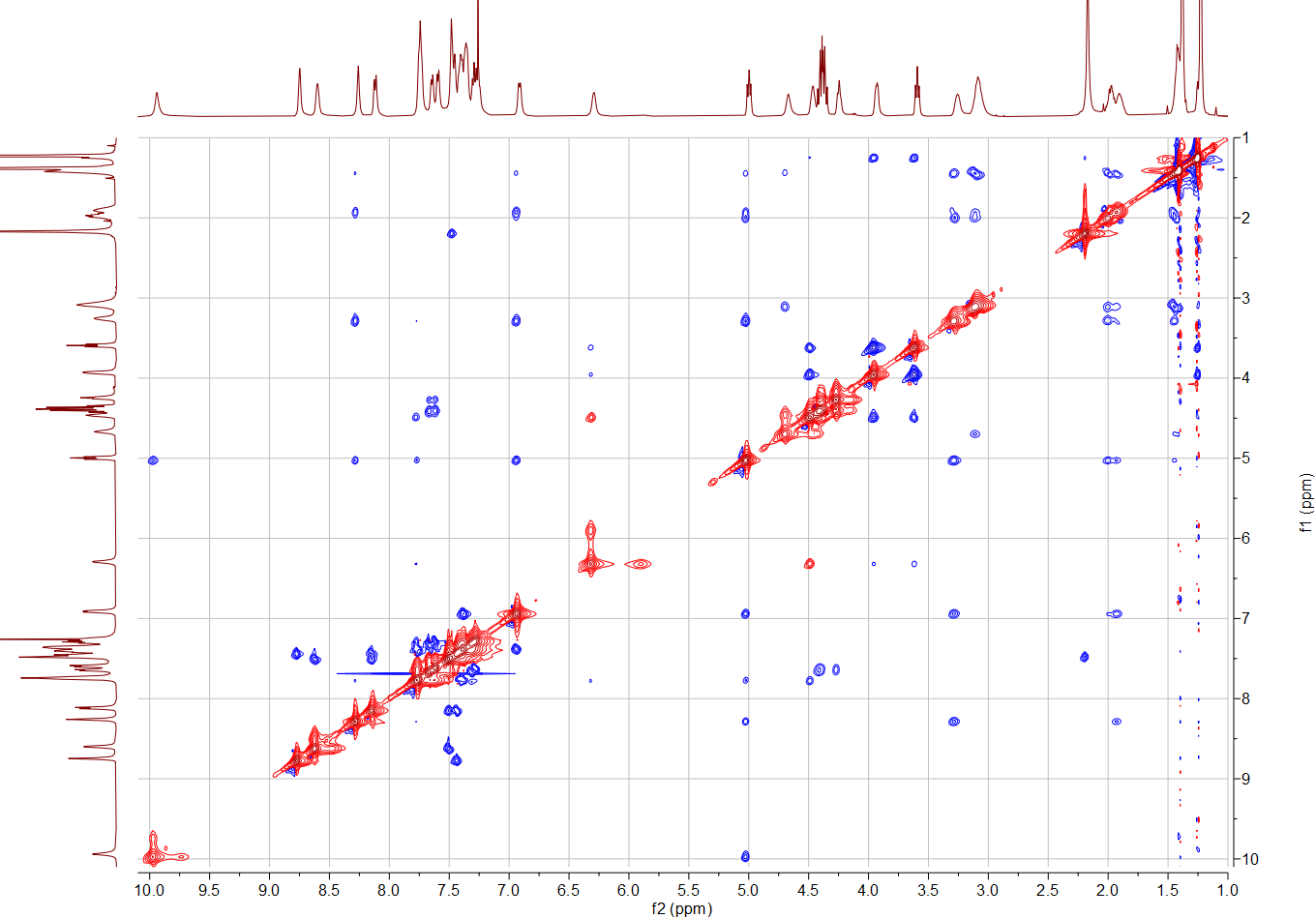


^1^H-NMR (400 MHz) in DMSO-*d_6_*


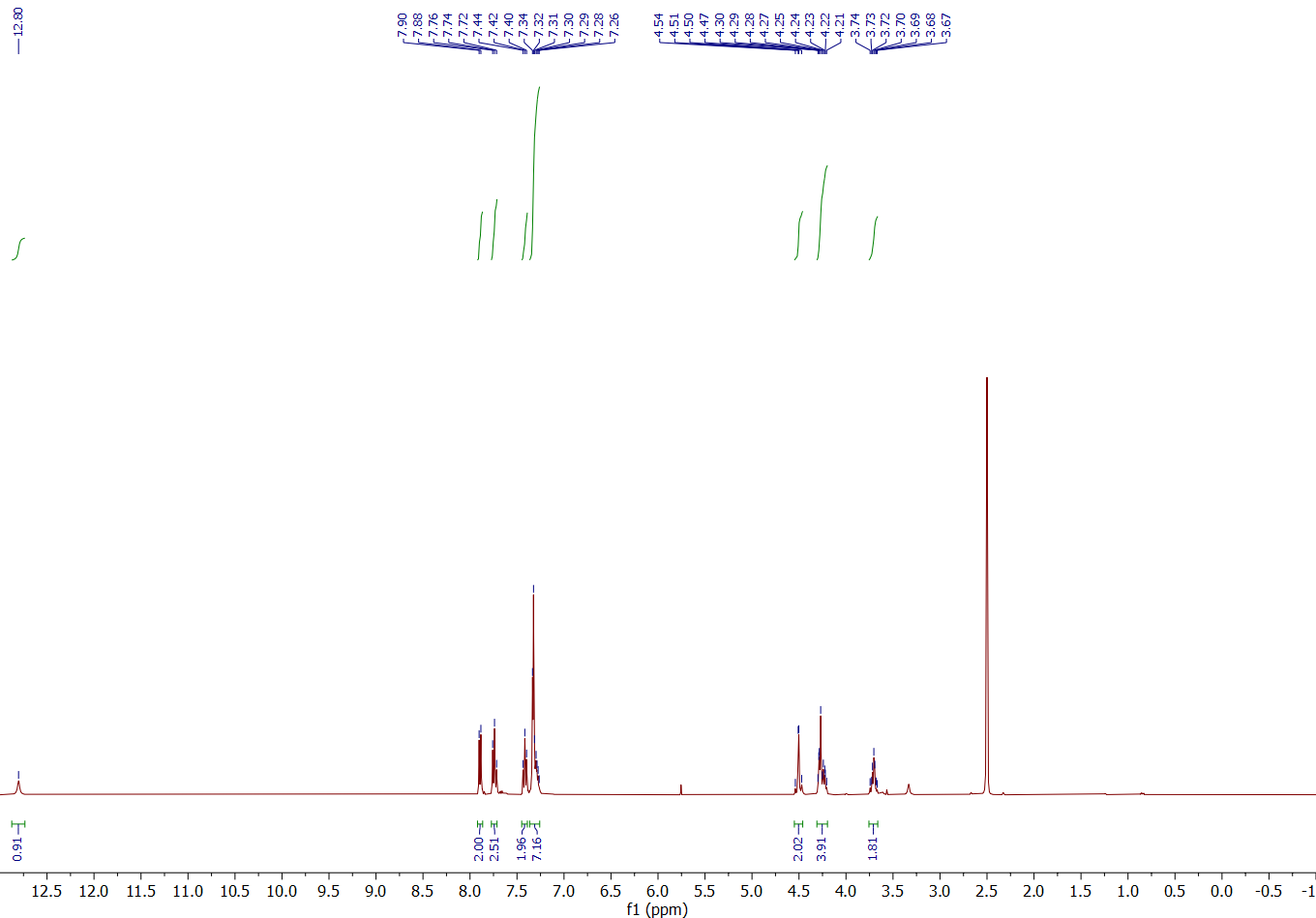


^13^C-NMR (101 MHz) in CDCl_3_


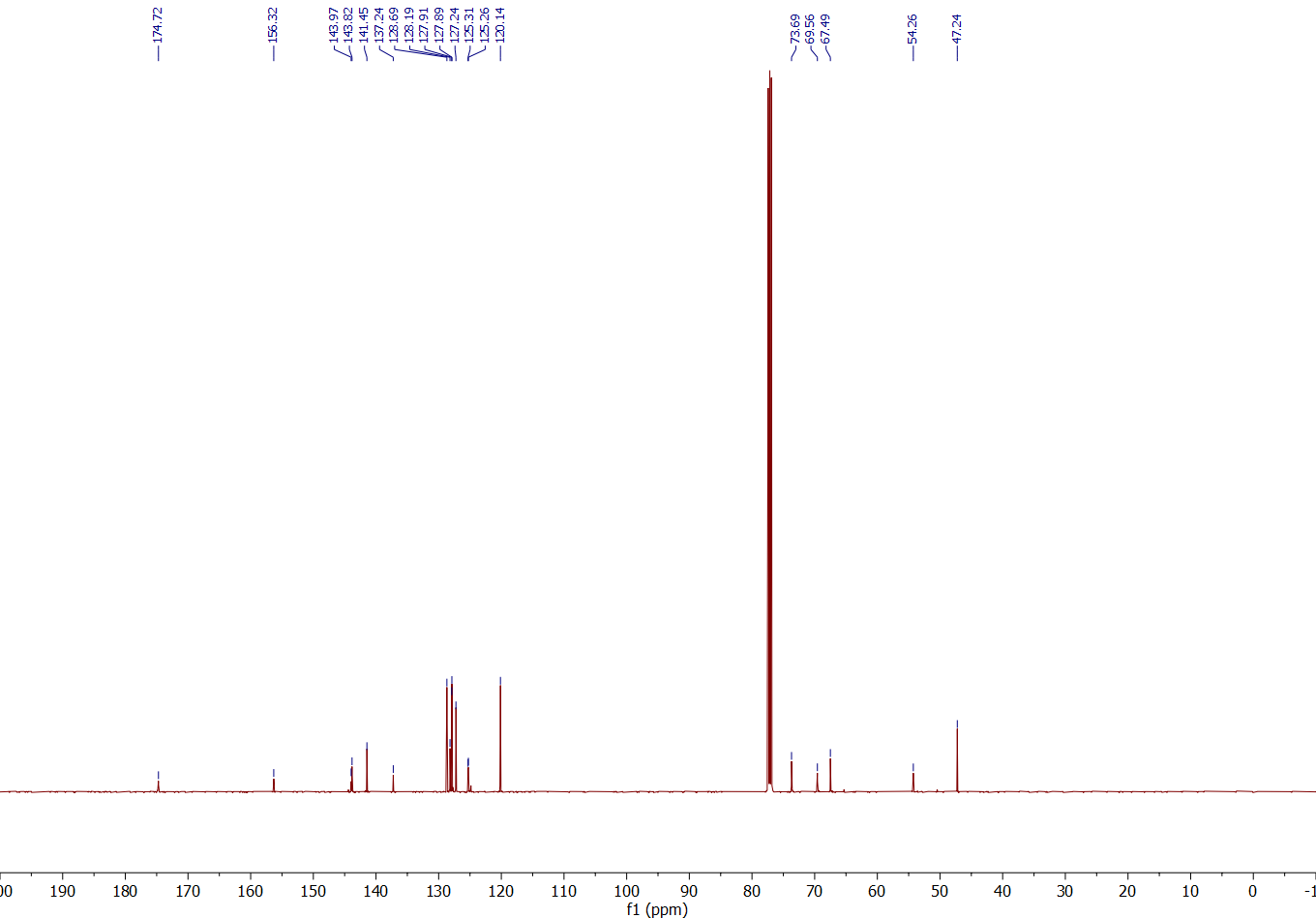


^1^H-NMR (400 MHz) in CDCl_3_


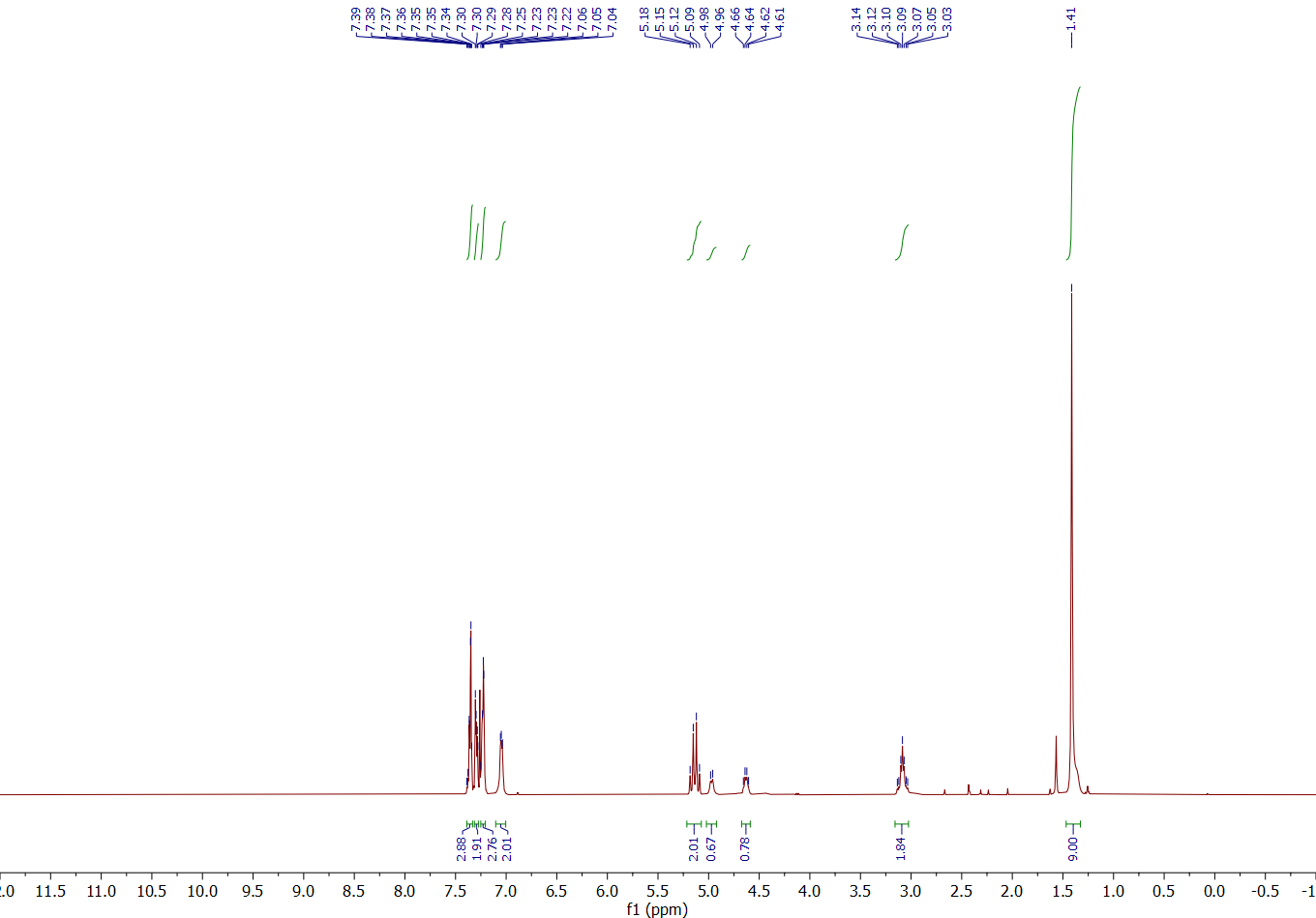


^13^C-NMR (101 MHz) in CDCl_3_


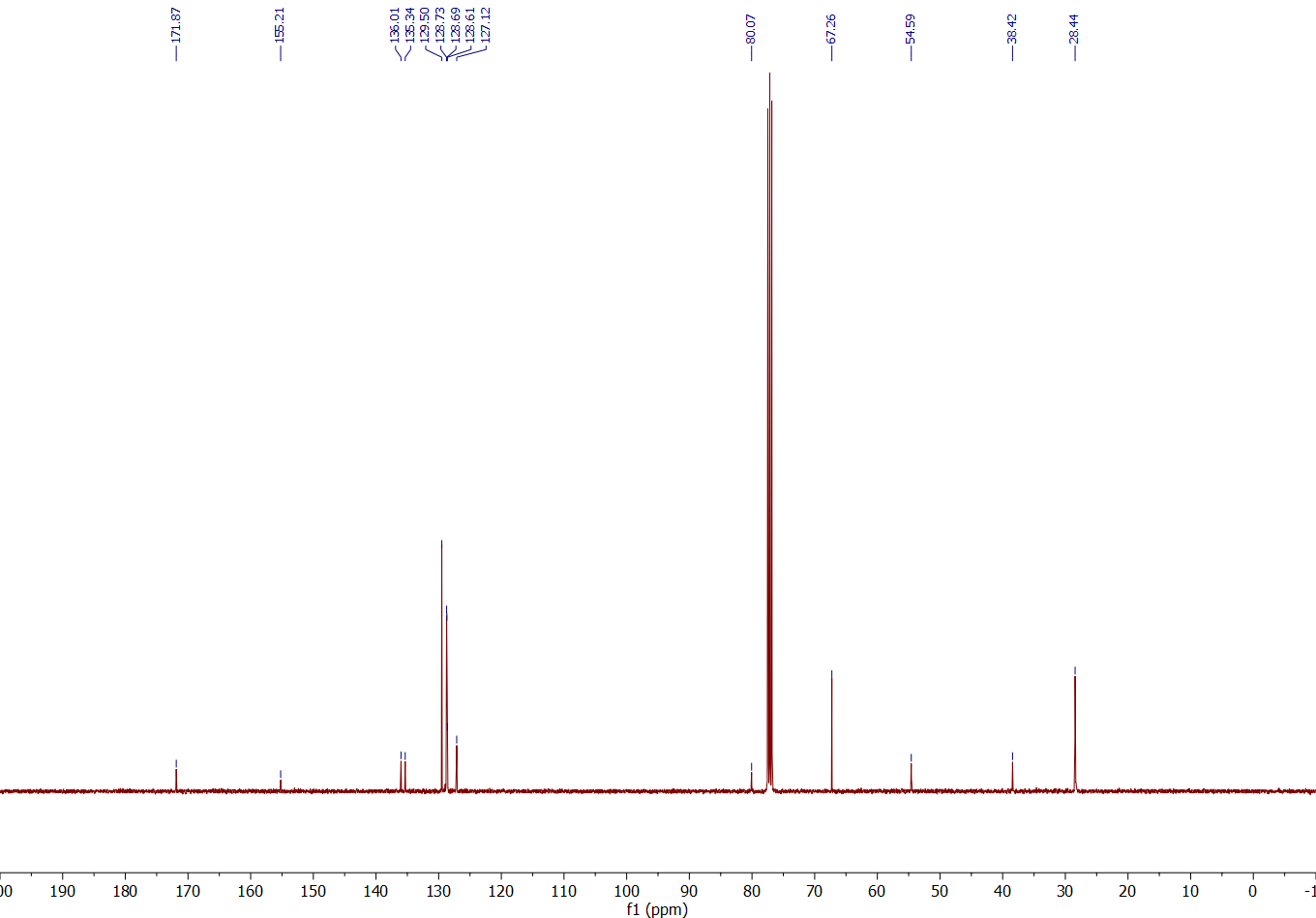


^1^H-NMR (400 MHz) in CDCl_3_


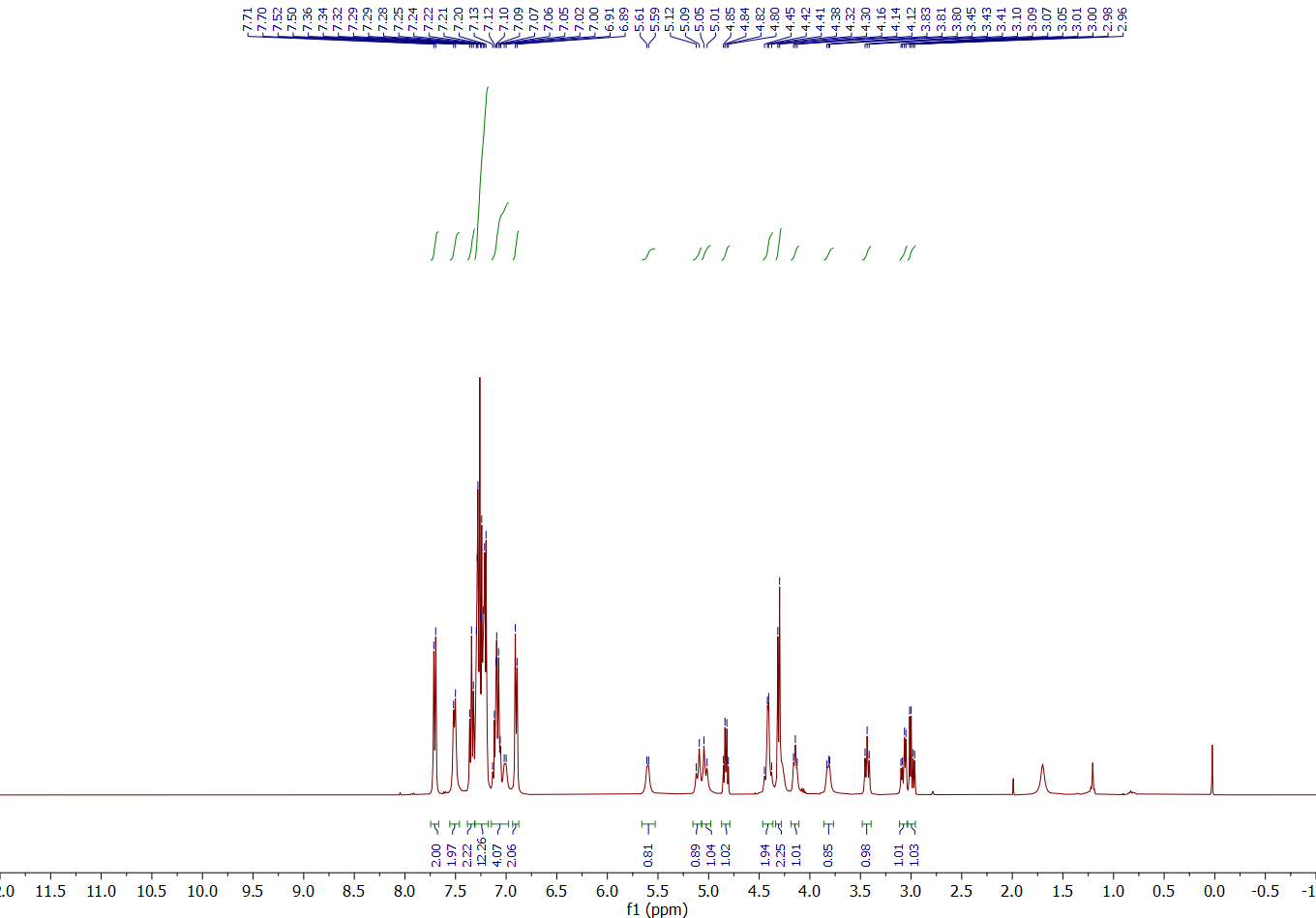


^13^C-NMR (101 MHz) in CDCl_3_


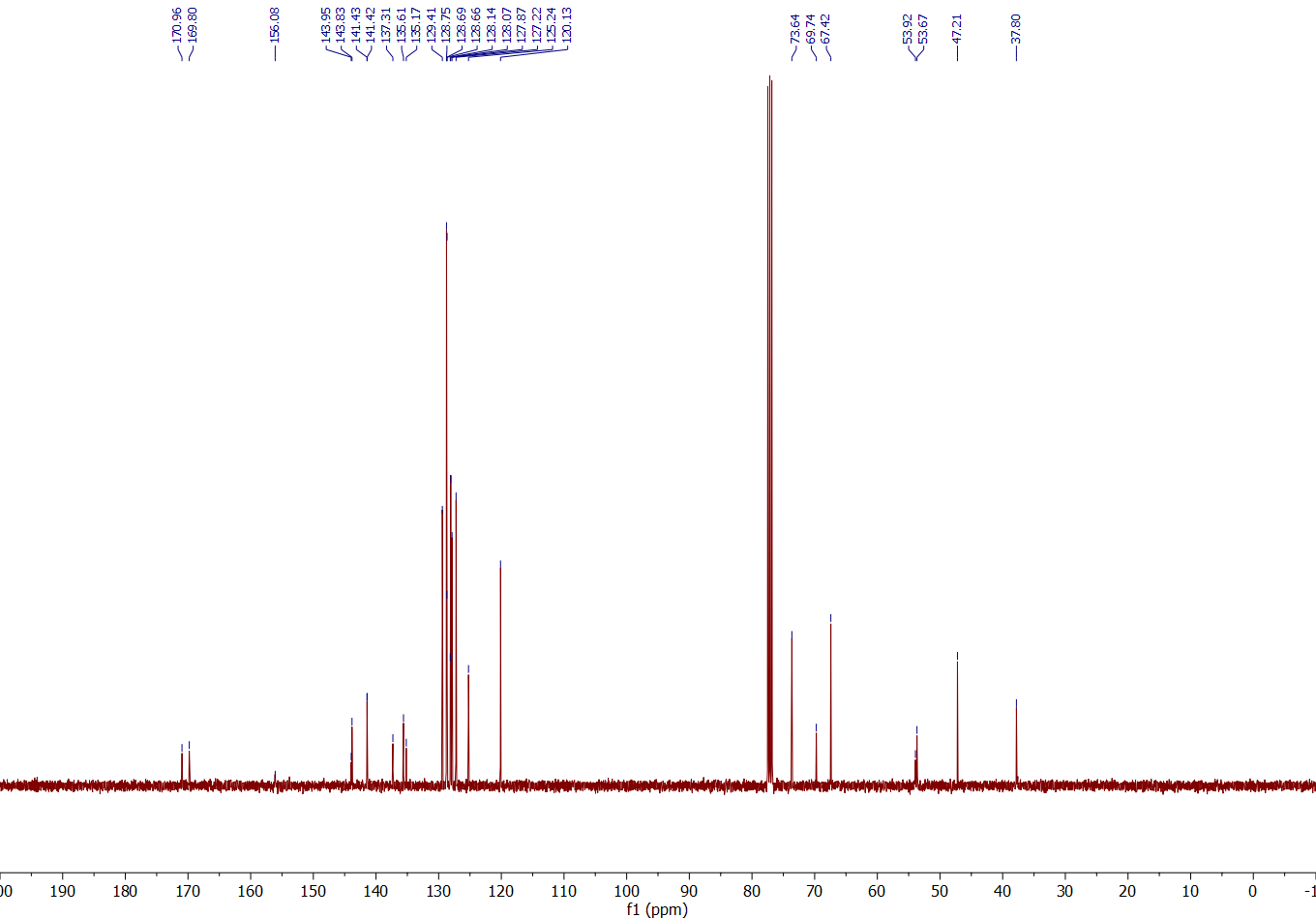


^1^H-NMR (500 MHz) in methanol-*d_4_*


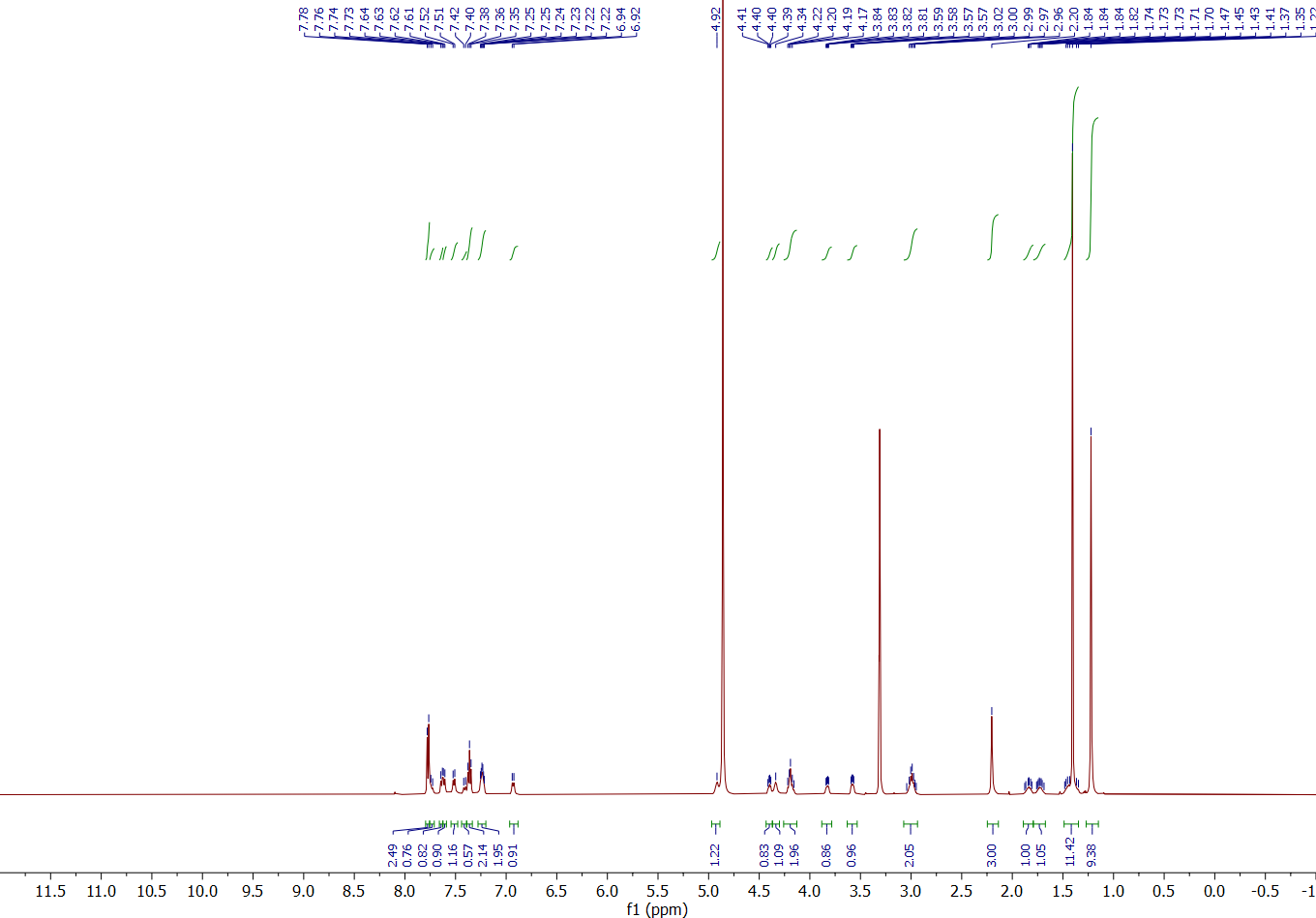


^13^C-NMR (126 MHz) in methanol-*d_4_*


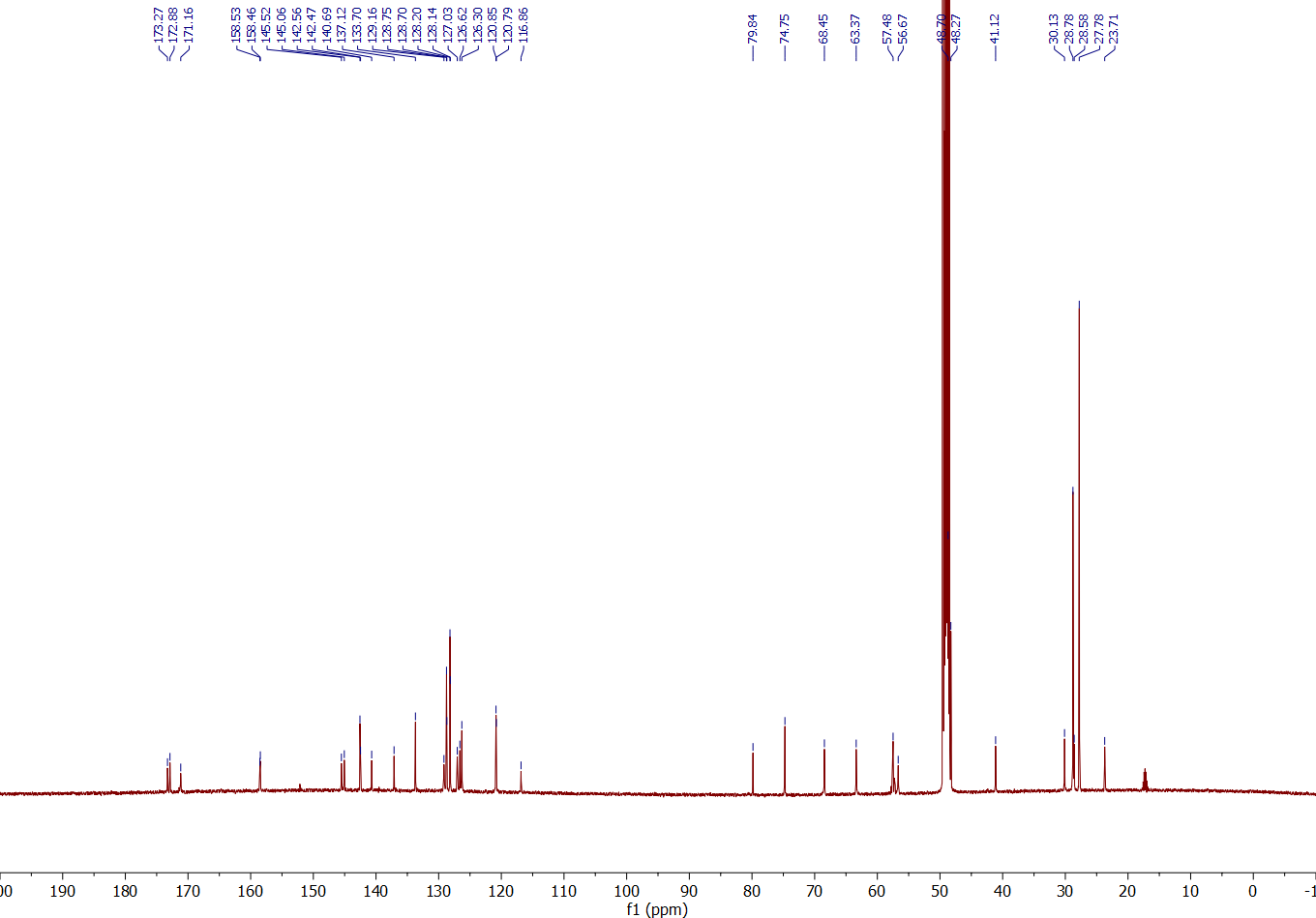


^1^H-NMR (500 MHz) in methanol-*d_4_*


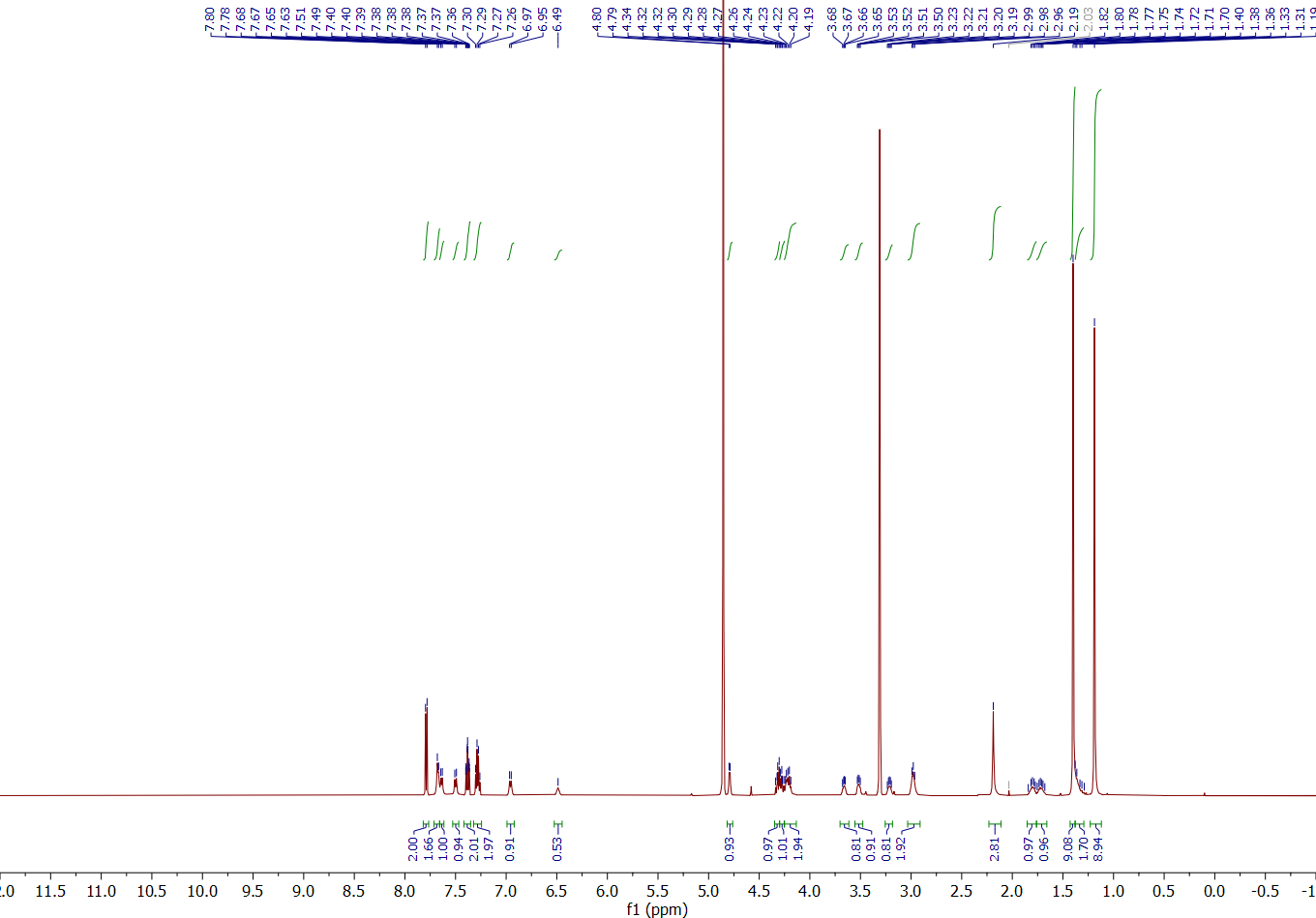


^13^C-NMR (126 MHz) in methanol-*d_4_*


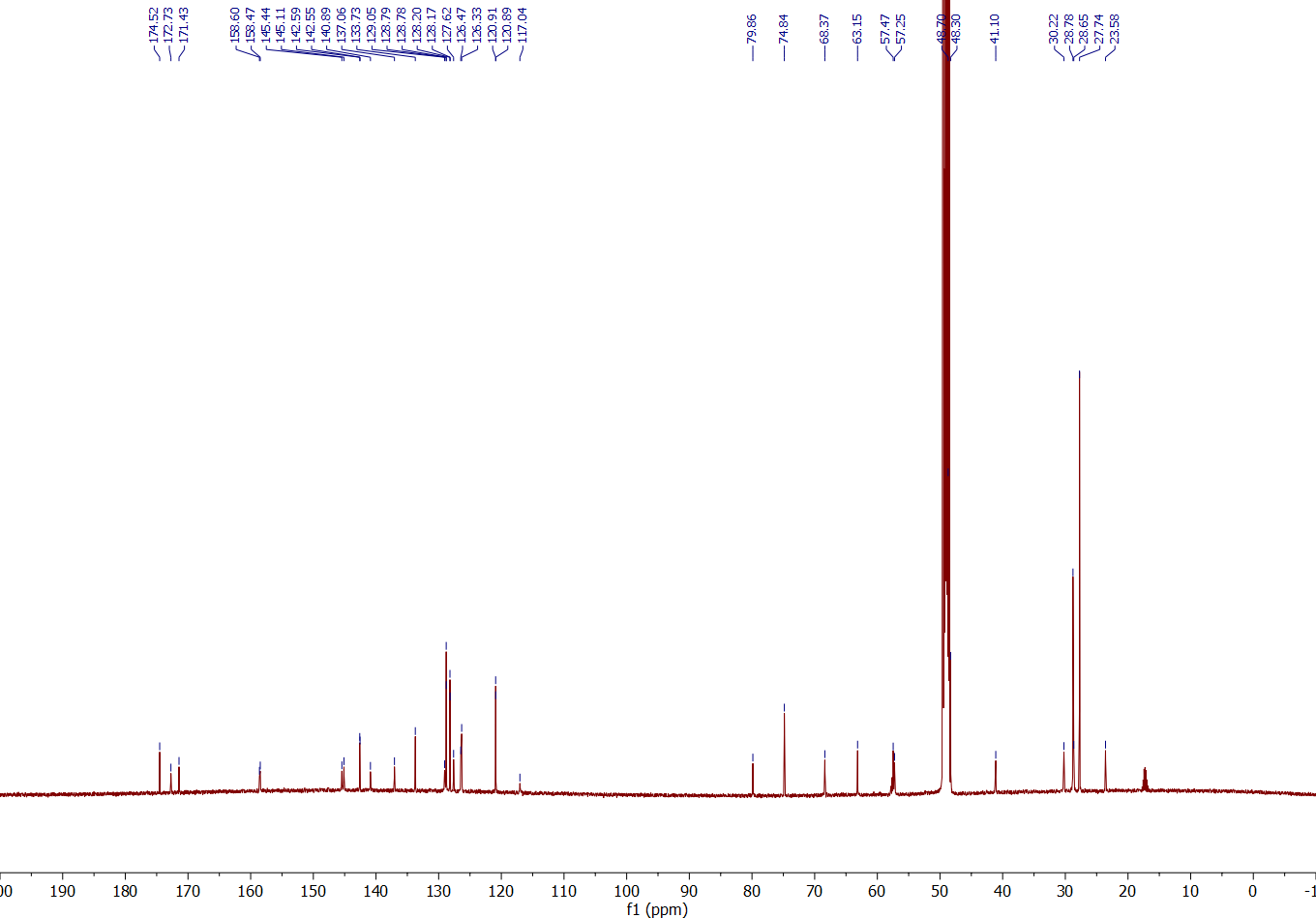


^1^H-NMR (500 MHz) in acetone-*d_6_*


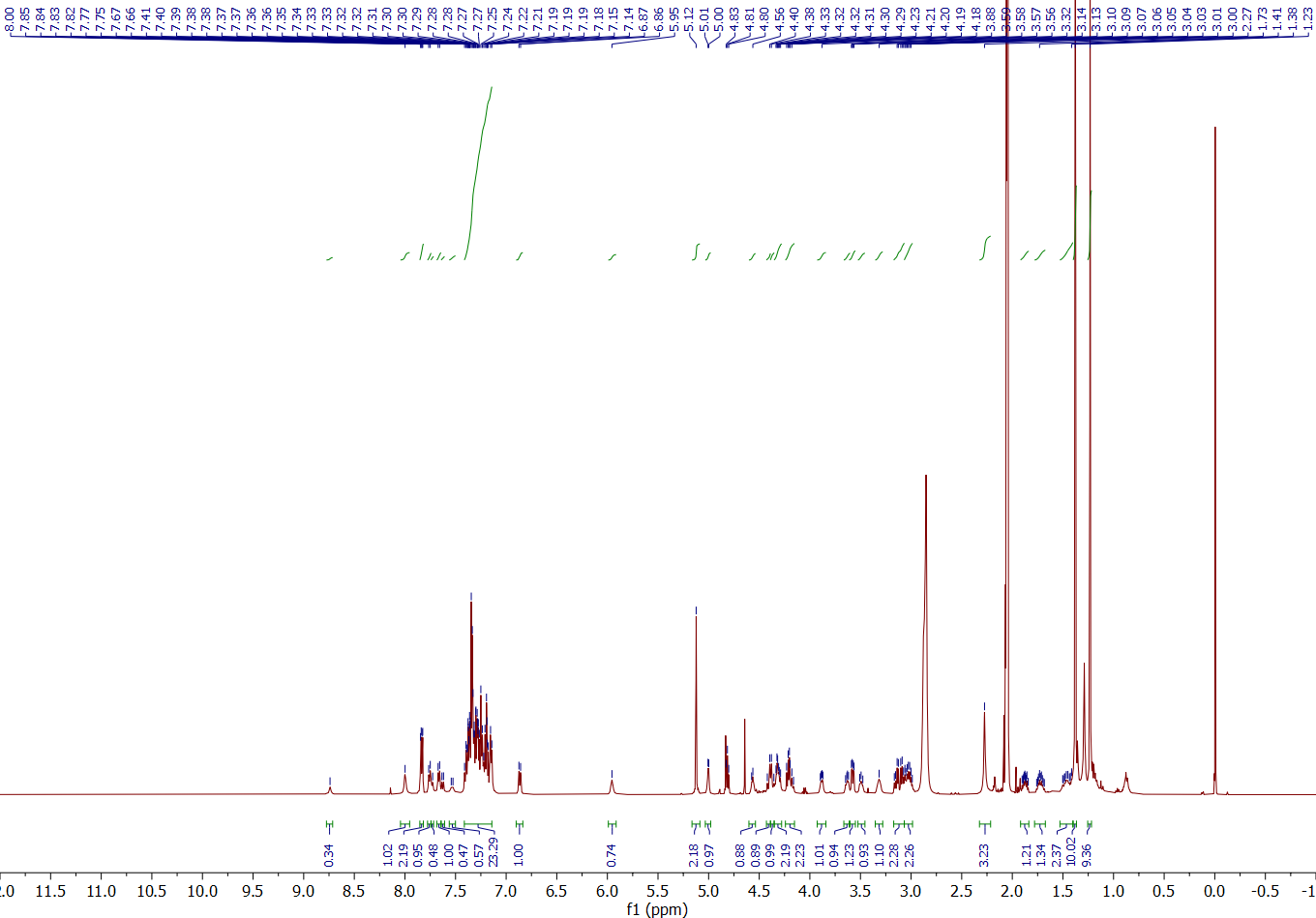


^13^C-NMR (126 MHz) in acetone-*d_6_*


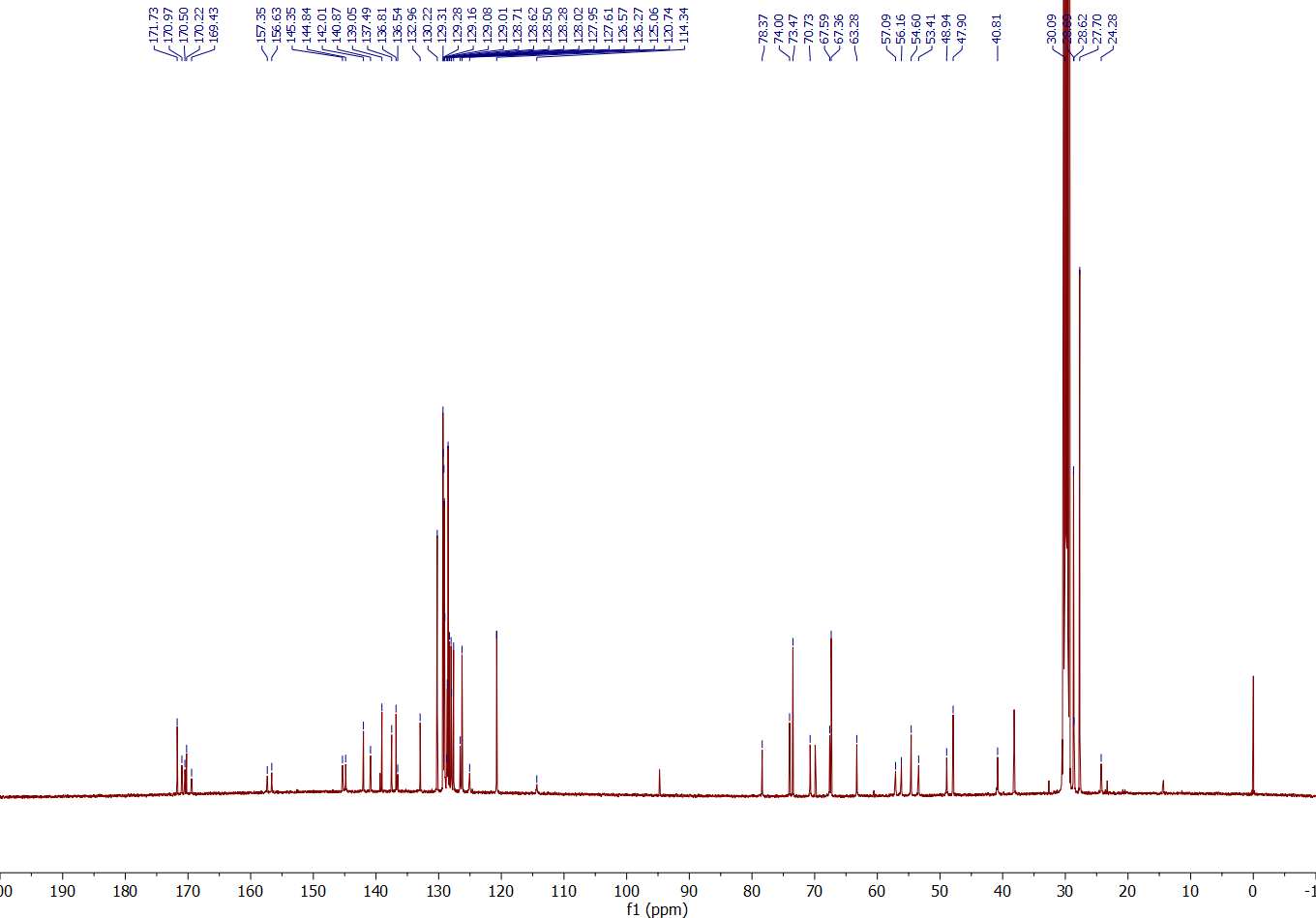


^1^H-NMR (500 MHz) in methanol-*d_4_*


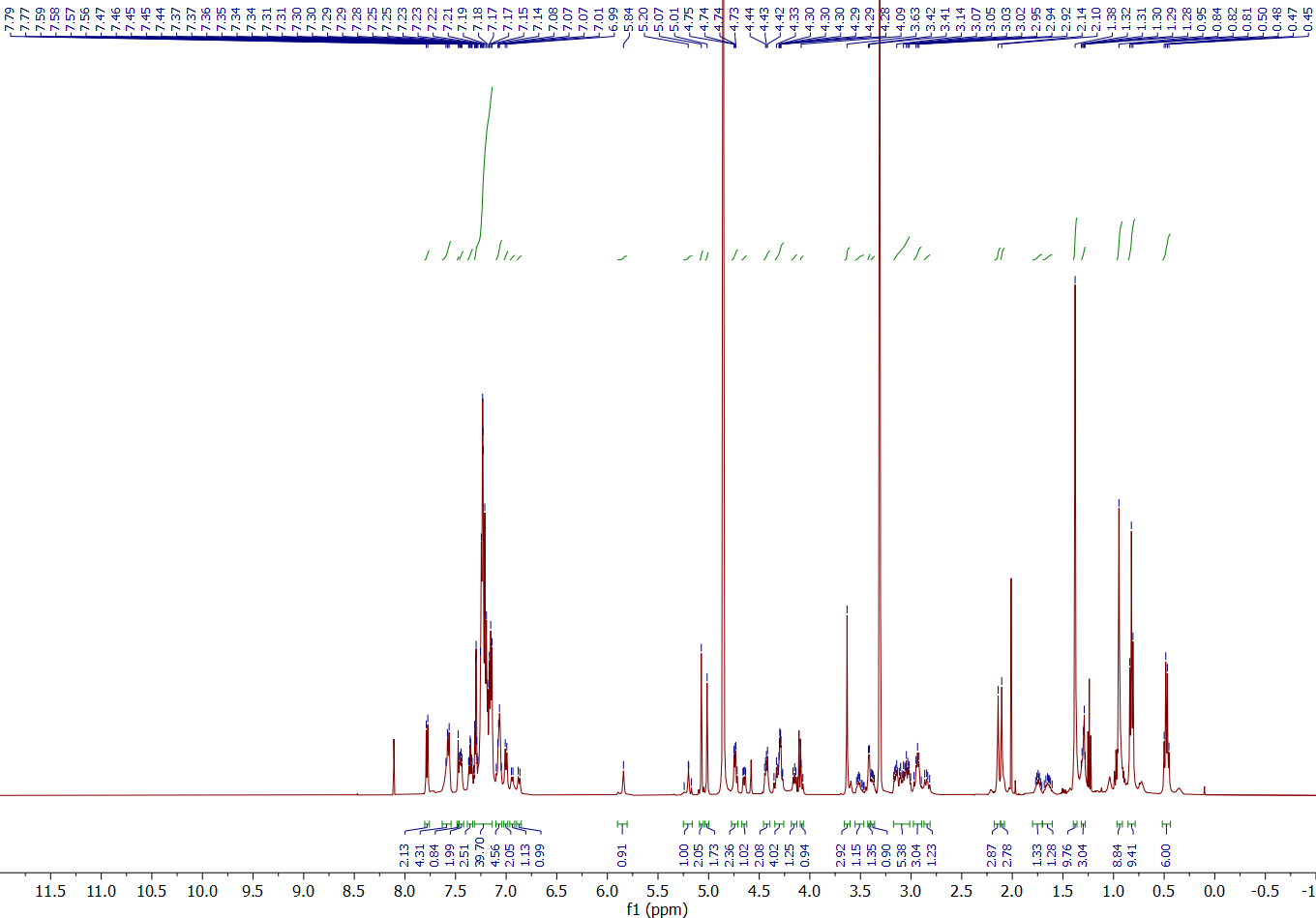


^13^C-NMR (126 MHz) in methanol-*d_4_*


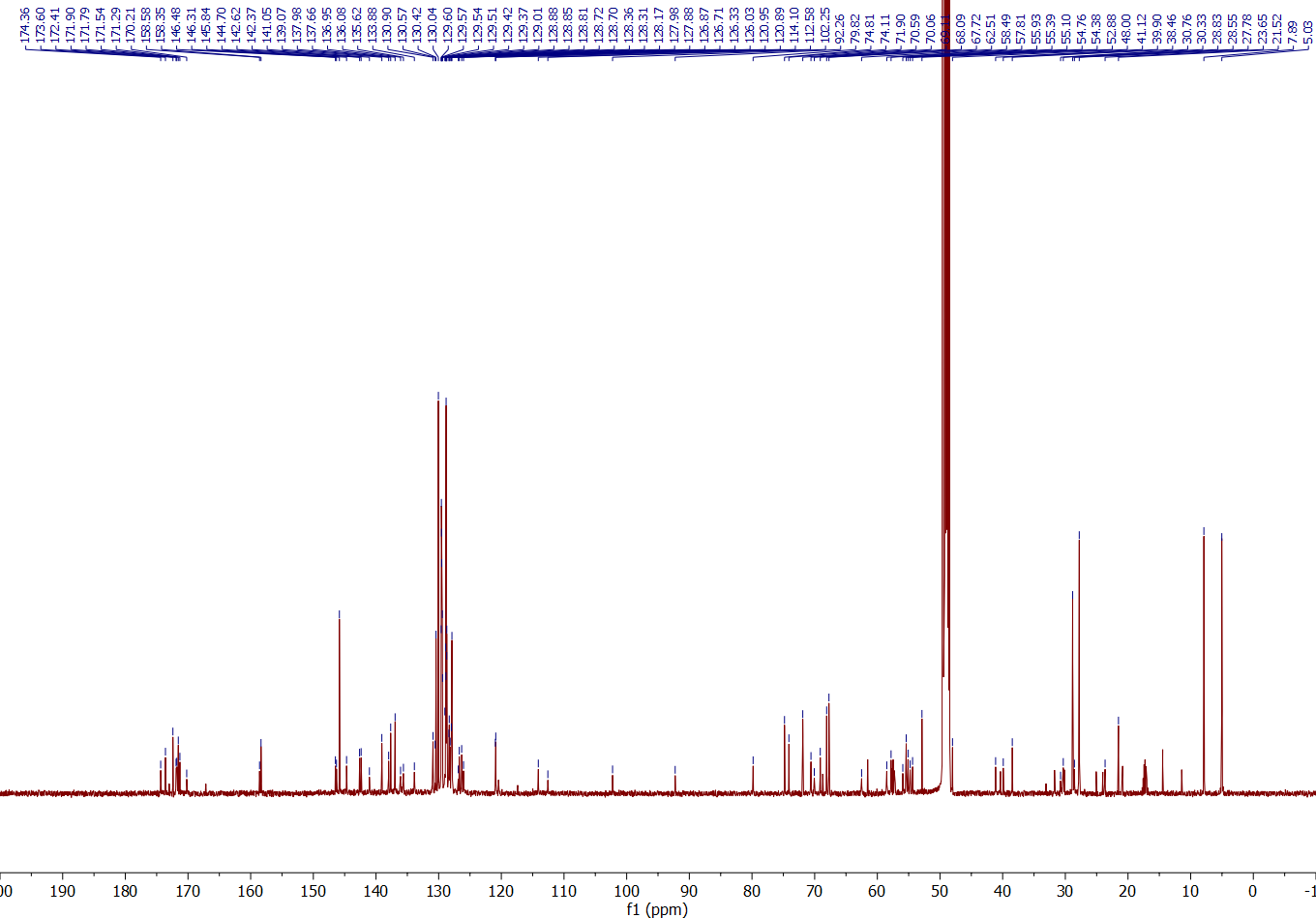


^1^H-NMR (500 MHz) in CDCl_3_


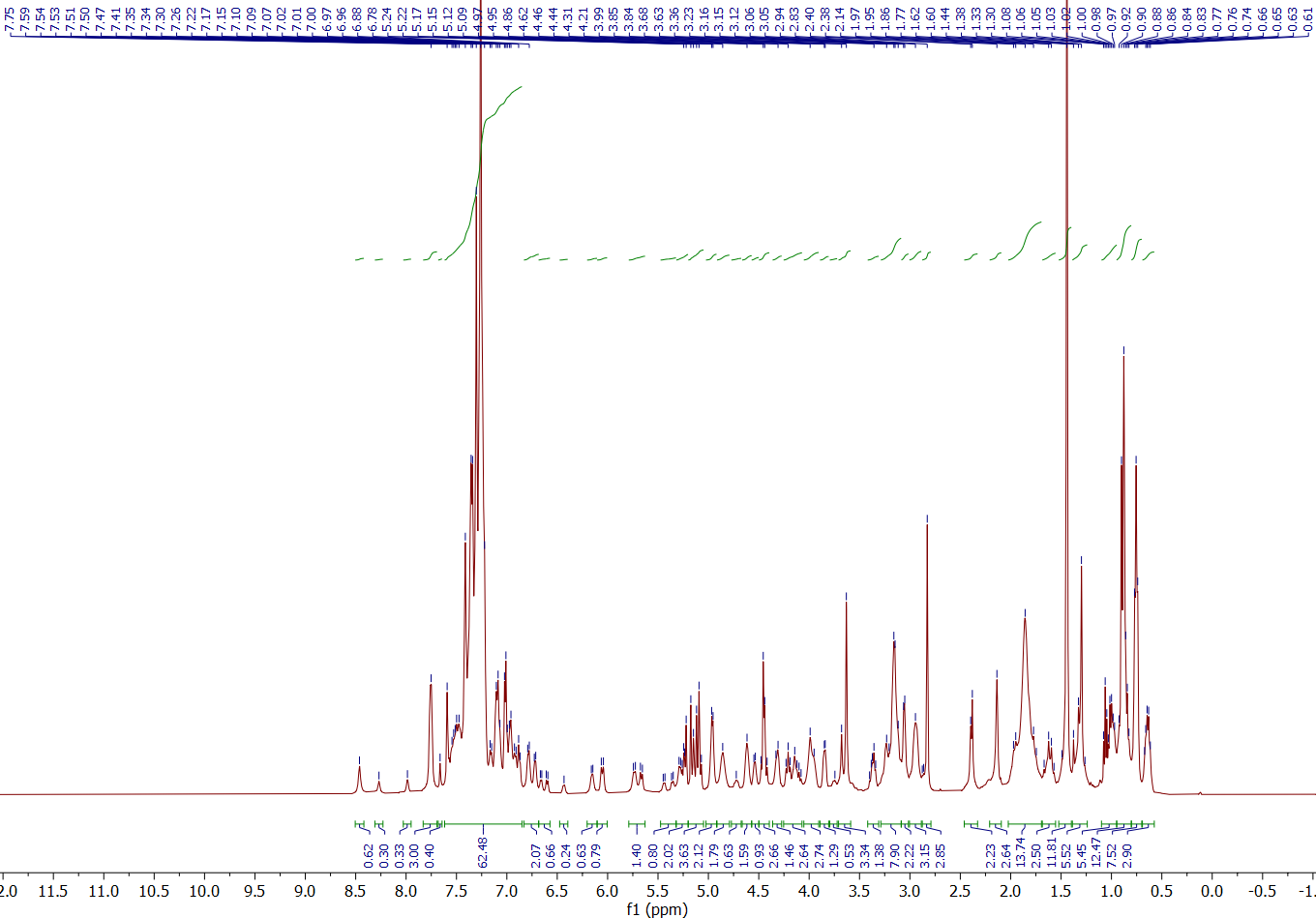


^13^C-NMR (126 MHz) in CDCl_3_


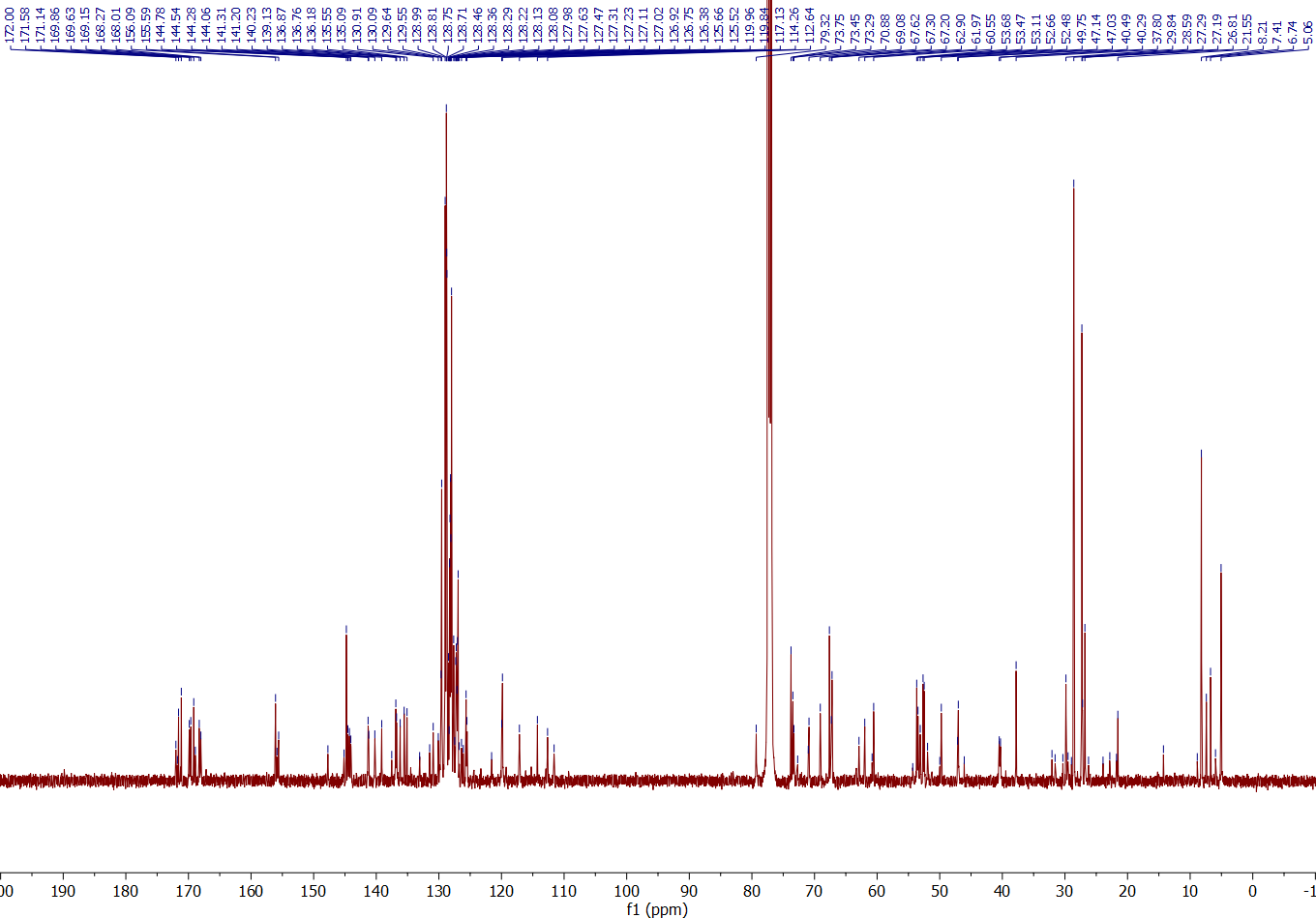


COSY-NMR for compound **35** in CDCl_3_


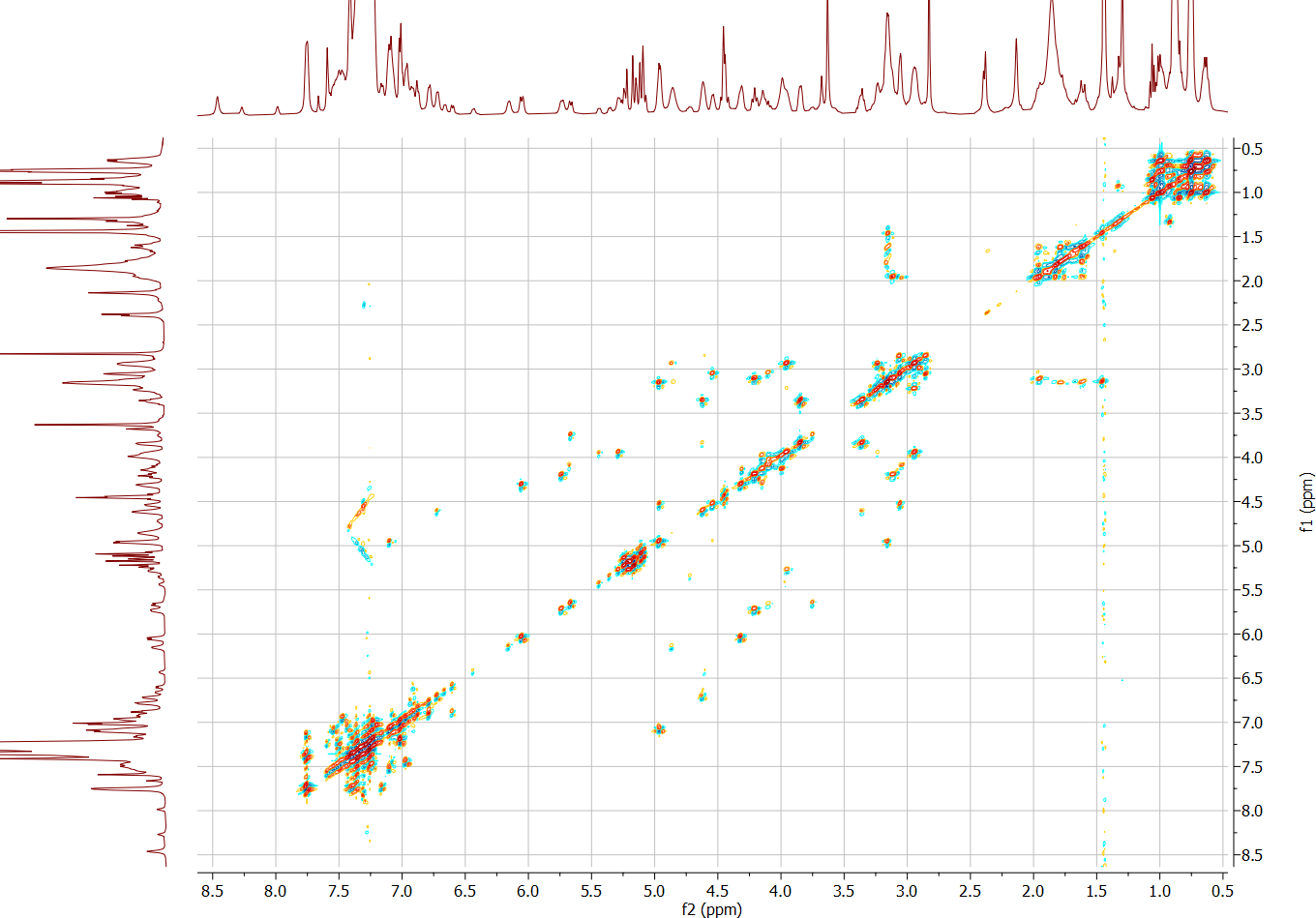


HSQC-NMR for compound **35** in CDCl_3_


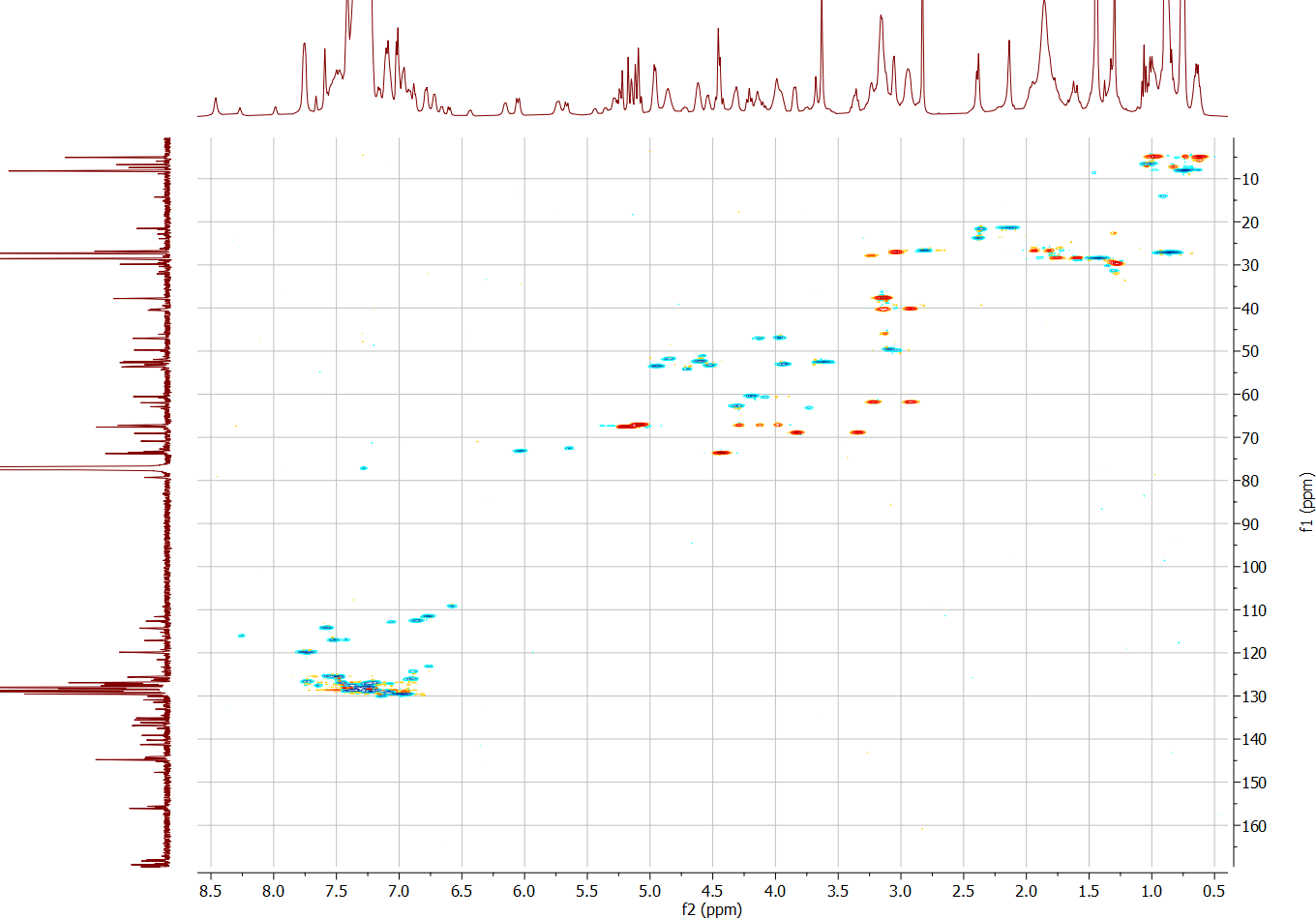


HMBC-NMR for compound **35** in CDCl_3_


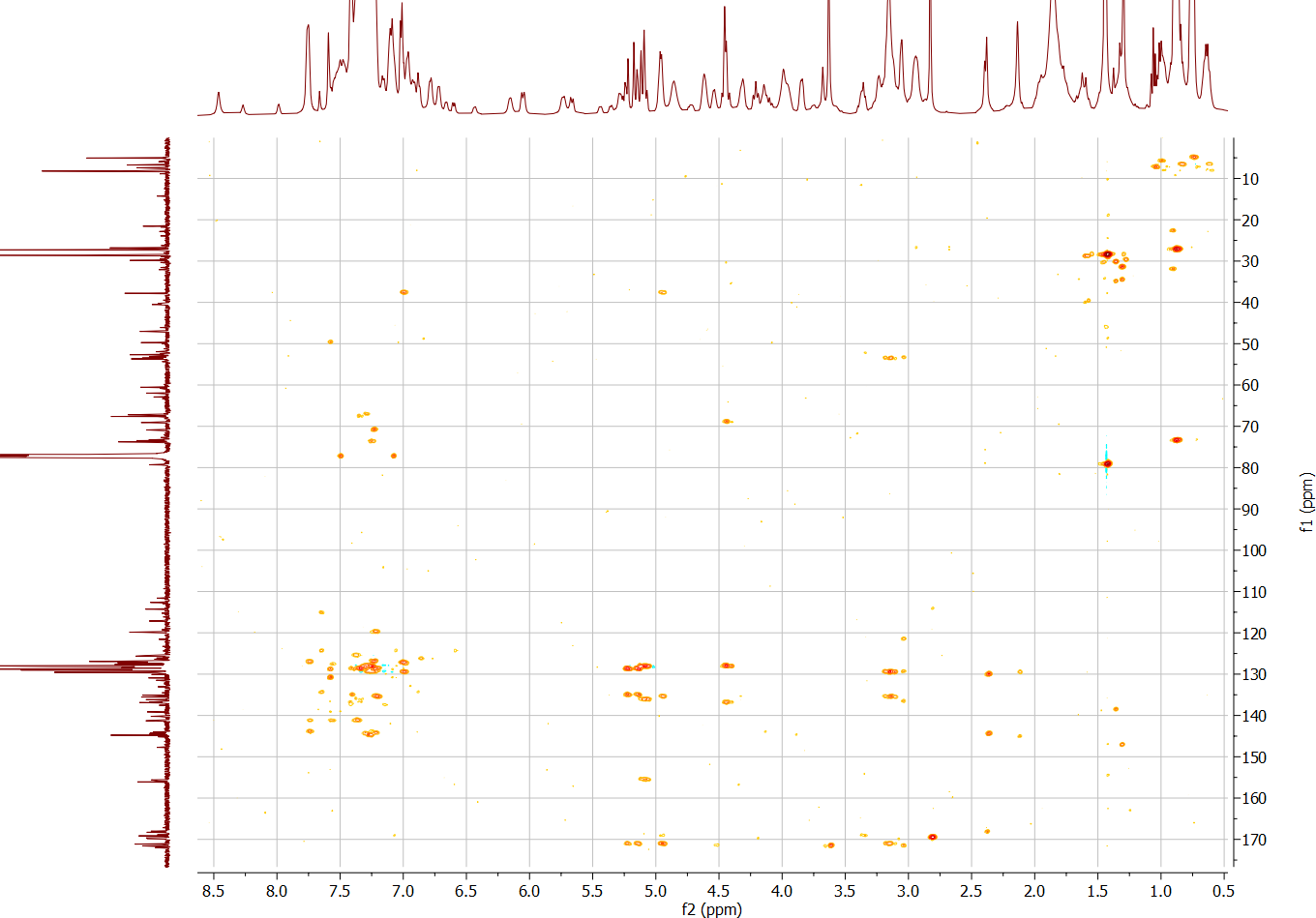


NOESY-NMR for compound **35** in CDCl_3_


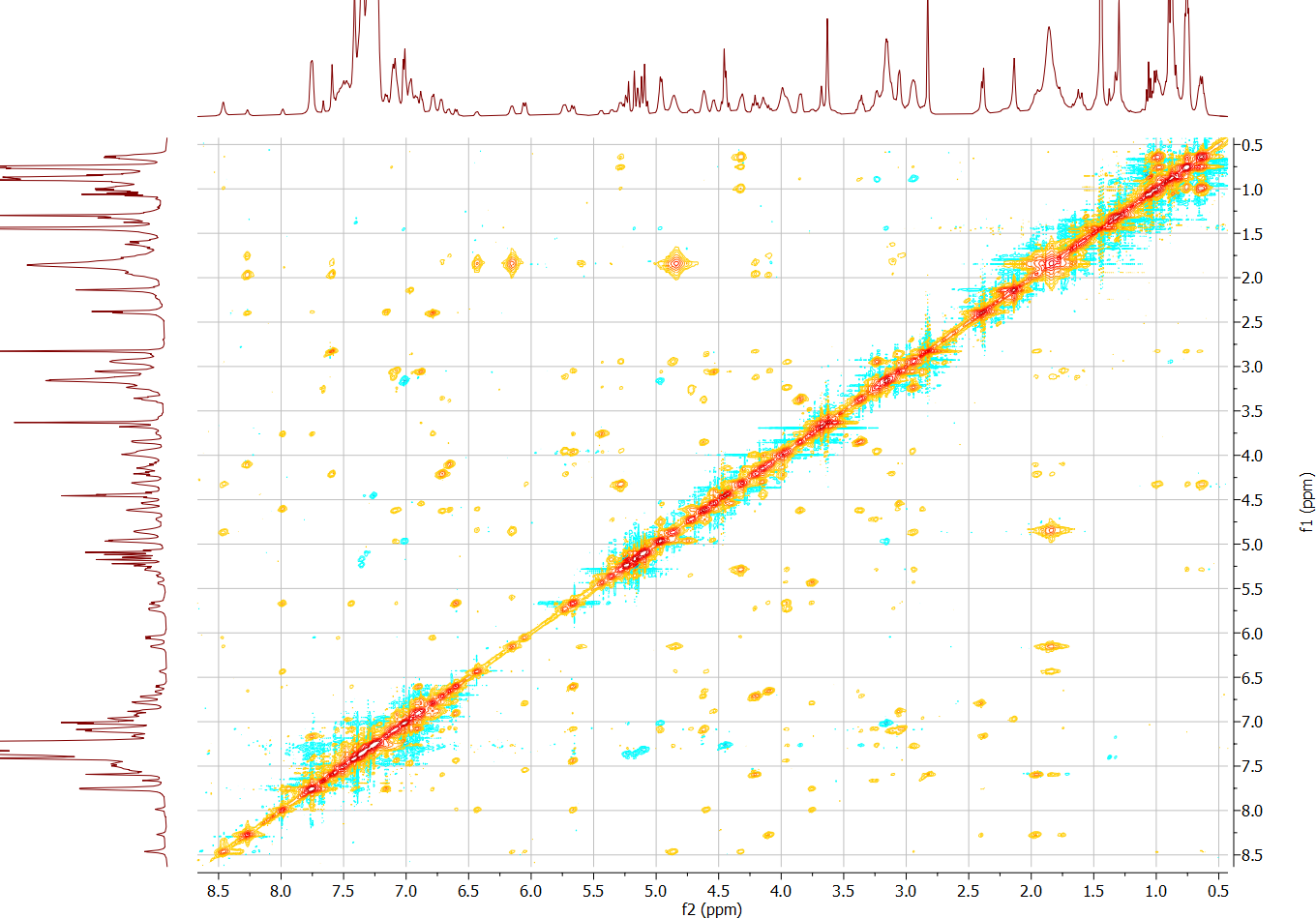


^1^H-NMR (500 MHz) in CDCl_3_

^13^C-NMR (126 MHz) in CDCl_3_

COSY-NMR for compound **36** in CDCl_3_

HSQC-NMR for compound **36** in CDCl_3_

HMBC-NMR for compound **36** in CDCl_3_

NOESY-NMR for compound **36** in CDCl_3_

^1^H-NMR (500 MHz, 25 °C) in DMSO-*d_6_*

^1^H-NMR (500 MHz, 80 °C) in DMSO-*d_6_*

^13^C-NMR (126 MHz, 25 °C) in DMSO-*d_6_*

^13^C-NMR (126 MHz, 80 °C) in DMSO-*d_6_*

^1^H-NMR (500 MHz) in DMSO-*d_6_*

^13^C-NMR (126 MHz) in DMSO-*d_6_*

^1^H-NMR (400 MHz) in methanol-*d_4_*

^13^C-NMR (126 MHz) in methanol-*d_4_*

^1^H-NMR (500 MHz) in methanol-*d_4_*

^13^C-NMR (126 MHz) in methanol-*d_4_*

^1^H-NMR (500 MHz) in methanol-*d_4_*

^13^C-NMR (101 MHz) in methanol-*d_4_*

^1^H-NMR (400 MHz) in methanol-*d_4_*

^13^C-NMR (126 MHz) in methanol-*d_4_*

^1^H-NMR (500 MHz) in methanol-*d_4_*

^13^C-NMR (126 MHz) in methanol-*d_4_*

^1^H-NMR (500 MHz) in methanol-*d_4_*

^13^C-NMR (126 MHz) in methanol-*d_4_*

^1^H-NMR (500 MHz) in methanol-*d_4_*

^13^C-NMR (126 MHz) in methanol-*d_4_*

^1^H-NMR (500 MHz) in CDCl_3_

^13^C-NMR (126 MHz) in CDCl_3_

^1^H-NMR (500 MHz) in CDCl_3_

^13^C-NMR (126 MHz) in CDCl_3_

1. most signals in the ^13^C-NMR spectrum were split at room temperature due to rotamerism. [↑](#footnote-ref-1)
2. because of low stability of the product **SI-2**, it was used directly in the next steps without further purification and was not fully characterized. [↑](#footnote-ref-2)
3. some signals in the ^1^H-NMR spectrum were split at room temperature due to rotamerism. [↑](#footnote-ref-3)
4. signal only detected at room temperature. [↑](#footnote-ref-4)
5. **SI-33** and **SI-34** were separated for analytical purposes but were employed in the next step as a mixture. [↑](#footnote-ref-5)
6. due to a very low concentration of the measured sample, some signals of quaternary carbons could not be detected in the ^13^C-NMR spectrum. [↑](#footnote-ref-6)
